# Dual fluorescent phenanthridinones and crinasiadine derivatives by consecutive palladium-catalyzed three-component syntheses

**DOI:** 10.1039/d5ra06934c

**Published:** 2025-12-10

**Authors:** Regina Kohlbecher, Thomas J. J. Müller

**Affiliations:** a Heinrich Heine University Duesseldorf, Faculty of Mathematics and Natural Sciences, Institute of Organic Chemistry and Macromolecular Chemistry Universitätstrasse 1 Düsseldorf D-40225 Germany ThomasJJ.Mueller@hhu.de

## Abstract

The sequential concatenation of Buchwald–Hartwig amination, Suzuki coupling, and lactamization in a consecutive palladium-catalyzed three-component synthesis provides direct access to functionalized *N*-arylsubstituted phenanthridinones and alkaloid-analogous crinasiadines starting from simple, readily available *ortho*-bromoanilines. A modified reaction sequence, consisting of Suzuki coupling, amide bond formation (lactamization), and subsequent alkylation at the amide nitrogen, provides *N*-alkylsubstituted phenanthridinones and crinasiadines, including two natural products, which are known for their cytotoxic activity against various cancer cell lines. A comprehensive investigation of the photophysical properties reveals dual emission of the *N*-aryl substituted derivatives, characterized by locally excited states (LE band) and intramolecular charge transfer states (CT band). Quantum chemical calculations rationalize the dual emission and suggest that the LE band derives from the *pseudo-N-intra* conformation, whereas the CT band arises from the *pseudo-N-extra* conformation in the excited state.

## Abstract 2

Sequential Pd-catalysis concatenates arylation, lactamization, and amination in a one-pot fashion to diverse emissive phenanthridinones. Dual emission is due to transition from a locally excited state and a charge transfer excited state (TDDFT).

## Graphical abstract

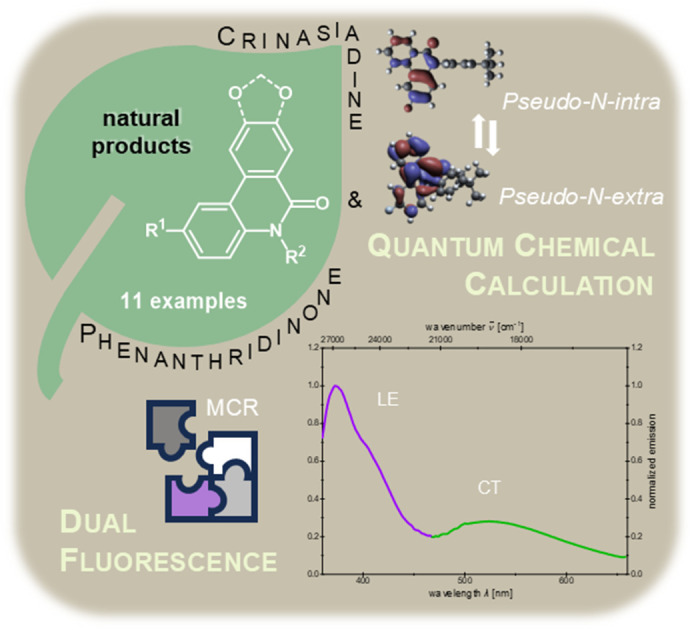

## Introduction

Phenanthridinones are major heterocyclic building blocks that occur in a variety of complex natural products and pharmaceuticals, exhibiting a broad spectrum of pharmacological activities.^1,2^ The significance of their scaffolds has aroused great interest in synthetic and medicinal chemistry to design new methodologies and novel derivatives of pharmaceutical relevance.^3^

**Fig. 1:**
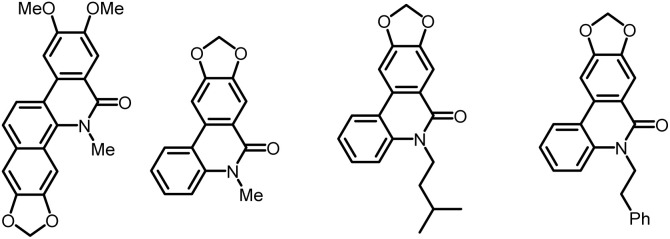
Phenanthridinone-based natural products.

The access to phenanthridinones is facilitated through a wide range of methods, encompassing both traditional and innovative strategies.^3^ Gräbe and Wander first synthesized 6(5*H*)-phenanthridinone in 1893, starting from 2′-aminobiphenyl-2-carboxylic acid.^4^ Later, numerous additional methods, such as the alkaline hydroxylation of phenanthridine,^5^ Beckmann rearrangement,^6^ or anomalous Schmidt reaction,^7^ were reported. However, these classical methods have limitations as they often require multistep syntheses of the starting materials and generally result in low to moderate overall yields. Therefore, the quest for methods using environmentally benign chemicals, energy efficient microwave-assisted reactions, and catalytic processes has become increasingly interesting.^3^ In 2013, Tanimoto *et al.* established an efficient one-step synthesis of *N*-unsubstituted phenanthridinones, including the natural product phenaglydon.^8^ This method implements 2-halobenzoates and 2-aminophenylboronic acids in a Pd-catalyzed Suzuki coupling reaction.^3,8^ The phenanthridinone scaffold is a frequently occurring structural motif in bioactive plant alkaloids.^3,9^ For instance, the phenanthridinone-based alkaloid oxynitidine was isolated from the methanol extracts of the root bark and wood of the climbing plant *Zanthoxylum nitidum* (Fig. 1, left).^10–12^ These dried plant extracts are known for their anti-inflammatory, analgesic, antimicrobial, and anticancer properties in traditional Chinese medicine.^11,13^ The scaffold is also found in various Amaryllidaceae alkaloids, which exhibit antiproliferative effects on cancer cells.^14–18^ For instance, three crinasiadines (Fig. 1) were isolated from the Amaryllidaceae plant *Zephyranthes candida*, where *N*-phenethylcrinasiadine exhibits the strongest cytotoxicity against leukemia, lung, and colon cancer cell lines.^19^

Recent research highlights the broad pharmacological potential of phenanthridinones, including anticancer activity,^20^ PARP1 inhibition,^21,22^ cardioprotective effects,^23,24^ regulation of cholesterol metabolism,^25–28^ topoisomerase I inhibition,^3,29^ and estrogen receptor modulation.^30^

Phenanthridinone derivatives are also gaining increasing interest in the field of organic electronics.^31–33^ In particular, the photophysical properties of novel phenanthridinones have only scarcely been recognized so far, as they can provide valuable information on SAR, especially since some are known for their dual emission behavior.^34,35^

Multicomponent reactions (MCRs) of functional chromophores enable rapid and diversity-oriented syntheses of compound libraries.^36–38^ By definition, MCRs allow for the synthesis of target structures from three or more reactants in a single reaction vessel, ensuring that significant parts of the employed starting materials are incorporated into the final product while drastically reducing the operational effort required for isolation.^36–41^

Recently, the concatenation of Suzuki coupling and Buchwald–Hartwig amination in a consecutive sequentially palladium-catalyzed MCR has successfully paved avenues to libraries of functionalized phenothiazines, carbazoles, indoles, and triarylamines.^42^ Herein, we report a novel, diversified, and sequentially palladium-catalyzed MCR that allows direct access to functionalized phenanthridinones as well as alkaloid-analogous crinasiadines starting from simple, readily available starting materials. Additionally, investigations of the photophysical properties and quantum chemical calculations open up a deeper understanding of the electronic structure of phenanthridinone derivatives.

## Results and discussion

### Syntheses

**Scheme 1:**
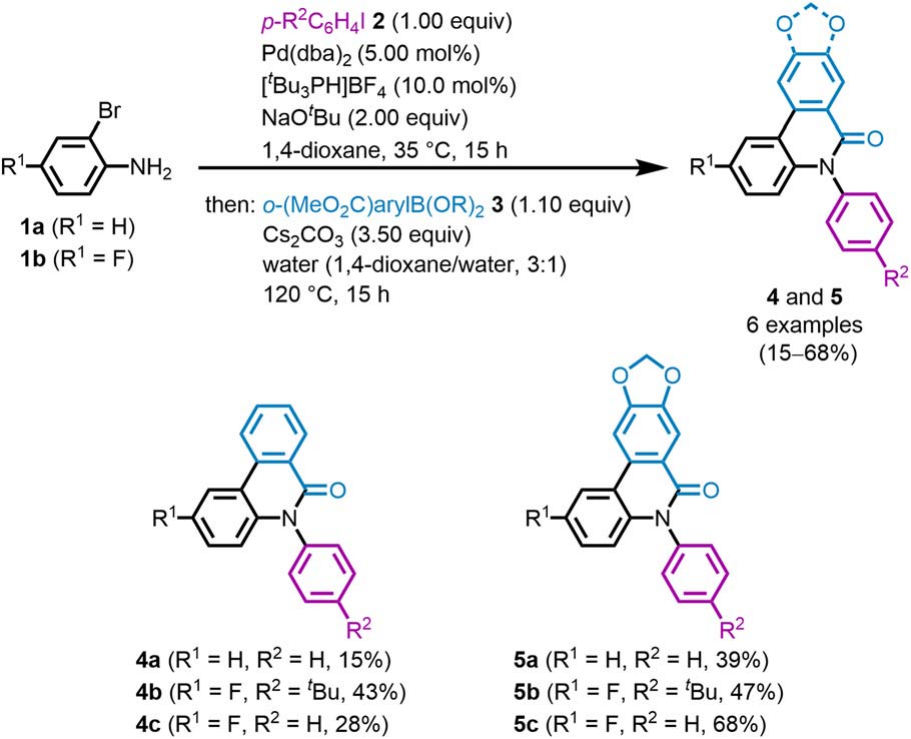
BHSL synthesis of *N*-arylphenanthridinones 4 and *N*-arylcrinasiadines 5 (all reactions were performed on a 0.5 mmol scale in 1,4-dioxane (3.5 mL) and yields are given after purification by flash chromatography on silica gel).

Starting from *ortho*-bromoaniline 1, a palladium-catalyzed three-component synthesis was envisioned to provide direct access to phenanthridinones and crinasiadines. After comprehensive optimization (for details see SI, chpt. 5.1), a novel Buchwald-Hartwig amination-Suzuki coupling-lactamization sequence (BHSL-sequence) was developed, based on a previously optimized, highly diversified, sequentially palladium-catalyzed one-pot process for the synthesis of *para*-biaryl-substituted triarylamines (*p*-bTAA).^43^ This approach aims to construct a substance library of diverse phenanthridinone derivatives in a modular manner. By switching the Buchwald-Hartwig amination and the Suzuki coupling compared to the *p*-bTAA synthesis,^43^ the formation of poorly soluble 6(5*H*)-phenanthridinone intermediates could be circumvented, enabling the successful synthesis of the target molecules. Due to their reactivity, the first step of the Buchwald–Hartwig amination requires an aryl iodide 2 and an *ortho*-bromoaniline 1 to exclude homocoupling of the aniline.^44^ Additionally, the aryl iodide should only be employed in one equivalent to avoid twofold arylation of the aniline nitrogen. Furthermore, anhydrous conditions have to be ensured in this step, as traces of water might convert NaO^*t*^Bu into sodium hydroxide, thereby reducing reaction efficiency due to its lower basicity.^45^ In the next step, cyclization between the aniline intermediate and a 2-(ethoxycarbonyl)arylboronic acid 3 has to be initiated. In addition to the Suzuki coupling, the *N*-arylaniline as a nucleophile should attack the ethyl ester to form the lactam. The addition of water and Cs_2_CO_3_ in this step suppresses the formation of the undesired dihydrophenazine byproduct (see SI). Ultimately, *ortho*-bromoanilines 1 and aryl iodides 2 are successfully subjected to Buchwald–Hartwig amination in the presence of catalytic amounts of Pd(dba)_2_ and [^*t*^Bu_3_PH]BF_4_, as well as an excess of NaO^*t*^Bu, at 35 °C for 15 h. The subsequent addition of 2-(methoxycarbonyl)arylboronic acids 3, Cs_2_CO_3_, and water at 120 °C for 15 h induces cyclization, affording after flash chromatography three *N*-arylphenanthridinones 4 and three *N*-arylcrinasiadines 5 in moderate to good yields ranging from 15 to 68% (Scheme 1). This corresponds to a yield per bond formation of up to 89%. The presence of a fluoro substituent and a *tert*-butyl group improves solubility and consequently leads to higher yields. The benzodioxole boronic acid ester 3b required for the preparation of *N*-arylcrinasiadines 5 can be successfully synthesized from piperonal according to a literature protocol (see SI).^46–48^ In comparison to *N*-arylphenanthridinones 4, the 1,3-benzodioxole causes an increase in yield, possibly due to improved solubility and easier purification of *N*-arylcrinasiadines 5.

**Scheme 2:**
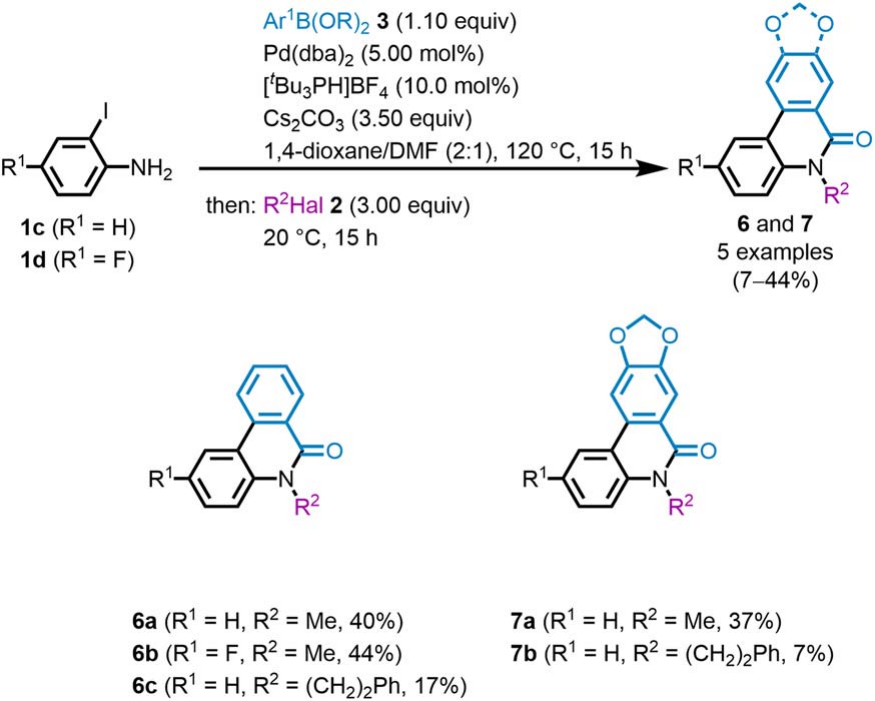
SLS sequence of *N*-alkylphenanthridinones 6 and *N*-alkylcrinasiadines 7 (all reactions were performed on a 0.5 mmol scale in 1,4-dioxane (3.5 mL) and yields are given after purification by flash chromatography on silica gel).

Furthermore, the optimized Suzuki coupling can be successfully combined with lactamization and nucleophilic substitution in a consecutive three-component reaction (see SI). Here, a solvent mixture of 1,4-dioxane and DMF in a ratio of 2 : 1 is used for the Suzuki coupling, which increases the solubility of the 6(5*H*)-phenanthridinone intermediate and allows the subsequent alkylation of the lactam nitrogen with alkyl halides 2 to proceed more efficiently. In addition, an iodoaniline 1 is used in the first step, as iodides are more reactive than bromides.^44^ The Suzuki coupling-lactamization-substitution (SLS) sequence finally provides after flash chromatography, access to three *N*-alkylphenanthridinones 6 and two *N*-alkylcrinasiadines 7 with yields ranging from 7 to 44% (Scheme 2). These include the two natural products *N*-methylcrinasiadin 7a and *N*-phenethylcrinasiadin 7b, which are known for their cytotoxicity against leukemia, lung, and colon cancer cells.^19^ The literature known syntheses to date for the preparation of 7a and 7b are multi-step syntheses, but no one-pot methodologies, *i.e.*, consecutive MCR.^19,49^ The incorporation of a fluorine substituent in derivative 6b improves solubility and consequently leads to the highest yield. In addition to methyl iodide (2c), (2-bromoethyl)benzene (2d) can also be employed in the terminal alkylation. However, the inclusion of 2d leads to a lower yield. It cannot be excluded that the presence of the palladium catalyst triggers the dehydrobromination of the substrate by competing β-H-elimination.^50^

The structures of all phenanthridinone analogues 4–7 were assigned by extensive NMR spectroscopy, mass spectrometry, and IR spectroscopy (see SI). In addition, the molecular composition of the novel compounds was determined by HPLC-HRMS (see SI).

### Photophysical properties

Although *N*-arylphenanthridinones have been shown to display dual emission,^34,35^ the ease of their accessibility through our one-pot sequence prompted us to take a closer look at the photophysics, in particular, to scrutinize the origin of the observed dual emission. This phenomenon was attributed to a conformational change of the *N*-aryl substituent as supported by solvatochromism and time-resolved fluorescence studies.^35^ Alternatively, an anti-Kasha behavior was discussed, which involves emission from higher excited states as substantiated by quantum chemical calculations.^34^ Therefore, the photophysical properties of all compounds were investigated by UV/vis absorption and fluorescence spectroscopy in dichloromethane or acetonitrile (Table 1).

**Table 1:** Selected photophysical properties (absorption maxima in solution with absorption coefficients *ε* and emission maxima in solution with fluorescence quantum yields *Φ*_em_ and Stokes shifts Δ*ν̃*_s_) of all phenanthridinones 4–7

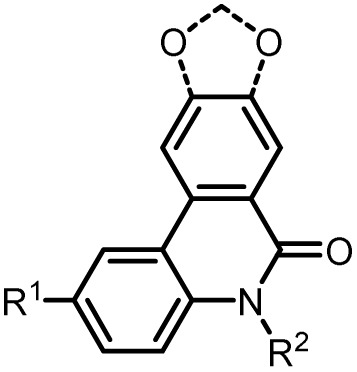					
Compound	R^1^	R^2^	Solvent	*λ*_max,abs_ a [nm] (*ε* [m^−1^ cm^−1^])	*λ*_max,em_ b [nm],/Δ*ν̃*_s_^d^ [cm^−1^] (*Φ*_em_)^c^
4a	H	Ph	CH_2_Cl_2_	232 (35 300), 237 (35 000), 260 (13 200), 306 (7200), 322 (7300), 336 (5700)	372/2900, 524/10 700
4b	F	*p*-^*t*^BuC_6_H_4_	CH_2_Cl_2_	237 (34 700), 261 (13 000), 273 (sh, 8100), 319 (5400), 331 (7200), 345 (sh, 6100)	382/2800, 530/10 100 (0.01)
			MeCN	243 (sh, 40 700), 248 (44 100), 255 (sh, 41 900), 281 (sh, 14 100), 291 (sh, 9400), 329 (sh, 8300), 348 (8400), 363 (sh, 7100)	376/2600, 579/10 300
4c	F	Ph	CH_2_Cl_2_	236 (41 000), 260 (15 100), 272 (sh, 9600), 314 (6100), 329 (8900), 343 (7400)	374/2400, 548/10 900
5a	H	Ph	CH_2_Cl_2_	249 (4800), 270 (15 700), 286 (sh, 10 900), 296 (sh, 9700), 311 (sh, 11 500), 318 (11 500), 339 (6600)	367/2100, 519/12 200
5b	F	*p*-^*t*^BuC_6_H_4_	CH_2_Cl_2_	249 (43 400), 274 (13 100), 287 (sh, 10 500), 298 (sh, 8200), 317 (sh, 9700), 329 (10 300), 346 (8000)	372/2000, 550/10 700 (0.02)
			MeCN	247 (35 600), 268 (35 500), 292 (sh, 10 200), 306 (sh, 8100), 316 (sh, 6700), 328 (sh, 8400), 345 (6400), 365 (6300)	372/2100, 562/9600
5c	F	Ph	CH_2_Cl_2_	248 (16 900), 273 (5200), 287 (4200), 299 (3400), 316 (sh, 3800), 329 (3900), 346 (5300)	371/2000, 543/10 500
6a	H	Me	CH_2_Cl_2_	233 (49 200), 239 (50 000), 262 (18 600), 276 (sh, 9000), 312 (sh, 7000), 325 (9700), 339 (7600)	367/2300
6b	F	Me	CH_2_Cl_2_	236 (39 500), 253 (13 100), 262 (15 700), 273 (8900), 317 (sh, 6300), 331 (9100), 346 (7700)	373/2100
6c	H	Phenethyl	CH_2_Cl_2_	234 (33 400), 240 (34 400), 252 (sh, 12 300), 262 (13 200), 275 (sh, 7100), 310 (sh, 5700), 325 (6400), 339 (5300)	367/2300
7a	H	Me	CH_2_Cl_2_	247 (45 000), 269 (17 300), 285 (sh, 10 500), 297 (sh, 9500), 313 (sh, 12 800), 319 (11 900), 324 (sh, 11 100), 340 (8700)	364/2000
7b	H	Phenethyl	CH_2_Cl_2_	249 (35 700), 270 (15 900), 286 (sh, 8400), 297 (sh, 7500), 313 (sh, 8700), 318 (8700), 325 (sh, 8100), 340 (5400)	364/2000

a Recorded at *T* = 293 K, *c* = 10^−5^m.

b Recorded at *T* = 293 K, *c* = 10^−7^m.

c Absolute over all quantum yields recorded at *T* = 293 K, *c* = 10^−6^m.

d Δ*ν̃*_s_ = 1/*λ*_max,abs_ − 1/*λ*_max,em_.

**Fig. 2:**
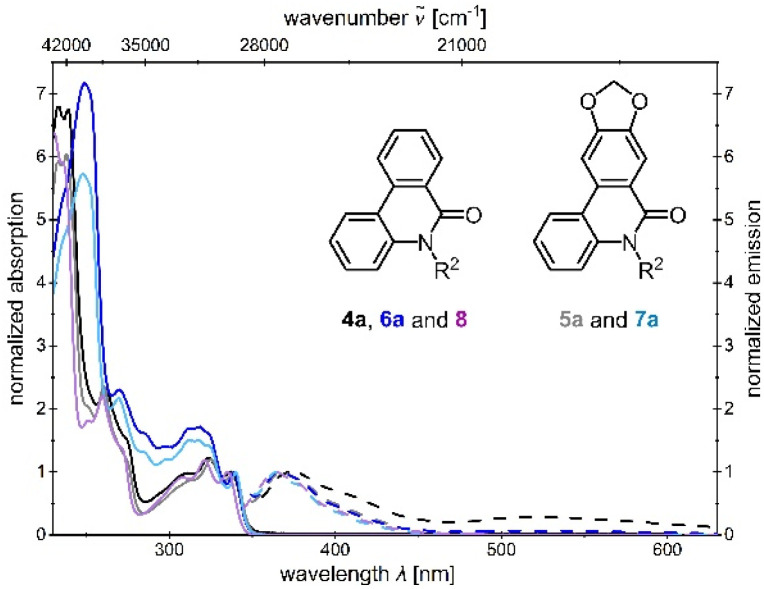
Comparison of the normalized UV/vis absorption and emission spectra of phenanthridinones 4a, 5a, 6a, 7a and 6(5*H*)-phenanthridinone 8 (absorption spectra recorded in CH_2_Cl_2_, *T* = 293 K, *c* = 10^−5^m (bold lines) and emission spectra recorded in CH_2_Cl_2_, *T* = 293 K, *c* = 10^−7^m (dashed lines)).

The *N*-aryl/*N*-alkylphenanthridinones 4 and 6 exhibit an intense absorption maximum with a shoulder at high energy in the UV. In contrast, the *N*-aryl/*N*-alkylcrinasiadines 5 and 7 display only one characteristic maximum without a shoulder in this region, which, unlike compounds 4 and 6, is slightly bathochromically shifted. The subsequent longer wavelength maxima of compounds 5 and 7 are also slightly bathochromically shifted in comparison to 4 and 6. At lowest energy, three maxima appear for each compound, which are attributed to vibronic fine structure of the same electronic transition. The longest wavelength absorption maxima of all compounds 4–7 occurring at a similar wavelength to that of the unsubstituted 6(5*H*)-phenanthridinone 8 (R=H, isolated intermediate) (Fig. 2). Therefore, the longest wavelength absorption maximum of all compounds probably corresponds to the HOMO → LUMO transition, in accordance with literature for compound 8.^35,51^

Furthermore, the absorption maxima at lower energy of the *N*-aryl/*N*-alkylphenanthridinones 4b–c and 6b, as well as the *N*-arylcrinasiadines 5b–c, are very similar and slightly bathochromically shifted compared to compounds 4a, 5a, 6a, 6c, and 7a–b devoid of fluorine substituent at position R^1^. Consequently, the substituent at position R^2^, unlike that at position R^1^, has only a minor influence on the spectra for the present derivatives. Therefore, it can be assumed that the longest wavelength absorption band is primarily associated with the phenanthridinone moiety.

**Fig. 3:**
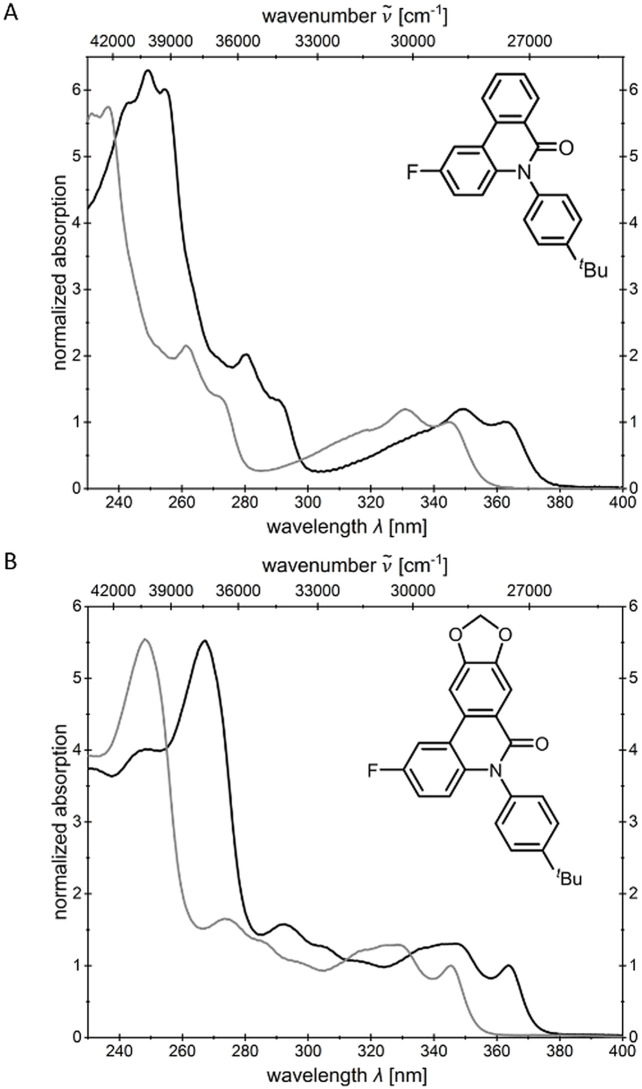
Comparison of the normalized absorption spectra of *N*-arylphenanthridinone derivatives 4b (A) and 5b (B) in acetonitrile (black lines) and dichloromethane (gray lines) (*T* = 293 K, *c* = 10^−5^m).

The polarity of the solvents influences the position of the absorption maxima, as illustrated by the bathochromic shift observed in acetonitrile (Table 1). This suggests that the absorption bands cannot be attributed solely to a locally excited state (LE band), but rather also possess partial charge transfer (CT) character. The shapes of the absorption bands remain largely unchanged, which indicates a structurally similar electronic transition in both solvents (Fig. 3).

**Fig. 4:**
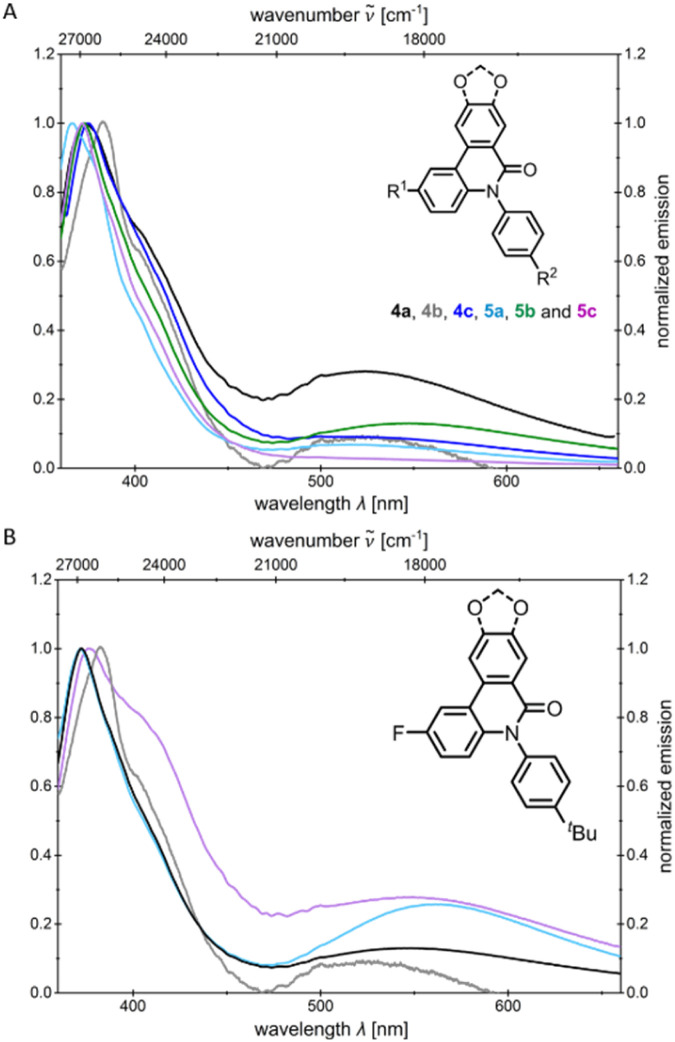
Normalized emission spectra of *N*-arylphenanthridinone derivatives 4–5 in CH_2_Cl_2_ (A) and comparison of emission spectra of 4b and 5b in CH_2_Cl_2_ and of 4b and 5b in MeCN (B) (emission spectra recorded in CH_2_Cl_2_ or MeCN, *T* = 293 K, *c* = 10^−7^m).

The emission spectra of the *N*-arylsubstituted compounds 4 and 5 show two emission maxima, in contrast to those of the *N*-alkylsubstituted compounds 6 and 7, which indicate dual fluorescence (Fig. 4). The shorter wavelength emission band only displays a small Stokes shift Δν̃_s_, indicating emission from an LE band, as only minor geometric changes are likely to occur between the ground and excited states. Moreover, the shape and position of the LE band closely resemble the emission maximum of 6(5*H*)-phenanthridinone 8 (Fig. 2).^35,51^ The emission is therefore likely to originate from the phenanthridinone core.

The second emission maximum is, unlike the LE band, strongly bathochromically shifted. This maximum is only weakly significant for compounds 4c and 5c, which bear a fluorine substituent at R^1^ and no substituent at R^2^. Investigations of the emission of 4b and 5b in dichloromethane and acetonitrile reveal that the intensity of the longer wavelength emission band increases relative to the LE band with increasing solvent polarity (Fig. 4). Moreover, since in acetonitrile the LE transition hardly shifts, the insensitivity to solvent polarity (Table 1) can be attributed to a π–π* transition. In contrast, the longer wavelength emission maxima exhibit a pronounced bathochromic shift with increasing polarity form dichloromethane to acetonitrile (positive solvatochromism). This emission maximum is therefore likely a CT band, as polar solvents stabilize charge separation more effectively than nonpolar ones.^35^ However, the LE emission remains more dominant in both solvents. Independent of the excitation wavelength the emission maxima appear at the same wavelength. The degassed and non-degassed emission spectra of compounds 4b and 5b in acetonitrile differ only marginally (see SI), which likely rules out the assignment of the longest wavelength emission band to be phosphorescence. Quantum chemical calculations might unravel the origin of the bands, either from a higher excited state (anti-Kasha behavior)^34^ or from a conformational change^35^ in the first excited state (*vide infra*).

Overall, a comparison of the emission spectra of the *N*-alkyl substituted compounds 6 and 7, as well as 6(5*H*)-phenanthridinone 8 (see SI), indicates that the presence of an aryl group at the nitrogen is essential for the occurrence of dual emission, as both an LE and a CT band were only observed for compounds 4 and 5. Furthermore, the nature of the substituents at positions R^1^ and R^2^ influences the intensity ratio of the LE to the CT band. The CT emission increases relative to the LE band for R^1^ bearing an electron-withdrawing substituent and for R^2^ bearing an electron-donating substituent, which is consistent with observations of Demeter *et al.*^35^ This additionally supports the hypothesis of a population of the phenanthridinone core in the excited state, which might be scrutinized through theoretical analyses (*vide infra*).

The absolute fluorescence quantum yield *Φ*_em_ could only be determined for compounds 4b and 5b, with values of 1% and 2%, respectively, whereas the other compounds exhibit emission that is too weak to be recorded (Table 1). At room temperature, the dominant deactivation process of the excited *S*_1_ state in phenanthridinones is intersystem crossing (ISC) to the triplet state, which accounts for the very low fluorescence quantum yield *Φ*_em_.^35^

### Calculated electronic structure

A deeper insight into the electronic structure of *N*-arylphenanthridinones 4 and *N*-arylcrinasiadines 5 can be obtained by (TD)DFT calculations exemplarily for compounds 4b and 5b with the program package Gaussian 16.^52^ In analogy to the literature, the B3LYP^53,54^ functional and the Pople basis set 6-31G* (ref. 55) were employed for the quantum chemical analysis of the phenanthridinones.^34^ All minima were confirmed unambiguously by analytical frequency analysis (NIMAG = 0). Based on the absorption and emission properties in solution, the intrinsic polarizable continuum model (PCM) with dichloromethane as the dielectric medium was applied in the quantum chemical calculations.^56^ The calculated absorption and emission energies agree quantitatively well with the experimental data in dichloromethane for the selected examples, with only minor over- or underestimation of the energy levels (Table 2).

**Table 2:** (TD)DFT calculations on the UV/vis absorption and emission maxima of *N*-arylphenanthridinone 4b and *N*-arylcrinasiadine 5b (Gaussian 16, B3LYP/6-31G*, PCM CH_2_Cl_2_, for details see SI)

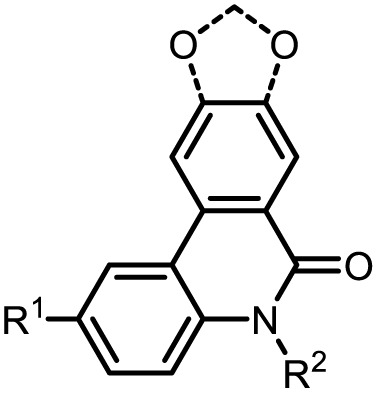						
Com-pound	R^1^	R^2^	*λ*_max,abs(exp)_ a [nm] (*ε* [m^−1^ cm^−1^])	*λ*_max,abs(calc)_ [nm] (oscillator strength *f*), most dominant contribution	*λ*_max,em(exp)_ b [nm]	*λ*_max,em(calc)_ [nm] (oscillator strength *f*), most dominant contribution
4b	F	^*t*^Bu	237 (34 700)	229 (0.0247) HOMO-3 → LUMO+1 (70%)	382, 530	405 (0.3277) HOMO → LUMO (95%), 579 (0.1270) HOMO → LUMO (99%)
				231 (0.3859) HOMO → LUMO+4 (49%)		
				235 (0.2417) HOMO-4 → LUMO (41%)		
				248 (0.0256) HOMO-3 → LUMO (88%)		
			261 (13 000)	256 (0.1342) HOMO-1 → LUMO+1 (47%)		
				269 (0.0506) HOMO-1 → LUMO (71%)		
			273 (sh, 8100)	292 (0.0311) HOMO → LUMO+1 (90%)		
			319 (5400)	316 (0.2320) HOMO → LUMO (93%)		
			331 (7200)			
			345 (sh, 6100)			
5b	F	^*t*^Bu	249 (43 400)	241 (0.1814) HOMO → LUMO+4 (53%)	372, 550	386 (0.3533) HOMO → LUMO (96%), 563 (0.1417) HOMO → LUMO (99%)
			274 (13 100)	247 (0.5248) HOMO-3 → LUMO (44%)		
			287 (sh, 10 500)	251 (0.0154) HOMO → LUMO+3 (84%)		
				268 (0.1603) HOMO → LUMO (77%)		
				286 (0.1849) HOMO-1 → LUMO (49%)		
			298 (sh, 8200)	301 (0.0114) HOMO → LUMO+1 (51%)		
			317 (sh, 9700)	319 (0.2547) HOMO → LUMO (89%)		
			329 (10 300)			
			346 (8000)			

a Recorded in CH_2_Cl_2_, *T* = 293 K, *c* = 10^−5^m.

b Recorded in CH_2_Cl_2_, *T* = 293 K, *c* = 10^−7^m.

**Fig. 5:**
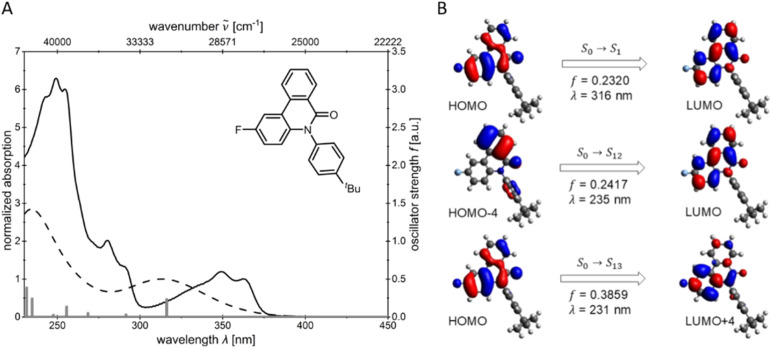
Comparison of the calculated (Gaussian 16, B3LYP/6-31G*, PCM CH_2_Cl_2_ (dashed lines)) and experimentally determined (recorded in CH_2_Cl_2_, *T* = 293 K, *c* = 10^−5^m (bold lines)) UV/vis spectra of *N*-arylphenanthridinone 4b with the calculated transitions shown as gray bars (A) and calculated molecular orbitals of 4b for the dominant electronic transitions (B) (Gaussian 16, B3LYP/6-31G*, PCM CH_2_Cl_2_, isosurface value 0.025 a.u.).

**Fig. 6:**
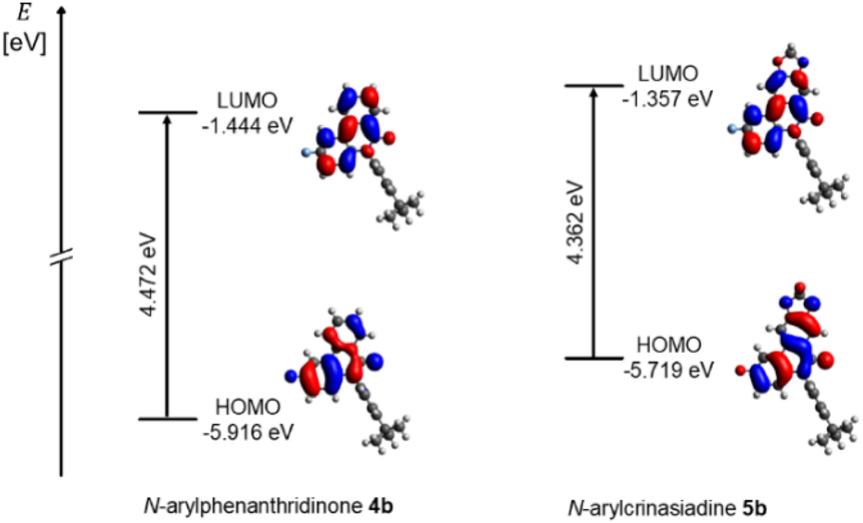
Selected Kohn–Sham FMO of *N*-arylphenanthridinone 4b and *N*-arylcrinasiadine 5b employing the PCM with dichloromethane as solvent (Gaussian 16, B3LYP/6-31G*, PCM CH_2_Cl_2_, isosurface value at 0.025 a.u.).

Since the absorption spectra of structures 4b and 5b in dichloromethane display very similar profiles, only the calculated absorption spectrum of 4b will be discussed in detail (Fig. 5). The vertical bars represent the calculated transitions and illustrate that the longest wavelength absorption band is primarily dominated by the HOMO → LUMO transition (Fig. 5A). In the calculated spectrum, the transitions combine into broad bands at both lower and higher energy. In the experimental spectrum, these appear as weakly pronounced shoulders, indicating the superposition of multiple transitions. Frontier molecular orbitals (FMO) are expectedly involved in the dominant electronic transitions (Fig. 5B). The S_0_–S_1_ and S_0_–S_13_ transitions mainly occur between delocalized π-orbitals of the same conjugated system, indicating an LE character of these states. Only the S_0_–S_12_ transition, in which the electron density is transferred from the more localized HOMO−4 to the delocalized LUMO, suggests a slight CT character. TDDFT calculations showed that S_1_ predominantly results from the HOMO → LUMO transition. In contrast, the higher excited states are composed of multiple orbital pairs and can thus be interpreted as mixtures of various electronic transitions. The energies of the FMO of compounds 4b and 5b are visualized in dichloromethane (Fig. 6). A comparison of *N*-arylphenanthridinone 4b with the structurally related crinasiadine 5b reveals that the additional oxygen atoms in crinasiadine exert electron-donating effects, leading to a slight increase of both the HOMO and LUMO energy levels.^57^ Since the HOMO is raised slightly higher in comparison to the LUMO, the energy gap Δ*E*(*E*_HOMO_ − *E*_LUMO)_ of crinasiadine 5b is slightly smaller than that of compound 4b.

However, this difference is not reflected in the experimental absorption spectra, as the position of the longest-wavelength absorption band is nearly identical for both compounds. This can be explained by the fact that the experimental band results from the superposition of several electronic transitions and therefore cannot be directly equated with the calculated HOMO → LUMO transition. For both compounds 4b and 5b, the coefficient densities of the HOMO and LUMO are predominantly localized on the phenanthridinone moiety, confirming the LE character of the lowest electronic transitions.

**Fig. 7:**
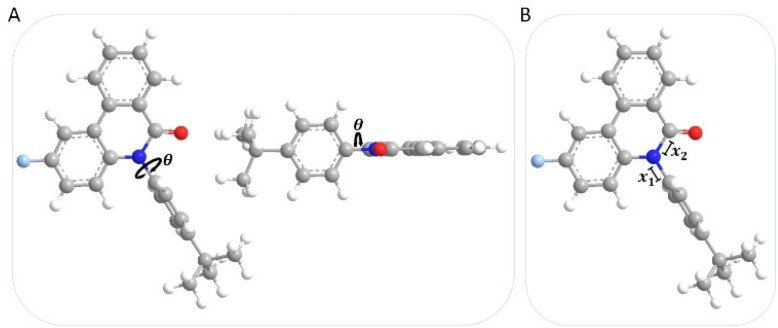
Illustration of the minimum geometry of 4b as the starting geometry for the torsion scan with the torsion angle *θ*, which describes the rotation around the *N*-aryl bond (A), with *x*_1_ as the *N*-aryl bond length and *x*_2_ as the N–CO amide bond length (B).

Demeter *et al.* investigated various dual fluorescent *N*-arylphenanthridinones, including *N*-arylphenanthridinone 4a. They recorded fluorescence decay curves in both the LE and CT regions of the spectrum and observed biexponential decays. From this, they concluded that the CT state is not directly excited from the ground state but originates instead from the initially excited LE state.^35^ They further postulated that the CT state must be significantly more planar than the LE state, as no CT fluorescence is observed in twisted *N*-arylphenanthridinones. In these cases, complete planarity is sterically hindered by *ortho*-methyl substituents on the aryl group.^35^ Based on these results, a potential energy scan was carried out in both the ground (S_0_ and S_0_*) and excited states (S_1_) to investigate the geometry dependence of the electronic states and possible relaxation pathways between the LE and CT states using quantum chemical methods. The CN-bond rotation was modeled by a torsional scan by 360° rotation of the *N*-aryl substituent with the torsion angle *θ* (Fig. 7). The initial geometry is defined as a rotational angle *θ* of 0°. The energy curves of compounds 4b and 5b are very analogous in the vibrationally relaxed (S_0_) and vibrationally excited (S_0_*) ground states (see SI), with the perpendicular geometry representing the global minimum in each case (NIMAG = 0), *i.e.* thermodynamically preferred conformations. In contrast, the coplanar orientation of the phenanthridinone unit and the aryl substituent occurs in a transition state with an imaginary frequency (NIMAG = 1). This rotational barrier Δ*E* amounts to 0.72 eV (17.0 kcal mol^−1^) for compound 4b and 0.74 eV (17.1 kcal mol^−1^) for compound 5b, which can be thermally surmounted at room temperature, yet significantly slowed down kinetically.^58^ The rate constants *k* (*k*(4b) = 4.14 s^−1^ and *k*(5b) = 1.90 s^−1^) of the rotation around the C–N-bond can be estimated using the Eyring equation^59^ to proceed quite slowly.

**Fig. 8:**
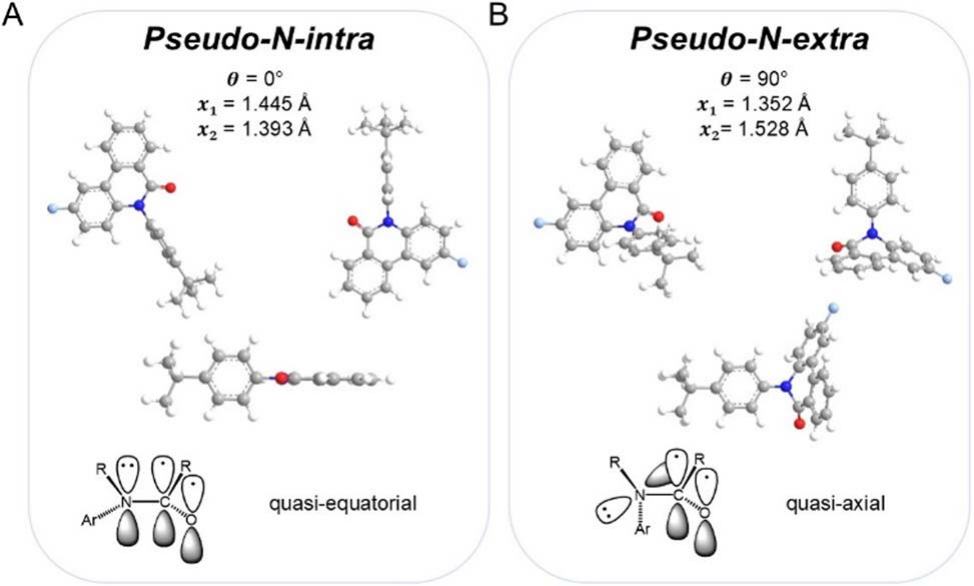
Comparison of the excited state geometries of *pseudo-N-intra* (A) and *pseudo-N-extra* (B) from different perspectives for compound 4b (top, Gaussian 16, B3LYP/6-31G*, PCM CH_2_Cl_2_), along with a schematic representation of the *N*-aryl group (Ar) in the quasi-equatorial *intra*-conformation and quasi-axial *extra*-conformation (bottom, R = anellated benzene rings).

The energy curves of compounds 4b and 5b in the excited state (S_1_) also exhibit very similar profiles (see SI). In contrast to the ground states, however, the coplanar geometry (NIMAG = 0) now represents the global minimum, while a conformer slightly twisted from a perpendicular geometry constitutes a second local minimum (NIMAG = 0). The coplanar geometry reveals a shorter *N*-aryl bond length *x*_1_, which therefore indicates partial double bond character and suggests a more efficient π-conjugation compared to the perpendicular geometry. Furthermore, the N–CO amide bond length *x*_2_ is elongated in the coplanar geometry. Both geometries resemble the *N-intra* and *N-extra* conformations of phenothiazine.^60^ In the *N-intra* conformation, the nitrogen lone pair of phenothiazine overlaps with the π-system of the anellated benzene rings due to the quasi-equatorial orientation of the substituent. In the *N-extra* conformation, the substituent adopts a quasi-axial position. Unlike in phenothiazine, however, the nitrogen atom of the amide group is not sp^3^-hybridized and pyramidal, but sp^2^-hybridized and arranged in a trigonal planar geometry.^35,61,62^ Therefore, it is more appropriate to refer to a perpendicular *pseudo-N-intra* conformation and a coplanar *pseudo-N-extra* conformation. These two conformations are illustrated exemplarily for compound 4b (Fig. 8).

The energy barrier Δ*E*(S_1_(LE)-S_1_(CT)) between the two conformers S_1_(LE) and S_1_(CT) in the excited state is 0.33 eV (7.61 kcal mol^−1^) for compound 4b and 0.30 eV (7.07 kcal mol^−1^) for compound 5b, and is easily overcome at room temperature,^58^ which may explain the occurrence of dual emission at this temperature. The calculated rate constants *k* in the excited state (*k*(4b) = 1.63 × 10^7^ s^−1^ and *k*(5b) = 5.24 × 10^7^ s^−1^) support this hypothesis, as the conformational interconversion proceeds with high efficiency. Assuming a Boltzmann distribution, the theoretical Gibbs free energy Δ*G* of both conformers (Δ*G*(4b) = 0.30 eV (6.89 kcal mol^−1^) and Δ*G*(5b) = 0.23 eV (5.22 kcal mol^−1^)) yields the equilibrium constants *K* for dyes 4b (7.29 × 10^−6^) and 5b (1.27 × 10^−4^) corresponding to a population ratio of 99.987 to 0.013 in favor of the coplanar *pseudo-N-extra* conformation S_1_(CT). The dual emission is therefore a consequence of the kinetic competition between emission from the *pseudo-N-intra* conformation S_1_(LE) and conformational interconversion to conformer S_1_(CT), leading to partial emission from both conformers.^35^

For a quantitative assessment of the dual emission behavior, the ratio of the integral emission intensities of both bands on a Jacobian energy-corrected scale can be considered (see SI, chpt. 9.5) and compared with the theoretically calculated oscillator strengths.^63^ For dye 4b, the integrated emission spectra give a ratio *A*_LE_/*A*_CT_ of 2.14, while the quantum-chemically calculated oscillator strengths give a ratio *f*_LE_/*f*_CT_ of 2.58. Thus, *f*_LE_/*f*_CT_ moderately overestimates *A*_LE_/*A*_CT_ but remains consistent in trend with the experimental result, thereby supporting the assignment of the two emission channels. For dye 5b, however, *f*_LE_/*f*_CT_ (= 2.49) does not adequately capture *A*_LE_/*A*_CT_ (= 4.25). The deviations can be attributed to differences in baseline choice and contributions from nonradiative processes.^64,65^

**Fig. 9:**
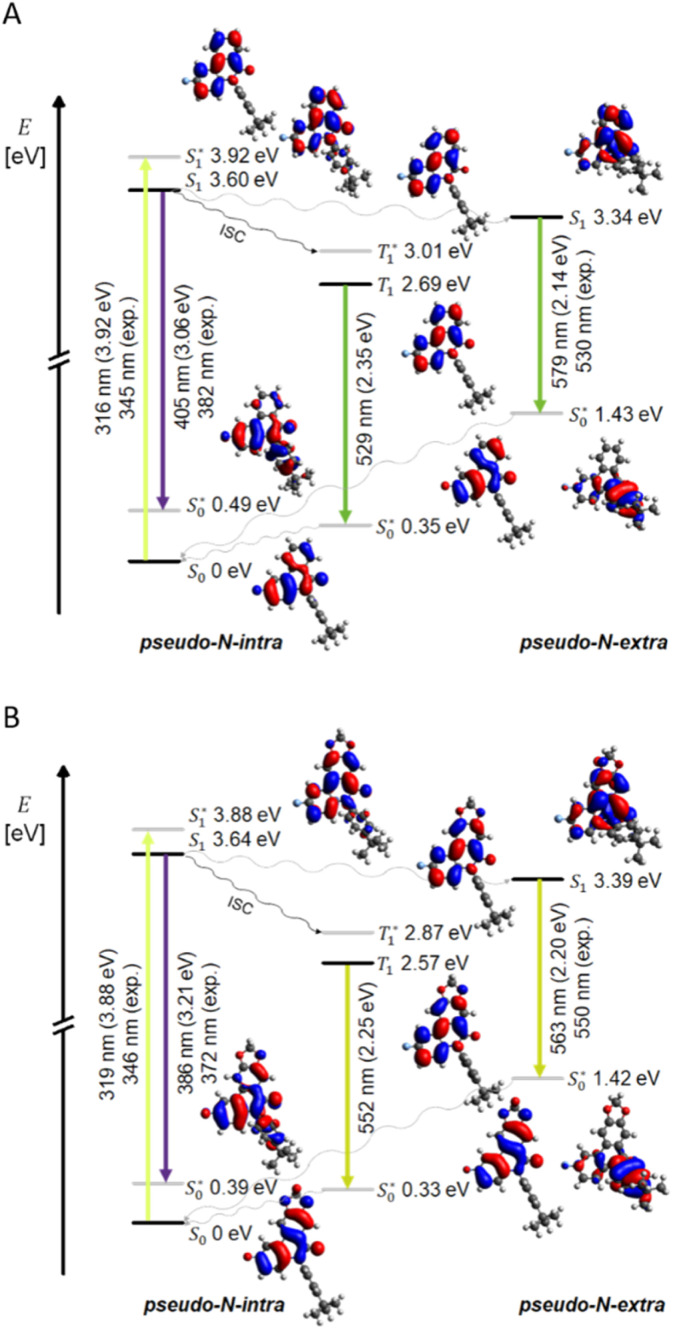
Schematic Jabłoński diagrams and Kohn–Sham-FMOs of the calculated S_1_ and T_1_ states, including the S_0_–S_1_* transition (absorption at the longest wavelength), the S_1_–S_0_* transition of the *pseudo-N-intra* (LE fluorescence) and *pseudo-N-extra* (CT fluorescence), the S_1_–T_1_* transition (ISC), as well as the T_1_–S_0_* transition of *pseudo-N-intra* (phosphorescence) for *N*-arylphenanthridinone 4b (A) and *N*-arylcrinasiadine 5b (B) (Gaussian 16, B3LYP/6-31G*, PCM CH_2_Cl_2_, isosurface value 0.025 a.u.).

Jabłoński diagrams illustrate the absorption and emission of compounds 4b (Fig. 9 (A)) and 5b (Fig. 9(B)). In the *pseudo-N-intra* conformation, the vibrationally excited S_1_^*^ state lies only slightly above the vibrationally relaxed S_1_ LE-state in energy (Δ*E*(4b) = 0.32 eV and Δ*E*(5b) = 0.24 eV), which represent small Stokes shifts (Δ*ν̃*_s(exp)_ (4b) = 2800 cm^−1^*vs.* Δ*ν̃*_s(calc)_ (4b) = 7000 cm^−1^ and Δ*ν̃*_s(exp_ (5b) = 2000 cm^−1^*vs.* Δ*ν̃*_s(calc)_ (5b) = 5400 cm^−1^). As a result, only minimal structural changes occur and the aryl substituent only slightly twists. This twisting causes the aryl unit to bear less electron density compared to the Franck–Condon geometry. Nevertheless, the electron density remains predominantly localized on the phenanthridinone moiety. Even after emission from the S_1_ state, the coefficient density remains localized on the phenanthridinone core and the resulting transition can be assigned to the short wavelength LE band in the experimental emission spectrum. However, due to coefficient density on the aryl substituent in the vibrationally excited S_0_* state, the transition cannot be regarded as a pure LE transition.

The conformational change to the *pseudo-N-extra* conformer lowers of the excited-state energy level. Planarization enables more efficient delocalization of the electron density, which is also reflected in the shorter *N*-aryl bond length *x*_1_ of the conformer. In the *pseudo-N-extra* conformation, the vibrationally excited S_1_^*^ state lies significantly above the vibrationally relaxed S_1_ CT-state in energy (Δ*E* (4b) = 0.58 eV and Δ*E* (5b) = 0.49 eV). As a result of significant structural changes, which cause large Stokes shifts (Δ*ν̃*_s(exp)_ (4b) = 10 100 cm^−1^*vs.* Δ*ν̃*_s(calc)_ (4b) = 12 800 cm^−1^ and Δ*ν̃*_s(exp)_ (5b) = 10 700 cm^−1^*vs.* Δ*ν̃*_s(calc)_ (5b) = 13 200 cm^−1^) and a smaller energy gap Δ*E*, the redshifted emission is observed. Therefore, S_1_–S_0_* CT-transition from the phenanthridinone to the aryl unit occurs. The coefficient distribution of the T_1_ state of the *pseudo-N-intra* conformation is for both compounds 4b and 5b very similar to the S_1_ state but lies energetically well below the S_1_ state of the *pseudo-N-extra* conformation (see SI).

Nevertheless, according to El-Sayed's rule ISC of similar transitions can be considered to occur less probable.^66^ The quantum chemically calculated phosphorescence of compounds 4b and 5b corresponds well with the experimentally observed long-wave emission bands. But the nearly identical experimental emission spectra in degassed and non-degassed solutions (see SI) indicate that no significant oxygen-induced triplet quenching occurs. Furthermore, the relatively large energy gap Δ*E*_ST_ between T_1_ and S_1_ in the *pseudo-N-intra* renders an ISC unlikely. Alternatively, a conformational change to the *pseudo-N-extra* conformation might occur prior to ISC, from which a transition to the T_1_ state of this geometry could take place, or an ISC to higher triplet states might occur, which could account for the low fluorescence quantum yield *Φ*_em_.^35^

The localization of the LUMO on the phenanthridinone moiety in both conformers further explains the experimentally observed substituent effects on the LE to CT band ratio (Fig. 6). The weak fluorine acceptor at position R^1^ stabilizes the electron density in the excited state and thus favors both bands, while electron donors would probably cause destabilization. Donors at R^2^ facilitate mesomeric delocalization in the LUMO especially in a planar geometry thus enhancing CT emission. In contrast, acceptors at R^2^ withdraw electron density and destabilize the planar conformation and shift the emission ratio in favor of the LE band, which is consistent with experimental observations.

## Conclusions

The consecutive sequentially palladium catalyzed three-component synthesis provides a concise approach to functionalized phenanthridinones and crinasiadines in a one-pot fashion. While *N*-aryl derivatives are obtained by this sequence, *N*-alkyl derivatives form by Suzuki arylation–lactamization followed by *N*-alkylation, also in a one-pot fashion. Particularly noteworthy is the concise synthesis of two natural products, *N*-methylcrinasiadine and *N*-phenethylcrinasiadine, whose cytotoxic effects against leukemia, lung, and colon cancer cells underscore the biological relevance of the synthetic derivatives.

Photophysical investigations reveal dual fluorescence of these compounds which can be attributed to involvement of LE (locally excited) and CT (charge transfer) states. The substitution pattern clearly affects the emission behavior, allowing fine-tuning of electronic properties to achieve desired material characteristics. Quantum chemical calculations provide initial rationalization of the origin of the LE and CT bands and suggest that the LE band emerges from the *pseudo-N-intra* conformation, while the redshifted CT band arises from the *pseudo-N-extra* conformation.

The results pave the way for the development of novel, accurately tunable phenanthridinone and crinasiadine emitters. Further studies on the syntheses of alkaloid analogues with increased emission quantum yield are currently underway.

## Author contributions

The work consists of parts of the planned PhD thesis of R. K., which is supervised by T. J. J. M. Methodologic and synthetic studies, analytical characterization of the synthetic samples, photophysics (steady-state absorption and emission spectroscopy), and quantum chemical calculation by R. K., who compiled the obtained data. The conceptualization was outlined by T. J. J. M. The writing of the first draft was completed by R. K., and the review and editing was completed by R. K. and T. J. J. M. Project administration and funding acquisition by T. J. J. M. Both authors have read and agreed to the published version of the manuscript.

## Conflicts of interest

There are no conflicts to declare.

## Supplementary Material

RA-015-D5RA06934C-s001

## Data Availability

The data supporting this article have been included as part of the supplementary information (SI). Supplementary information: synthetic details, ^1^H and ^13^C NMR spectra, HPLC traces, absorption and emission spectra as well as details on the (TD)DFT calculations. See DOI: https://doi.org/10.1039/d5ra06934c.
