# Supplementary material for: Dual fluorescent phenanthridinones and crinasiadine derivatives by consecutive palladium-catalyzed three-component syntheses
Source: RSC Adv. 2025 Dec 10;15(57):49364–73. doi: 10.1039/d5ra06934c (PMC12695140; doi:10.1039/d5ra06934c)
Supplement: RA-015-D5RA06934C-s001 [file RA-015-D5RA06934C-s001.pdf]

Supplementary Information (SI) for RSC Advances.
This journal is © The Royal Society of Chemistry 2025

Supporting Information

Dual fluorescent phenanthridinones and crinasiadine derivatives by consecutive palladium-catalyzed three-component syntheses

Regina Kohlbecher, and Thomas J. J. Müller*

Heinrich Heine University Duesseldorf, Faculty of Mathematics and Natural Sciences, Institute of Organic Chemistry and Macromolecular Chemistry, Universitätstrasse 1, Düsseldorf D-40225, Germany

E-mail: ThomasJJ.Mueller@hhu.de

Table of contents

1 General considerations ... 3
2 Synthesis and spectroscopic data of 6-bromobenzo[*d*][1,3]dioxolo-5-carbaldehyde[1] 5
3 Synthesis and spectroscopic data of methyl 6-bromobenzo[*d*][1,3]-dioxol-5-carboxylate[3] ... 5
4 Synthesis and spectroscopic data of Piperonal boronic acid pinacol ester **3b**[4] ... 6
5 Synthesis of *N*-arylphenanthridinones 4 and *N*-arylcrinasiadines **5** ... 7
5.1 Optimization of *N*-arylphenanthridinones 4 and *N*-arylcrinasiadines **5** ... 7
5.2 General procedure (**GP1**) for the synthesis of *N*-arylphenanthridinones **4** and *N*-arylcrinasiadines **5** ... 15
5.3 Spectroscopic data of *N*-arylphenanthridinones 4 and *N*-arylcrinasiadines **5** ... 17
6 Synthesis of *N*-alkylphenanthridinones 6 and *N*-alkylcrinasiadines **7** ... 21
6.1 Optimization of *N*-alkylphenanthridinones 6 and *N*-alkylcrinasiadines **7** ... 21
6.2 General procedure (**GP2**) for the synthesis of *N*-alkylphenanthridinones **6** and *N*-alkylcrinasiadines **7** ... 22
6.3 Spectroscopic data of *N*-alkylphenanthridinones **6** and *N*-alkylcrinasiadines **7** ... 24
7 Synthesis of 6(*5H*)-phenanthridinone **8** ... 27
8 ^{1}H and ^{13}C spectra of *N*-arylphenanthridinones 4 and *N*-arylcrinasiadines **5** ... 28
9 ^{1}H and ^{13}C spectra of *N*-alkylphenanthridinones 6 and *N*-alkylcrinasiadines **7** ... 35
10 High-Performance Liquid Chromatography (HPLC) Data of *N*-arylphenanthridinones 4 and *N*-arylcrinasiadines **5** ... 40
10.1 HPLC Data of 5-(4-(*tert*-Butyl)phenyl)-2-fluorophenanthridin-6(5*H*)-one (**4b**) ... 40
10.2 HPLC Data of 2-Fluoro-5-phenylphenanthridin-6(5*H*)-one (**4c**) ... 40
10.3 HPLC Data of 5-(4-(*tert*-Butyl)phenyl)-2-fluoro-[1,3]dioxolo[4,5-j]phenanthridin-6(5*H*)-one (**5b**) ... 41
10.4 HPLC Data of 2-Fluoro-5-Phenyl-[1,3]dioxolo[4,5-j]phenanthridin-6(5*H*)-one (**5c**) ... 41

11 Absorption and emission spectra ... 42
11.1 Absorption and emission spectra of 6(5*H*)-phenanthridinone (**8**) ... 42
11.2 Absorption and emission spectra of *N*-arylphenanthridinones **4** and *N*-arylcrinasiadines **5** ... 42
11.3 Absorption and emission spectra of *N*-alkylphenanthridinones **6** and *N*-alkylcrinasiadines **7** ... 46
11.4 Degassed and non-degassed emission spectra of *N*-arylphenanthridinone **4b** and *N*-arylcrinasiadine **5b** ... 48
11.5 Jacobian[10] energy-corrected emission spectra and corresponding integrated emission intensities of *N*-arylphenanthridinone **4b** and *N*-arylcrinasiadine **5b** ... 50
12 Data of quantum chemical calculations ... 52
12.1 Comparison of the potential scans of 5-(4-(*tert*-butyl)phenyl)-2-fluorophenanthridin-6(5*H*)-one (**4b**) and 5-(4-(*tert*-Butyl)phenyl)-2-fluoro-[1,3]dioxolo[4,5-j]phenanthridin-6(5*H*)-one (**5b**) ... 52
12.2 Quantum chemical calculation data of 5-(4-(*tert*-butyl)phenyl)-2-fluorophenanthridin-6(5*H*)-one (**4b**) ... 53
12.2.1 Computed xyz-coordinates of compound **4b** (B3LYP/6-31G* PCM CH_2Cl_2) ... 53
12.2.2 Computed excitations energies of compound **4b** (B3LYP/6-31G* PCM CH_2Cl_2) ... 54
12.2.3 Computed LE emission energies of compound **4b** (B3LYP/6-31G* PCM CH_2Cl_2) ... 56
12.2.4 Potential scan of compound **4b** ground state (B3LYP/6-31G* PCM CH_2Cl_2) ... 58
12.2.5 Potential scan of compound **4b** excited state (B3LYP/6-31G* PCM CH_2Cl_2) ... 100
12.2.6 Computed CT emission energies of compound **4b** (B3LYP/6-31G* PCM CH_2Cl_2) 146
12.2.7 Computed excitations energies of the triplet state of compound **4b** (B3LYP/6-31G* PCM CH_2Cl_2) ... 146
12.2.8 Computed emission energies of the triplet state of compound **4b** (B3LYP/6-31G* PCM CH_2Cl_2) ... 149
12.3 Quantum chemical calculation data of 5-(4-(*tert*-Butyl)phenyl)-2-fluoro-[1,3]dioxolo[4,5-j]phenanthridin-6(5*H*)-one (**5b**) ... 153
12.3.1 Computed xyz-coordinates of compound **5b** (B3LYP/6-31G* PCM CH_2Cl_2) ... 153
12.3.2 Computed excitations energies of compound **5b** (B3LYP/6-31G* PCM CH_2Cl_2) ... 154
12.3.3 Computed emission energies of compound **5b** (B3LYP/6-31G* PCM CH_2Cl_2) ... 156
12.3.4 Potential scan of compound **5b** ground state (B3LYP/6-31G* PCM CH_2Cl_2) ... 157
12.3.5 Potential scan of compound **5b** excited state (B3LYP/6-31G* PCM CH_2Cl_2) ... 202
12.3.6 Computed CT emission energies of compound **5b** (B3LYP/6-31G* PCM CH_2Cl_2) 249
12.3.7 Computed excitations energies of the triplet state of compound **5b** (B3LYP/6-31G* PCM CH_2Cl_2) ... 250
12.3.8 Computed emission energies of the triplet state of compound **5b** (B3LYP/6-31G* PCM CH_2Cl_2) ... 252
13 References ... 256

1 General considerations

All reactions were carried out in oven dried Schlenk glassware using septa and syringes under nitrogen atmosphere. The reagents and catalyst were purchased reagent-grade and used without purification. Dry solvents were dried by a solvent purification system.

The reaction progress was monitored qualitatively using TLC Silica gel 60 F234 aluminum sheets obtained from Macherey Nagel GmbH & Co. KG. The spots were detected with UV light at 254 and 366 nm. The purification of the products was performed on silica gel 60 M (0.04-0.063 mm) from Macherey-Nagel GmbH & Co. KG using flash technique under pressure of 2 bar. The crude mixtures were absorbed on Celite® 545 form Carl Roth GmbH & Co. KG before chromatographic purification. Ethyl acetate and mixtures of *n*-hexane/ethyl acetate were used as eluent.

^{1}H, ^{19}F, ^{13}C and 135-DEPT ^{13}C NMR spectra were recorded on Bruker AVIII-300 and AVIII-600. DMSO-d_6 and chloroform-d_1 were used as deuterated solvents. The resonances of the solvents were locked as internal standard (DMSO-d_6: ^{1}H δ 2.50 (water in DMSO-d_6 ^{1}H δ 3.33)*, ^{13}C δ 39.52; $CDCl_3$: ^{1}H δ 7.26, ^{13}C δ 77.00). For recording ^{19}F NMR spectra, trichlorofluoromethane (^{19}F δ 0.0) was added to the solvent chloroform-d_1, which served as an internal standard. A relaxation delay time d_1 of 10 s was selected for the spectrum recording. The multiplicities of the signals were abbreviated as follows: s: singlet; d: doublet; t: triplet; q: quartet; dd: doublet of doublet; dt: doublet of triplet; tt: triplet of triplets; ddd: doublet of doublet of doublets; m: multiplet. The type of carbon nucleus was determined based on 135-DEPT ^{13}C NMR spectra. For the description of the ^{13}C NMR spectra primary carbon nuclei are abbreviated with CH_3, secondary carbon nuclei with CH_2, tertiary carbon nuclei with CH and quaternary carbon nuclei with C_{quat}. The program Mestrenova 11.0 was used to evaluate the NMR spectra.

EI mass spectra were measured on Finnigan MAT TSQ 7000.

IR spectra were obtained on Shimadzu IR Affinity-1 which works with the attenuated total reflection (ATR) method. The intensity of signals is abbreviated as follows: s (strong), m (medium), w (weak).

The melting points (uncorrected) were measured on Büchi Melting Point B-540.

* Due to the water content in DMSO-d_6, the water peaks in the ^{1}H NMR spectra are also labeled with “DMSO-d_6”.

Absorption spectra were recorded in dichloromethane and acetonitrile (high performance liquid chromatography (HPLC) grade) at 293 K on a Perkin–Elmer UV/Vis/NIR Lambda 19 spectrometer. For the determination of the molar extinction coefficients ε absorption measurements at five different concentrations were carried out. Emission spectra were recorded in ethyl acetate HPLC grade at 293 K on a Hitachi F-7000 spectrometer.
Quantum chemical calculations were carried out utilizing the HPC-Cluster Hilbert of the Center for Information and Media Technology at the Heinrich Heine University Düsseldorf.

2 Synthesis and spectroscopic data of 6-bromobenzo[*d*][1,3]dioxolo-5-carbaldehyde[1]

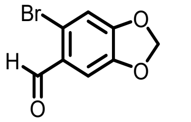

In a 100 mL three-neck flask with magnetic stir bar, piperonal (1.95 g, 13.0 mmol, 1.00 equiv) was placed and dissolved in dry methanol (10 mL) at 0 °C. Subsequently, elemental bromine (3.20 g, 20.0 mmol, 1.50 equiv) was dissolved in dry methanol (10 mL) and slowly added dropwise. After stirring at room temperature for 16 h, the reaction mixture was treated with a saturated sodium sulfite solution, dichloromethane, and deionized water, and the organic phase was separated. The aqueous phase was extracted three times with dichloromethane. The combined organic phases were then dried with anhydrous magnesium sulfate, and the solvent was removed under reduced pressure. After subsequent recrystallization from *n*-hexane, the product was obtained as colorless crystals in a yield of 83% (2.49 g, 10.9 mmol).

Mp 130 °C (Lit.: 132 – 134 °C[2]). R_f 0.47 (*n*-hexane/ethyl acetate 5:1). ^{1}H NMR (600 MHz, $CDCl_3$) δ 6.08 (s, 2 H), 7.06 (s, 1 H), 7.36 (s, 1 H), 10.18 (s, 1 H). ^{13}C NMR (150 MHz, $CDCl_3$) δ 102.9 (CH_2), 108.3 (CH), 113.4 (CH), 121.8 (C_{quat}), 128.2 (C_{quat}), 148.3 (C_{quat}), 153.5 (C_{quat}), 190.5 (C_{quat}). EI MS (70 eV, m/z (%)) 230 ($[C_8H_5{}^{81}BrO_3]^+$, 86), 229 ($C_8H_5{}^{79}BrO_3]^+$, 100), 228 ($[C_8H_3{}^{81}BrO_3]^+$, 21), 227 ($[C_8H_3{}^{79}BrO_3]^+$, 15), 201 ($[C_7H_4{}^{81}BrO_2]^+$, 27), 199 ($[C_7H_4{}^{79}BrO_2]^+$, 30), 145 ($[C_4H^{81}BrO]^+$, 7) 143 ($[C_4H^{79}BrO]^+$, 11), 63 ($[C_5H_3]^+$, 18), 62 (($[C_5H_2]^+$, 23).

3 Synthesis and spectroscopic data of methyl 6-bromobenzo[*d*][1,3]-dioxol-5-carboxylate[3]

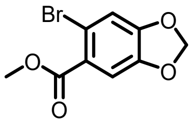

In a 250 mL two-neck flask, potassium hydroxide (813 mg, 14.5 mmol, 8.00 equiv) was placed and dissolved in methanol (50 mL) at 0 °C. Subsequently, 6-bromobenzo[d][1,3]dioxole-5-carbaldehyde (415 mg, 1.81 mmol, 1.00 equiv) was dissolved in methanol (80 mL) and added dropwise. This was followed by the addition of elemental iodine (1.84 g, 7.24 mmol, 4.00 equiv). The reaction mixture was stirred for 2 h at 0 °C and then for 14 h at 20 °C. The solution was then decolorized by the addition of sodium thiosulfate. Most of the solvent was subsequently removed under reduced pressure using a rotary evaporator. The residue was treated with a saturated sodium thiosulfate solution, dichloromethane, and deionized water, and the organic phase was separated. The aqueous phase was extracted three times with dichloromethane. The combined organic phases were then dried with anhydrous magnesium sulfate, and the solvent was removed under reduced pressure. After purification by column chromatography (*n*-hexane/ethyl acetate 20:1) and subsequent recrystallization from *n*-hexane, the product was obtained as colorless crystals in a yield of 94% (440 mg, 1.70 mmol).

Mp 88 °C (Lit.: 83 – 85 °C[3]). R_f 0.46 (*n*-hexane/ethyl acetate 15:1). ^1H NMR (600 MHz, $CDCl_3$) δ 3.89 (s, 3 H), 6.04 (s, 1H), 7.09 (s, 1 H), 7.32 (s, 1 H). ^{13}C NMR (150 MHz, $CDCl_3$) δ 52.5 (CH_3), 102.6 (CH_2), 111.2 (CH), 114.5 (CH), 115.1 (C_{quat}), 124.7 (C_{quat}), 147.3 (C_{quat}), 151.1 (C_{quat}), 165.9 (C_{quat}). EI MS (70 eV, m/z (%)) 260 ($[C_9H_7{}^{81}BrO_4]^+$, 69), 258 ($[C_9H_7{}^{79}BrO_4]^+$, 74), 229 ($[C_8H_4{}^{81}BrO_3]^+$, 100), 227 ($[C_8H_4{}^{79}BrO_3]^+$, 100), 201 ($[C_7H_4{}^{81}BrO_2]^+$, 25), 199 ($[C_7H_4{}^{79}BrO_2]^+$, 27), 143 (14), 62 (18).

4 Synthesis and spectroscopic data of Piperonal boronic acid pinacol ester 3b[4]

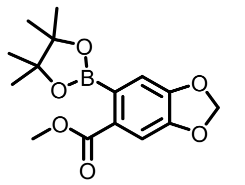

In a sealed Schlenk tube, methyl 6-bromobenzo[d][1,3]dioxole-5-carboxylate (130 mg, 0.500 mmol, 1.00 equiv), palladium(II) acetate (6 mg, 25.0 μmol, 5.00 mol%), 2-dicyclohexylphosphino-2′,4′,6′-triisopropylbiphenyl (48 mg, 100 μmol, 20.0 mol%), and triethylamine (202 mg, 2.00 mmol, 4.00 equiv) were placed and dissolved in dry 1,4-dioxane (1.3 mL). After the slow dropwise addition of pinacolborane (192 mg, 1.50 mmol, 3.00 equiv), the mixture was stirred for 15 h at 80 °C. The reaction mixture was then treated with deionized water and dichloromethane, and the organic phase was separated. The aqueous phase was extracted three times with dichloromethane. The combined organic phases were then dried with anhydrous magnesium sulfate, and the solvent was removed under reduced pressure. After purification by column chromatography (*n*-hexane/ethyl acetate 5:1), the product **3b** was obtained as a pale-yellow resin in a yield of 49% (75 mg, 0.245 mmol).

R_f 0.41 (*n*-hexane/ethyl acetate 5:1). ^1H NMR (600 MHz, $CDCl_3$) δ 1.40 (s, 12 H), 3.87 (s, 3 H), 6.01 (s, 2 H), 6.87 (s, 1 H), 7.38 (s, 1 H). ^{13}C NMR (150 MHz, $CDCl_3$) δ 25.0 (CH_3), 52.4 (CH_3), 84.1 (C_{quat}), 101.8 (CH_2), 109.3 (CH), 111.5 (CH), 127.7 (C_{quat}), 148.4 (C_{quat}), 151.2 (C_{quat}), 167.9 (C_{quat}). EI MS (70 eV, m/z (%)) 306 ($[C_{15}H_{19}{}^{11}BO_6]^+$, 11), 305 ($[C_{15}H_{19}{}^{10}BO_6]^+$, 3), 248 ($[C_{13}H_{17}{}^{11}BO_4]^+$, 100), 247 ($[C_{13}H_{17}{}^{12}BO_4]^+$, 28), 233 ($[C_{12}H_{14}{}^{11}BO_4]^+$, 53), 232 ($[C_{12}H_{14}{}^{10}BO_4]^+$, 13), 193 ($[C_9H_{10}{}^{11}BO_4]^+$, 32), 192 ($[C_9H_{10}{}^{10}BO_4]^+$, 22), 191 ($[C_8H_4{}^{11}BO_5]^+$, 88), 190 ($[C_8H_4{}^{10}BO_5]^+$, 28), 175 ($[C_8H_4{}^{11}BO_4]^+$, 66), 174 ($[C_8H_4{}^{10}BO_4]^+$, 16), 148 ($[C_7H_5{}^{11}BO_3]^+$, 11), 147 ($[C_7H_5{}^{10}BO_3]^+$, 24), 91 ($[C_{15}H_{10}NO]^+$, 18).

5 Synthesis of *N*-arylphenanthridinones 4 and *N*-arylcrinasiadines 5

5.1 Optimization of *N*-arylphenanthridinones 4 and *N*-arylcrinasiadines 5

Table S 1. Chemical shifts of the relevant compounds in the ^{19}F NMR spectrum

compound	chemical shift δ
trichlorofluoromethane[a]	0.0
2-bromo-4-fluoroaniline (**1b**)	-126.77
4-fluoro-2-iodoaniline (**1d**)	-126.81
phenanthridinone **A**	-120.69
diarylamine **B**	-121.59
N-arylphenanthridinone **4b**	-121.01
N-arylphenanthridinone **4c**	-120.85
dihydrophenazine **C**	-122.50
dihydrophenazine **D**	-123.71

[a] Reference

Table S2. Experimental details on the optimization of the 4-fluoro-2-haloaniline **1b** and **1d** used for the preparation of phenanthridinone **A**.

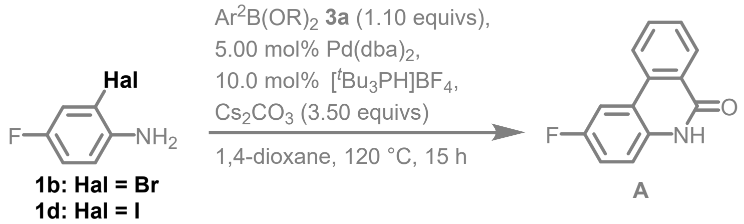

entry	aniline	yield (%)
1	2-bromo-4-fluoroaniline (**1b**)	30
2	4-fluoro-2-iodaniline (**1d**)	41

Table S3. Experimental details on the optimization of the ligand used for the preparation of phenanthridinone **A**.

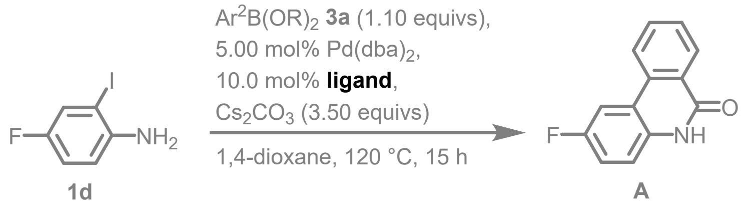

entry	ligand	yield of compound A (%)
1	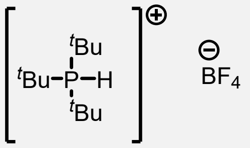 [$^tBu_3PH$]BF_4	41
2	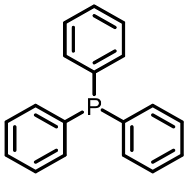 Triphenylphosphan	-

3	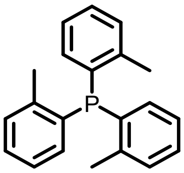 Tri(*o*-tolyl)phosphan	-
4	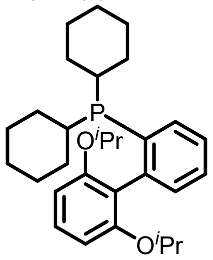 RuPhos	10
5	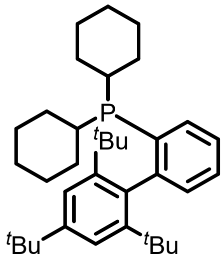 XPhos	16
6	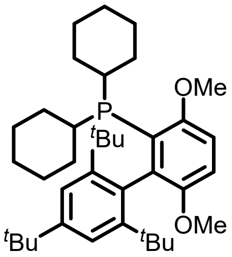 BrettPhos	10
7	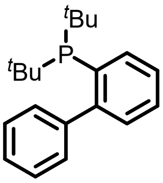 JohnPhos	7
8	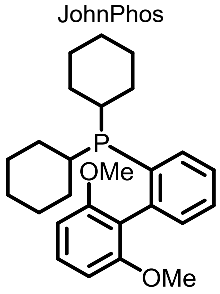 SPhos	16

9	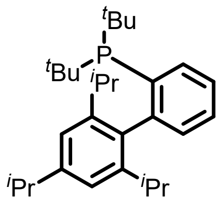 ^{t}BuXPhos	-
10	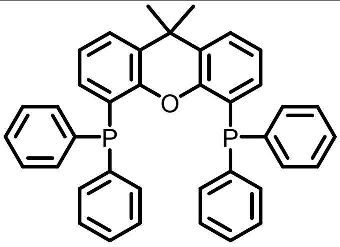 Xantphos	6
11	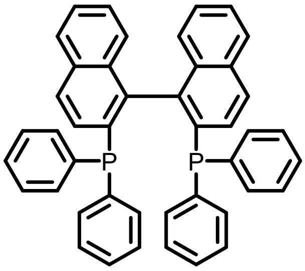 BINAP	-
12	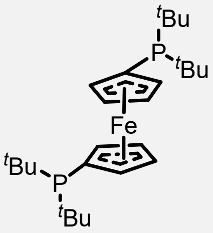 DTBPF	43
13	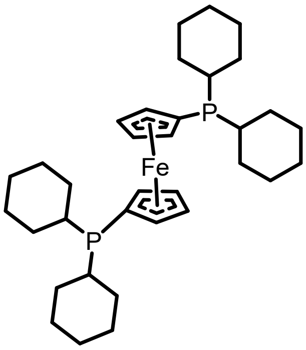 DCPF	12

14	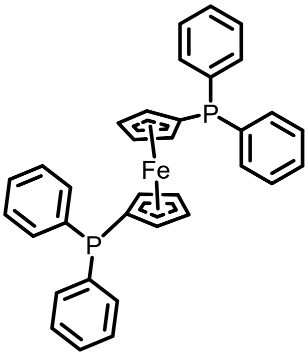 DPPF	10

Table S4. Experimental details on the optimization of the base used for the synthesis of phenanthridinone **A**.

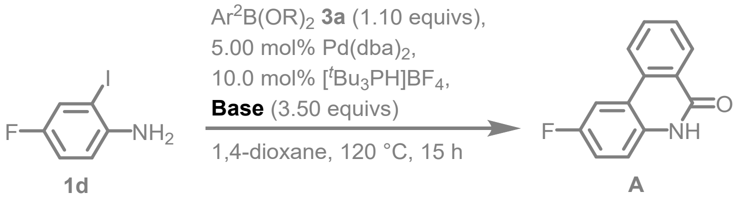

entry	base	yield of compound A (%)
1	$NaHCO_3$	-
2	Na_2CO_3	6
3	NaO*t*Bu	1
4	K_2CO_3	36
5	CsF	40
6	Cs_2CO_3	41

Table S5. Experimental details on the optimization of the amount of cesium carbonate base used for the synthesis of phenanthridinone **A**.

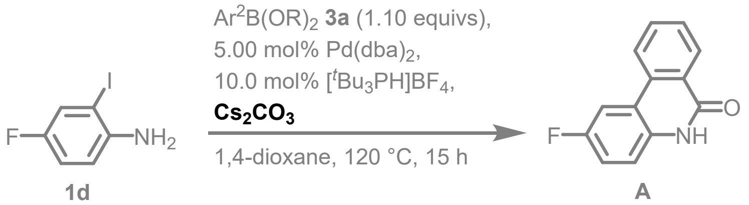

entry	equivs of Cs_2CO_3	yield of compound A (%)
1	2.50	10
2	3.50	41
3	4.50	-

Table S6. Experimental details on the optimization of the solvent used for the preparation of phenanthridinone **A**.

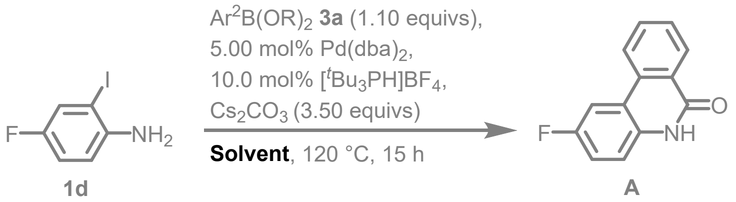

entry	solvent	yield of compound A (%)
1	1,4-dioxane	41
2	DMSO	3
3	DMF	9

Table S7. Experimental details on the optimization of the solvent mixture used for the preparation of phenanthridinone **A**.

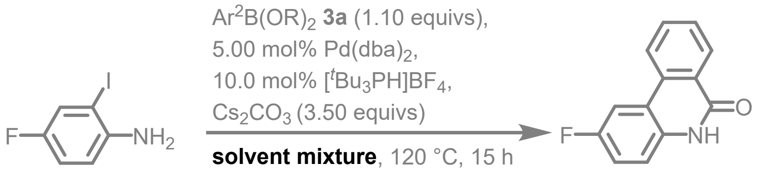

1d **A**

entry	Solvent mixture (ratio)	yield of compound A (%)
1	1,4-dioxane/DMF (1:1)	30
2	1,4-dioxane/DMF (2:1)	45
3	1,4-dioxane/DMF (3:1)	31

Table S8. Experimental details on the optimization of the solvent mixture for the preparation of phenanthridinone **A**.

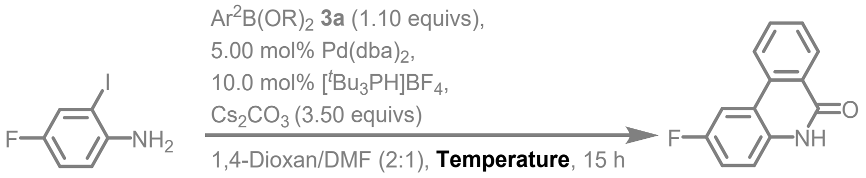

1d **A**

entry	temperature (°C)	yield of compound A (%)
1	80	14
2	100	3
3	120	45
4	140	15

Table S9. Experimental details on the optimization of the ligand used for the preparation of phenanthridinone **A**.

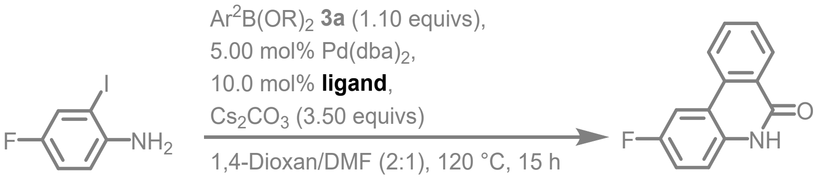

1d **A**

entry	ligand	yield of compound A (%)
1	$[^tBu_3PH]BF_4$	45
2	DTBPF	9

Table S10. Experimental details on the optimization of the reaction temperature for the presentation of diarylamine **B**.

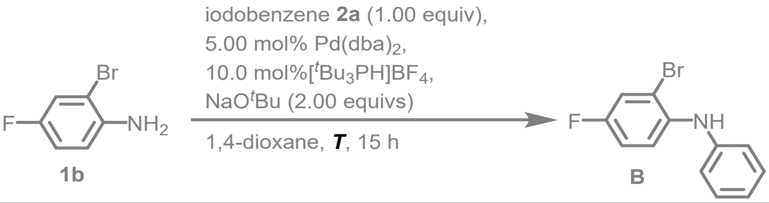

1b **B**

entry	*T* (°C)	yield of compound B (%)
1	20	55
2	30	67
3	35	86
4	40	72
5	50	56
6	60	30

Table S11. Experimental details for optimizing the amount of sodium tert-butoxide used for the preparation of diarylamine **B**.

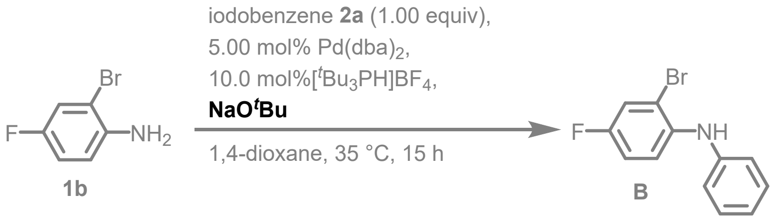

entry	NaO^{t}Bu equivs	yield of compound B (%)
1	1.50	62
2	2.00	86
3	2.50	58

Table S12. Experimental details for optimizing the amount of aryl halide (**2a**) used for the preparation of diarylamine **B**.

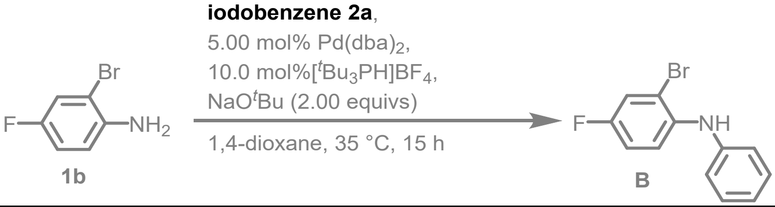

entry	aryl halide 2a equivs	yield of compound B (%)
1	1.10	74
2	1.00	86
3	0.90	64

Table S13. Experimental details on the optimization of the base used for the presentation of diarylamine **B**.

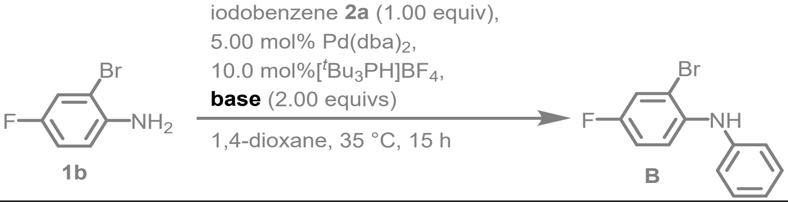

entry	base	yield of compound B (%)
1	Na_2CO_3	1
2	NaO^{t}Bu	86
3	K_2CO_3	1
4	KO^{t}Bu	5
5	Cs_2CO_3	10

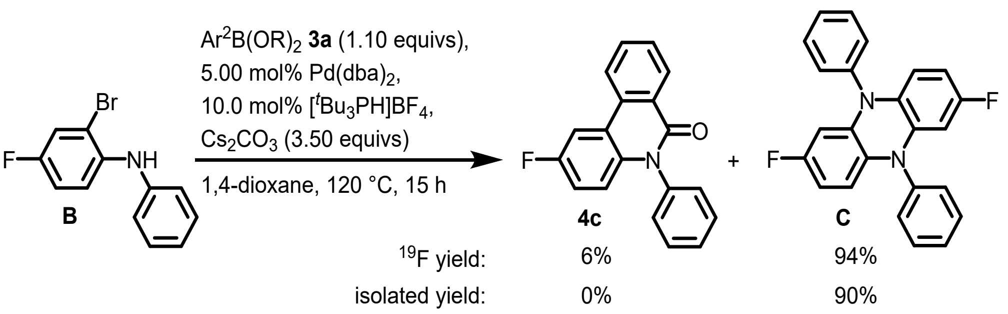

Scheme S1. *Suzuki* coupling and homocoupling of the diarylamine **B**.

Table S14. Experimental details on the optimization of the reaction temperature for the preparation of *N*-arylphenanthridinone **4c**.

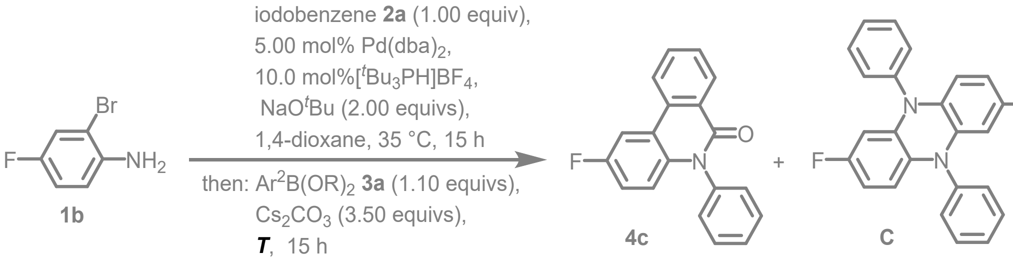

entry	temperature (°C)	yield *N*-arylphenanthridinone 4c (%)	yield dihydrophenazine C (%)
1	20	-	-
2	35	-	-
3	60	-	6
4	80	6	67
5	120	6	94

Table S15. Experimental details on the optimization of the amount of water and the reaction temperature in the *Suzuki* coupling for the preparation of the *N*-arylphenanthridinone **4c**.

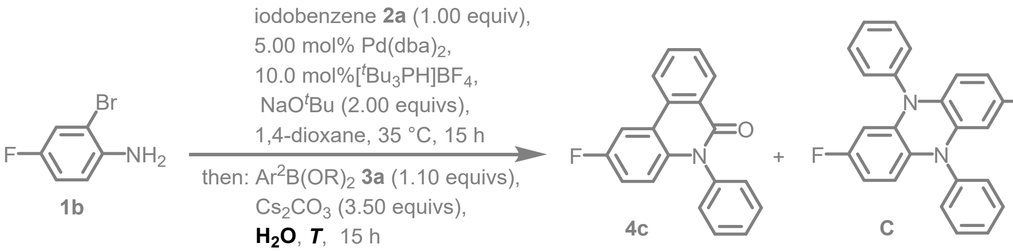

entry	ratio 1,4-dioxane:water	temperature	yield *N*-arylphenanthridinone 4c (%)	yield dihydrophenazine C (%)
1	3:1	80 °C	15	52
2	3:1	100 °C	6	30
3	2:1	120 °C	38	33
4	3:1	120 °C	40	30
5	4:1	120 °C	1	67
6	5:1	120 °C	3	32
7	1:1	120 °C	25	43

Table S16. Experimental details to optimize the amount of boronic acid for the preparation of *N*-arylphenanthridinone **4b**.

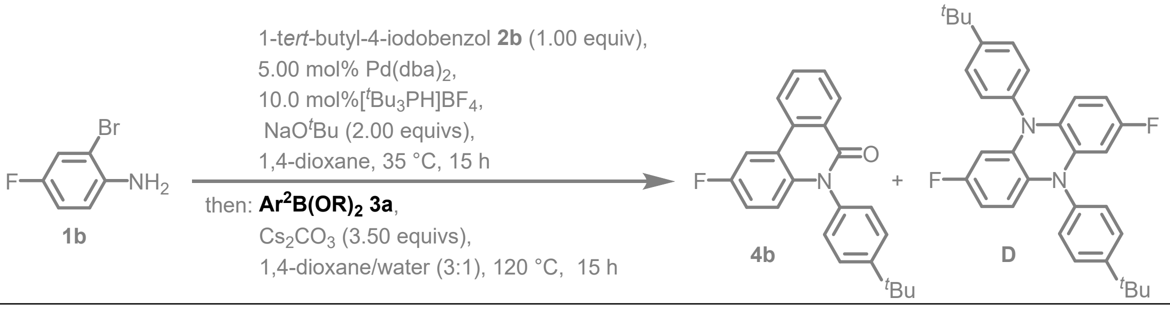

entry	boronic acid 2n equivs	yield *N*-arylphenanthridinone 4b (%)	yield dihydrophenazine D (%)
1	1.10	47	31
2	2.00	20	29
3	2.50	20	30
4	3.00	8	14

Table S17. Experimental details to optimize the amount of boronic acid for the preparation of *N*-arylphenanthridinone **4b**.

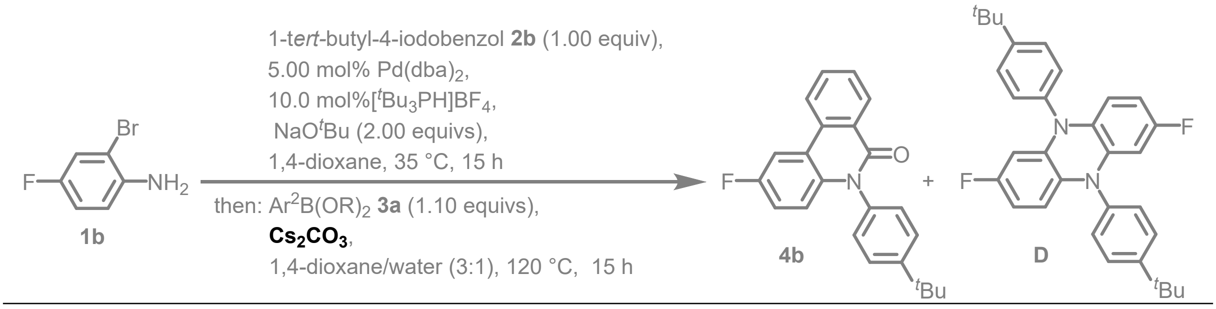

entry	Cs_2CO_3 equivs	yield *N*-arylphenanthridinon 4b (%)	yield dihydrophenazin D (%)
1	0	1	66
2	2.5	37	33
3	3.5	47	31
4	4.5	13	35

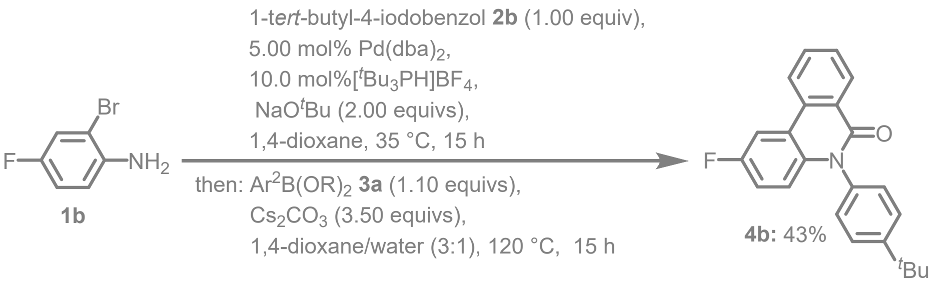

Scheme S2. Optimized reaction conditions for the three-component synthesis of *N*-arylphenanthridinone **4b**.

5.2 General procedure (GP1) for the synthesis of *N*-arylphenanthridinones 4 and *N*-arylcrinasiadines 5

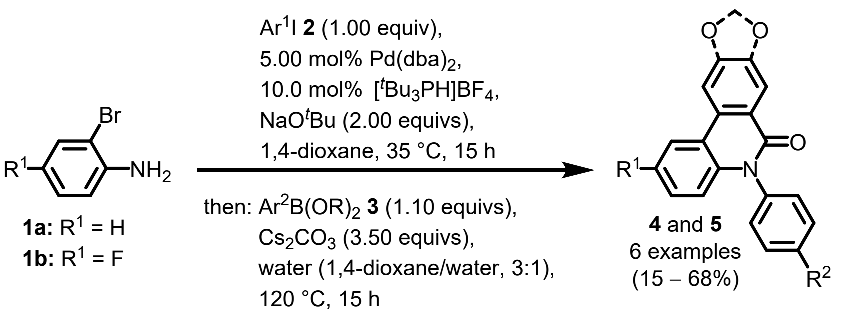

In a sealed Schlenk tube, bromoaniline **1** (0.500 mmol, 1.00 equiv), aryl iodide **2** (0.500 mmol, 1.00 equiv), bis(dibenzylideneacetone)palladium(0) (14.0 mg, 25.0 μmol, 5.00 mol%), tri-*tert*-butylphosphonium tetrafluoroborate (15.0 mg, 50.0 μmol, 10.0 mol%), and sodium *tert*-butoxide (96 mg, 1.00 mmol, 2.00 equiv) were placed and dissolved in dry 1,4-dioxane (3.5 mL). The reaction mixture was then stirred for 15 h at 35 °C. After cooling to room temperature, (hetero)arylboronic acid **3** (0.550 mmol, 1.10 equiv), cesium carbonate (570 mg, 1.75 mmol, 3.50 equiv), and water (1.2 mL) were added under a nitrogen atmosphere. The suspension was then stirred for 15 h at 120 °C. After cooling to room temperature, the reaction mixture was treated with dichloromethane and deionized water, and the organic phase was separated. The aqueous phase was then extracted three times with dichloromethane. The combined organic phases were dried with anhydrous magnesium sulfate, and the solvent was removed under reduced pressure. The crude product was purified by column chromatography and subsequently recrystallized.

Table S18. Experimental details for the synthesis of *N*-arylphenanthridinones **4** and *N*-arylcrinasiadines **5**.

entry	bromoaniline **1**	aryl iodide **2**	arylboronic acid or ester **3**	yield of product **4** or **5**[a]
1	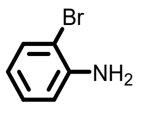<br>**1a**, 86 mg	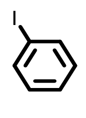<br>**2a**, 120 mg	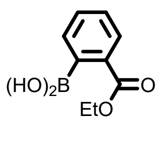<br>**3a**, 107 mg	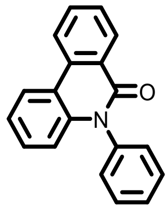<br>**4a**, 21 mg (15%)
2	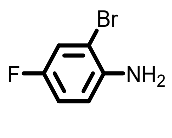<br>**1b**, 95 mg	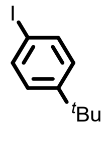<br>**2b**, 130 mg	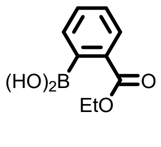<br>**3a**, 107 mg	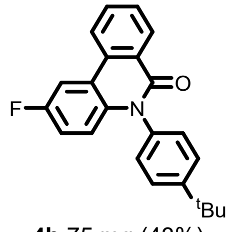<br>**4b**,75 mg (43%)

[a] Yields after flash chromatography on silica gel.

Table S19. Experimental details for the synthesis of *N*-arylphenanthridinones **4** and *N*-arylcrinasiadines **5**.

entry	bromoaniline **1**	aryl iodide **2**	arylboronic acid or ester **3**	yield of product **4** or **5**[a]
3	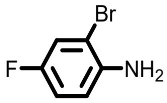 **1b**, 95 mg	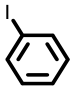 **2a**, 120 mg	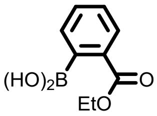 **3a**, 107 mg	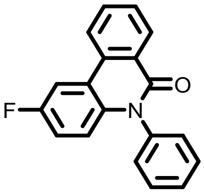 **4c**, 41 mg (28%)
4	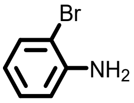 **1a**, 86 mg	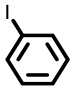 **2a**, 120 mg	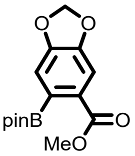 **3b**, 168 mg	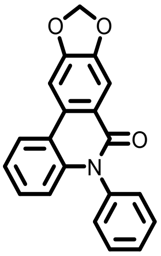 **5a**, 61 mg (39%)
5	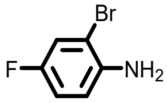 **1b**, 95 mg	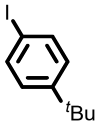 **2b**, 130 mg	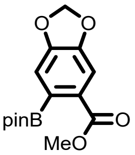 **3b**, 168 mg	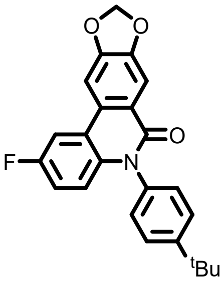 **5b**, 87 mg (47%)
6	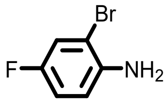 **1b**, 95 mg	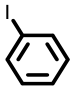 **2a**, 120 mg	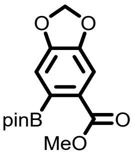 **3b**, 168 mg	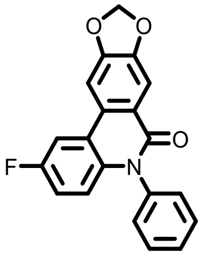 **5c**, 113 mg (68%)

[a] Yields after flash chromatography on silica gel.

5.3 Spectroscopic data of *N*-arylphenanthridinones 4 and *N*-arylcrinasiadines 5

5-Phenylphenanthridin-6(5*H*)-one (4a)

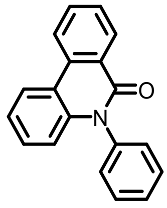

The synthesis was performed by GP1. After chromatography on silica gel (*n*-hexane/ethyl acetate 2:1) and recrystallization from *n*-hexane, compound **4a** (21 mg, 77.0 μmol, 15%) was isolated as colorless crystals.

Mp 219 °C (Lit.: 220 – 222 °C[5]). R_f 0.23 (*n*-hexane/ethyl acetate 5:1). ^{1}H NMR (600 MHz, DMSO-d$_6$) δ 6.55 (dd, ^3J = 8.4 Hz, ^4J = 1.2 Hz, 1 H), 7.33 (td, ^3J = 7.6 Hz, ^4J = 1.2 Hz, 1 H), 7.38-7.42 (m, 3H), 7.56-7.59 (m, 1 H), 7.65 (dd, ^3J = 8.4 Hz, 7.0 Hz, 2 H), 7.70 (ddd, ^3J = 8.0 Hz, 7.1 Hz, ^4J = 1.0 Hz, 1 H), 7.92 (ddd, ^3J = 8.4 Hz, 7.2 Hz, ^4J = 1.5 Hz, 1 H), 8.35 (dd, ^3J = 8.0 Hz, ^4J = 1.4 Hz, 1 H), 8.55 (dd, ^3J = 8.0 Hz, ^4J = 1.6 Hz, 1 H), 8.62 (d, ^3J = 8.2 Hz, 1 H). ^{13}C NMR (150 MHz, DMSO-d$_6$) δ 116.4 (CH), 118.3 (C$_{quat}$), 122.6 (CH), 122.7 (CH), 123.6 (C$_{quat}$), 125.3 (CH), 128.0 (CH), 128.4 (CH), 128.7 (CH), 129.3 (CH), 129.5 (CH), 130.1 (CH), 133.2 (CH), 133.7 (C$_{quat}$), 138.2 (C$_{quat}$), 138.8 (C$_{quat}$), 160.4 (C$_{quat}$). EI MS (70 eV, m/z (%)) 271 (49), 270 ([M]$^+$, 100), 241 ([$C_{17}H_7NO$]$^+$, 28), 121 ([C_7H_7NO]$^+$, 28).

5-(4-(*tert*-Butyl)phenyl)-2-fluorophenanthridin-6(5*H*)-one (4b)

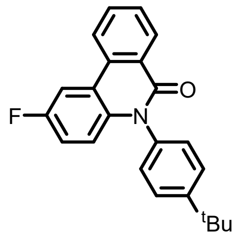

The synthesis was performed by GP1. After chromatography on silica gel (*n*-hexane/ethyl acetate 10:1) and recrystallization from *n*-hexane, compound **4b** (75 mg, 0.217 mmol, 43%) was isolated as colorless crystals.

Mp 208 °C. R_f 0.21 (*n*-hexane/ethyl acetate 10:1). ^{1}H NMR (600 MHz, DMSO-d$_6$) δ 1.39 (s, 9 H), 6.55 (dd, ^3J = 9.3 Hz, ^4J = 4.9 Hz, 1 H), 7.21-7.33 (m, 3 H), 7.59-7.68 (m, 2 H), 7.73 (ddd, ^3J = 8.2 Hz, 7.2 Hz, ^{4}J = 1.1 Hz, 1 H), 7.93 (ddd, ^3J = 8.4 Hz, 7.1 Hz, ^4J = 1.5 Hz, 1 H), 8.35 (dd, ^3J = 8.0 Hz, ^4J = 1.4 Hz, 1 H), 8.42 (dd, ^3J = 10.2 Hz, ^4J = 2.9 Hz, 1 H), 8.62 (d, ^3J = 8.2 Hz, 1 H). ^{13}C NMR (150 MHz, DMSO-d$_6$) δ 31.2 (CH$_3$), 34.6 (C$_{quat}$), 109.6 (d, CH, ^2J = 24.2 Hz), 116.9 (d, CH, ^2J = 23.5 Hz), 118.3 (d, CH, ^3J = 8.8 Hz), 119.8 (d, C$_{quat}$, ^3J = 8.6 Hz), 123.2 (CH), 125.5 (C$_{quat}$), 126.9 (CH), 128.1 (CH), 128.7 (CH), 129.0 (CH), 132.9 (d, C$_{quat}$, ^4J = 2.8 Hz), 133.2 (CH), 135.4 (C$_{quat}$), 135.6 (C$_{quat}$), 151.2 (C$_{quat}$), 158.0 (d, C$_{quat}$, ^2J = 239.4 Hz), 160.2 (C$_{quat}$). EI MS (70 eV, m/z (%)) 346 (19), 345 ([M]$^+$, 69), 331 (26), 330 ([$C_{23}H_{21}FN$]$^+$, 100), 196 ([$C_{13}H_5FO$]$^+$, 33), 151 (35). IR ($\tilde{\nu}$ [cm^{-1}]) 1647 (s), 1607 (w), 1589 (m), 1570 (w), 1497 (m), 1460 (w), 1447 (w), 1423 (m), 1408 (w), 1393 (w), 1341 (m), 1331 (m), 1308 (m), 1290 (w), 1265 (w), 1231 (w), 1184 (m), 1136 (m), 1105 (w), 1042 (w), 1022 (w), 970 (w), 899 (m),

889 (m), 839 (m), 824 (m), 802 (s), 772 (s), 716 (m), 692 (m), 665 (m), 617 (s). HRMS (ESI) calcd. for $[C_{23}H_{20}FNO]^+$ 346.1602, found 346.1605. HPLC (ethyl acetate): 95% (6.8 min).

2-Fluoro-5-phenylphenanthridin-6(5*H*)-one (4c)

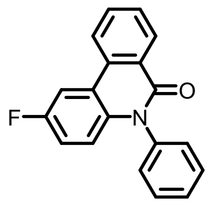

The synthesis was performed by GP1. After chromatography on silica gel (*n*-hexane/ethyl acetate 2:1) and recrystallization from *n*-hexane, compound **4c** (41 mg, 0.142 mmol, 28%) was isolated as colorless crystals.

Mp 286 °C. R_f 0.07 (*n*-hexane/ethyl acetate 10:1). ^{1}H NMR (600 MHz, DMSO-d$_6$) δ 6.55 (dd, ^3J = 9.2 Hz, ^4J = 4.9 Hz, 1H), 7.28 (ddd, ^3J = 9.3 Hz, 8.0 Hz, ^4J = 2.9 Hz, 1 H), 7.37-7.44 (m, 2 H), 7.50-7.62 (m, 1 H), 7.63-7.69 (m, 2 H), 7.74 (ddd, ^3J = 8.1 Hz, 7.2 Hz, ^4J = 1.1 Hz, 1 H), 7.93 (ddd, ^3J = 8.3 Hz, 7.2 Hz, ^4J = 1.5 Hz, 1 H), 8.35 (dd, ^3J = 7.9 Hz, ^4J = 1.0 Hz, 1 H), 8.44 (dd, ^3J = 10.3 Hz, ^4J = 2.9 Hz, 1 H), 8.63 (d, ^3J = 8.6 Hz, 1 H). ^{13}C NMR (150 MHz, DMSO-d$_6$) δ 109.7 (d, CH, ^2J = 24.3 Hz), 116.8 (d, CH, ^2J = 23.5 Hz), 118.2 (d, CH, ^3J = 8.6 Hz), 119.8 (d, C$_{quat}$, ^3J = 8.4 Hz), 123.3 (CH), 125.5 (CH), 128.1 (CH), 128.8 (CH), 129.1 (CH), 129.3 (CH), 130.2 (CH), 132.9 (d, C$_{quat}$, ^4J = 2.5 Hz), 133.3 (C$_{quat}$), 135.6 (C$_{quat}$), 138.1 (C$_{quat}$), 158.0 (d, C$_{quat}$, ^1J = 239.2 Hz), 160.1 (C$_{quat}$). EI MS (70 eV, m/z (%)) 290 (18), 289 ([M]$^+$, 89), 288 ($[C_{19}H_{11}FNO]^+$, 100), 259 ($[C_{19}H_{17}N]^+$, 17). IR ($\tilde{\nu}$ [cm^{-1}]) 1651 (s), 1609 (w), 1587 (w), 1564 (w), 1539 (w), 1491 (m), 1445 (w), 1422 (w), 1335 (m), 1310 (w), 1285 (w), 1269 (w), 1227 (w), 1213 (w), 1184 (m), 1173 (w), 1152 (w), 1136 (m), 1123 (w), 1030 (w), 962 (w), 916 (w), 897 (w), 866 (m), 827 (m), 766 (m), 756 (m), 727 (m), 716 (m), 691 (s), 667 (w), 650 (m), 610 (m). HRMS (ESI) calcd. for $[C_{19}H_{12}FNO+H]^+$ 290.0976, found 290.0967. HPLC (acetonitrile): 99% (5.2 min).

5-Phenyl-[1,3]dioxolo[4,5-j]phenanthridin-6(5*H*)-one (5a)

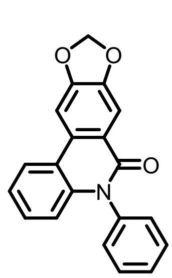

The synthesis was performed by GP1. After chromatography on silica gel (*n*-hexane/ethyl acetate 2:1) and recrystallization from *n*-hexane, compound **5a** (61 mg, 0.193 mmol, 39%) was isolated as colorless crystals.

Mp 232 °C. R_f 0.10 (*n*-hexane/ethyl acetate 5:1). ^{1}H NMR (600 MHz, DMSO-d$_6$) δ 6.26 (s, 2 H), 6.51 (dd, ^3J = 8.4 Hz, ^4J = 1.2 Hz, 1 H), 7.28 (ddd, ^3J = 8.2 Hz, 7.1 Hz, ^4J = 1.2 Hz, 1 H), 7.32-7.34 (m, 1 H), 7.35-7.37 (m, 2 H), 7.56 (tt, , ^3J = 6.8 Hz, ^4J = 1.2 Hz, 1 H), 7.62-7.65 (m, 2 H), 7.66 (s, 1 H), 8.15 (s, 1 H), 8.44 (dd, ^3J = 8.1 Hz, ^3J = 1.5 Hz, 1 H). ^{13}C NMR (150 MHz, DMSO-d$_6$) δ 101.5 (CH), 102.4 (CH$_2$), 105.5 (CH), 116.2 (CH), 118.4 (C$_{quat}$), 120.6 (C$_{quat}$), 122.5

(CH), 123.6 (CH), 128.6 (CH), 128.8 (CH), 129.3 (CH), 130.0 (CH), 130.7 (C_{quart}), 138.1 (C_{quat}), 138.2 (C_{quat}), 148.4 (C_{quat}), 152.5 (C_{quat}), 159.7 (C_{quat}). EI MS (70 eV, m/z (%)) 316 (22), 315 (95), 314 ($[M]^+$, 100), 256 ($[C_{19}H_{14}N]^+$, 18), 228 ($[C_{14}H_{14}NO_2]^+$, 22), 114 (31), 77 ($[C_6H_5]^+$, 13). IR ($\tilde{\nu}$ [cm^{-1}]) 1649 (m), 1636 (m), 1622 (m), 1599 (m), 1574 (w), 1499 (m), 1479 (m), 1456 (s), 1393 (m), 1341 (m), 1310 (m), 1269 (m), 1252 (m), 1238 (m), 1196 (m), 1165 (w), 1144 (w), 1119 (w), 1069 (m), 1030 (s), 1005 (w), 974 (w), 928 (m), 903 (w), 881 (w), 833 (w), 814 (w), 781 (w), 758 (m), 739 (s), 694 (s), 675 (m), 635 (m), 611 (w). Anal. calcd. for $C_{20}H_{13}NO_3$ [315.1] C 76.18, H 4.16, N 4.44; Found C 76.27, H 4.18, N 4.26.

5-(4-(*tert*-Butyl)phenyl)-2-fluoro-[1,3]dioxolo[4,5-j]phenanthridin-6(5*H*)-one (5b)

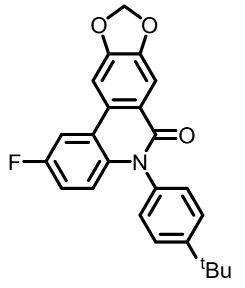

The synthesis was performed by GP1. After chromatography on silica gel (*n*-hexane/ethyl acetate 5:1) and recrystallization from *n*-hexane, compound **5b** (87 mg, 0.223 mmol, 45%) was isolated as colorless crystals.

Mp 296 °C. R_f 0.23 (*n*-hexane/ethyl acetate 5:1). ^{1}H NMR (600 MHz, DMSO-d$_6$) δ 1.38 (s, 9 H), 6.27 (s, 2 H), 6.51 (dd, ^3J = 9.2 Hz, ^4J = 5.0 Hz, 1 H), 7.24 (ddd, ^3J = 9.2 Hz, 7.9 Hz, ^4J = 2.8 Hz, 1 H), 7.26-7.29 (m, 2 H), 7.59-7.67 (m, 3 H), 8.16 (s, 1 H), 8.32 (dd, ^3J = 10.5 Hz, ^3J = 2.9 Hz, 1 H). ^{13}C NMR (150 MHz, DMSO-d$_6$) δ 30.9 (CH_3), 101.8 (CH), 102.3 (CH_2), 105.3 (CH), 109.2 (d, CH, ^2J = 23.8 Hz), 115.8 (d, CH, ^2J = 23.9 Hz), 117.8 (d, CH, ^3J = 8.6 Hz), 119.5 (d, C_{quat}, ^3J = 8.4 Hz), 120.8 (C_{quat}), 126.6 (CH), 128.4 (CH), 129.6 (d, C_{quat}, ^4J = 2.7 Hz), 134.7 (C_{quat}), 135.2 (C_{quat}), 148.6 (C_{quat}), 150.8 (C_{quat}), 152.2 (C_{quat}), 157.7 (d, C_{quat}, ^1J = 238.2 Hz), 159.3 (C_{quat}). EI MS (70 eV, m/z (%)) 390 (22), 389 ($[M]^+$, 75), 388 (11), 375 ($[C_{24}H_{22}FNO_2]^+$, 29), 374 ($[C_{23}H_{18}FNO_3]^+$, 100), 332 ($[C_{20}H_{11}FNO_3]^+$, 13), 240 ($[C_{16}H_{15}FN]^+$, 23), 182 (21), 173 ($[C_{11}H_{11}NO]^+$, 36). IR ($\tilde{\nu}$ [cm^{-1}]) 1649 (s), 1622 (m), 1584 (w), 1501 (s), 1485 (s), 1456 (s), 1429 (w), 1406 (w), 1383 (m), 1362 (w), 1341 (w), 1315 (m), 1298 (m), 1277 (w), 1256 (s), 1204 (s), 1177 (m), 1146 (w), 1113 (w), 1059 (w), 1036 (s), 1007 (m), 968 (w), 924 (m), 899 (m), 860 (s), 839 (w), 810 (s), 779 (w), 725 (w), 716 (w), 687 (w), 656 (m), 638 (w), 617 (s). HRMS (ESI) calcd. for $[C_{24}H_{20}FNO_3+H]^+$ 390.1500, found 390.1505. HPLC (acetone): 99% (6.8 min).

2-Fluoro-5-Phenyl-[1,3]dioxolo[4,5-j]phenanthridin-6(5*H*)-one (5c)

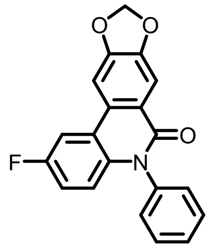

The synthesis was performed by GP1. After chromatography on silica gel (*n*-hexane/ethyl acetate 2:1) and recrystallization from *n*-hexane, compound **5c** (113 mg, 0.339 mmol, 68%) was isolated as colorless crystals.

Mp 320 °C. R_f 0.09 (*n*-hexane/ethyl acetate 5:1). ^{1}H NMR (600 MHz, DMSO-d$_6$) δ 6.28 (s, 2 H), 6.51 (dd, ^3J = 9.2 Hz, ^4J = 5.0 Hz, 1 H), 7.22 (ddd, ^3J = 9.3 Hz, 7.9 Hz, ^4J = 2.9 Hz, 1 H), 7.35-7.41 (m, 2 H), 7.54-7.60 (m, 1 H), 7.61-7.69 (m, 3 H), 8.18 (s, 1 H), 8.34 (dd, ^3J = 10.5 Hz, ^4J = 2.9 Hz, 1 H). ^{13}C NMR (150 MHz, DMSO-d$_6$) δ 102.1 (CH), 102.6 (CH$_2$), 105.6 (CH), 108.9 (d, C$_{quart}$, ^3J = 7.6 Hz), 109.5 (d, CH, ^2J = 24.3 Hz), 117.6 (d, CH, ^3J = 7.0 Hz), 121.1 (C$_{quart}$), 128.8 (CH), 129.3 (CH), 129.8 (d, CH, ^2J = 27.7 Hz), 130.1 (CH), 131.1 (d, C$_{quart}$, ^4J = 3.7 Hz), 138.2 (C$_{quart}$), 140.7 (C$_{quart}$), 140.8 (C$_{quart}$), 148.9 (C$_{quart}$), 152.5 (C$_{quart}$), 159.5 (d, C$_{quart}$, ^1J = 238.2 Hz). EI MS (70 eV, m/z (%)) 334 (15), 333 (69), 332 ([M]$^+$, 100), 274 ([$C_{18}H_{12}NO_2$]$^+$, 23), 246 ([$C_{14}H_{13}FNO_2$]$^+$, 26), 245 ([$C_{14}H_{12}FNO_2$]$^+$, 11), 182 ([$C_{12}H_5FN$]$^+$, 14), 138 (13), 123 ([$C_7H_7O_2$]$^+$, 21). IR ($\tilde{\nu}$ [cm^{-1}]) 1647 (s), 1626 (w), 1611 (w), 1582 (w), 1562 (w), 1504 (m), 1485 (s), 1452 (m), 1423 (m), 1381 (m), 1342 (w), 1315 (m), 1302 (m), 1287 (w), 1269 (w), 1254 (m), 1240 (m), 1196 (m), 1177 (m), 1140 (w), 1119 (w), 1105 (w), 1076 (w), 1061 (w), 1038 (s), 1003 (m), 991 (w), 968 (w), 937 (s), 887 (w), 852 (s), 812 (m), 793 (w), 779 (w), 758 (s), 727 (w), 712 (w), 696 (s), 654 (s), 625 (s), 602 (s). HRMS (ESI) calcd. for [$C_{20}H_{12}FNO_3$+H]$^+$ 334.0874, found 334.0855. HPLC (acetonitrile): 99% (5.2 min).

6 Synthesis of *N*-alkylphenanthridinones 6 and *N*-alkylcrinasiadines 7

6.1 Optimization of *N*-alkylphenanthridinones 6 and *N*-alkylcrinasiadines 7

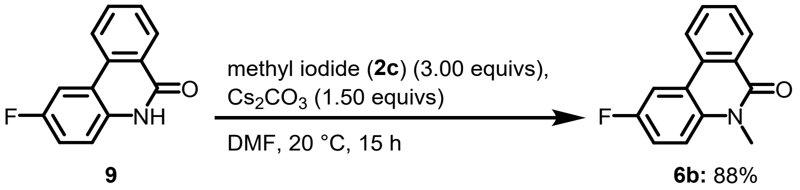

Scheme S3. Preparation of the *N*-alkylphenanthridinone **6b** via nucleophilic substitution with methyl iodide (**2c**).

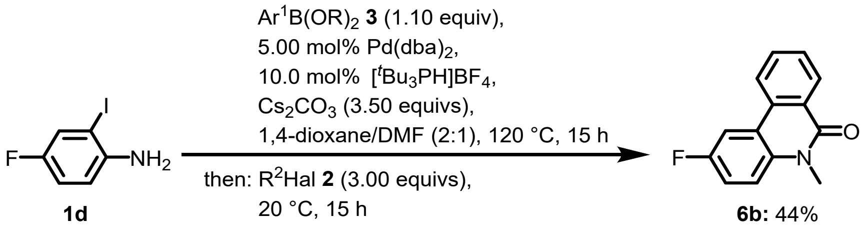

Scheme S4. Optimized reaction conditions for the three-component synthesis of *N*-alkylphenanthridinone **6b**.

6.2 General procedure (GP2) for the synthesis of *N*-alkylphenanthridinones 6 and *N*-alkylcrinasiadines 7

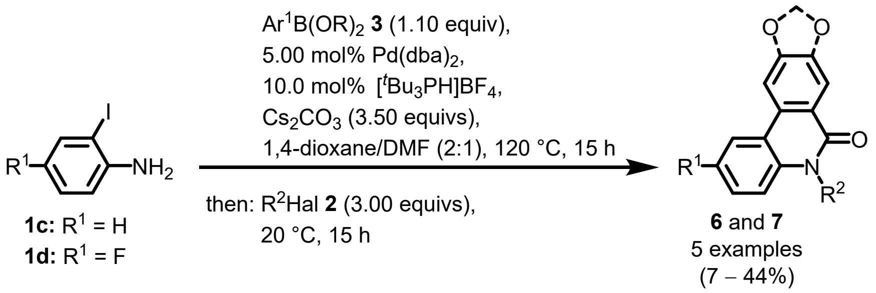

In a sealed Schlenk tube, 2-iodoaniline **1** (0.500 mmol, 1.00 equiv), (hetero)arylboronic acid **3** (0.550 mmol, 1.10 equiv), bis(dibenzylideneacetone)palladium(0) (14.0 mg, 25.0 µmol, 5.00 mol%), tri-*tert*-butylphosphonium tetrafluoroborate (15.0 mg, 50.0 µmol, 10.0 mol%), and cesium carbonate (570 mg, 1.75 mmol, 3.50 equiv) were placed and dissolved in dry 1,4-dioxane (2.3 mL) and dry DMF (1.2 mL). The reaction mixture was then stirred for 15 h at 120 °C. After cooling to room temperature, alkyl halide **2** (1.50 mmol, 3.00 equiv) was added under a nitrogen atmosphere, and the suspension was stirred for 15 h at 20 °C. After cooling to room temperature, the reaction mixture was treated with dichloromethane and deionized water, and the organic phase was separated. The aqueous phase was then extracted three times with dichloromethane. The combined organic phases were dried with anhydrous magnesium sulfate, and the solvent was removed under reduced pressure. The crude product was purified by column chromatography and subsequently recrystallized.

Table S20. Experimental details for the synthesis of *N*-alkylphenanthridinones **6** and *N*-alkylcrinasiadines **7**.

entry	iodoaniline **1**	arylboronic acid or ester **3**	aryl halide **2**	yield of product **6** or **7**[a]
1	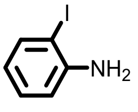 **1c**, 110 mg	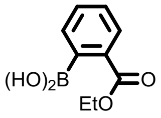 **3a**, 107 mg	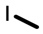 **2c**, 213 mg	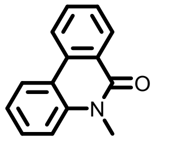 **6a**, 42 mg (40%)
2	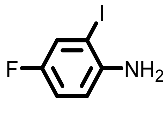 **1d**, 119 mg	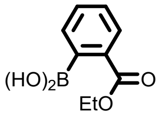 **3a**, 107 mg	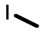 **2c**, 213 mg	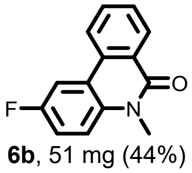 **6b**, 51 mg (44%)

[a] Yields after flash chromatography on silica gel.

Table 20. Experimental details for the synthesis of *N*-alkylphenanthridinones **6** and *N*-alkylcrinasiadines **7**.

entry	iodoaniline **1**	arylboronic acid or ester **3**	aryl halide **2**	yield of product **6** or **7**[a]
3	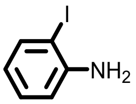 **1c**, 110 mg	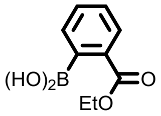 **3a**, 107 mg	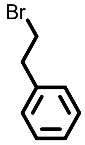 **2d**, 278 mg	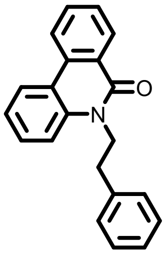 **6c**, 27 mg (17%)
4	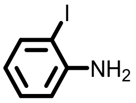 **1c**, 110 mg	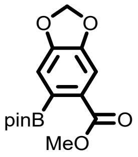 **3b**, 168 mg	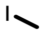 **2c**, 213 mg	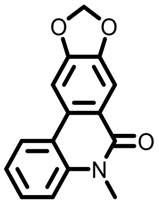 **7a**, 47 mg (37%)
5	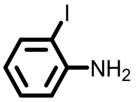 **1c**, 110 mg	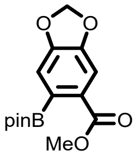 **3b**, 168 mg	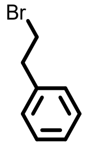 **2d**, 278 mg	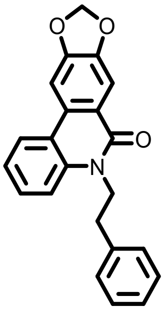 **7b**, 12 mg (7%)

[a] Yields after flash chromatography on silica gel.

6.3 Spectroscopic data of *N*-alkylphenanthridinones 6 and *N*-alkylcrinasiadines 7

5-Methylphenanthridin-6(5*H*)-one (6a)

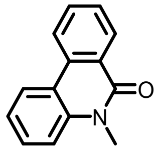

The synthesis was performed by GP2. After chromatography on silica gel (*n*-hexane/ethyl acetate 2:1) and recrystallization from *n*-hexane, compound **6a** (42 mg, 0.201 mmol, 40%) was isolated as colorless crystals.

Mp 111 °C (Lit.: 108 – 110 °C[6]). R_f 0.52 (*n*-hexane/ethyl acetate 2:1). ^{1}H NMR (300 MHz, $CDCl_3$) δ 3.82 (s, 3 H), 7.33 (ddd, ^3J = 8.2 Hz, 7.1 Hz, ^4J = 1.2 Hz, 1 H), 7.42 (dd, ^3J = 8.5 Hz, ^4J = 1.2 Hz, 1 H), 7.57 (dddd, ^3J = 9.9 Hz, 8.6 Hz, 7.1 Hz, ^4J = 1.3 Hz, 2 H), 7.76 (ddd, ^3J = 8.4 Hz, 7.2 Hz, ^4J = 1.5 Hz, 1 H), 8.25-8.33 (m, 2 H), 8.56 (ddd, ^3J = 8.0 Hz, ^4J = 1.5 Hz, 0.6 Hz, 1 H). ^{13}C NMR (150 MHz, $CDCl_3$) δ 30.1 (CH_3), 115.2 (CH), 119.5 (CH), 121.8 (C_{quat}), 122.6 (CH), 123.4 (C_{quat}), 125.8 (C_{quat}), 128.1 (CH), 129.1 (CH), 129.7 (CH), 132.6 (CH), 133.7 (CH), 138.2 (C_{quat}), 161.8 (C_{quat}). EI MS (70 eV, m/z (%)) 209 ($[M]^+$, 100), 180 ($[C_{13}H_{10}N]^+$, 35), 178 ($[C_{13}H_8N]^+$, 33), 152 ($[C_{12}H_8]^+$, 19).

2-Fluoro-5-methylphenanthridin-6(5*H*)-one (6b)

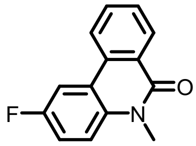

The synthesis was performed by GP2. After chromatography on silica gel (*n*-hexane/ethyl acetate 1:1) and recrystallization from *n*-hexane, compound **6b** (51 mg, 0.224 mmol, 44%) was isolated as colorless crystals.

Mp 170 – 171 °C (Lit.: 126 – 128 °C[7]). R_f 0.20 (*n*-hexane/ethyl acetate 5:1). ^{1}H NMR (600 MHz, DMSO-d_6) δ 3.72 (s, 3 H), 7.47 (ddd, ^3J = 9.2 Hz, 8.0 Hz, ^4J = 2.9 Hz, 1 H), 7.60 (dd, ^3J = 9.2 Hz, 4.8 Hz, 1 H), 7.68 (ddd, ^3J = 8.0 Hz, 7.1 Hz, ^4J = 1.1 z, 1 H), 7.85 (ddd, ^3J = 8.3 Hz, 7.1 Hz, ^4J = 1.5 Hz, 1 H), 8.33 8.38 (m, 2 H), 8.53 (d, ^3J = 8.1 Hz, 1 H). ^{13}C NMR (150 MHz, DMSO-d_6) δ 30.0 (CH_3), 109.6 (d, CH, ^2J = 23.9 Hz), 117.0 (d, CH, ^2J = 23.4 Hz), 117.6 (d, CH , ^3J = 8.3 Hz), 120.0 (d, C_{quat}, ^3J = 8.1 Hz), 123.0 (CH), 125.2 (C_{quat}), 128.1 (CH), 128.8 (CH), 132.4 (d, C_{quat}, ^4J = 2.9 Hz), 132.8 (CH), 134.3 (C_{quat}), 158.0 (d, C_{quat}, ^1J = 238.8 Hz), 160.1 (C_{quat}). EI MS (70 eV, m/z (%)) 227 ($[M]^+$, 100), 226 ($[C_{14}H_9FNO]^+$, 24), 198 ($[C_{13}H_9FN]^+$, 31), 196 ($[C_{13}H_7FN]^+$, 31), 170 ($[C_{11}H_8NO]^+$, 20).

5-Phenethylphenanthridin-6(5*H*)-one (6c)

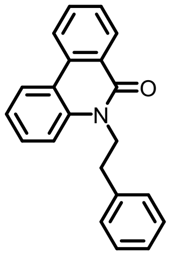

The synthesis was performed by GP2. After chromatography on silica gel (*n*-hexane/ethyl acetate 10:1) and recrystallization from *n*-hexane, compound **6c** (27 mg, 0.085 mmol, 17%) was isolated as colorless crystals.

Mp 91 °C (Lit.: 81.2 – 82.2 °C[8]). R_f 0.29 (*n*-hexane/ethyl acetate 10:1). ^{1}H NMR (600 MHz, DMSO-d$_6$) δ 2.91-3.05 (m, 2 H), 4.46-4.65 (m, 2 H), 7.22-7.27 (m, 1 H), 7.34 (dd, ^3J = 8.4 Hz, 6.8 Hz, 2 H), 7.36-7.41 (m, 3 H), 7.63-7.69 (m, 2 H), 7.72 (dd, ^3J = 8.6 Hz, ^4J = 1.1 Hz, 1 H), 7.87 (ddd, ^3J = 8.3 Hz, 7.1 Hz, ^4J = 1.5 Hz, 1 H), 8.38 (ddd, ^3J = 7.9 Hz, ^4J =1.5, 0.5 Hz, 1 H), 8.54 (dd, ^3J = 8.1 Hz, ^4J = 1.5 Hz, 1 H), 8.56 (dd, ^3J = 8.1 Hz, ^4J = 0.9 Hz, 1 H). ^{13}C NMR (150 MHz, DMSO-d$_6$) δ 33.1 (CH$_2$), 43.4 (CH$_2$), 115.5 (CH), 118.6 (CH), 122.5 (CH), 123.9 (CH), 124.8 (C$_{quat}$), 126.5 (CH), 128.0 (CH), 128.2 (CH), 128.5 (CH), 128.8 (CH), 130.2 (CH), 132.9 (CH), 133.3 (C$_{quat}$), 136.5 (C$_{quat}$), 138.4 (C$_{quat}$),138.5 (C$_{quat}$), 160.1 (C$_{quat}$). EI MS (70 eV, m/z (%)) 299 ([M]$^+$, 100), 208 ([$C_{14}H_{10}NO$]$^+$, 14), 196 (16), 195 ([$C_{13}H_9NO$]$^+$, 100), 178 ([$C_{13}H_8N$]$^+$, 75), 152 ([$C_{11}H_6N$]$^+$, 20), 151 (12), 91 ([C_7H_7]$^+$, 16), 77 ([C_6H_5]$^+$, 12).

5-Methyl-[1,3]dioxolo[4,5-j]phenanthridin-6(5*H*)-one (7a)

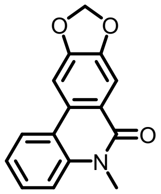

The synthesis was performed by GP2. After chromatography on silica gel (*n*-hexane/ethyl acetate 2:1) and recrystallization from *n*-hexane, compound **7a** (47 mg, 0.186 mmol, 37%) was isolated as colorless crystals.

Mp 244 °C (Lit.: 243 – 245 °C[9]). R_f 0.12 (*n*-hexane/ethyl acetate 5:1). ^{1}H NMR (300 MHz, DMSO-d$_6$) δ 3.71 (s, 3 H), 6.23 (s, 2 H), 7.32 (ddd, ^3J = 8.2 Hz, 6.4 Hz, ^4J = 1.8 Hz, 1 H), 7.53-7.59 (m, 2 H), 7.69 (s, 1 H), 8.07 (s, 1 H), 8.40 (dd, ^3J = 8.0 Hz, ^3J = 1.3 Hz, 1 H). ^{13}C NMR (150 MHz, DMSO-d$_6$) δ 29.7 (CH$_3$), 101.3 (CH), 102.3 (CH$_2$), 105.6 (CH), 115.4 (CH), 118.5 (C$_{quat}$), 120.4 (C$_{quat}$), 122.3 (CH), 123.6 (CH), 129.2 (CH), 130.1 (C$_{quat}$), 137.0 (C$_{quat}$), 148.2 (C$_{quat}$), 152.1 (C$_{quat}$), 159.7 (C$_{quat}$). EI MS (70 eV, m/z (%)) 254 (17), 253 ([M]$^+$, 100), 252 (14).

5-Phenethyl-[1,3]dioxolo[4,5-j]phenanthridin-6(5*H*)-one (7b)

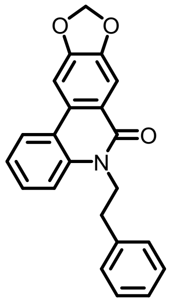

The synthesis was performed by GP2. After chromatography on silica gel (*n*-hexane/ethyl acetate 5:1) and recrystallization

from *n*-hexane, compound **7b** (12 mg, 0.035 mmol, 7%) was isolated as colorless crystals.

Mp 154 °C (Lit.: 154 – 157 °C[9]). R_f 0.21 (*n*-hexane/ethyl acetate 5:1). ^{1}H NMR (600 MHz, DMSO-d$_6$) δ 2.90-3.07 (m, 2 H), 4.47-4.60 (m, 2 H), 6.24 (s, 2 H), 7.23-7.27 (m, 1 H), 7.30-7.35 (m, 3 H), 7.36-7.40 (m, 2H), 7.59 (ddd, ^3J = 8.5 Hz, 7.0 Hz, ^4J = 1.4 Hz, 1 H), 7.68 (dd, ^3J = 8.6 Hz, ^4J = 1.1 Hz, 1 H), 7.69 (s, 1 H), 8.08 (s, 1 H), 8.43 (dd, ^3J = 8.3 Hz, ^4J = 1.4 Hz, 1 H). ^{13}C NMR (150 MHz, DMSO-d$_6$) δ 33.2 (CH$_2$), 43.4 (CH$_2$), 101.3 (CH), 102.3 (CH$_2$), 105.5 (CH), 115.3 (CH), 118.7 (C$_{quat}$), 120.3 (C$_{quat}$), 122.3 (CH), 123.9 (CH), 126.5 (CH), 128.5 (CH), 128.8 (CH), 129.4 (CH), 130.2 (C$_{quat}$), 135.9 (C$_{quat}$), 138.5 (C$_{quat}$), 148.2 (C$_{quat}$), 152.3 (C$_{quat}$), 159.4 (C$_{quat}$). EI MS (70 eV, m/z (%)) 344 (18), 343 ([M]$^+$, 47), 252 ([$C_{15}H_{10}NO_3$]$^+$, 24), 240 (17), 239 ([$C_{14}H_8NO_3$]$^+$, 100), 223 (17), 222 ([$C_{14}H_8NO_2$]$^+$, 62), 91 ([C_7H_7]$^+$, 14).

7 Synthesis of 6(*5H*)-phenanthridinone 8

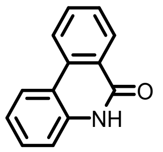

In a sealed Schlenk tube were placed 2-iodoaniline (219 mg, 1.00 mmol, 1.00 equiv), 2-(ethoxycarbonyl)phenylboronic acid **3a** (233 mg, 1.20 mmol, 1.20 equiv), bis(dibenzylideneacetone)palladium(0) (29.0 mg, 50.0 µmol, 5.00 mol%), tri tert-butylphosphonium tetrafluoroborate (29.0 mg, 100 µmol, 10.0 mol%) and cesium carbonate (1.14 g, 3.50 mmol, 3.50 equiv) and the mixture was dissolved in dry 1,4-dioxane (4 mL). The reaction mixture was degassed with nitrogen for 5 min and then stirred for 15 h at 120 °C. After cooling to room temperature, deionized water and dichloromethane were added to the resulting suspension and the organic layer was separated. The aqueous phase was extracted three times with dichloromethane. The combined organic phases were dried over anhydrous magnesium sulfate and the solvent was removed under reduced pressure. The crude product was purified by column chromatography (*n*-hexane/ethyl acetate, 1:1). After final recrystallization from acetone, product **8** was obtained as a colorless solid in 71% yield (138 mg, 0.707 mmol).

Mp 295°C (Lit.: 295 – 296 °C[11]). R_f 0.46 (*n*-hexane/ethyl acetate 1:1). ^{1}H NMR (600 MHz, DMSO-d$_6$) δ 7.24-7.29 (m, 1 H), 7.37 (dd, ^3J = 8.2 Hz, ^4J = 1.2 Hz, 1 H), 7.46-7.52 (m, 1 H), 7.64 (t, ^3J = 7.5 Hz, 1 H), 7.83-7.88 (m, 1 H), 8.32 (dd, ^3J = 8.0 Hz, ^4J = 1.5 Hz, 1 H), 8.39 (d, ^3J = 8.0 Hz, 1 H), 8.51 (d, ^3J = 8.1 Hz, 1 H), 11.68 (s, 1 H). ^{13}C NMR (150 MHz, DMSO-d$_6$) δ 116.1 (CH), 117.5 (C_{quart}), 122.3 (CH), 122.6 (CH), 123.3 (CH), 125.7 (C_{quart}), 127.5 (CH), 127.9 (CH), 129.6 (CH), 132.8 (CH), 134.3 (C_{quart}), 136.6 (C_{quart}), 160.8 (C_{quart}). EI MS (70 eV, m/z (%)) 195 ([M]$^+$, 100), 167 ([$C_{13}H_{11}$]$^+$, 40), 166 ([$C_{13}H_{10}$]$^+$, 21), 139 (15). Anal. calcd. for $C_{13}H_9NO$ [195.2] C 79.98, H 4.65, N 7.17; Found C 80.12, H 4.46, N 7.15.

8 ^{1}H and ^{13}C spectra of *N*-arylphenanthridinones 4 and *N*-arylcrinasiadines 5

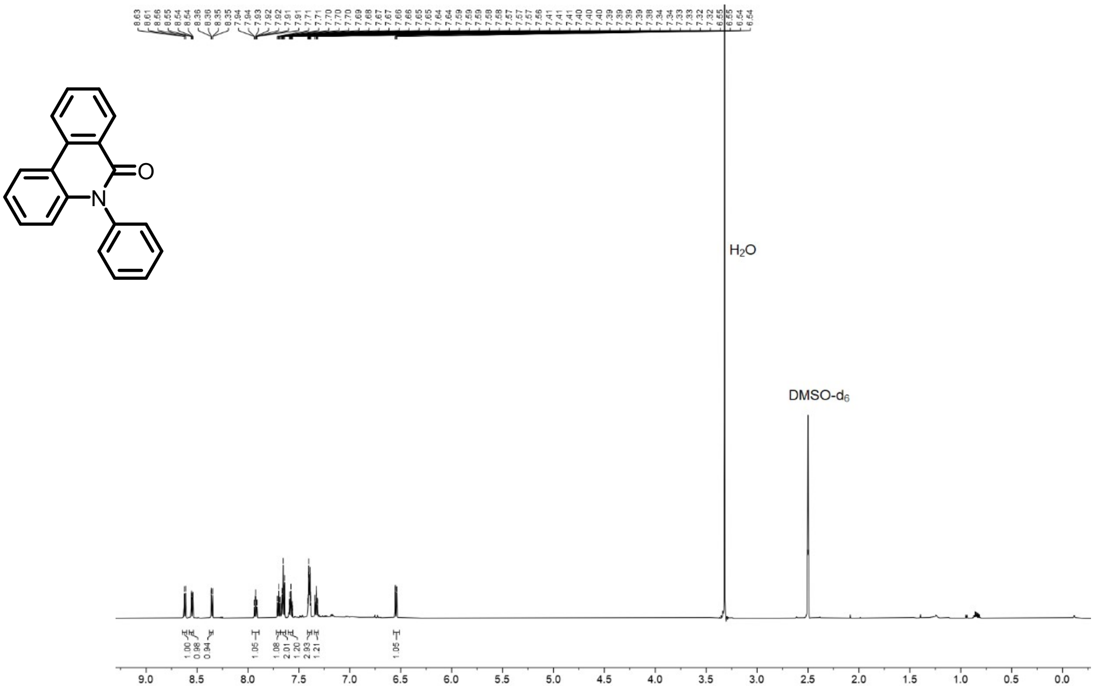

Figure S1. ^{1}H NMR spectrum of 5-Phenylphenanthridin-6(5*H*)-one (**4a**) (DMSO-d_6, 600 MHz, 293 K)

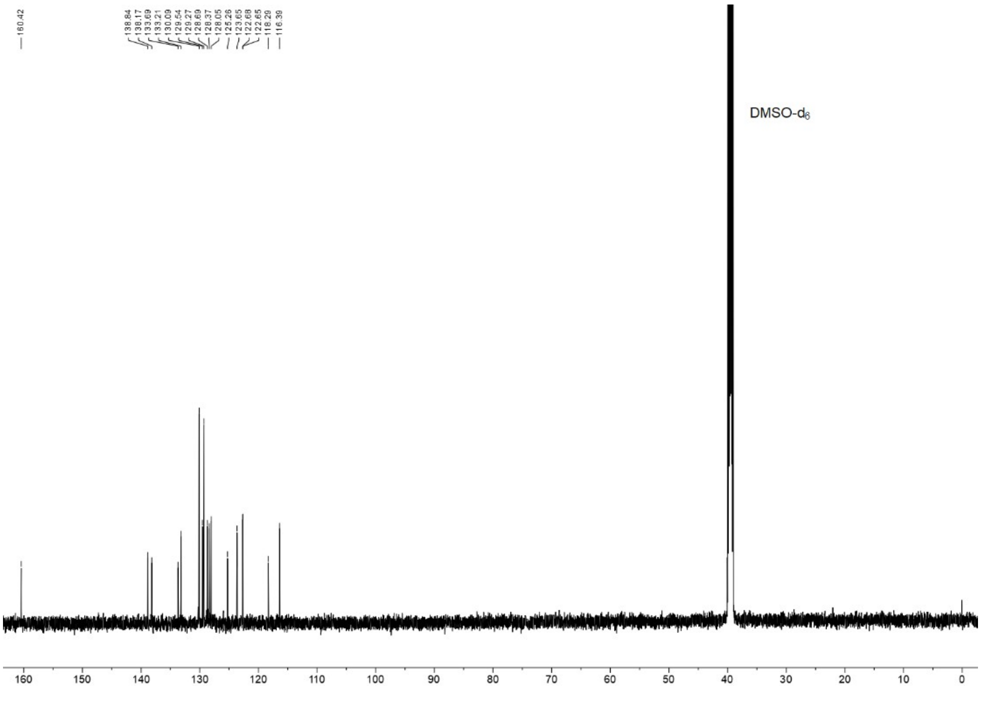

Figure S2. ^{13}C NMR spectrum of 5-Phenylphenanthridin-6(5*H*)-one (**4a**) (DMSO-d_6, 150 MHz, 293 K)

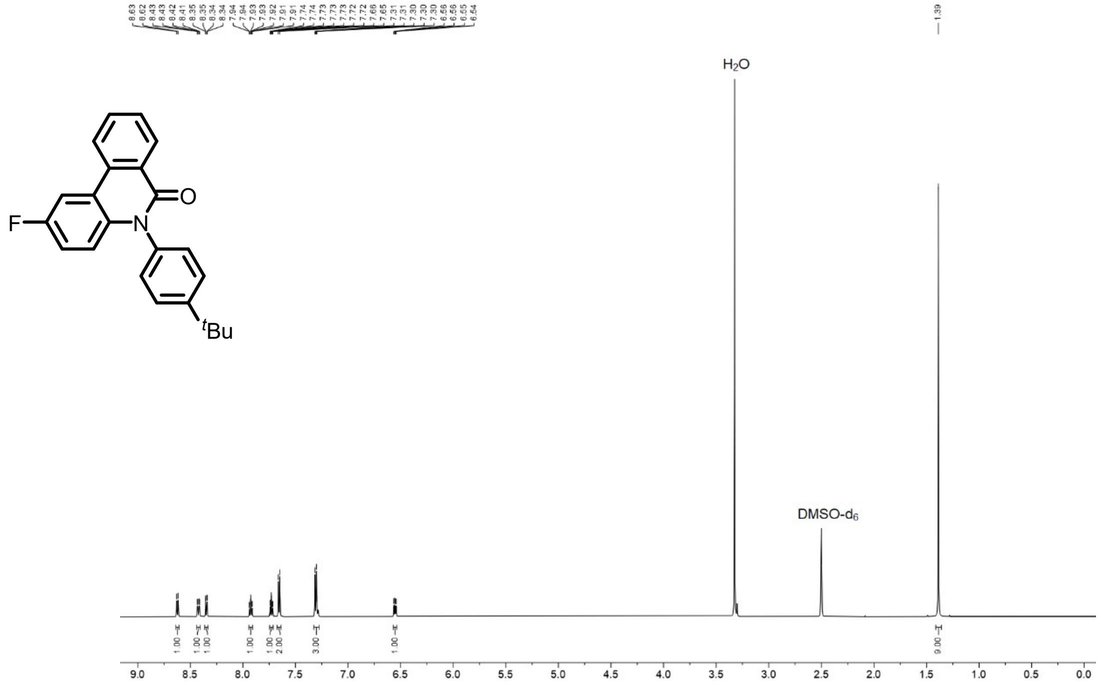

Figure S3. ^{1}H NMR spectrum of 5-(4-(*tert*-butyl)phenyl)-2-fluorophenanthridin-6(5*H*)-one (**4b**) (DMSO-d_6, 600 MHz, 293 K)

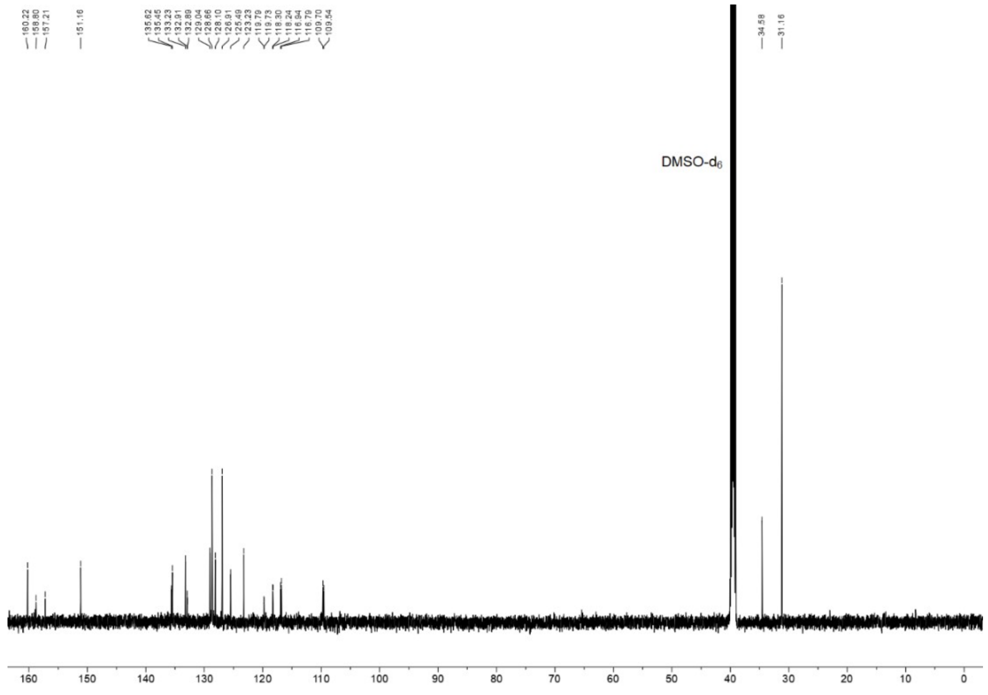

Figure S4. ^{13}C NMR spectrum of 5-(4-(*tert*-butyl)phenyl)-2-fluorophenanthridin-6(5*H*)-one (**4b**) (DMSO-d_6, 150 MHz, 293 K)

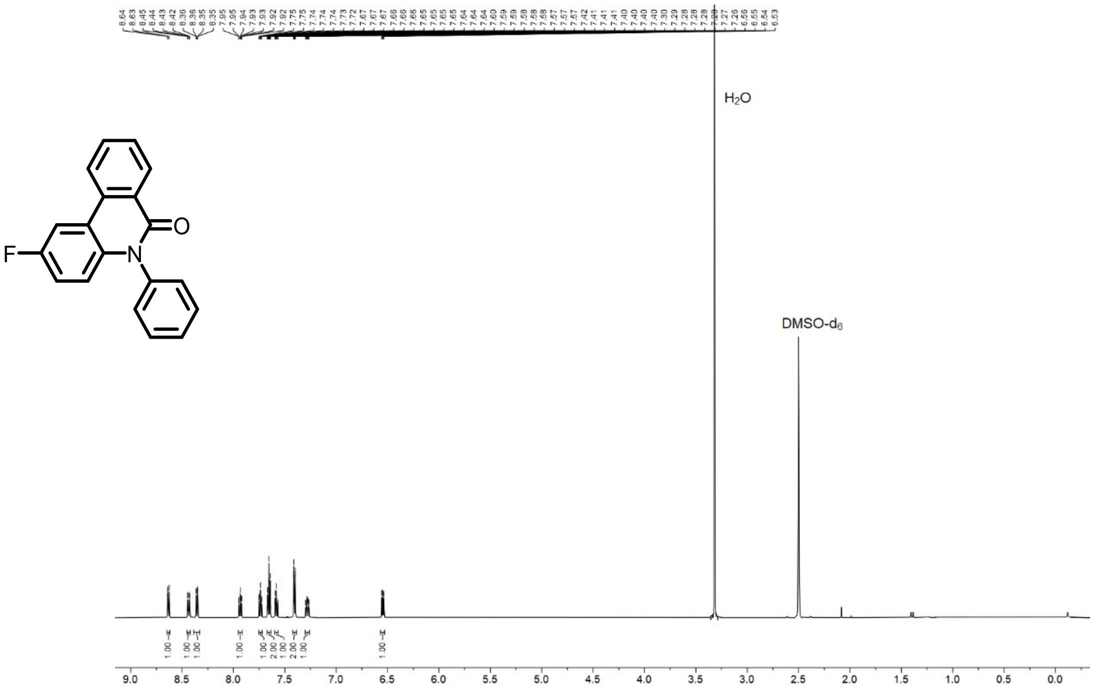

Figure S5. ^{1}H NMR spectrum of 2-fluoro-5-phenylphenanthridin-6(5*H*)-one (**4c**) (DMSO-d$_6$, 600 MHz, 293 K)

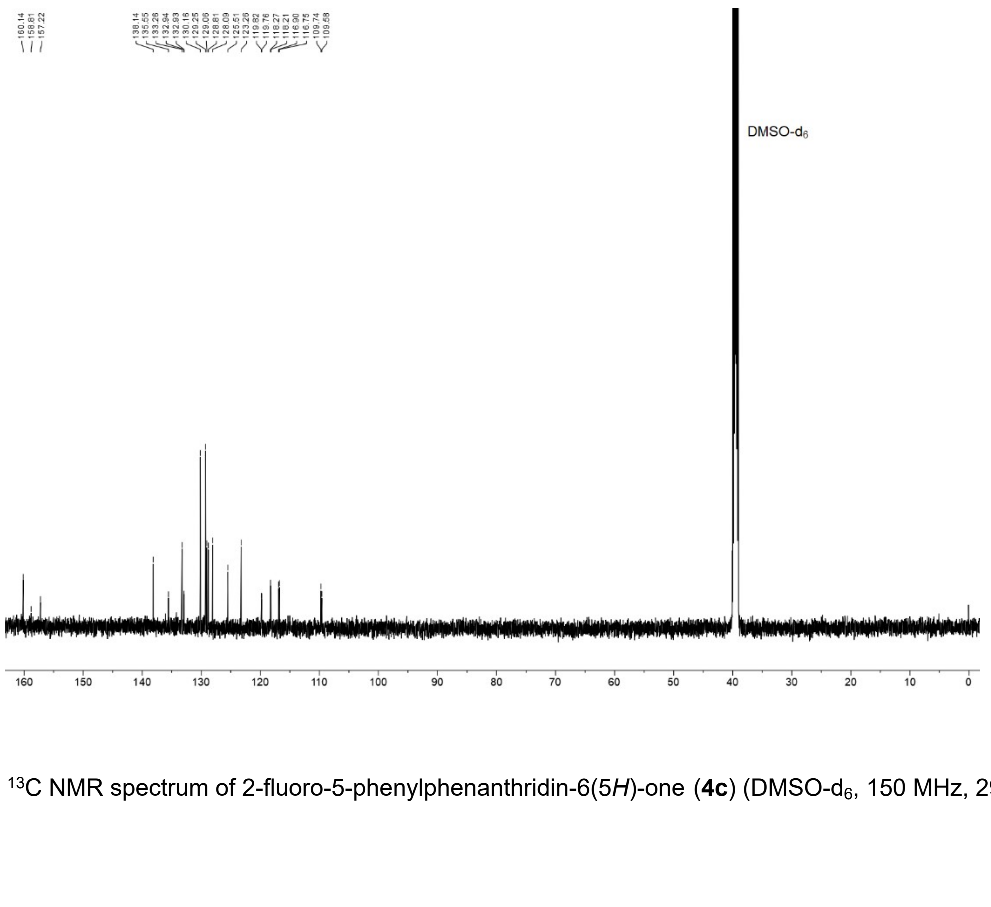

Figure S6. ^{13}C NMR spectrum of 2-fluoro-5-phenylphenanthridin-6(5*H*)-one (**4c**) (DMSO-d$_6$, 150 MHz, 293 K)

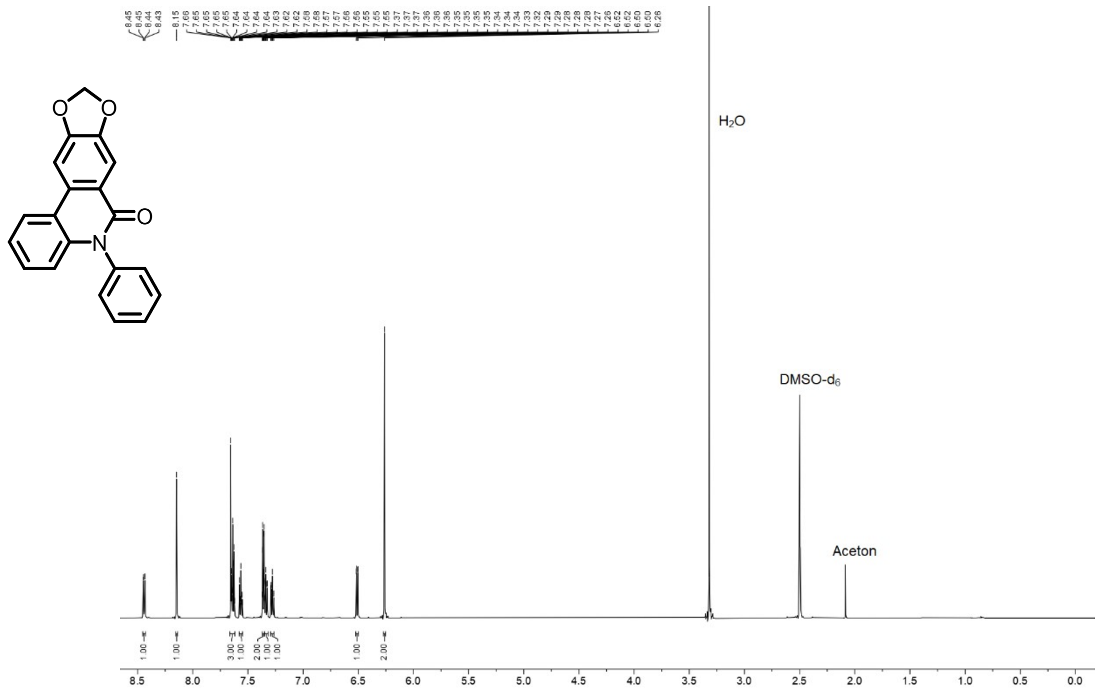

Figure S7. ^{1}H NMR spectrum of 5-phenyl-[1,3]dioxolo[4,5-j]phenanthridin-6(5*H*)-one (**5a**) (DMSO-d_6, 600 MHz, 293 K)

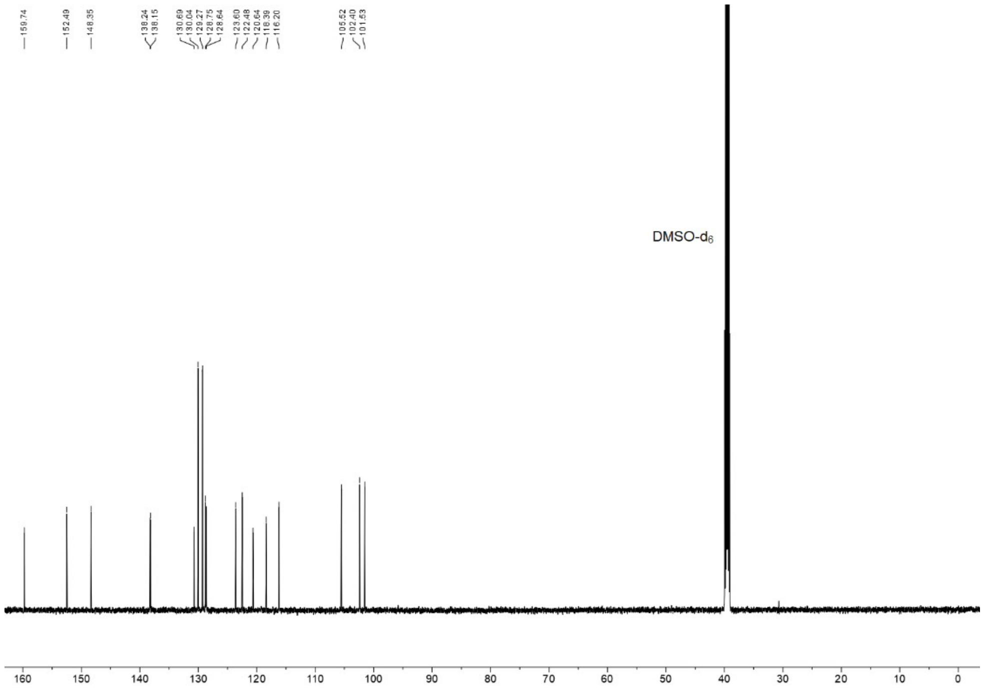

Figure S8. ^{13}C NMR spectrum of 5-phenyl-[1,3]dioxolo[4,5-j]phenanthridin-6(5*H*)-one (**5a**) (DMSO-d_6, 150 MHz, 293 K)

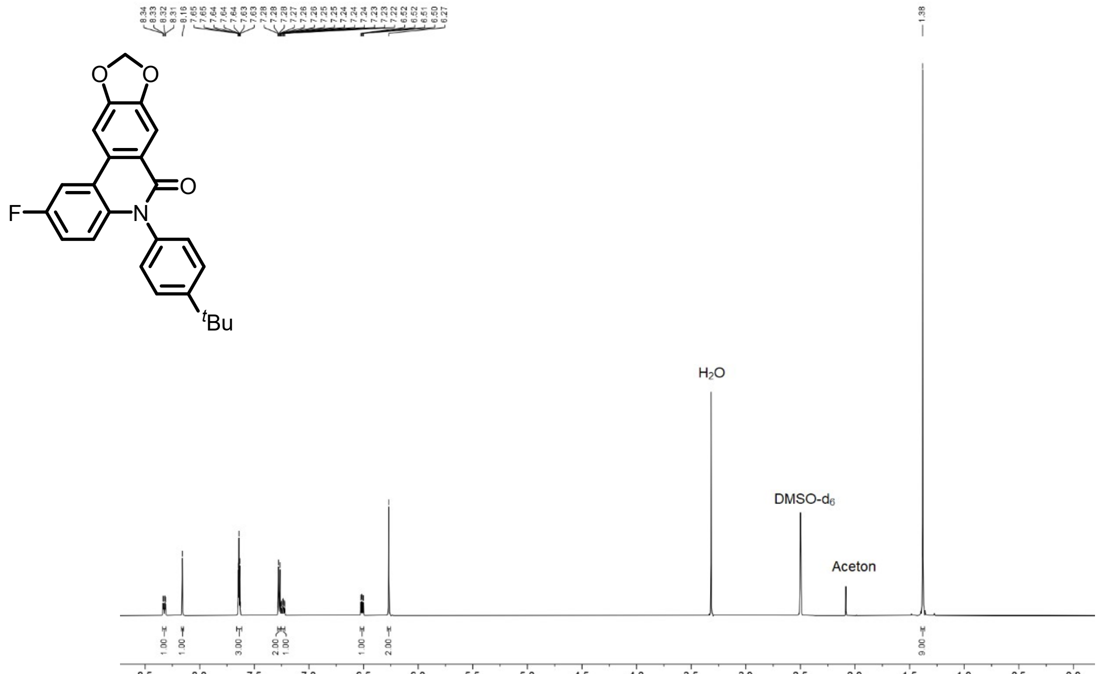

Figure S9. ^{1}H NMR spectrum of 5-(4-(*tert*-butyl)phenyl)-2-fluoro-[1,3]dioxolo[4,5-j]phenanthridin-6(5*H*)-one (**5b**) (DMSO-d_6, 600 MHz, 293 K)

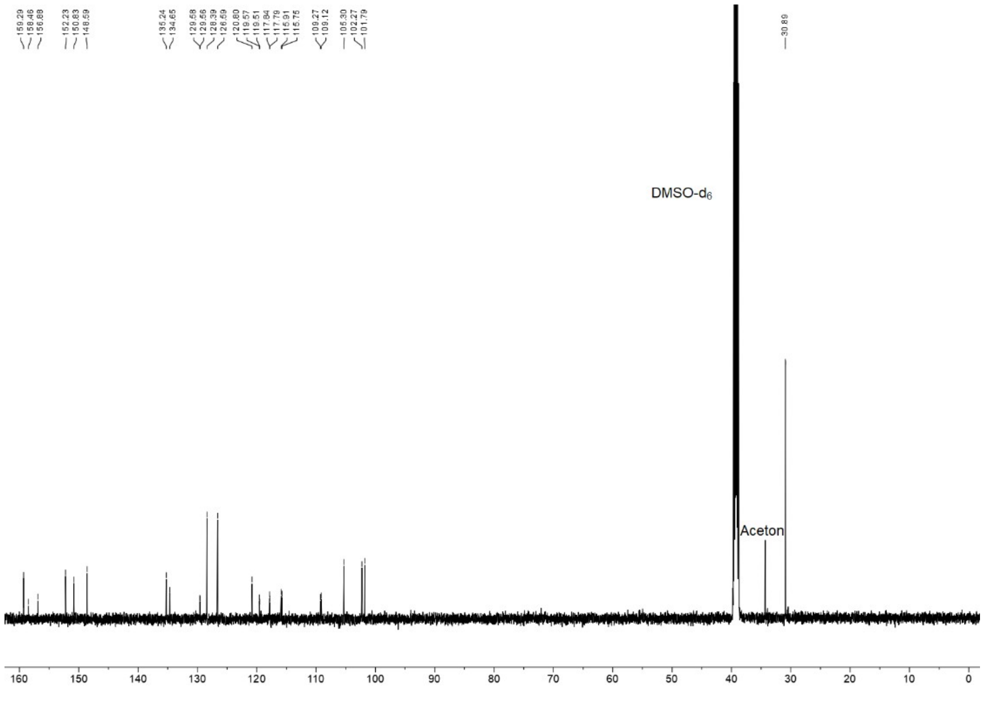

Figure S10. ^{13}C NMR spectrum of 5-(4-(*tert*-butyl)phenyl)-2-fluoro-[1,3]dioxolo[4,5-j]phenanthridin-6(5*H*)-one (**5b**) (DMSO-d_6, 150 MHz, 293 K)

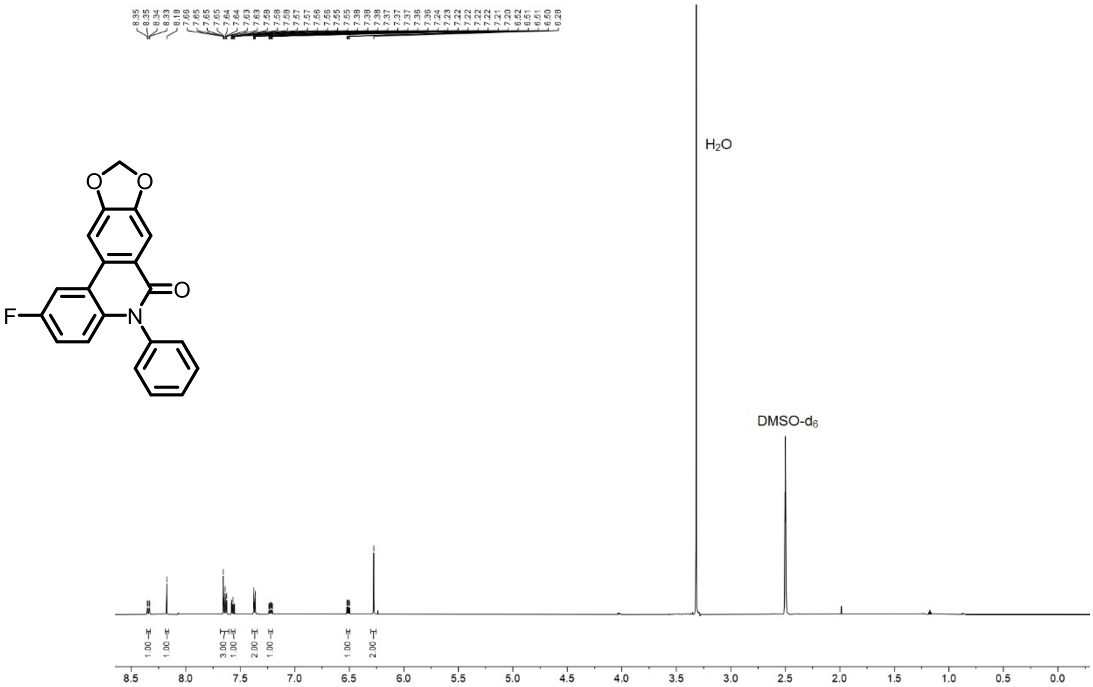

Figure S11. ^{1}H NMR spectrum of 2-fluoro-5-phenyl-[1,3]dioxolo[4,5-j]phenanthridin-6(5*H*)-one (**5c**) (DMSO-d$_6$, 600 MHz, 293 K)

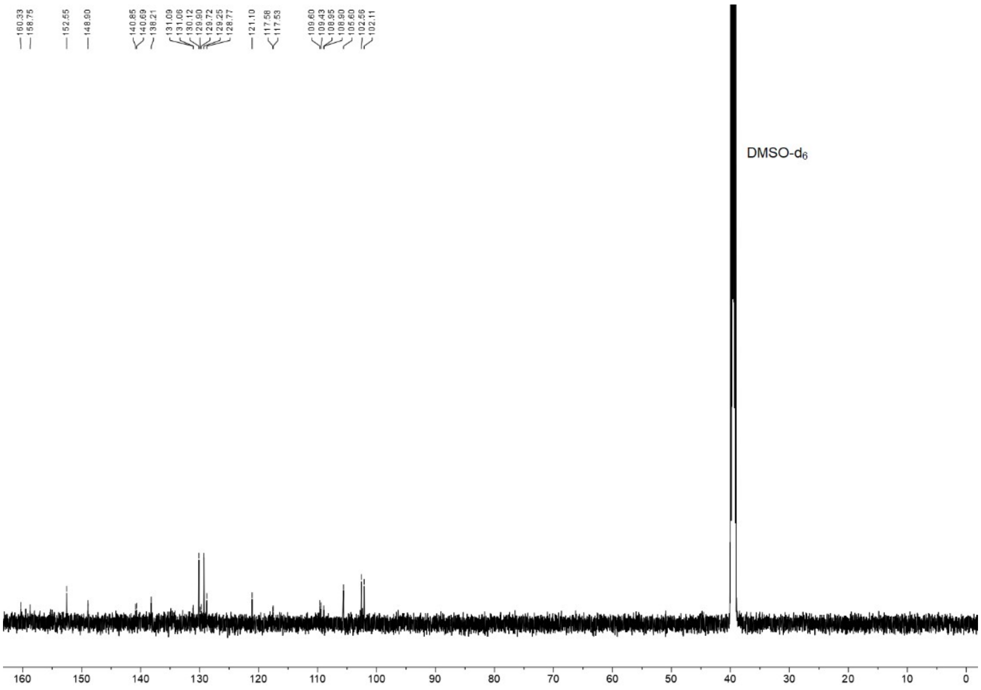

Figure S12. ^{13}C NMR spectrum of 2-fluoro-5-phenyl-[1,3]dioxolo[4,5-j]phenanthridin-6(5*H*)-one (**5c**) (DMSO-d$_6$, 150 MHz, 293 K)

9 ^{1}H and ^{13}C spectra of *N*-alkylphenanthridinones 6 and *N*-alkylcrinasiadines 7

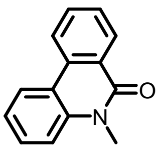

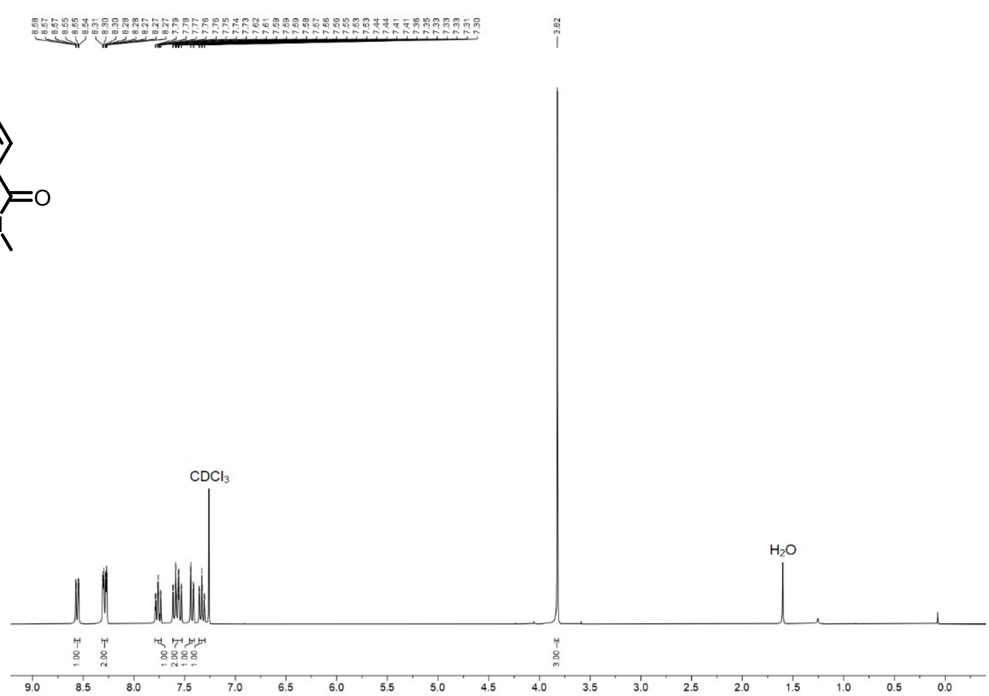

Figure S13. ^{1}H NMR spectrum of 5-methylphenanthridin-6(5*H*)-one (**6a**) ($CDCl_3$, 300 MHz, 293 K)

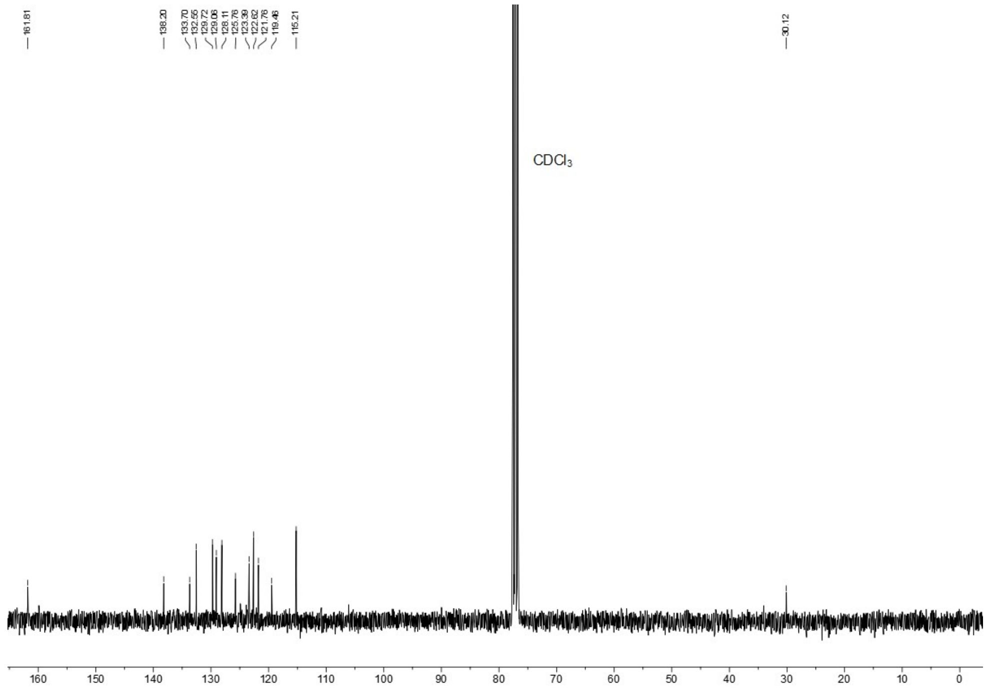

Figure S14. ^{13}C NMR spectrum of 5-methylphenanthridin-6(5*H*)-one (**6a**) ($CDCl_3$, 75 MHz, 293 K)

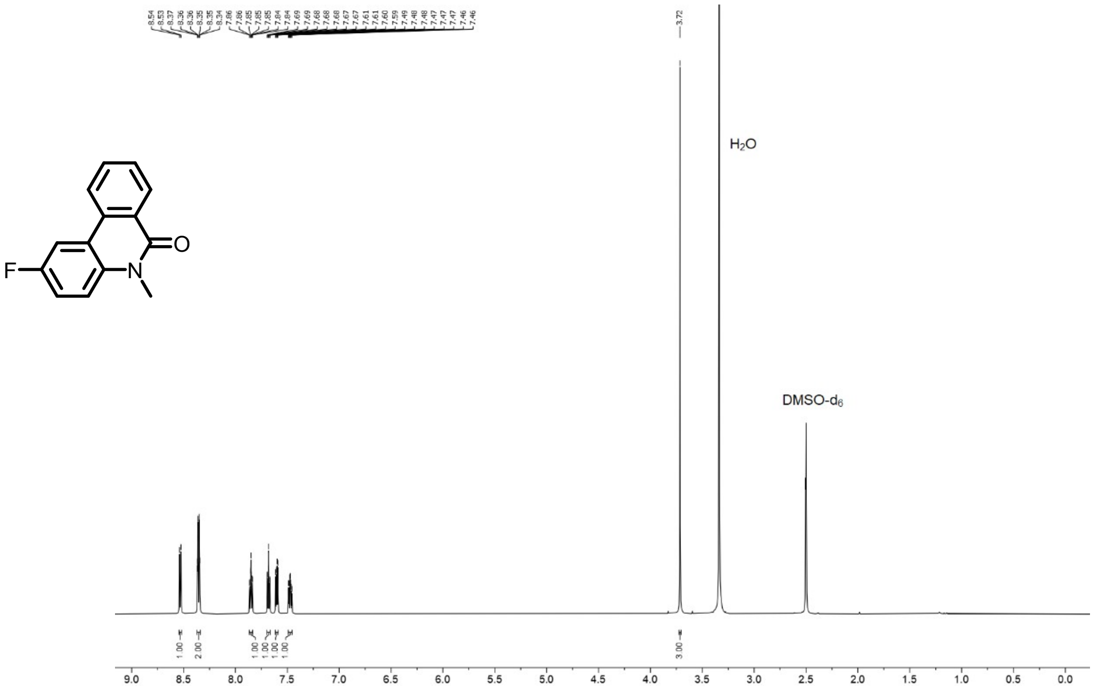

Figure S15. ^{1}H NMR spectrum of 2-fluoro-5-methylphenanthridin-6(5*H*)-one (**6b**) (DMSO-d_6, 600 MHz, 293 K)

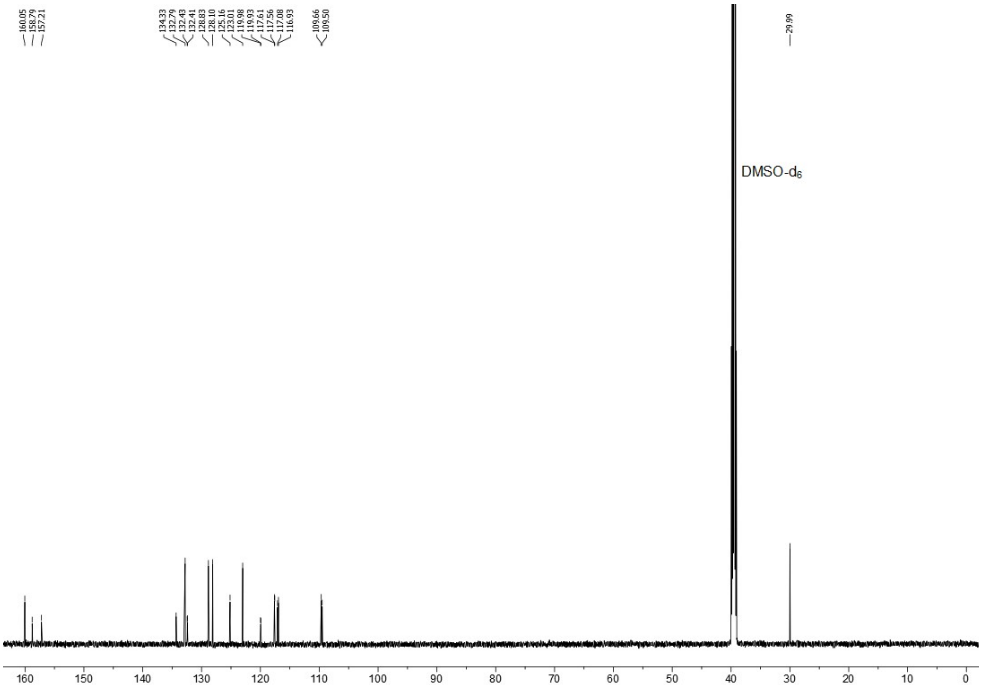

Figure S16. ^{13}C NMR spectrum of 2-fluoro-5-methylphenanthridin-6(5*H*)-one (**6b**) (DMSO-d_6, 150 MHz, 293 K)

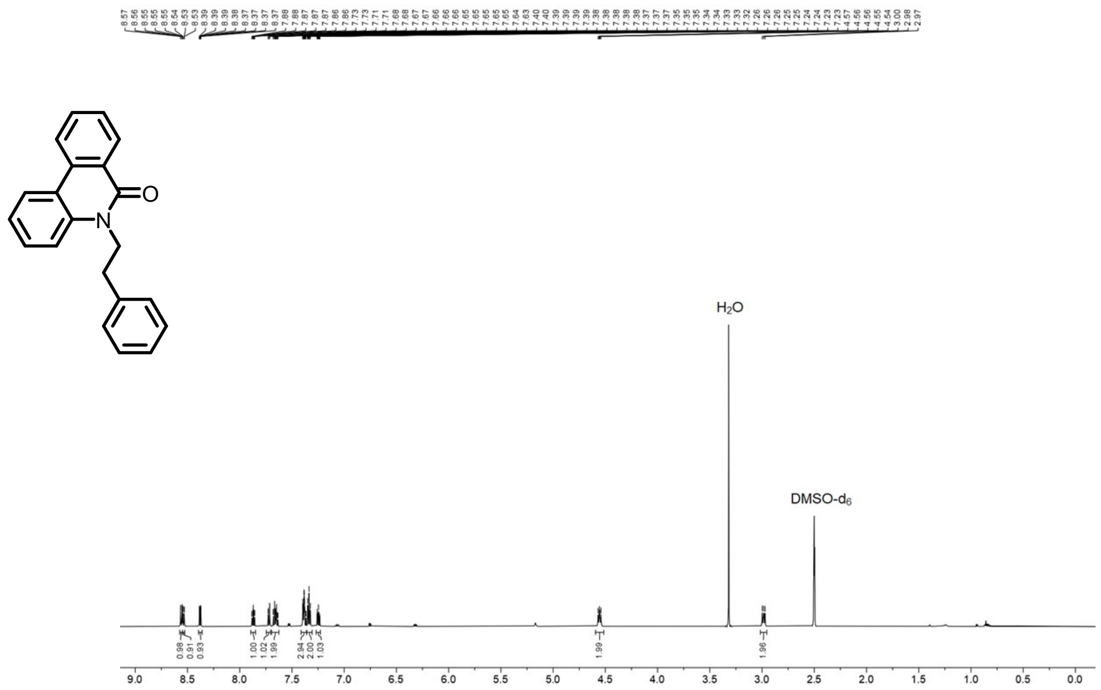

Figure S17. ^{1}H NMR spectrum of 5-phenethylphenanthridin-6(5*H*)-one (**6c**) (DMSO-d_6, 600 MHz, 293 K)

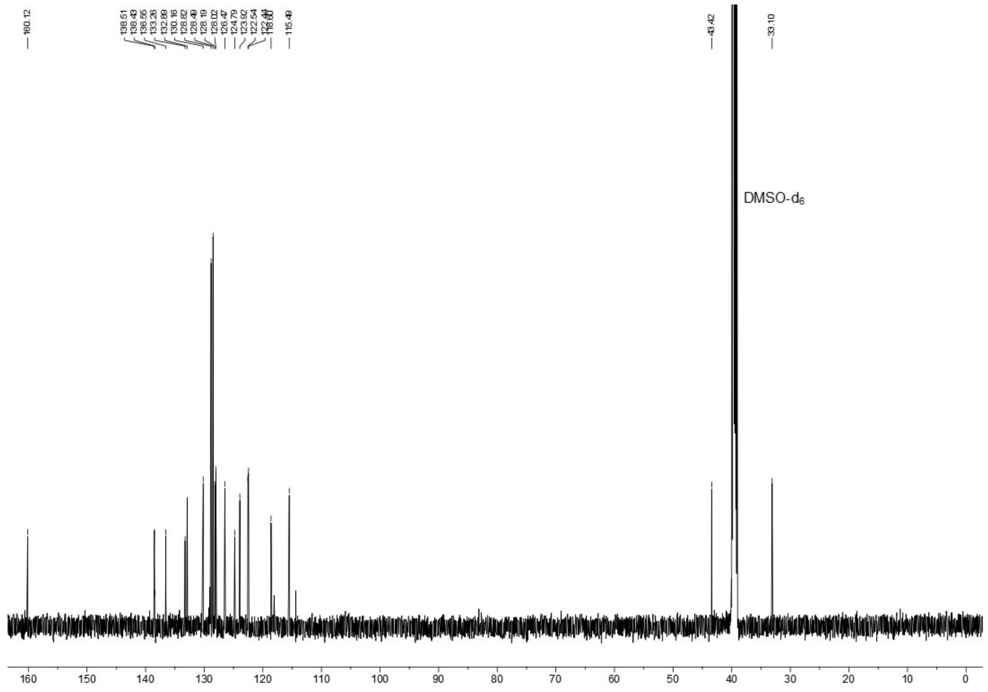

Figure S18. ^{13}C NMR spectrum of 5-phenethylphenanthridin-6(5*H*)-one l(**6c**) (DMSO-d_6, 150 MHz, 293 K)

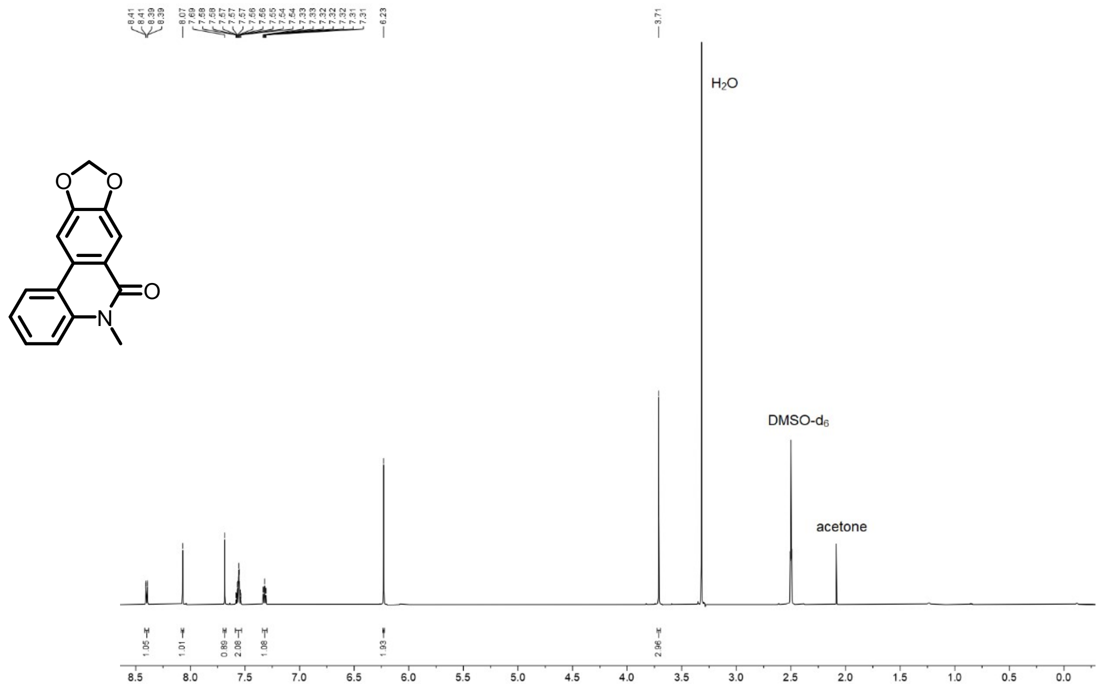

Figure S19. ^{1}H NMR spectrum of 5-methyl-[1,3]dioxolo[4,5-j]phenanthridin-6(5*H*)-one (**7a**) (DMSO-d_6, 600 MHz, 293 K)

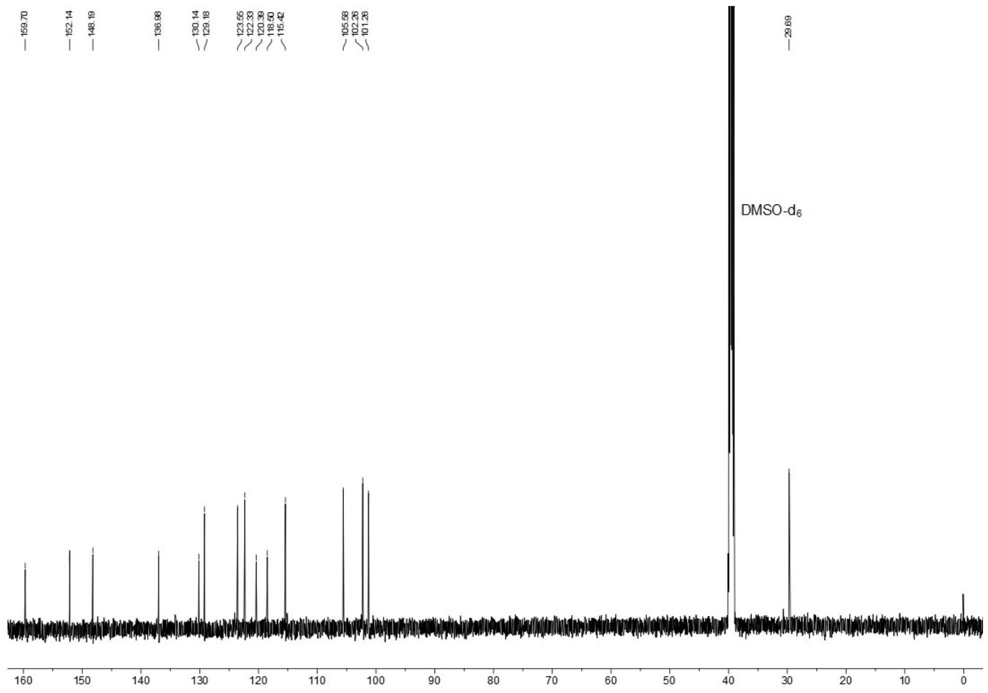

Figure S20. ^{13}C NMR spectrum of 5-methyl-[1,3]dioxolo[4,5-j]phenanthridin-6(5*H*)-one (**7a**) (DMSO-d_6, 150 MHz, 293 K)

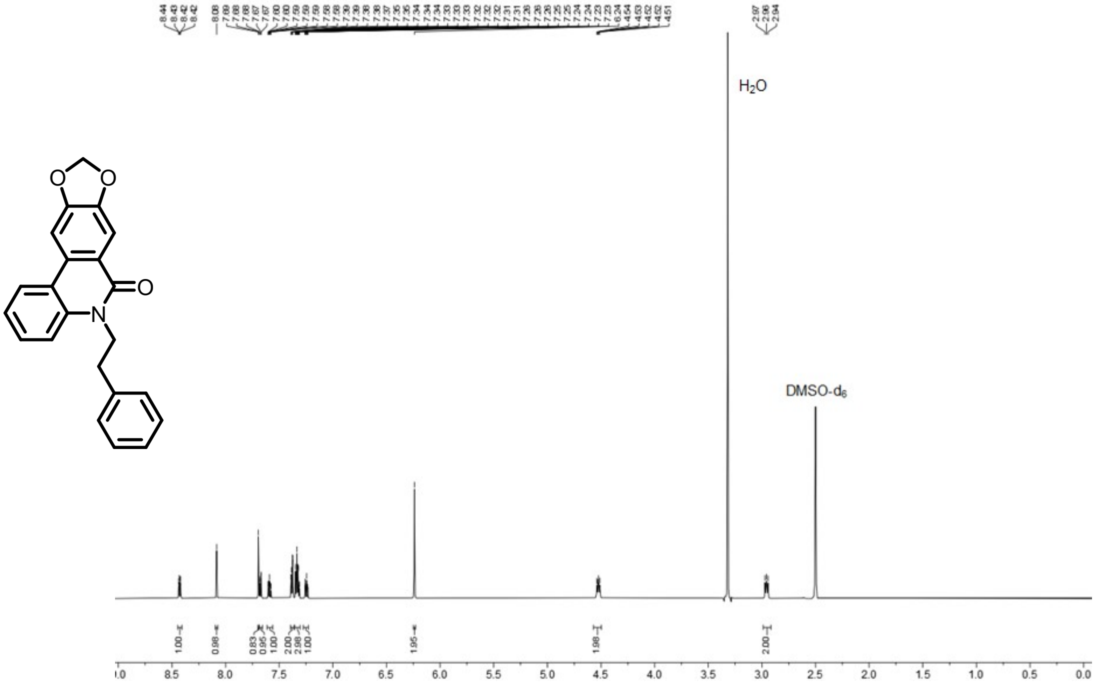

Figure S21. ^{1}H NMR spectrum of 5-phenethyl-[1,3]dioxolo[4,5-j]phenanthridin-6(5*H*)-one (**7b**) (DMSO-d_6, 600 MHz, 293 K)

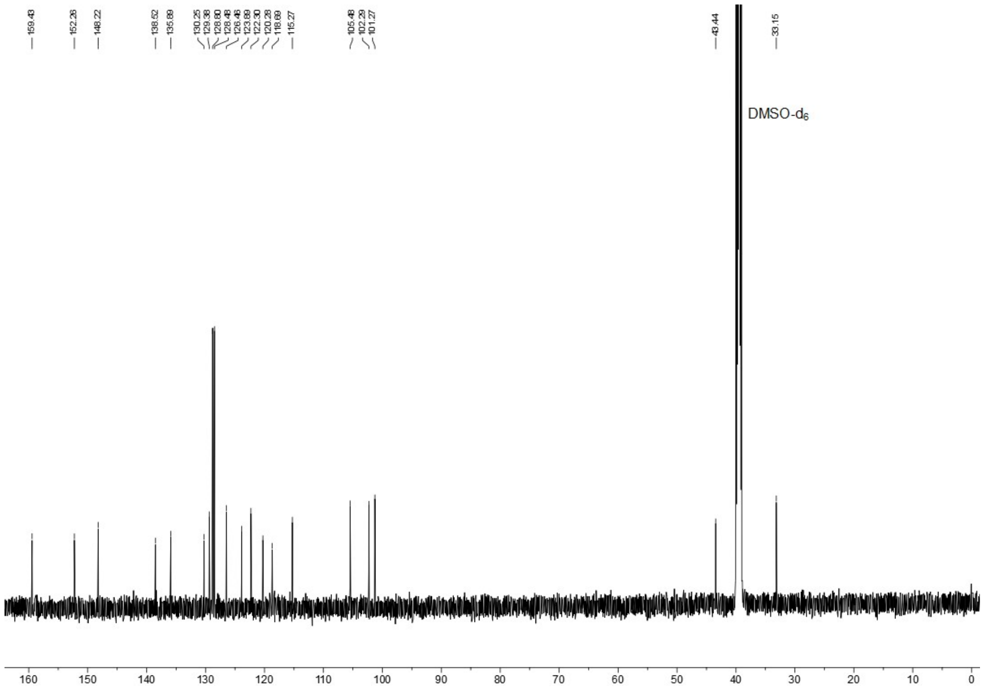

Figure S22. ^{13}C NMR spectrum of 5-phenethyl-[1,3]dioxolo[4,5-j]phenanthridin-6(5*H*)-one (**7b**) (DMSO-d_6, 150 MHz, 293 K)

10 High-Performance Liquid Chromatography (HPLC) Data of *N*-arylphenanthridinones 4 and *N*-arylcrinasiadines 5

10.1 HPLC Data of 5-(4-(*tert*-Butyl)phenyl)-2-fluorophenanthridin-6(5*H*)-one (4b)

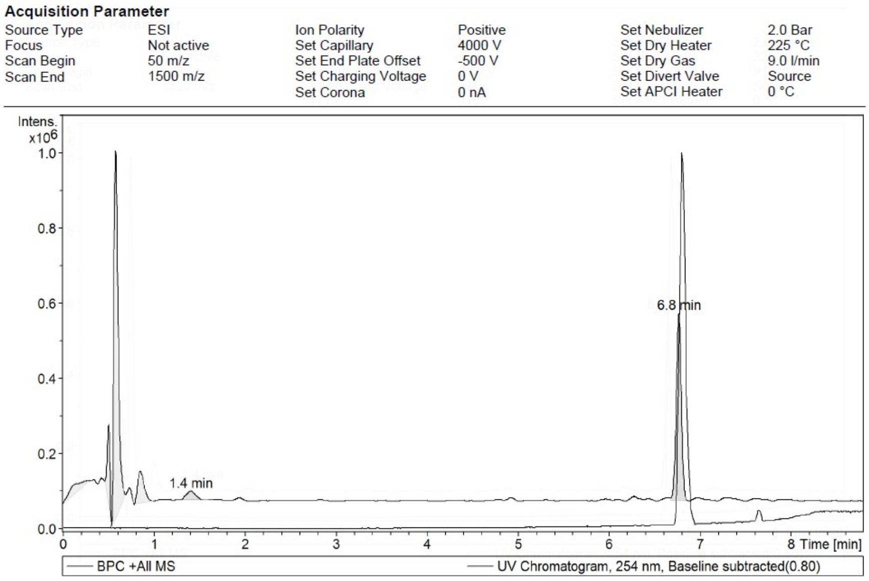

10.2 HPLC Data of 2-Fluoro-5-phenylphenanthridin-6(5*H*)-one (4c)

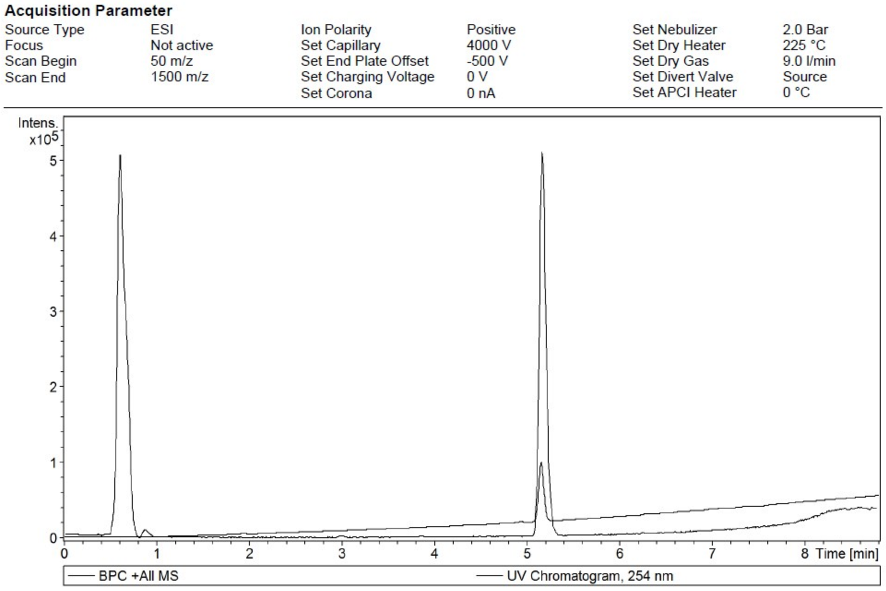

10.3 HPLC Data of 5-(4-(*tert*-Butyl)phenyl)-2-fluoro-[1,3]dioxolo[4,5-j]phenanthridin-6(5*H*)-one (5b)

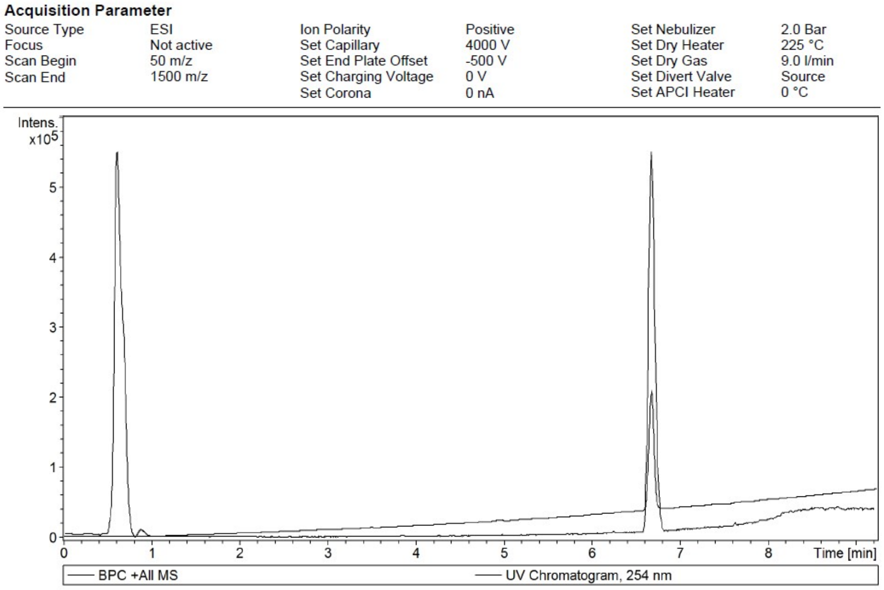

10.4 HPLC Data of 2-Fluoro-5-Phenyl-[1,3]dioxolo[4,5-j]phenanthridin-6(5*H*)-one (5c)

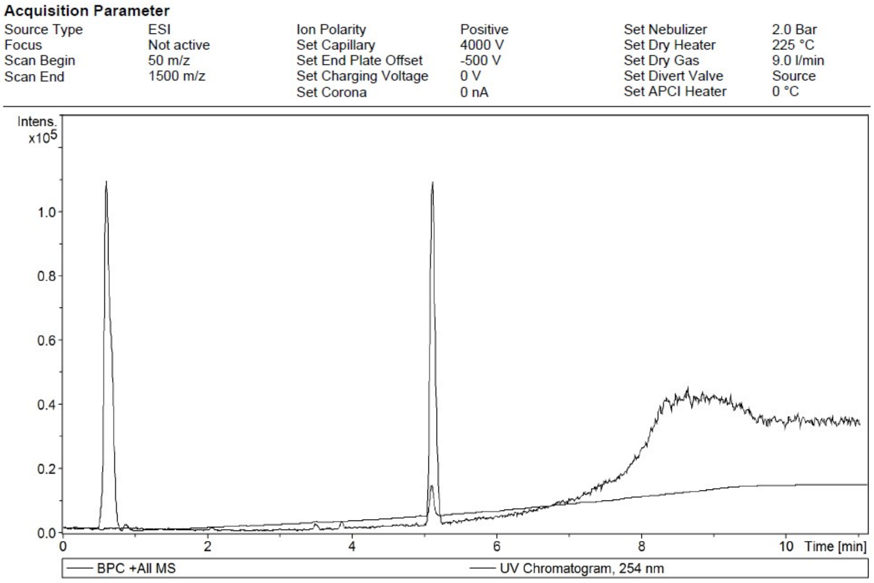

11 Absorption and emission spectra

11.1 Absorption and emission spectra of 6(5*H*)-phenanthridinone (8)

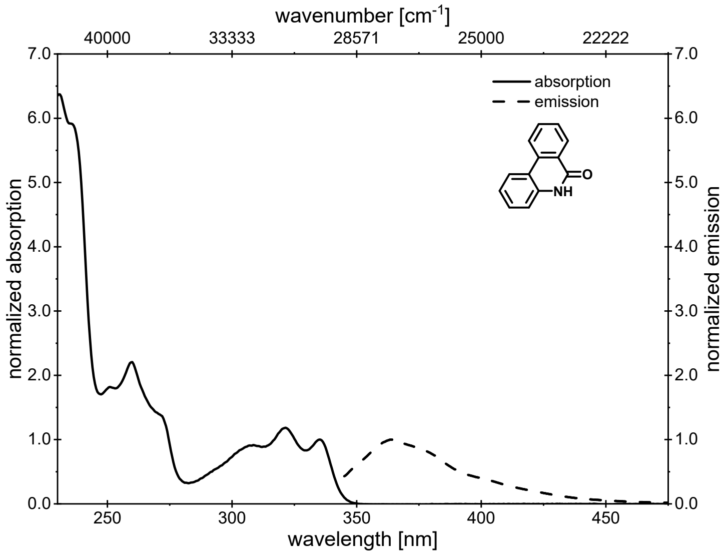

Recorded in dichloromethane, T = 293 K, c_{abs}(**8**) = 10^{-5} M, c_{em}(**8**) = 10^{-7} M, $\lambda_{ex} = \lambda_{max,\ abs}$.

11.2 Absorption and emission spectra of *N*-arylphenanthridinones 4 and *N*-arylcrinasiadines 5

Absorption and emission spectra of 5-phenylphenanthridin-6(5*H*)-one (4a)

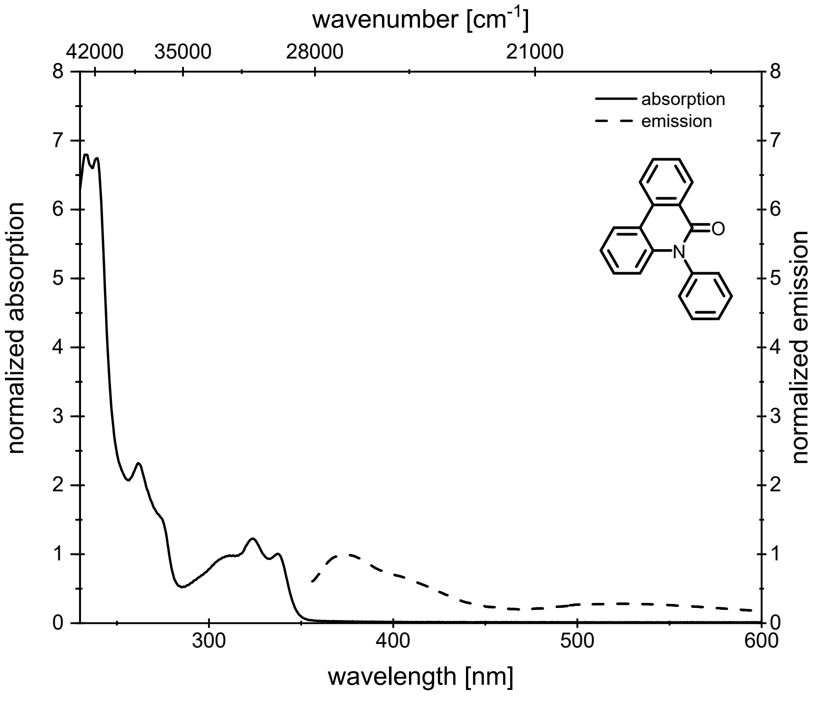

Recorded in dichloromethane, T = 293 K, c_{abs}(**4a**) = 10^{-5} M, c_{em}(**4a**) = 10^{-7} M, $\lambda_{ex} = \lambda_{max,\ abs}$.

Absorption and emission spectra of 5-(4-(*tert*-butyl)phenyl)-2-fluorophenanthridin-6(5*H*)-one (4b)

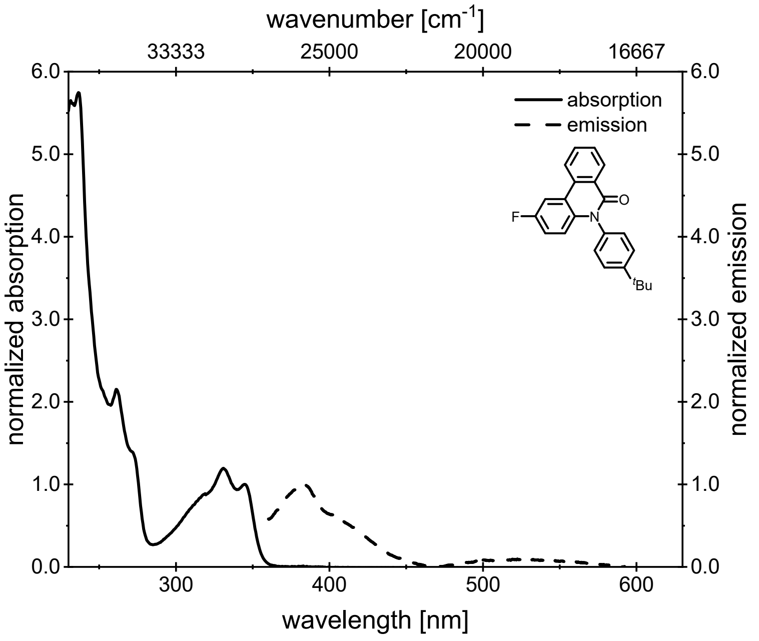

Recorded in dichloromethane, T = 293 K, c_{abs}(**4b**) = 10^{-5} M, c_{em}(**4b**) = 10^{-7} M, λ_{ex} = $\lambda_{max,\ abs}$.

Absorption and emission spectra of 2-fluoro-5-phenylphenanthridin-6(5*H*)-one (4c)

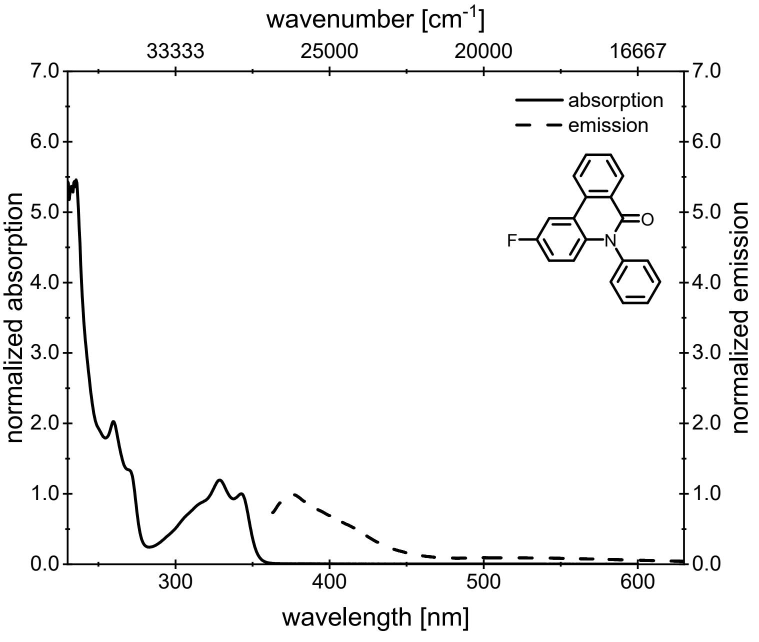

Recorded in dichloromethane, T = 293 K, c_{abs}(**4c**) = 10^{-5} M, c_{em}(**4c**) = 10^{-7} M, λ_{ex} = $\lambda_{max,\ abs}$.

Absorption and emission spectra of 5-phenyl-[1,3]dioxolo[4,5-j]phenanthridin-6(5*H*)-one (5a)

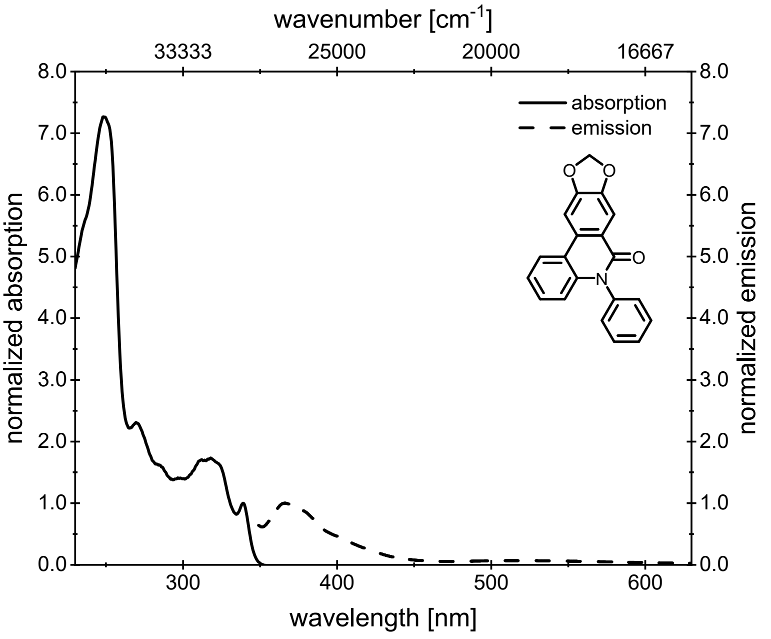

Recorded in dichloromethane, T = 293 K, c_{abs}(**5a**) = 10^{-5} M, c_{em}(**5a**) = 10^{-7} M, λ_{ex} = $\lambda_{max,\ abs}$.

Absorption and emission spectra of 5-(4-(*tert*-butyl)phenyl)-2-fluoro-[1,3]dioxolo[4,5-j]phenanthridin-6(5*H*)-one (5b)

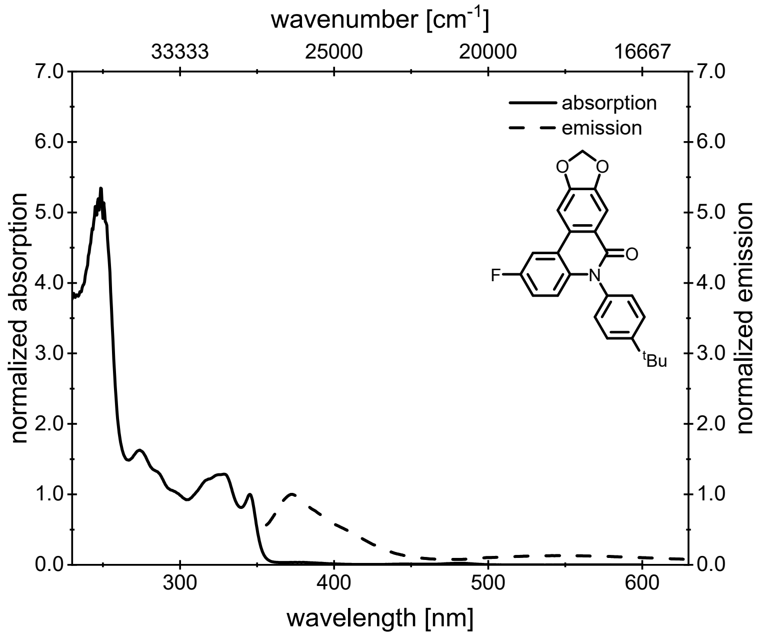

Recorded in dichloromethane, T = 293 K, c_{abs}(**5b**) = 10^{-5} M, c_{em}(**5b**) = 10^{-7} M, λ_{ex} = $\lambda_{max,\ abs}$.

Absorption and emission spectra of 5-(4-(*tert*-butyl)phenyl)-2-fluoro-[1,3]dioxolo[4,5-j]phenanthridin-6(5*H*)-one (5b)

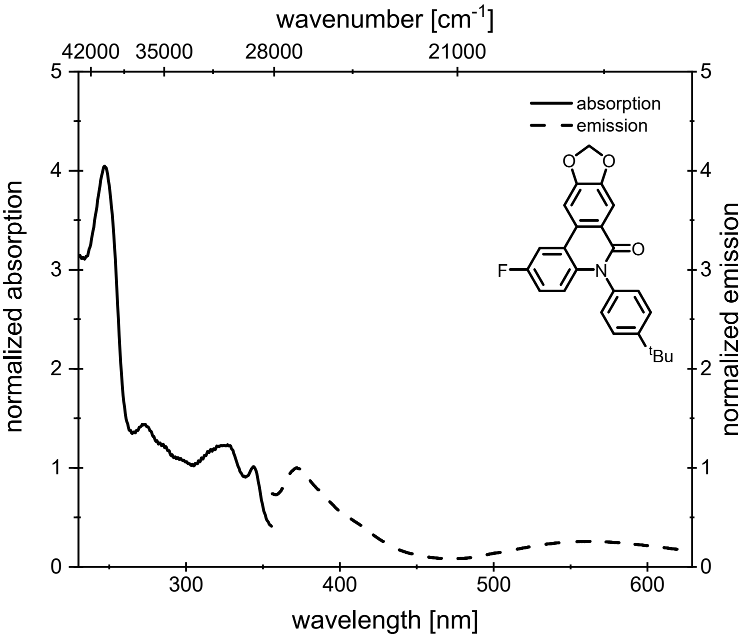

Recorded in acetonitrile, T = 293 K, c_{abs}(**5b**) = 10^{-5} M, c_{em}(**5b**) = 10^{-7} M, $\lambda_{ex} = \lambda_{max,\ abs}$.

Absorption and emission spectra of 2-fluoro-5-phenyl-[1,3]dioxolo[4,5-j]phenanthridin-6(5*H*)-one (5c)

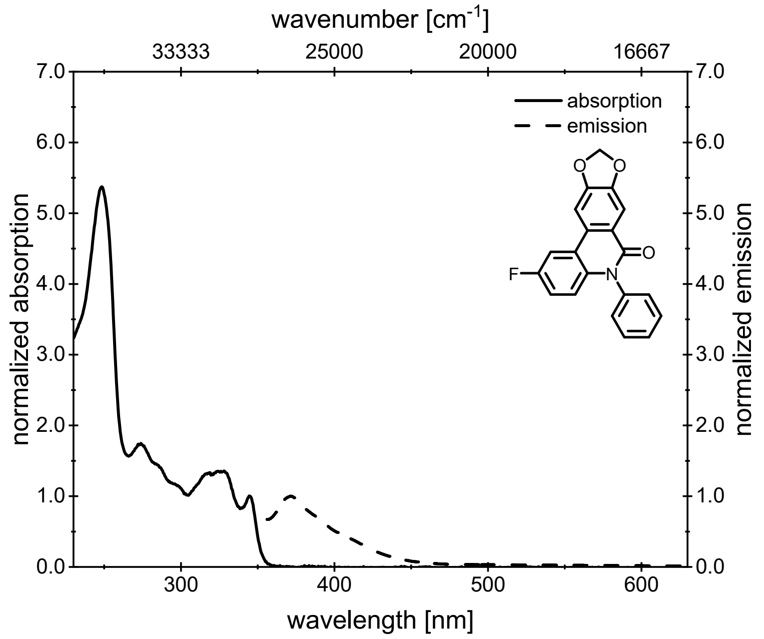

Recorded in dichloromethane, T = 293 K, c_{abs}(**5c**) = 10^{-5} M, c_{em}(**5c**) = 10^{-7} M, $\lambda_{ex} = \lambda_{max,\ abs}$.

11.3 Absorption and emission spectra of *N*-alkylphenanthridinones 6 and *N*-alkylcrinasiadines 7

Absorption and emission spectra of 5-methylphenanthridin-6(5*H*)-one (6a)

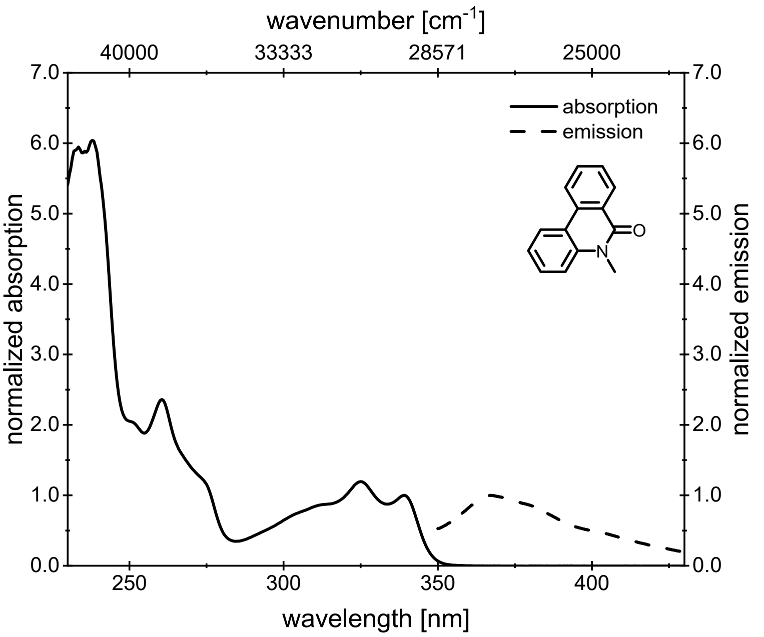

Recorded in dichloromethane, T = 293 K, c_{abs}(**6a**) = 10^{-5} M, c_{em}(**6a**) = 10^{-7} M, $\lambda_{ex} = \lambda_{max,\ abs}$.

Absorption and emission spectra of 2-fluoro-5-methylphenanthridin-6(5*H*)-one (6b)

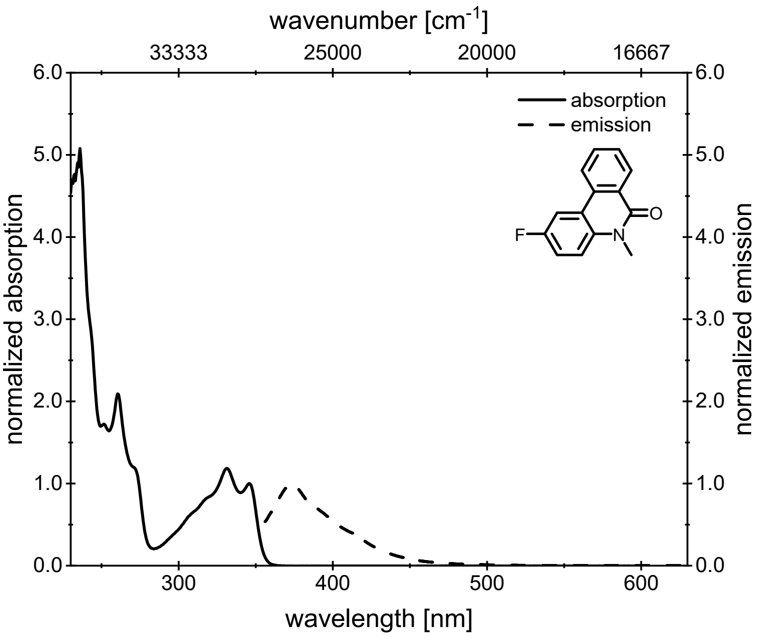

Recorded in dichloromethane, T = 293 K, c_{abs}(**6b**) = 10^{-5} M, c_{em}(**6b**) = 10^{-7} M, $\lambda_{ex} = \lambda_{max,\ abs}$.

Absorption and emission spectra of 5-phenethylphenanthridin-6(5*H*)-one (6c)

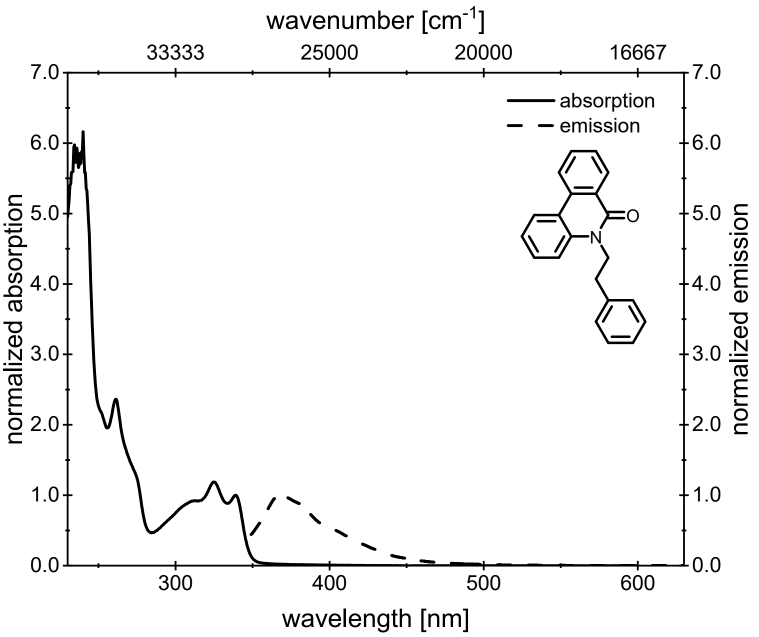

Recorded in dichloromethane, T = 293 K, c_{abs}(**6c**) = 10−5 M, c_{em}(**6c**) = 10−7 M, $\lambda_{ex} = \lambda_{max,\ abs}$.

Absorption and emission spectra of 5-methyl-[1,3]dioxolo[4,5-j]phenanthridin-6(5*H*)-one (7a)

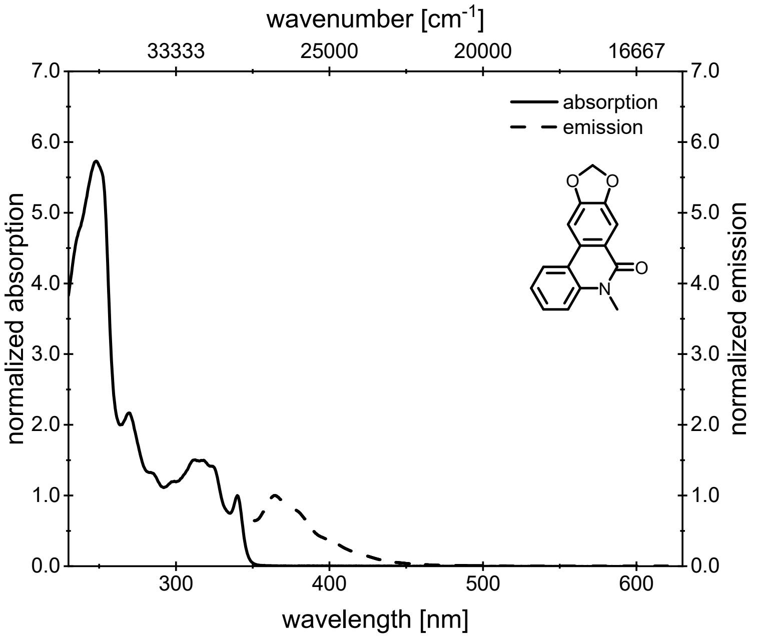

Recorded in dichloromethane, T = 293 K, c_{abs}(**7a**) = 10−5 M, c_{em}(**7a**) = 10−7 M, $\lambda_{ex} = \lambda_{max,\ abs}$.

Absorption and emission spectra of 5-methyl-[1,3]dioxolo[4,5-j]phenanthridin-6(5*H*)-one (7b)

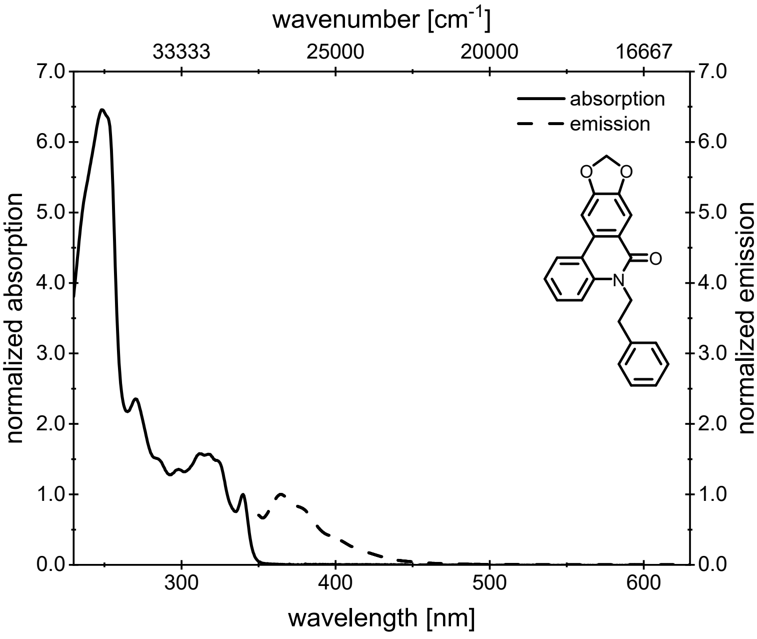

Recorded in dichloromethane, T = 293 K, c_{abs}(**7b**) = 10^{-5} M, c_{em}(**7b**) = 10^{-7} M, λ_{ex} = $\lambda_{max,\ abs}$.

11.4 Degassed and non-degassed emission spectra of *N*-arylphenanthridinone 4b and *N*-arylcrinasiadine 5b

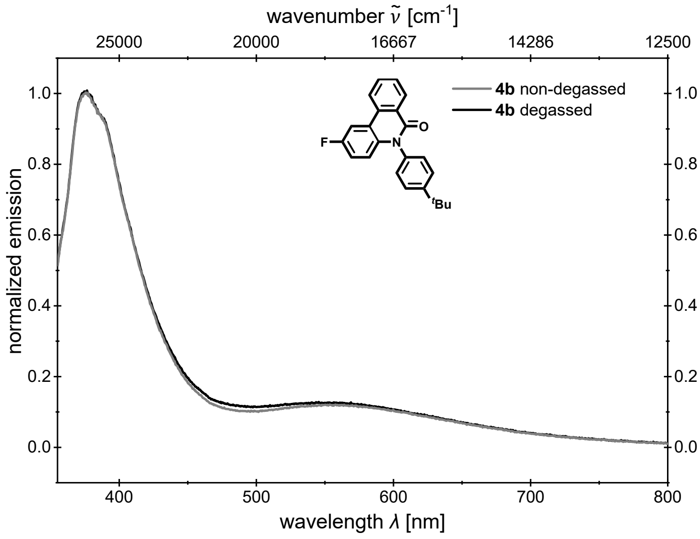

Recorded in acetonitrile, T = 298 K, c_{em}(**4b**) = 10^{-7} M, λ_{ex} = $\lambda_{max,\ abs}$.

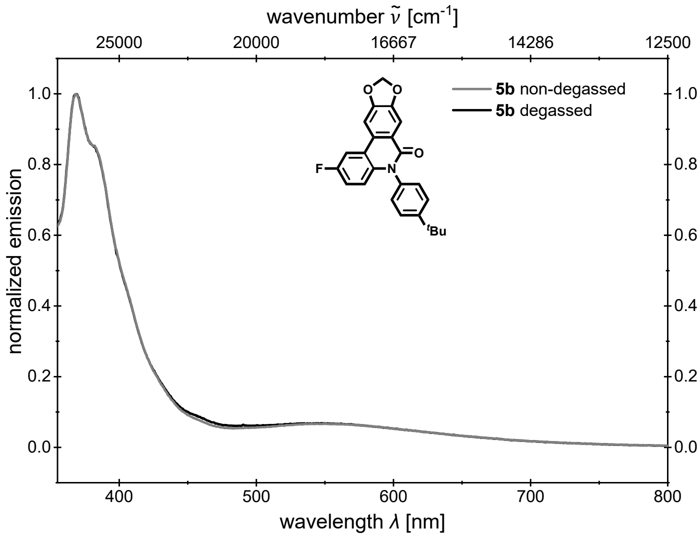

Recorded in acetonitrile, T = 298 K, c_{em}(**5b**) = 10^{-7} M, $\lambda_{ex} = \lambda_{max,\ abs}$.

11.5 Jacobian[10] energy-corrected emission spectra and corresponding integrated emission intensities of *N*-arylphenanthridinone 4b and *N*-arylcrinasiadine 5b

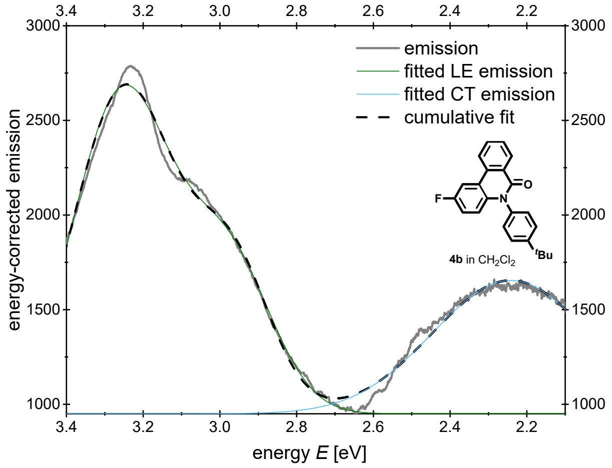

Jacobian energy-corrected simulated emission spectra of *N*-arylphenanthridinone **4b** in dichloromethane, generated from superimposed Gaussian functions, compared to the corresponding Jacobian energy-corrected experimental emission spectra (recorded in CH_2Cl_2, T = 293 K, c = 10^{-5} M, gray solid line).

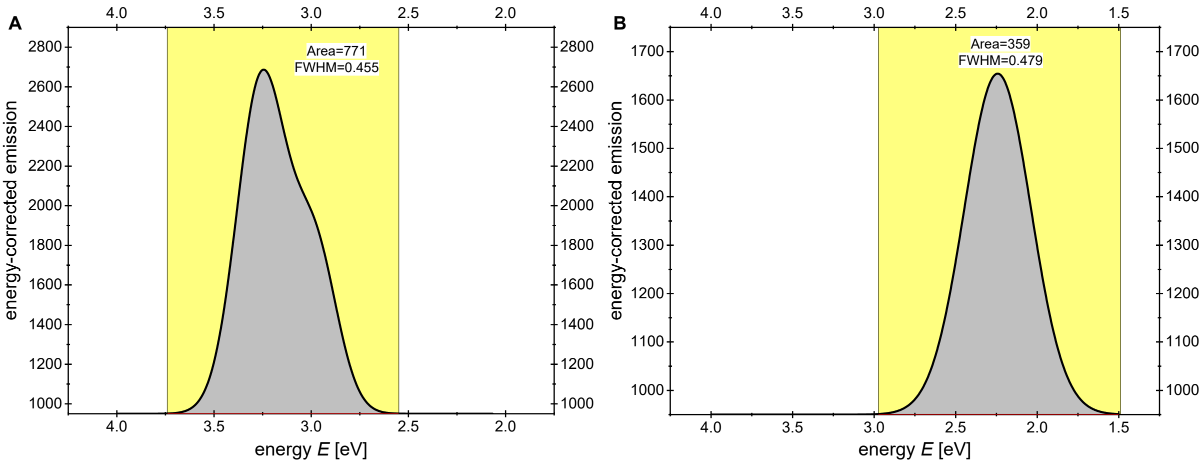

Integrated emission intensity of the fitted LE emission (**A**) and the fitted CT emission (**B**) of *N*-arylphenanthridinone **4b**.

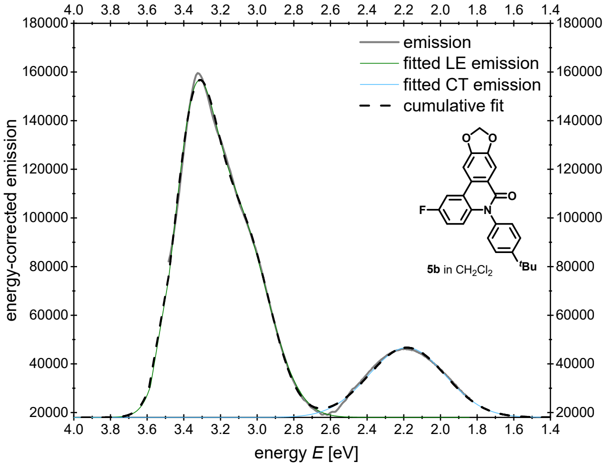

Jacobian energy-corrected simulated emission spectra of *N*-arylcrinasiadine **5b** in dichloromethane, generated from superimposed Gaussian functions, compared to the corresponding Jacobian energy-corrected experimental emission spectra (recorded in CH_2Cl_2, T = 293 K, c = 10^{-5} M, gray solid line).

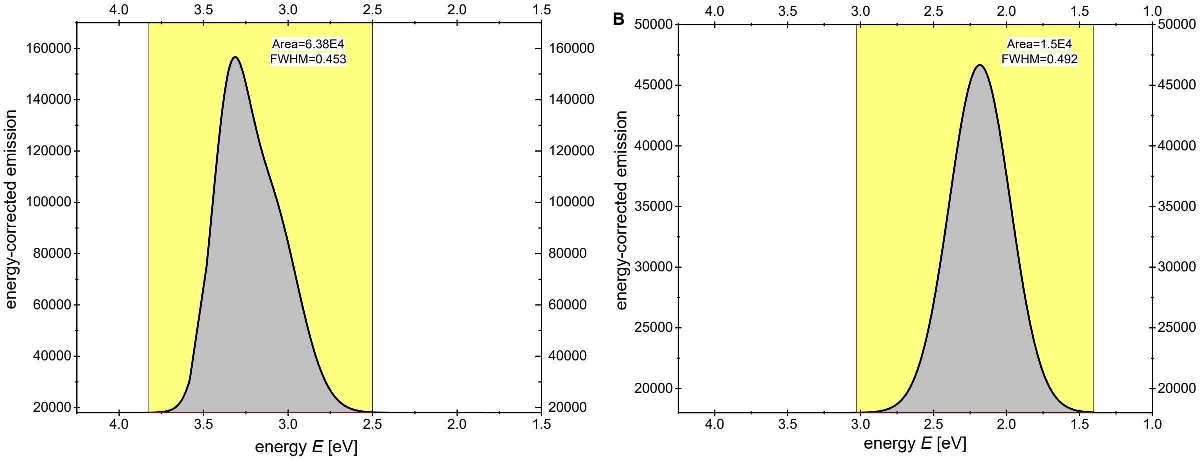

Integrated emission intensity of the fitted LE emission (**A**) and the fitted CT emission (**B**) of *N*-arylcrinasiadine **5b**.

12 Data of quantum chemical calculations

12.1 Comparison of the potential scans of 5-(4-(*tert*-butyl)phenyl)-2-fluorophenanthridin-6(5*H*)-one (4b) and 5-(4-(*tert*-Butyl)phenyl)-2-fluoro-[1,3]dioxolo[4,5-j]phenanthridin-6(5*H*)-one (5b)

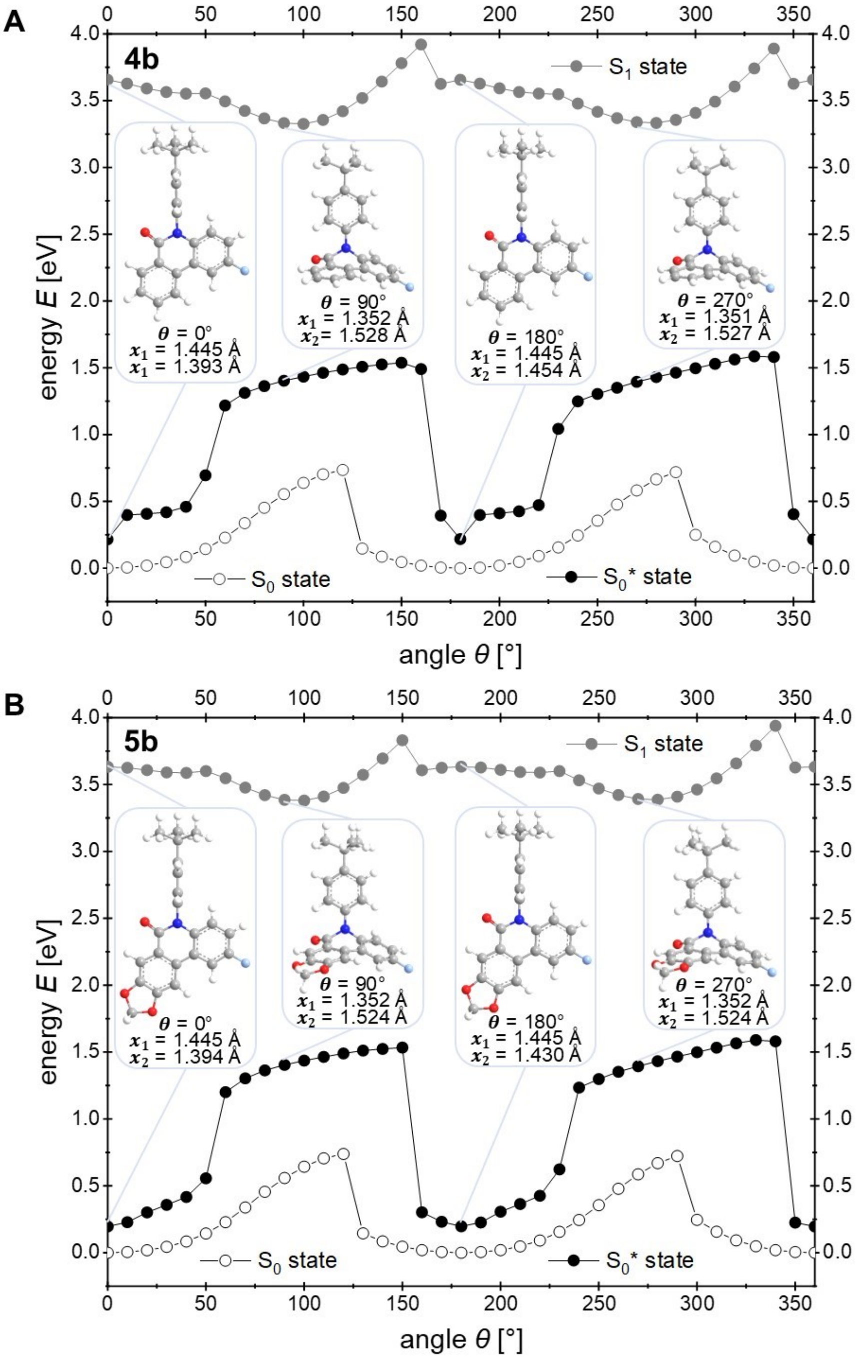

Potential energy scan of rotation of the *N*-aryl substituent along the torsional angle θ, starting from the geometry of compounds **4b** (**A**) and **5b** (**B**) at $\theta = 0°$, in the S_0 (open dots), S_0* (filled dots), and S_1 state (grey dots) in dichloromethane. Shown are the respective geometries after 90° rotation and x_1, the *N*-aryl bond length and x_2, the *N*-*CO* amide bond length (Gaussian 16, B3LYP/6-31G*, PCM CH_2Cl_2).

12.2 Quantum chemical calculation data of 5-(4-(*tert*-butyl)phenyl)-2-fluorophenanthridin-6(5*H*)-one (4b)

12.2.1 Computed xyz-coordinates of compound 4b (B3LYP/6-31G* PCM CH_2Cl_2)

C	-2.746525	3.18432	-0.002734
C	-1.356662	3.256873	0.003212
C	-0.628506	2.075155	0.007489
C	-1.276129	0.825937	0.005774
C	-2.692608	0.76706	-0.000664
C	-3.413465	1.976299	-0.004729
N	-0.519074	-0.362816	0.010394
C	-1.080226	-1.638008	0.007529
C	-2.55563	-1.704276	0.000985
C	-3.352843	-0.53928	-0.002968
C	-3.14871	-2.977518	-0.001206
C	-4.528507	-3.110176	-0.007185
C	-5.330818	-1.959202	-0.011158
C	-4.754266	-0.696784	-0.009117
O	-0.365114	-2.640792	0.010468
C	0.925375	-0.296467	0.016973
C	1.618512	-0.257589	1.223674
C	3.013746	-0.186494	1.224704
C	3.747602	-0.152279	0.030221
C	3.020566	-0.19403	-1.173495
C	1.62995	-0.264979	-1.187606
F	-3.465145	4.331815	-0.006734
C	5.284609	-0.072364	-0.003898
C	5.900519	-0.035141	1.407567
C	5.846351	-1.309912	-0.74398
C	5.715309	1.211529	-0.752976
H	-0.860898	4.221567	0.004494
H	0.452717	2.119924	0.012352
H	-4.496389	1.986337	-0.009474
H	-2.497348	-3.844489	0.001936
H	-4.984857	-4.095531	-0.008786
H	-6.413207	-2.05189	-0.015903
H	-5.402348	0.172306	-0.012371
H	1.070489	-0.2841	2.160975
H	3.523135	-0.158904	2.180997
H	3.544783	-0.171763	-2.12429
H	1.089302	-0.29729	-2.128972
H	6.991821	0.021912	1.329265
H	5.563994	0.838462	1.977456
H	5.657073	-0.935277	1.983315
H	6.941323	-1.262844	-0.781431
H	5.479962	-1.37129	-1.774059
H	5.563026	-2.235747	-0.23033

H	5.338367	2.107007	-0.245367
H	6.809242	1.277784	-0.7909
H	5.344695	1.227493	-1.783289

SCF Done: E(RB3LYP) = -1118.36221818 A.U. after 14 cycles

Zero-point correction = 0.372888 (Hartree/Particle)

Thermal correction to Energy = 0.394492

Thermal correction to Enthalpy = 0.395436

Thermal correction to Gibbs Free Energy = 0.321657

Sum of electronic and zero-point Energies = -1117.989330

Sum of electronic and thermal Energies = -1117.967726

Sum of electronic and thermal Enthalpies = -1117.966782

Sum of electronic and thermal Free Energies = -1118.040562

12.2.2 Computed excitations energies of compound 4b (B3LYP/6-31G* PCM CH_2Cl_2)

Excitation energies and oscillator strengths:

Excited State 1: Singlet-A 3.9214 eV 316.17 nm f=0.2320 <S**2>=0.000
90 -> 93 -0.11391
91 -> 92 0.68202
This state for optimization and/or second-order correction.
Total Energy, E(TD-HF/TD-DFT) = -1118.21810996
Copying the excited state density for this state as the 1-particle RhoCI density.

Excited State 2: Singlet-A 4.2527 eV 291.54 nm f=0.0311 <S**2>=0.000
87 -> 92 -0.16898
91 -> 93 0.67190

Excited State 3: Singlet-A 4.5472 eV 272.66 nm f=0.0005 <S**2>=0.000
86 -> 92 0.36817
86 -> 93 0.27948
89 -> 92 0.48641
89 -> 93 0.18169

Excited State 4: Singlet-A 4.6162 eV 268.58 nm f=0.0506 <S**2>=0.000
87 -> 92 0.16784
87 -> 93 -0.12878
90 -> 92 0.59398
90 -> 93 0.24329
91 -> 96 0.15004

Excited State 5: Singlet-A 4.7795 eV 259.41 nm f=0.0005 <S**2>=0.000
86 -> 92 -0.36467
86 -> 93 -0.27793
89 -> 92 0.50348

89 -> 93 -0.13312

Excited State 6: Singlet-A 4.8504 eV 255.62 nm f=0.1342 <S**2>=0.000
87 -> 92 0.27119
88 -> 92 0.14729
90 -> 92 -0.23159
90 -> 93 0.48203
91 -> 92 0.11833
91 -> 94 0.18918
91 -> 96 -0.21484

Excited State 7: Singlet-A 4.8568 eV 255.28 nm f=0.0135 <S**2>=0.000
90 -> 93 -0.13448
91 -> 94 0.65936
91 -> 95 -0.12151

Excited State 8: Singlet-A 5.0078 eV 247.58 nm f=0.0256 <S**2>=0.000
87 -> 92 -0.14872
88 -> 92 0.66342
89 -> 93 -0.11195
90 -> 93 -0.11747

Excited State 9: Singlet-A 5.0388 eV 246.06 nm f=0.0010 <S**2>=0.000
89 -> 93 -0.12852
91 -> 94 0.12500
91 -> 95 0.66872

Excited State 10: Singlet-A 5.0435 eV 245.83 nm f=0.0013 <S**2>=0.000
86 -> 92 -0.37639
86 -> 93 0.15670
88 -> 92 0.10526
89 -> 93 0.53651
91 -> 95 0.15971

Excited State 11: Singlet-A 5.2383 eV 236.69 nm f=0.0018 <S**2>=0.000
86 -> 92 -0.26473
86 -> 93 0.52561
89 -> 93 -0.33360

Excited State 12: Singlet-A 5.2810 eV 234.77 nm f=0.2417 <S**2>=0.000
85 -> 92 0.10547
87 -> 92 0.45267
90 -> 93 -0.37603
91 -> 96 -0.29877

Excited State 13: Singlet-A 5.3573 eV 231.43 nm f=0.3859 <S**2>=0.000
85 -> 93 0.12627
87 -> 92 0.27699

87 -> 93 -0.12348
88 -> 93 0.10595
90 -> 92 -0.21149
90 -> 96 -0.13999
90 -> 97 0.10572
91 -> 93 0.12557
91 -> 96 0.49566
91 -> 97 0.14375

Excited State 14: Singlet-A 5.3998 eV 229.61 nm f=0.0123 <S**2>=0.000
88 -> 93 -0.33549
88 -> 94 0.36091
88 -> 95 -0.11403
89 -> 94 0.15887
89 -> 95 0.41195

Excited State 15: Singlet-A 5.4189 eV 228.80 nm f=0.0247 <S**2>=0.000
86 -> 93 -0.12126
88 -> 93 0.58969
88 -> 94 0.19791
89 -> 95 0.22308

12.2.3 Computed LE emission energies of compound 4b (B3LYP/6-31G* PCM CH_2Cl_2)

Excitation energies and oscillator strengths:
Excited State 1: Singlet-A 3.1103 eV 398.62 nm f=0.2521 <S**2>=0.000
91 -> 92 0.69985
This state for optimization and/or second-order correction.
Total Energy, E(TD-HF/TD-DFT) = -1118.22986912
Copying the excited state density for this state as the 1-particle RhoCI density.

Excited State 2: Singlet-A 3.6893 eV 336.07 nm f=0.0121 <S**2>=0.000
90 -> 92 -0.20799
91 -> 93 0.66591

Excited State 3: Singlet-A 3.9714 eV 312.19 nm f=0.0974 <S**2>=0.000
86 -> 92 0.11959
88 -> 92 0.11666
89 -> 92 -0.10689
90 -> 92 0.61609
91 -> 93 0.17222
91 -> 94 -0.16228

Excited State 4: Singlet-A 4.1772 eV 296.81 nm f=0.0218 <S**2>=0.000
86 -> 92 -0.31606
88 -> 92 -0.38342
89 -> 92 0.42146
90 -> 92 0.13704

Excited State 5: Singlet-A 4.3199 eV 287.01 nm f=0.0208 <S**2>=0.000
88 -> 92 0.47577
89 -> 92 0.48999
90 -> 93 -0.10270

Excited State 6: Singlet-A 4.3562 eV 284.62 nm f=0.2089 <S**2>=0.000
87 -> 92 -0.17232
90 -> 92 0.10524
90 -> 93 0.19935
91 -> 94 0.62497

Excited State 7: Singlet-A 4.6058 eV 269.19 nm f=0.0088 <S**2>=0.000
86 -> 92 0.52313
87 -> 92 0.12853
88 -> 92 -0.28957
88 -> 93 0.10093
89 -> 92 0.17842
90 -> 93 -0.15531
91 -> 95 0.16596

Excited State 8: Singlet-A 4.6563 eV 266.27 nm f=0.1856 <S**2>=0.000
86 -> 92 0.19291
87 -> 92 -0.33882
89 -> 92 0.11978
90 -> 92 -0.16667
90 -> 93 0.45933
91 -> 94 -0.22635
91 -> 96 0.11102

Excited State 9: Singlet-A 4.8364 eV 256.36 nm f=0.1075 <S**2>=0.000
86 -> 92 -0.10529
89 -> 92 -0.11680
89 -> 94 -0.18499
91 -> 95 0.61916
91 -> 96 0.11643

Excited State 10: Singlet-A 4.9626 eV 249.84 nm f=0.2433 <S**2>=0.000
87 -> 92 0.36198
88 -> 93 0.36961
90 -> 93 0.35544
90 -> 94 -0.13017
91 -> 96 -0.22732

Excited State 11: Singlet-A 5.0763 eV 244.24 nm f=0.0304 <S**2>=0.000
86 -> 92 0.12753
86 -> 93 -0.12981
88 -> 93 -0.15130

89 -> 93 0.64901

Excited State 12: Singlet-A 5.1349 eV 241.45 nm f=0.1978 <S**2>=0.000
88 -> 93 0.35062
89 -> 93 0.13736
90 -> 93 -0.10775
90 -> 94 -0.27923
91 -> 95 -0.11234
91 -> 96 0.45750

Excited State 13: Singlet-A 5.1751 eV 239.58 nm f=0.1728 <S**2>=0.000
85 -> 92 -0.14235
87 -> 92 -0.32646
88 -> 93 0.38691
90 -> 93 -0.21625
90 -> 94 0.14900
91 -> 96 -0.31687

Excited State 14: Singlet-A 5.3599 eV 231.32 nm f=0.0261 <S**2>=0.000
86 -> 93 0.13706
87 -> 92 0.10299
88 -> 93 0.10357
89 -> 93 0.13816
90 -> 94 0.45203
91 -> 96 0.15359
91 -> 97 0.40092

Excited State 15: Singlet-A 5.4865 eV 225.98 nm f=0.2097 <S**2>=0.000
85 -> 92 -0.38739
86 -> 93 -0.18495
87 -> 92 0.15397
87 -> 93 0.48183
91 -> 97 0.11572

12.2.4 Potential scan of compound 4b ground state (B3LYP/6-31G* PCM CH_2Cl_2)

D(4,7,16,17)
0 -1118.36221818 89.7321
8 -1118.36203573 99.7321
13 -1118.36151163 109.7321
17 -1118.36056026 119.7321
22 -1118.35912163 129.732
27 -1118.35692421 139.732
32 -1118.35381294 149.732
37 -1118.34981414 159.732
43 -1118.34559512 169.732
48 -1118.34185493 179.732
53 -1118.33873715 -170.268

59 -1118.3364122 -160.268
65 -1118.33518676 -150.2679
79 -1118.35679542 -140.2678
84 -1118.35903127 -130.2678
88 -1118.36053027 -120.2678
92 -1118.36148199 -110.2678
96 -1118.36202852 -100.2678
101 -1118.36222523 -90.2678
105 -1118.36206125 -80.2678
109 -1118.36149877 -70.2679
114 -1118.36048434 -60.268
118 -1118.35886609 -50.2679
122 -1118.35649963 -40.2679
127 -1118.35323519 -30.268
133 -1118.34914562 -20.268
140 -1118.34471913 -10.268
146 -1118.34082082 -0.268
152 -1118.33776926 9.732
158 -1118.3358048 19.732
175 -1118.35303058 29.7322
180 -1118.35630923 39.7323
184 -1118.35874732 49.7322
188 -1118.36041406 59.7323
193 -1118.36146025 69.7322
198 -1118.36201614 79.7322
202 -1118.36220704 89.7322

Progress of structural optimization and emission calculation

-1118.362218
-1118.361918
-1118.362018
-1118.362024
-1118.362033
-1118.362035
-1118.362036
-1118.362036
-1118.362036
-1118.361392
-1118.361507
-1118.361509
-1118.361512
-1118.361512
-1118.360451
-1118.360559
-1118.36056
-1118.36056
-1118.358964
-1118.359119

-1118.359121
-1118.359122
-1118.359122
-1118.356756
-1118.356916
-1118.356923
-1118.356924
-1118.356924
-1118.353553
-1118.353802
-1118.353812
-1118.353813
-1118.353813
-1118.349361
-1118.34978
-1118.349811
-1118.349814
-1118.349814
-1118.344719
-1118.345453
-1118.345583
-1118.345593
-1118.345595
-1118.345595
-1118.340985
-1118.341798
-1118.341852
-1118.341855
-1118.341855
-1118.337883
-1118.338691
-1118.338735
-1118.338737
-1118.338737
-1118.335499
-1118.336362
-1118.336408
-1118.336412
-1118.336412
-1118.336412
-1118.334155
-1118.335079
-1118.335171
-1118.335185
-1118.335187
-1118.335187
-1118.334321
-1118.335809

-1118.337218
-1118.331216
-1118.344828
-1118.319631
-1118.35213
-1118.355111
-1118.356051
-1118.356641
-1118.356778
-1118.356793
-1118.356795
-1118.356795
-1118.358856
-1118.359025
-1118.359031
-1118.359031
-1118.359031
-1118.360399
-1118.360529
-1118.36053
-1118.36053
-1118.361358
-1118.361481
-1118.361482
-1118.361482
-1118.361911
-1118.362027
-1118.362028
-1118.362029
-1118.362093
-1118.362222
-1118.362225
-1118.362225
-1118.362225
-1118.36192
-1118.36206
-1118.362061
-1118.362061
-1118.361322
-1118.361497
-1118.361499
-1118.361499
-1118.360251
-1118.360477
-1118.360484
-1118.360484
-1118.360484
-1118.358581

-1118.358853
-1118.358866
-1118.358866
-1118.356149
-1118.356473
-1118.356499
-1118.3565
-1118.352781
-1118.353202
-1118.353234
-1118.353235
-1118.353235
-1118.348532
-1118.34908
-1118.34914
-1118.349145
-1118.349146
-1118.349146
-1118.343788
-1118.344573
-1118.344688
-1118.344718
-1118.344719
-1118.344719
-1118.344719
-1118.339681
-1118.3407
-1118.340807
-1118.34082
-1118.340821
-1118.340821
-1118.336703
-1118.337661
-1118.337745
-1118.337768
-1118.337769
-1118.337769
-1118.334762
-1118.335672
-1118.335778
-1118.335804
-1118.335805
-1118.335805
-1118.334312
-1118.335229
-1118.335426
-1118.335653
-1118.335733

-1118.335901
-1118.337361
-1118.340849
-1118.344827
-1118.348816
-1118.350737
-1118.352391
-1118.352807
-1118.35302
-1118.353029
-1118.35303
-1118.353031
-1118.35591
-1118.356276
-1118.356308
-1118.356309
-1118.356309
-1118.35844
-1118.358737
-1118.358747
-1118.358747
-1118.360175
-1118.360407
-1118.360414
-1118.360414
-1118.361251
-1118.361455
-1118.36146
-1118.36146
-1118.36146
-1118.361831
-1118.362012
-1118.362016
-1118.362016
-1118.362016
-1118.362058
-1118.362206
-1118.362207

Geometries of structural optimization

1 Energy:-1118.36221818
C -2.746525 3.184320 -0.002734
C -1.356662 3.256873 0.003212
C -0.628506 2.075155 0.007489
C -1.276129 0.825937 0.005774
C -2.692608 0.767060 -0.000664
C -3.413465 1.976299 -0.004729

N -0.519074 -0.362816 0.010394
C -1.080226 -1.638008 0.007529
C -2.555630 -1.704276 0.000985
C -3.352843 -0.539280 -0.002968
C -3.148710 -2.977518 -0.001206
C -4.528507 -3.110176 -0.007185
C -5.330818 -1.959202 -0.011158
C -4.754266 -0.696784 -0.009117
O -0.365114 -2.640792 0.010468
C 0.925375 -0.296467 0.016973
C 1.618512 -0.257589 1.223674
C 3.013746 -0.186494 1.224704
C 3.747602 -0.152279 0.030221
C 3.020566 -0.194030 -1.173495
C 1.629950 -0.264979 -1.187606
F -3.465145 4.331815 -0.006734
C 5.284609 -0.072364 -0.003898
C 5.900519 -0.035141 1.407567
C 5.846351 -1.309912 -0.743980
C 5.715309 1.211529 -0.752976
H -0.860898 4.221567 0.004494
H 0.452717 2.119924 0.012352
H -4.496389 1.986337 -0.009474
H -2.497348 -3.844489 0.001936
H -4.984857 -4.095531 -0.008786
H -6.413207 -2.051890 -0.015903
H -5.402348 0.172306 -0.012371
H 1.070489 -0.284100 2.160975
H 3.523135 -0.158904 2.180997
H 3.544783 -0.171763 -2.124290
H 1.089302 -0.297290 -2.128972
H 6.991821 0.021912 1.329265
H 5.563994 0.838462 1.977456
H 5.657073 -0.935277 1.983315
H 6.941323 -1.262844 -0.781431
H 5.479962 -1.371290 -1.774059
H 5.563026 -2.235747 -0.230330
H 5.338367 2.107007 -0.245367
H 6.809242 1.277784 -0.790900
H 5.344695 1.227493 -1.783289

2 Energy:-1118.36203573
C -2.763585 3.183421 -0.160651
C -1.375345 3.265575 -0.105272
C -0.641804 2.089916 -0.026058
C -1.281790 0.836820 -0.005410
C -2.697048 0.768842 -0.051968
C -3.423607 1.971966 -0.133187

N -0.518515 -0.345432 0.076818
C -1.073850 -1.623328 0.116877
C -2.548525 -1.697973 0.081066
C -3.350758 -0.539893 -0.007553
C -3.135453 -2.973205 0.129146
C -4.513903 -3.114436 0.091732
C -5.321114 -1.970304 0.003284
C -4.750679 -0.706077 -0.045726
O -0.355180 -2.621815 0.174359
C 0.925344 -0.272394 0.067074
C 1.639109 -0.354916 1.259314
C 3.034532 -0.294336 1.242842
C 3.749065 -0.152097 0.044525
C 3.001956 -0.075349 -1.144954
C 1.610761 -0.137379 -1.141717
F -3.487551 4.324734 -0.240111
C 5.286007 -0.081450 -0.008533
C 5.925157 -0.173832 1.390029
C 5.823668 -1.253460 -0.864210
C 5.717069 1.259815 -0.648845
H -0.885045 4.232908 -0.122171
H 0.438004 2.142652 0.021462
H -4.505820 1.974566 -0.173346
H -2.480475 -3.834869 0.195603
H -4.965431 -4.101257 0.130107
H -6.402467 -2.069769 -0.027391
H -5.402388 0.157609 -0.113987
H 1.106615 -0.466766 2.199057
H 3.559875 -0.361683 2.188460
H 3.510352 0.030656 -2.098667
H 1.055270 -0.082179 -2.073417
H 7.015450 -0.117065 1.298518
H 5.604800 0.647850 2.040743
H 5.684280 -1.119967 1.887794
H 6.918309 -1.212409 -0.916000
H 5.439880 -1.220368 -1.889237
H 5.539724 -2.218639 -0.429400
H 5.356432 2.110204 -0.058594
H 6.810850 1.320406 -0.698496
H 5.331018 1.369257 -1.667753

3 Energy:-1118.36151163
C -2.782339 3.175963 -0.317066
C -1.398354 3.273854 -0.207699
C -0.659363 2.109541 -0.051450
C -1.287939 0.850876 -0.010611
C -2.700202 0.768760 -0.102910
C -3.432731 1.960401 -0.263124

N -0.517736 -0.319444 0.153727
C -1.068919 -1.598446 0.242917
C -2.541416 -1.685218 0.167641
C -3.347090 -0.540955 -0.014855
C -3.121696 -2.960847 0.264577
C -4.496833 -3.115463 0.184457
C -5.307380 -1.984916 0.001876
C -4.743474 -0.720659 -0.096404
O -0.349136 -2.589857 0.366384
C 0.925434 -0.242428 0.126711
C 1.660975 -0.440791 1.291872
C 3.056296 -0.393683 1.253715
C 3.751045 -0.152694 0.059593
C 2.983164 0.035243 -1.103853
C 1.591810 -0.015051 -1.079671
F -3.511781 4.305714 -0.473085
C 5.287400 -0.093046 -0.016710
C 5.951321 -0.317932 1.354979
C 5.795548 -1.188023 -0.985107
C 5.721857 1.296361 -0.541730
H -0.915659 4.244586 -0.240599
H 0.416486 2.176387 0.042858
H -4.512763 1.950832 -0.341544
H -2.464239 -3.812163 0.401748
H -4.943189 -4.102400 0.261162
H -6.386126 -2.095067 -0.063850
H -5.397583 0.132275 -0.237667
H 1.145513 -0.631372 2.228039
H 3.597737 -0.551460 2.179300
H 3.474891 0.217705 -2.054739
H 1.021688 0.122511 -1.993864
H 7.040343 -0.264580 1.247506
H 5.653240 0.444955 2.083214
H 5.708053 -1.302534 1.770091
H 6.889401 -1.152853 -1.054818
H 5.392844 -1.058964 -1.995225
H 5.509094 -2.185764 -0.633385
H 5.381756 2.093346 0.129676
H 6.815159 1.350821 -0.607039
H 5.318084 1.501031 -1.538879

4 Energy:-1118.36056026
C -2.803233 3.161677 -0.470584
C -1.427367 3.282659 -0.299501
C -0.683102 2.135247 -0.064758
C -1.295492 0.868690 -0.009857
C -2.701994 0.766260 -0.154004
C -3.440540 1.940635 -0.395236

N -0.517111 -0.283407 0.237492
C -1.064167 -1.563537 0.366806
C -2.532945 -1.666962 0.250647
C -3.340830 -0.543683 -0.025193
C -3.105722 -2.941632 0.395595
C -4.475357 -3.115027 0.272215
C -5.287844 -2.005154 -0.004301
C -4.731309 -0.742347 -0.150871
O -0.344928 -2.545319 0.551962
C 0.925281 -0.202342 0.193124
C 1.681788 -0.512422 1.320288
C 3.076492 -0.481465 1.257457
C 3.752858 -0.151163 0.074417
C 2.965294 0.143255 -1.053166
C 1.574300 0.109129 -1.004983
F -3.537976 4.274065 -0.705318
C 5.288128 -0.107610 -0.027503
C 5.973621 -0.461541 1.305846
C 5.760860 -1.121009 -1.097346
C 5.735164 1.316000 -0.437787
H -0.955271 4.258135 -0.343718
H 0.385261 2.222638 0.082026
H -4.516130 1.913378 -0.517684
H -2.446859 -3.777302 0.603471
H -4.915902 -4.100878 0.386969
H -6.362212 -2.130282 -0.105339
H -5.386765 0.094381 -0.364221
H 1.183621 -0.775311 2.247598
H 3.632324 -0.725350 2.155444
H 3.440449 0.395041 -1.996677
H 0.991400 0.325144 -1.895526
H 7.061243 -0.416001 1.181651
H 5.702314 0.239400 2.103488
H 5.720981 -1.474658 1.638612
H 6.853804 -1.096737 -1.183961
H 5.343434 -0.897844 -2.084809
H 5.463629 -2.141267 -0.828990
H 5.421981 2.055628 0.308196
H 6.827687 1.359928 -0.521823
H 5.314330 1.613510 -1.403989

5 Energy:-1118.35912163
C -2.809158 3.141630 -0.611056
C -1.447619 3.288883 -0.362881
C -0.704688 2.161088 -0.045253
C -1.300158 0.886075 0.012636
C -2.695847 0.761019 -0.199470
C -3.434111 1.915292 -0.525253

N -0.518207 -0.243722 0.348871
C -1.068862 -1.519719 0.526839
C -2.529615 -1.642644 0.344382
C -3.329809 -0.547723 -0.042353
C -3.099036 -2.913929 0.529053
C -4.457261 -3.110790 0.335230
C -5.261412 -2.029098 -0.054096
C -4.708176 -0.770054 -0.240154
O -0.359966 -2.488431 0.798140
C 0.924028 -0.164949 0.281359
C 1.706131 -0.576837 1.358418
C 3.099176 -0.561487 1.260608
C 3.752220 -0.153417 0.089378
C 2.940649 0.233541 -0.992151
C 1.551441 0.216622 -0.909710
F -3.542811 4.233846 -0.928319
C 5.284774 -0.121097 -0.048268
C 5.997660 -0.584383 1.236275
C 5.717552 -1.054200 -1.204491
C 5.740950 1.324539 -0.359746
H -0.986573 4.269499 -0.409537
H 0.350613 2.271286 0.166181
H -4.501402 1.868331 -0.701931
H -2.446587 -3.728734 0.822068
H -4.895047 -4.093773 0.480954
H -6.326393 -2.173371 -0.212576
H -5.356611 0.044715 -0.541623
H 1.230932 -0.900862 2.277169
H 3.672209 -0.881136 2.123399
H 3.395018 0.544201 -1.928350
H 0.953489 0.501518 -1.770043
H 7.082507 -0.542520 1.088607
H 5.755400 0.056566 2.091637
H 5.739007 -1.616887 1.497052
H 6.808203 -1.035199 -1.317443
H 5.278397 -0.750935 -2.160706
H 5.414071 -2.088823 -1.007621
H 5.456096 2.008318 0.448302
H 6.831608 1.362194 -0.468022
H 5.300414 1.700107 -1.289349

6 Energy:-1118.35692421
C -2.817556 3.119673 -0.735630
C -1.478314 3.302719 -0.403280
C -0.737466 2.199711 -0.005562
C -1.307674 0.912379 0.045453
C -2.685956 0.755540 -0.241359
C -3.423592 1.884281 -0.649099

N -0.521012 -0.187037 0.470946
C -1.072190 -1.461760 0.680937
C -2.520287 -1.613300 0.428095
C -3.310167 -0.554776 -0.064375
C -3.083093 -2.882786 0.644453
C -4.423757 -3.112298 0.377856
C -5.216507 -2.066793 -0.118729
C -4.669815 -0.809993 -0.336627
O -0.374313 -2.413138 1.029407
C 0.921414 -0.108839 0.381560
C 1.726132 -0.617089 1.400779
C 3.116401 -0.621864 1.264380
C 3.748331 -0.148899 0.107068
C 2.915579 0.326070 -0.921929
C 1.529772 0.332977 -0.800939
F -3.549551 4.186357 -1.132473
C 5.276610 -0.140428 -0.072099
C 6.013372 -0.696242 1.161181
C 5.655806 -1.010220 -1.294602
C 5.757388 1.311349 -0.308884
H -1.034648 4.291524 -0.444791
H 0.297995 2.339086 0.275744
H -4.477713 1.810532 -0.885364
H -2.439562 -3.670737 1.018928
H -4.856285 -4.093537 0.548791
H -6.267142 -2.237463 -0.335820
H -5.308507 -0.023097 -0.721408
H 1.274047 -0.992562 2.309974
H 3.703661 -1.012367 2.087638
H 3.349756 0.686045 -1.850118
H 0.920088 0.681357 -1.628402
H 7.094614 -0.666203 0.986529
H 5.806860 -0.105155 2.060696
H 5.740194 -1.737783 1.364972
H 6.743008 -1.007325 -1.438453
H 5.196573 -0.640097 -2.217269
H 5.334538 -2.048326 -1.151543
H 5.510721 1.950588 0.546780
H 6.845244 1.332325 -0.446192
H 5.300196 1.752149 -1.201061

7 Energy:-1118.35381294
C -2.812308 3.093028 -0.856104
C -1.506904 3.318852 -0.427926
C -0.774619 2.244834 0.054568
C -1.313965 0.943442 0.092753
C -2.664565 0.747825 -0.282269
C -3.395451 1.846500 -0.776357

N -0.527453 -0.119283 0.612193
C -1.088141 -1.384375 0.872227
C -2.513151 -1.574467 0.525631
C -3.277518 -0.565402 -0.092812
C -3.071802 -2.840358 0.772646
C -4.381369 -3.114071 0.409914
C -5.146278 -2.118492 -0.215842
C -4.604024 -0.865171 -0.463422
O -0.415295 -2.307207 1.328376
C 0.916181 -0.049806 0.498734
C 1.744314 -0.646166 1.451960
C 3.129588 -0.673150 1.268275
C 3.738709 -0.145217 0.124042
C 2.884189 0.411912 -0.843955
C 1.504479 0.444841 -0.676152
F -3.536410 4.129981 -1.336352
C 5.259827 -0.162323 -0.105305
C 6.020232 -0.812804 1.065778
C 5.577457 -0.961815 -1.391728
C 5.770739 1.289458 -0.266850
H -1.085538 4.317705 -0.459371
H 0.231880 2.416857 0.413440
H -4.429295 1.740993 -1.080380
H -2.449444 -3.591498 1.245365
H -4.810347 -4.092451 0.604514
H -6.171192 -2.325315 -0.510523
H -5.219489 -0.116552 -0.949293
H 1.319405 -1.063607 2.353684
H 3.730484 -1.128403 2.047357
H 3.295246 0.815639 -1.764715
H 0.884242 0.851286 -1.467464
H 7.095852 -0.798961 0.857471
H 5.858294 -0.274503 2.006617
H 5.725398 -1.857872 1.213737
H 6.659259 -0.975738 -1.571433
H 5.098865 -0.523403 -2.273785
H 5.234201 -1.998973 -1.303071
H 5.568239 1.878999 0.634864
H 6.853856 1.293055 -0.439002
H 5.296819 1.796724 -1.113876

8 Energy:-1118.34981414
C -2.798124 3.065913 -0.963437
C -1.537241 3.340845 -0.439859
C -0.816919 2.299512 0.124632
C -1.318716 0.982889 0.148314
C -2.630155 0.740493 -0.320413
C -3.351830 1.805352 -0.896043

N -0.538062 -0.038821 0.760190
C -1.127753 -1.271823 1.108990
C -2.509819 -1.519876 0.639358
C -3.225516 -0.579571 -0.126758
C -3.066370 -2.780207 0.917354
C -4.321220 -3.116986 0.433275
C -5.032302 -2.192319 -0.345628
C -4.493832 -0.942845 -0.620971
O -0.508783 -2.137221 1.724906
C 0.907367 0.005940 0.620790
C 1.755868 -0.677454 1.498703
C 3.133175 -0.728774 1.262129
C 3.721920 -0.145581 0.135995
C 2.849297 0.494551 -0.761413
C 1.478540 0.555068 -0.542056
F -3.511721 4.070215 -1.521514
C 5.232970 -0.188275 -0.147136
C 6.012146 -0.933415 0.953040
C 5.484879 -0.910796 -1.492309
C 5.779605 1.256841 -0.233533
H -1.144668 4.351622 -0.459437
H 0.152525 2.507424 0.559894
H -4.359382 1.663306 -1.266527
H -2.485100 -3.478475 1.508384
H -4.747592 -4.091773 0.650383
H -6.011679 -2.451144 -0.737859
H -5.064623 -0.248180 -1.226962
H 1.357736 -1.138450 2.388622
H 3.744009 -1.250036 1.990687
H 3.237080 0.943124 -1.671633
H 0.852137 1.021418 -1.292889
H 7.080423 -0.935127 0.709334
H 5.896454 -0.453097 1.931335
H 5.691851 -1.977524 1.044045
H 6.559263 -0.941576 -1.710504
H 4.989931 -0.403699 -2.327299
H 5.115360 -1.942168 -1.458125
H 5.625128 1.791481 0.710904
H 6.855807 1.243418 -0.444360
H 5.291128 1.830348 -1.028446

9 Energy:-1118.34559512
C -2.810852 3.042672 -1.061382
C -1.595308 3.377382 -0.469517
C -0.873644 2.376529 0.163661
C -1.328012 1.043918 0.186640
C -2.596890 0.741077 -0.354605
C -3.322931 1.763805 -0.996095

N -0.549956 0.063932 0.871397
C -1.185924 -1.100884 1.353787
C -2.499663 -1.443845 0.759342
C -3.156303 -0.593423 -0.151082
C -3.041029 -2.702433 1.070602
C -4.216655 -3.128736 0.470043
C -4.862624 -2.296578 -0.455612
C -4.341688 -1.045793 -0.760764
O -0.650409 -1.848975 2.168231
C 0.894465 0.060969 0.706112
C 1.741667 -0.725350 1.499533
C 3.109557 -0.809189 1.221490
C 3.699642 -0.153604 0.137575
C 2.830676 0.590859 -0.677662
C 1.468504 0.684615 -0.419591
F -3.526500 4.007436 -1.682288
C 5.201225 -0.226474 -0.185120
C 5.975119 -1.085405 0.832704
C 5.396698 -0.846299 -1.589604
C 5.799861 1.200331 -0.169404
H -1.241508 4.402373 -0.487988
H 0.058547 2.628371 0.654762
H -4.304354 1.575517 -1.413733
H -2.510513 -3.330397 1.777159
H -4.629736 -4.103978 0.709640
H -5.777063 -2.628300 -0.939056
H -4.858031 -0.421245 -1.481623
H 1.347646 -1.246105 2.356381
H 3.712751 -1.415268 1.888494
H 3.212507 1.100582 -1.557891
H 0.852954 1.230872 -1.122127
H 7.037499 -1.104451 0.565012
H 5.896500 -0.682789 1.849021
H 5.618888 -2.121729 0.846463
H 6.464043 -0.896796 -1.836891
H 4.901899 -0.256263 -2.368335
H 4.990274 -1.863539 -1.628799
H 5.685649 1.662423 0.818053
H 6.870083 1.165845 -0.406775
H 5.316132 1.852890 -0.904000

10 Energy:-1118.34185493
C -2.846602 3.004312 -1.179787
C -1.669011 3.401411 -0.551872
C -0.935572 2.445971 0.137797
C -1.340496 1.099206 0.178591
C -2.577563 0.734553 -0.396978
C -3.316529 1.709564 -1.093701

N -0.555978 0.159923 0.914005
C -1.219554 -0.943509 1.498124
C -2.483674 -1.373082 0.853289
C -3.102626 -0.608497 -0.154884
C -3.007997 -2.623968 1.217307
C -4.125777 -3.130479 0.568858
C -4.729462 -2.386802 -0.455000
C -4.227277 -1.140857 -0.810755
O -0.736617 -1.578485 2.431465
C 0.885604 0.113007 0.732331
C 1.703421 -0.786880 1.434145
C 3.064763 -0.901816 1.140856
C 3.684983 -0.161128 0.130504
C 2.847227 0.699325 -0.595701
C 1.489489 0.825501 -0.322665
F -3.572983 3.923708 -1.854265
C 5.182689 -0.260833 -0.202210
C 5.916310 -1.261858 0.710142
C 5.356301 -0.725086 -1.668194
C 5.841367 1.128293 -0.026199
H -1.354715 4.438832 -0.584546
H -0.037013 2.747011 0.663062
H -4.277595 1.473830 -1.534605
H -2.510190 -3.184080 2.000774
H -4.525044 -4.101128 0.847334
H -5.596784 -2.783109 -0.975205
H -4.709590 -0.583312 -1.606518
H 1.291559 -1.374746 2.238098
H 3.639617 -1.602002 1.737165
H 3.248758 1.283633 -1.419003
H 0.904905 1.465141 -0.968832
H 6.977587 -1.296515 0.439605
H 5.852577 -0.973761 1.765644
H 5.515337 -2.276675 0.607525
H 6.421360 -0.792601 -1.921286
H 4.889738 -0.029992 -2.374312
H 4.907098 -1.713059 -1.822033
H 5.744330 1.479857 1.007547
H 6.909784 1.075989 -0.268625
H 5.387037 1.879742 -0.680833

11 Energy:-1118.33873715
C -2.887062 2.951184 -1.306836
C -1.741303 3.406386 -0.661722
C -0.993685 2.496614 0.075561
C -1.352333 1.138992 0.142624
C -2.567971 0.718516 -0.442800
C -3.320485 1.645317 -1.186851

N -0.556895 0.235385 0.911496
C -1.230912 -0.816612 1.575590
C -2.469046 -1.312322 0.927948
C -3.067893 -0.624604 -0.146288
C -2.981512 -2.547606 1.354237
C -4.066290 -3.117667 0.701216
C -4.646991 -2.453242 -0.387894
C -4.157470 -1.220493 -0.804853
O -0.767030 -1.360096 2.573042
C 0.881524 0.154529 0.721748
C 1.654118 -0.858013 1.315690
C 3.012645 -0.995105 1.025174
C 3.679804 -0.162366 0.120855
C 2.888702 0.813653 -0.501292
C 1.532211 0.965125 -0.227804
F -3.625563 3.824071 -2.028092
C 5.177261 -0.284452 -0.205236
C 5.854603 -1.418771 0.586744
C 5.354747 -0.575168 -1.714758
C 5.889718 1.043738 0.144966
H -1.462795 4.453110 -0.715241
H -0.128846 2.847566 0.625354
H -4.265815 1.366083 -1.636558
H -2.500461 -3.046950 2.187899
H -4.456505 -4.077217 1.026899
H -5.487394 -2.900207 -0.911192
H -4.623322 -0.723157 -1.649052
H 1.211917 -1.525459 2.038160
H 3.547572 -1.789194 1.534795
H 3.325776 1.478868 -1.240968
H 0.990353 1.703375 -0.800143
H 6.917822 -1.465648 0.325794
H 5.785561 -1.258021 1.668676
H 5.414229 -2.395776 0.357106
H 6.419958 -0.655823 -1.963423
H 4.926890 0.218972 -2.335843
H 4.868732 -1.517796 -1.991928
H 5.791434 1.271539 1.212706
H 6.958667 0.975833 -0.091271
H 5.476528 1.887457 -0.418078

12 Energy:-1118.3364122
C -2.934416 2.880772 -1.445599
C -1.809941 3.389860 -0.805612
C -1.045167 2.528154 -0.026901
C -1.364364 1.164234 0.081996
C -2.570314 0.693781 -0.488986
C -3.339003 1.570493 -1.274028

N -0.553411 0.292651 0.871728
C -1.221506 -0.713054 1.610430
C -2.455231 -1.257107 0.994575
C -3.051671 -0.640644 -0.124010
C -2.959301 -2.468650 1.491654
C -4.032984 -3.088375 0.865389
C -4.609378 -2.497625 -0.267335
C -4.128452 -1.286979 -0.754511
O -0.746916 -1.183733 2.638531
C 0.881637 0.188416 0.677670
C 1.598768 -0.927129 1.145044
C 2.957646 -1.081668 0.872052
C 3.682661 -0.160744 0.106953
C 2.948368 0.923121 -0.391436
C 1.590773 1.095689 -0.129146
F -3.687398 3.704898 -2.207909
C 5.183227 -0.299890 -0.197500
C 5.793025 -1.560023 0.445195
C 5.393604 -0.386973 -1.728141
C 5.935609 0.936075 0.350743
H -1.561250 4.441950 -0.891114
H -0.211211 2.930577 0.533712
H -4.275984 1.251727 -1.714985
H -2.479773 -2.912226 2.357236
H -4.417022 -4.030221 1.245485
H -5.440330 -2.984369 -0.769810
H -4.592365 -0.845992 -1.630546
H 1.113435 -1.675735 1.752245
H 3.445456 -1.961881 1.276537
H 3.432738 1.667130 -1.017887
H 1.100956 1.932141 -0.602521
H 6.860949 -1.614944 0.205901
H 5.698236 -1.547243 1.536999
H 5.322861 -2.476858 0.071580
H 6.461698 -0.477824 -1.960379
H 5.015282 0.502681 -2.242802
H 4.879617 -1.260753 -2.145194
H 5.814061 1.018785 1.437008
H 7.007768 0.856148 0.133574
H 5.572202 1.865907 -0.099729

13 Energy:-1118.33518676
C -3.002886 2.782608 -1.607896
C -1.873578 3.335228 -1.017386
C -1.083226 2.524707 -0.208057
C -1.380518 1.164793 -0.021449
C -2.599470 0.656623 -0.536507
C -3.392492 1.481250 -1.351508

N -0.545187 0.319443 0.771043
C -1.184946 -0.639968 1.591388
C -2.443273 -1.208468 1.053789
C -3.069630 -0.654947 -0.081454
C -2.937614 -2.384525 1.637710
C -4.032168 -3.034086 1.081690
C -4.637996 -2.509168 -0.067920
C -4.166501 -1.332755 -0.640743
O -0.664986 -1.058068 2.619853
C 0.889413 0.214001 0.581846
C 1.545577 -0.990493 0.892915
C 2.908605 -1.157438 0.659312
C 3.700527 -0.152451 0.088626
C 3.031183 1.029932 -0.248374
C 1.668573 1.217173 -0.015258
F -3.779101 3.555572 -2.400083
C 5.208255 -0.303756 -0.172624
C 5.738721 -1.682870 0.262667
C 5.490763 -0.131760 -1.684274
C 5.977601 0.782558 0.616318
H -1.639397 4.384509 -1.159607
H -0.263109 2.979254 0.328778
H -4.336763 1.131714 -1.751439
H -2.433559 -2.778740 2.513325
H -4.409190 -3.949036 1.528639
H -5.485007 -3.020342 -0.516461
H -4.654797 -0.943132 -1.527803
H 1.002948 -1.813880 1.334202
H 3.345381 -2.112788 0.928834
H 3.573407 1.849437 -0.711835
H 1.242344 2.150980 -0.341381
H 6.813960 -1.743715 0.060607
H 5.592999 -1.855981 1.335024
H 5.252494 -2.498285 -0.285005
H 6.564796 -0.230764 -1.883345
H 5.173670 0.851029 -2.048992
H 4.964789 -0.894129 -2.270559
H 5.805877 0.679735 1.694034
H 7.055446 0.693518 0.433565
H 5.671258 1.792028 0.321955

14 Energy:-1118.35679542
C -2.857767 2.376571 -2.114348
C -1.511656 2.261770 -2.449459
C -0.752698 1.286863 -1.819277
C -1.311921 0.438007 -0.843697
C -2.696304 0.531336 -0.558751
C -3.452475 1.530357 -1.202665

N -0.505692 -0.554611 -0.232252
C -1.041932 -1.518879 0.637186
C -2.495816 -1.452989 0.893204
C -3.306647 -0.433537 0.354654
C -3.043623 -2.422572 1.750426
C -4.390003 -2.391321 2.078119
C -5.203792 -1.376669 1.552438
C -4.672018 -0.414152 0.705823
O -0.327931 -2.359258 1.182656
C 0.932989 -0.396620 -0.219487
C 1.771076 -1.491552 -0.428522
C 3.156508 -1.349186 -0.320084
C 3.750665 -0.128088 0.023803
C 2.884602 0.953528 0.264301
C 1.503172 0.824808 0.163739
F -3.607427 3.328944 -2.716049
C 5.272648 0.059970 0.150496
C 6.049056 -1.236686 -0.147227
C 5.740390 1.144281 -0.849705
C 5.617548 0.509959 1.590511
H -1.076325 2.915990 -3.196978
H 0.288862 1.177953 -2.091350
H -4.511868 1.644128 -1.009961
H -2.383919 -3.186099 2.146672
H -4.810896 -3.143753 2.738293
H -6.259241 -1.340064 1.806954
H -5.327268 0.356605 0.316237
H 1.349053 -2.452180 -0.694970
H 3.770341 -2.223210 -0.505601
H 3.288476 1.919239 0.553783
H 0.867172 1.675689 0.385568
H 7.124248 -1.051695 -0.045992
H 5.784610 -2.039962 0.549755
H 5.869661 -1.594870 -1.167364
H 6.823412 1.295063 -0.765791
H 5.254223 2.107803 -0.663730
H 5.517708 0.848269 -1.881424
H 5.304139 -0.243670 2.322076
H 6.699929 0.653835 1.692835
H 5.129922 1.455199 1.851310

15 Energy:-1118.35903127
C -2.847602 2.286881 -2.215895
C -1.480815 2.217869 -2.469269
C -0.721205 1.293240 -1.767345
C -1.305406 0.447394 -0.804583
C -2.705515 0.500454 -0.591657
C -3.460922 1.448065 -1.309215

N -0.505963 -0.492779 -0.113097
C -1.042075 -1.431709 0.778220
C -2.506600 -1.403375 0.969215
C -3.325397 -0.446244 0.335043
C -3.061322 -2.347120 1.850118
C -4.423150 -2.352736 2.107970
C -5.245910 -1.401868 1.485710
C -4.707303 -0.464949 0.615061
O -0.318824 -2.221148 1.385158
C 0.932959 -0.350897 -0.114449
C 1.749463 -1.433574 -0.434338
C 3.138827 -1.309329 -0.365826
C 3.754012 -0.116245 0.037599
C 2.907992 0.953708 0.380562
C 1.522083 0.841051 0.321861
F -3.597708 3.189489 -2.889990
C 5.281437 0.052969 0.121610
C 6.035663 -1.223506 -0.296331
C 5.722570 1.203434 -0.814711
C 5.681197 0.396153 1.576743
H -1.028324 2.870013 -3.208393
H 0.338683 1.222166 -1.972529
H -4.532321 1.530143 -1.175529
H -2.394613 -3.061896 2.319148
H -4.849499 -3.084842 2.787229
H -6.314002 -1.394944 1.683795
H -5.370148 0.256319 0.150682
H 1.304131 -2.371192 -0.746421
H 3.739218 -2.171692 -0.631867
H 3.332080 1.895982 0.714809
H 0.897027 1.679519 0.613666
H 7.115264 -1.051696 -0.222828
H 5.791375 -2.072951 0.351537
H 5.815537 -1.507255 -1.331794
H 6.809213 1.340570 -0.759673
H 5.252484 2.154313 -0.542489
H 5.460348 0.983962 -1.856204
H 5.387920 -0.406308 2.263311
H 6.767674 0.526307 1.649741
H 5.210661 1.322804 1.921801

16 Energy:-1118.36053027
C -2.829495 2.193773 -2.311996
C -1.450335 2.165979 -2.496595
C -0.694465 1.290176 -1.730368
C -1.298620 0.450636 -0.775147
C -2.707810 0.468011 -0.622233
C -3.458363 1.365551 -1.405760

N -0.507956 -0.437496 -0.012936
C -1.046176 -1.349284 0.900201
C -2.516709 -1.353394 1.038276
C -3.336595 -0.457298 0.320590
C -3.079075 -2.269353 1.943010
C -4.450016 -2.308006 2.143361
C -5.274445 -1.419276 1.437031
C -4.728336 -0.509992 0.542102
O -0.318954 -2.091461 1.560453
C 0.932014 -0.312443 -0.026170
C 1.726494 -1.370489 -0.460210
C 3.118264 -1.261245 -0.424711
C 3.753961 -0.104841 0.049304
C 2.928629 0.942642 0.496377
C 1.540197 0.842962 0.472945
F -3.575617 3.048049 -3.050782
C 5.284763 0.048651 0.098280
C 6.015733 -1.200793 -0.428389
C 5.706586 1.257807 -0.770537
C 5.730887 0.288900 1.560536
H -0.984542 2.813849 -3.231223
H 0.376780 1.253420 -1.877534
H -4.536626 1.419334 -1.321710
H -2.410827 -2.936851 2.475437
H -4.882451 -3.018214 2.841762
H -6.349966 -1.439250 1.588628
H -5.392874 0.163192 0.012478
H 1.260234 -2.278049 -0.828861
H 3.704335 -2.103446 -0.774133
H 3.371780 1.855775 0.882601
H 0.927627 1.661515 0.839276
H 7.098384 -1.041745 -0.373883
H 5.782092 -2.091459 0.165845
H 5.764646 -1.410401 -1.474405
H 6.795377 1.384765 -0.739179
H 5.251897 2.190381 -0.420107
H 5.411776 1.111277 -1.816123
H 5.450711 -0.556271 2.199653
H 6.820242 0.405556 1.608853
H 5.280040 1.193403 1.982152

17 Energy:-1118.36148199
C -2.809260 2.084828 -2.415823
C -1.423395 2.094694 -2.543267
C -0.673305 1.271157 -1.715454
C -1.292711 0.445136 -0.758853
C -2.706508 0.430411 -0.655299
C -3.450476 1.274232 -1.501924

N -0.510841 -0.387521 0.069217
C -1.052014 -1.263041 1.011901
C -2.525573 -1.296174 1.106875
C -3.342957 -0.465813 0.310672
C -3.095077 -2.177156 2.041099
C -4.470963 -2.246186 2.194244
C -5.293176 -1.424332 1.408785
C -4.740012 -0.549725 0.484071
O -0.324016 -1.954496 1.724784
C 0.930404 -0.278495 0.045395
C 1.702869 -1.300154 -0.500079
C 3.096054 -1.202371 -0.493163
C 3.752175 -0.092068 0.057658
C 2.947557 0.921062 0.609676
C 1.557910 0.832372 0.614063
F -3.549550 2.887289 -3.216327
C 5.285126 0.047025 0.080131
C 5.992177 -1.157106 -0.570301
C 5.696215 1.323566 -0.692028
C 5.768878 0.157266 1.545993
H -0.947626 2.732763 -3.280091
H 0.404307 1.265890 -1.813171
H -4.532202 1.302096 -1.459243
H -2.428412 -2.794280 2.633072
H -4.909026 -2.929115 2.915964
H -6.372677 -1.469340 1.521892
H -5.403130 0.071750 -0.107020
H 1.217823 -2.170919 -0.930091
H 3.666726 -2.015256 -0.927101
H 3.408420 1.797879 1.054820
H 0.959063 1.623121 1.056550
H 7.077082 -1.009643 -0.530366
H 5.766639 -2.094073 -0.048572
H 5.713084 -1.274806 -1.623583
H 6.786610 1.439269 -0.677749
H 5.259645 2.225976 -0.251326
H 5.374117 1.269880 -1.738449
H 5.498075 -0.738598 2.116415
H 6.859927 0.263384 1.576707
H 5.335220 1.024171 2.055452

18 Energy:-1118.36202852
C -2.788165 1.967923 -2.517983
C -1.399466 2.012501 -2.599050
C -0.656172 1.242858 -1.714930
C -1.286977 0.434587 -0.751077
C -2.702549 0.390346 -0.687614
C -3.439094 1.178916 -1.591834

N -0.513808 -0.339713 0.137708
C -1.059601 -1.174373 1.111663
C -2.534107 -1.234727 1.171093
C -3.346218 -0.472527 0.303958
C -3.110720 -2.077783 2.135270
C -4.488674 -2.175961 2.249329
C -5.305746 -1.422885 1.392760
C -4.745539 -0.586216 0.437643
O -0.333489 -1.814349 1.873258
C 0.928671 -0.247364 0.105866
C 1.678729 -1.221604 -0.546913
C 3.072711 -1.133669 -0.564551
C 3.749799 -0.079573 0.065658
C 2.966588 0.887897 0.720914
C 1.576482 0.809877 0.747585
F -3.521596 2.717261 -3.374471
C 5.284067 0.046700 0.066052
C 5.966195 -1.097944 -0.706822
C 5.690128 1.385419 -0.595654
C 5.799529 0.019850 1.525000
H -0.916254 2.637505 -3.342213
H 0.424199 1.267360 -1.771945
H -4.522008 1.182938 -1.582652
H -2.447871 -2.643299 2.780742
H -4.932307 -2.829353 2.994610
H -6.386840 -1.492093 1.473951
H -5.404887 -0.018230 -0.208914
H 1.175680 -2.047904 -1.040187
H 3.626894 -1.908650 -1.081305
H 3.445476 1.720406 1.227684
H 0.992796 1.565453 1.265390
H 7.052913 -0.961600 -0.678890
H 5.744614 -2.076911 -0.266982
H 5.662452 -1.117878 -1.759700
H 6.781669 1.490605 -0.596167
H 5.272927 2.247364 -0.064433
H 5.344219 1.430291 -1.634863
H 5.532915 -0.922237 2.017609
H 6.891842 0.115482 1.541682
H 5.383787 0.839850 2.119885

19 Energy:-1118.36222523
C -2.769329 1.844383 -2.617210
C -1.380394 1.921955 -2.661585
C -0.643731 1.207301 -1.727150
C -1.281927 0.418831 -0.751938
C -2.697468 0.348286 -0.719136
C -3.427068 1.080305 -1.675031

N -0.516246 -0.298311 0.189115
C -1.067773 -1.092951 1.191862
C -2.542085 -1.173582 1.226617
C -3.347640 -0.476757 0.300162
C -3.125350 -1.975277 2.221534
C -4.503508 -2.095712 2.308836
C -5.314055 -1.408252 1.392937
C -4.747268 -0.613233 0.406634
O -0.345686 -1.687629 1.993161
C 0.927103 -0.221608 0.151637
C 1.657041 -1.132582 -0.607071
C 3.051375 -1.052218 -0.642020
C 3.747801 -0.068224 0.074407
C 2.984124 0.837752 0.832848
C 1.594042 0.768162 0.875436
F -3.496240 2.538686 -3.524195
C 5.282788 0.048194 0.057786
C 5.942238 -1.026126 -0.827559
C 5.686192 1.438764 -0.488585
C 5.824927 -0.113045 1.498213
H -0.892010 2.531303 -3.414306
H 0.436838 1.260504 -1.752307
H -4.509738 1.061570 -1.692245
H -2.467483 -2.491808 2.911651
H -4.952305 -2.716493 3.078499
H -6.395219 -1.496108 1.452203
H -5.401677 -0.095901 -0.285836
H 1.138440 -1.904235 -1.168458
H 3.589990 -1.777013 -1.241460
H 3.478755 1.616160 1.406192
H 1.024763 1.477138 1.469370
H 7.030394 -0.900453 -0.806107
H 5.719578 -2.039413 -0.474532
H 5.621116 -0.948168 -1.872549
H 6.778209 1.538245 -0.499321
H 5.283714 2.251544 0.125054
H 5.322487 1.578212 -1.513229
H 5.560743 -1.094296 1.908858
H 6.918024 -0.025500 1.502241
H 5.426714 0.652148 2.172732

20 Energy:-1118.36206125
C -2.761369 1.710667 -2.716213
C -1.374650 1.827798 -2.730558
C -0.641414 1.174834 -1.749457
C -1.280131 0.405439 -0.759335
C -2.694250 0.303831 -0.750777
C -3.420345 0.972107 -1.754852

N -0.518045 -0.248705 0.229894
C -1.073455 -1.010801 1.256076
C -2.546770 -1.110666 1.274657
C -3.347755 -0.480530 0.297998
C -3.133378 -1.869507 2.300815
C -4.510493 -2.010886 2.371117
C -5.316535 -1.389561 1.405341
C -4.746368 -0.638244 0.387268
O -0.354887 -1.566650 2.087911
C 0.925807 -0.188186 0.185667
C 1.635888 -1.027008 -0.669458
C 3.030435 -0.956063 -0.717625
C 3.747151 -0.053105 0.080574
C 3.003162 0.783616 0.932560
C 1.613205 0.724546 0.987306
F -3.485000 2.342378 -3.670323
C 5.283063 0.048311 0.054602
C 5.919138 -0.944975 -0.936262
C 5.696353 1.479728 -0.363810
C 5.840504 -0.253826 1.466323
H -0.885158 2.421033 -3.495325
H 0.437084 1.262611 -1.749562
H -4.501552 0.924652 -1.793986
H -2.478986 -2.336587 3.028441
H -4.961911 -2.597338 3.165762
H -6.396837 -1.494617 1.450683
H -5.397392 -0.171288 -0.343113
H 1.102187 -1.736876 -1.294733
H 3.552738 -1.624342 -1.392414
H 3.513397 1.500061 1.569448
H 1.059563 1.381026 1.651838
H 7.008933 -0.833940 -0.916792
H 5.688221 -1.984578 -0.677548
H 5.587131 -0.765293 -1.965181
H 6.789261 1.567963 -0.379881
H 5.311409 2.235360 0.328966
H 5.321638 1.719143 -1.365749
H 5.569699 -1.266581 1.786105
H 6.934549 -0.178663 1.464530
H 5.459359 0.448263 2.215231

21 Energy:-1118.36149877
C -2.757788 1.575310 -2.808384
C -1.375896 1.739561 -2.795683
C -0.645216 1.151913 -1.772487
C -1.279846 0.397228 -0.768067
C -2.690853 0.258201 -0.781495
C -3.414579 0.858949 -1.829223

N -0.519682 -0.188925 0.265944
C -1.079737 -0.914820 1.317467
C -2.550945 -1.040966 1.319309
C -3.346781 -0.486285 0.294312
C -3.139824 -1.756234 2.375166
C -4.514395 -1.926593 2.428135
C -5.315361 -1.380276 1.414022
C -4.742766 -0.673371 0.365952
O -0.366021 -1.424398 2.182299
C 0.924599 -0.149377 0.215383
C 1.614895 -0.911712 -0.724027
C 3.009671 -0.853970 -0.782596
C 3.747601 -0.039998 0.088303
C 3.023847 0.721930 1.023903
C 1.634064 0.677216 1.088485
F -3.478800 2.141007 -3.804819
C 5.284211 0.044645 0.052802
C 5.895680 -0.859757 -1.034004
C 5.711589 1.503748 -0.235866
C 5.853754 -0.393989 1.423212
H -0.888220 2.320067 -3.571303
H 0.428669 1.281332 -1.750591
H -4.492891 0.777779 -1.887219
H -2.489301 -2.167198 3.139168
H -4.967668 -2.478367 3.246211
H -6.393598 -1.509009 1.445098
H -5.390006 -0.263914 -0.401292
H 1.066329 -1.553842 -1.407033
H 3.514672 -1.462204 -1.524055
H 3.550546 1.369453 1.718628
H 1.097475 1.277570 1.816472
H 6.987031 -0.764615 -1.017949
H 5.653515 -1.915956 -0.870173
H 5.554544 -0.580930 -2.037534
H 6.805327 1.579967 -0.257380
H 5.344488 2.196030 0.529076
H 5.328610 1.840012 -1.206368
H 5.573324 -1.428735 1.651158
H 6.948611 -0.332147 1.414698
H 5.490314 0.240220 2.238507

22 Energy:-1118.36048434
C -2.755875 1.445882 -2.888496
C -1.382806 1.668942 -2.848269
C -0.655401 1.149887 -1.786700
C -1.281197 0.401063 -0.771746
C -2.686163 0.213472 -0.808762
C -3.407200 0.743800 -1.895901

N -0.522040 -0.111015 0.304134
C -1.089629 -0.792771 1.383991
C -2.556357 -0.960547 1.363099
C -3.343807 -0.497397 0.288158
C -3.147279 -1.634343 2.445014
C -4.515706 -1.852673 2.474131
C -5.308125 -1.398706 1.409041
C -4.733344 -0.733573 0.335163
O -0.385367 -1.240661 2.289402
C 0.922595 -0.100083 0.245664
C 1.591960 -0.787667 -0.764950
C 2.986791 -0.749353 -0.834097
C 3.748499 -0.027658 0.095311
C 3.047015 0.661927 1.101190
C 1.657555 0.637270 1.176772
F -3.473855 1.942689 -3.922887
C 5.285738 0.034259 0.049234
C 5.869886 -0.781952 -1.119307
C 5.735718 1.505673 -0.116590
C 5.861842 -0.531183 1.369482
H -0.899765 2.243850 -3.630918
H 0.410127 1.330957 -1.742603
H -4.480472 0.621713 -1.971942
H -2.503144 -1.976392 3.247348
H -4.970599 -2.371806 3.312418
H -6.381282 -1.567022 1.420409
H -5.373870 -0.396071 -0.471671
H 1.028023 -1.359972 -1.495609
H 3.472380 -1.299438 -1.631849
H 3.591657 1.238426 1.843100
H 1.141094 1.183513 1.959028
H 6.962872 -0.706807 -1.108087
H 5.610773 -1.844220 -1.045219
H 5.523177 -0.410892 -2.090556
H 6.830376 1.565459 -0.144158
H 5.388444 2.134797 0.709732
H 5.348833 1.931228 -1.049786
H 5.566221 -1.577214 1.510014
H 6.957448 -0.486337 1.353577
H 5.517452 0.035494 2.240845

23 Energy:-1118.35886609
C -2.754848 1.322639 -2.957768
C -1.395962 1.615650 -2.890715
C -0.673015 1.167263 -1.794944
C -1.284540 0.415265 -0.772902
C -2.679399 0.169431 -0.832937
C -3.396979 0.626969 -1.955265

N -0.525524 -0.018275 0.340224
C -1.101932 -0.659150 1.444334
C -2.561620 -0.876981 1.401234
C -3.337115 -0.513322 0.281135
C -3.154258 -1.511344 2.506054
C -4.512386 -1.787937 2.512382
C -5.292201 -1.435137 1.400732
C -4.715607 -0.809444 0.304349
O -0.410084 -1.042209 2.387887
C 0.919207 -0.043626 0.273242
C 1.569477 -0.658203 -0.796507
C 2.964137 -0.644433 -0.874381
C 3.748986 -0.014933 0.101193
C 3.068594 0.607860 1.163132
C 1.679730 0.606887 1.249331
F -3.468757 1.748292 -4.025848
C 5.286639 0.018792 0.045960
C 5.843788 -0.713347 -1.189553
C 5.767116 1.488991 -0.006231
C 5.860622 -0.662171 1.311508
H -0.921612 2.191887 -3.677668
H 0.379351 1.408292 -1.728979
H -4.462242 0.456667 -2.048509
H -2.519578 -1.778100 3.343610
H -4.968623 -2.275862 3.368489
H -6.356749 -1.651477 1.393392
H -5.346028 -0.550516 -0.538645
H 0.992554 -1.162339 -1.565708
H 3.430554 -1.140106 -1.718014
H 3.629868 1.115481 1.942065
H 1.183881 1.104251 2.074890
H 6.938181 -0.661714 -1.183657
H 5.562760 -1.772604 -1.197676
H 5.496560 -0.259020 -2.124564
H 6.862610 1.527905 -0.038254
H 5.439117 2.057511 0.870345
H 5.383116 1.995612 -0.899269
H 5.543555 -1.709693 1.370915
H 6.956828 -0.639245 1.289268
H 5.535021 -0.159908 2.228478

24 Energy:-1118.35649963
C -2.756186 1.205754 -3.017620
C -1.417445 1.575911 -2.927458
C -0.699727 1.198112 -1.802536
C -1.290833 0.435992 -0.775315
C -2.671251 0.126263 -0.854414
C -3.384893 0.510493 -2.006832

N -0.529899 0.082495 0.368192
C -1.113743 -0.529002 1.489941
C -2.564707 -0.797730 1.430685
C -3.326615 -0.532175 0.275086
C -3.157852 -1.394881 2.556002
C -4.502691 -1.730219 2.546680
C -5.267863 -1.477119 1.398375
C -4.690623 -0.888921 0.281696
O -0.434073 -0.855197 2.462811
C 0.914560 0.015802 0.293278
C 1.550866 -0.524066 -0.825570
C 2.945006 -0.538023 -0.908461
C 3.749061 -0.000827 0.105313
C 3.085442 0.559871 1.210966
C 1.697333 0.584181 1.304988
F -3.465217 1.559454 -4.114465
C 5.286653 0.000027 0.045103
C 5.821772 -0.654671 -1.242490
C 5.802824 1.458108 0.096396
C 5.847782 -0.783912 1.255591
H -0.956409 2.158502 -3.717604
H 0.334578 1.503166 -1.717111
H -4.438795 0.286865 -2.114732
H -2.534108 -1.587417 3.421550
H -4.959449 -2.188235 3.418875
H -6.321287 -1.741663 1.378303
H -5.309212 -0.706928 -0.589697
H 0.965543 -0.961896 -1.627522
H 3.395094 -0.981058 -1.789497
H 3.659758 1.001720 2.020070
H 1.220102 1.038391 2.164156
H 6.917166 -0.631458 -1.237850
H 5.513603 -1.703092 -1.325795
H 5.483712 -0.125368 -2.140711
H 6.898870 1.472991 0.062268
H 5.491096 1.969926 1.012941
H 5.428674 2.036599 -0.756244
H 5.506207 -1.825319 1.240513
H 6.944204 -0.785055 1.230572
H 5.536167 -0.341843 2.207729

25 Energy:-1118.35323519
C -2.752150 1.105511 -3.064414
C -1.440538 1.558349 -2.951579
C -0.731476 1.248799 -1.801104
C -1.298005 0.469724 -0.772258
C -2.657724 0.087955 -0.871556
C -3.364485 0.401884 -2.049680

N -0.536408 0.197839 0.397231
C -1.133495 -0.374912 1.537640
C -2.570152 -0.711722 1.456963
C -3.309250 -0.556200 0.267840
C -3.165398 -1.276688 2.597853
C -4.488436 -1.689266 2.568979
C -5.228593 -1.549219 1.385617
C -4.649442 -0.992576 0.253695
O -0.474905 -0.626225 2.545963
C 0.906966 0.080885 0.315846
C 1.531166 -0.388781 -0.842792
C 2.923859 -0.436598 -0.931900
C 3.746941 0.010240 0.109511
C 3.099906 0.516767 1.249665
C 1.713157 0.571376 1.352890
F -3.453406 1.390987 -4.185417
C 5.283480 -0.026014 0.041782
C 5.796271 -0.617022 -1.285108
C 5.838467 1.412257 0.177082
C 5.827951 -0.894969 1.200709
H -0.996663 2.153372 -3.742253
H 0.279020 1.620903 -1.695341
H -4.403144 0.121547 -2.171795
H -2.560382 -1.384906 3.490801
H -4.946744 -2.121876 3.453243
H -6.263585 -1.877219 1.350032
H -5.247524 -0.898167 -0.645498
H 0.939028 -0.762705 -1.670657
H 3.357098 -0.832384 -1.843428
H 3.686445 0.896501 2.081395
H 1.256179 0.989954 2.238756
H 6.891923 -0.624576 -1.284334
H 5.458355 -1.649554 -1.429439
H 5.470481 -0.025726 -2.148505
H 6.934461 1.400559 0.139234
H 5.542569 1.876386 1.123751
H 5.477265 2.050043 -0.638044
H 5.457795 -1.924010 1.126395
H 6.923837 -0.924535 1.169452
H 5.532479 -0.501437 2.178934

26 Energy:-1118.34914562
C -2.751115 1.011780 -3.107608
C -1.477795 1.560248 -2.977335
C -0.777523 1.327232 -1.803927
C -1.309209 0.529015 -0.771191
C -2.636316 0.055189 -0.888788
C -3.336996 0.291978 -2.088629

N -0.547577 0.349162 0.420467
C -1.168264 -0.148821 1.588268
C -2.573626 -0.599114 1.480663
C -3.273403 -0.587737 0.258800
C -3.167837 -1.136630 2.635311
C -4.447341 -1.668322 2.584809
C -5.143526 -1.677571 1.367230
C -4.566511 -1.145669 0.222043
O -0.550853 -0.264293 2.645269
C 0.893159 0.175911 0.337403
C 1.506724 -0.234455 -0.851925
C 2.896033 -0.329291 -0.947487
C 3.738195 0.021370 0.114741
C 3.108575 0.481506 1.282490
C 1.724504 0.581779 1.394787
F -3.445020 1.223294 -4.248973
C 5.271961 -0.067447 0.039833
C 5.760645 -0.590914 -1.324021
C 5.881337 1.337040 0.265375
C 5.784075 -1.028588 1.139121
H -1.060366 2.170714 -3.770591
H 0.200635 1.774316 -1.680936
H -4.353777 -0.055948 -2.221036
H -2.595946 -1.131630 3.556079
H -4.904174 -2.080697 3.479440
H -6.141929 -2.102416 1.315157
H -5.127859 -1.167624 -0.705270
H 0.908885 -0.542874 -1.701165
H 3.311094 -0.684222 -1.884074
H 3.707405 0.794161 2.133376
H 1.290676 0.971145 2.302802
H 6.855292 -0.638930 -1.328204
H 5.384375 -1.598904 -1.532706
H 5.455475 0.065966 -2.146610
H 6.976062 1.286570 0.222125
H 5.605064 1.749560 1.241414
H 5.542971 2.039512 -0.505124
H 5.376908 -2.036640 0.999218
H 6.878228 -1.095882 1.104560
H 5.501881 -0.688281 2.140978

27 Energy:-1118.34471913
C -2.781729 0.925536 -3.156369
C -1.562619 1.587041 -3.030239
C -0.858770 1.447372 -1.843582
C -1.331750 0.632756 -0.796693
C -2.607322 0.038239 -0.914376
C -3.315247 0.183119 -2.123991

N -0.566588 0.555893 0.407283
C -1.222882 0.189105 1.607806
C -2.554849 -0.449680 1.490428
C -3.197060 -0.620954 0.249148
C -3.125302 -0.974930 2.662162
C -4.317325 -1.682293 2.606594
C -4.949178 -1.877429 1.370037
C -4.398482 -1.353109 0.207721
O -0.685393 0.316388 2.705080
C 0.869180 0.330403 0.333331
C 1.481824 -0.024036 -0.877502
C 2.864444 -0.189354 -0.967366
C 3.713450 0.029957 0.123628
C 3.091771 0.431392 1.315489
C 1.714045 0.604806 1.424650
F -3.480217 1.049468 -4.307378
C 5.240679 -0.136894 0.054654
C 5.715887 -0.586326 -1.340007
C 5.922161 1.212230 0.386726
C 5.687933 -1.201296 1.084851
H -1.193009 2.212333 -3.835545
H 0.075404 1.981287 -1.719174
H -4.299940 -0.249559 -2.251325
H -2.603987 -0.824265 3.600484
H -4.753866 -2.089035 3.513842
H -5.876165 -2.441130 1.316328
H -4.905335 -1.518934 -0.736557
H 0.887179 -0.244888 -1.754286
H 3.268570 -0.496934 -1.925409
H 3.693255 0.642563 2.195497
H 1.294954 0.948604 2.355818
H 6.806251 -0.694655 -1.339604
H 5.287082 -1.553995 -1.624652
H 5.456608 0.144374 -2.114685
H 7.013148 1.105565 0.349839
H 5.655520 1.567855 1.387557
H 5.631856 1.985779 -0.333682
H 5.229039 -2.173068 0.868804
H 6.777263 -1.325270 1.054168
H 5.412707 -0.919483 2.106654

28 Energy:-1118.34082082
C -2.843685 0.810381 -3.211338
C -1.674285 1.560511 -3.120766
C -0.951398 1.511902 -1.936758
C -1.357095 0.700718 -0.861471
C -2.587406 0.012872 -0.945286
C -3.316375 0.067607 -2.148424

N -0.579790 0.707844 0.338786
C -1.260061 0.467125 1.560129
C -2.518993 -0.312287 1.484196
C -3.120108 -0.636055 0.252315
C -3.052567 -0.806762 2.685435
C -4.162520 -1.640116 2.668099
C -4.748321 -1.988520 1.443014
C -4.236552 -1.491143 0.250366
O -0.790860 0.804712 2.642648
C 0.851539 0.453912 0.281755
C 1.493008 0.193558 -0.939192
C 2.870125 -0.025592 -1.003973
C 3.690622 0.034199 0.127182
C 3.042104 0.327222 1.336050
C 1.671015 0.554021 1.422404
F -3.560553 0.847778 -4.356860
C 5.211042 -0.191601 0.086720
C 5.715708 -0.505199 -1.334470
C 5.934617 1.081318 0.587645
C 5.579072 -1.381559 1.004743
H -1.358929 2.184535 -3.949774
H -0.060558 2.120134 -1.835798
H -4.272351 -0.432397 -2.246567
H -2.567679 -0.534928 3.616242
H -4.569278 -2.026600 3.597756
H -5.609267 -2.650508 1.421435
H -4.705626 -1.774470 -0.685815
H 0.926710 0.085893 -1.853702
H 3.291863 -0.248422 -1.977912
H 3.618005 0.405718 2.254249
H 1.239422 0.813201 2.375344
H 6.800235 -0.660328 -1.313965
H 5.258477 -1.416046 -1.737747
H 5.512274 0.316904 -2.030196
H 7.021083 0.931511 0.572948
H 5.646879 1.336185 1.613147
H 5.701474 1.941381 -0.050779
H 5.089651 -2.302662 0.667542
H 6.663012 -1.548803 0.993993
H 5.279513 -1.202599 2.042809

29 Energy:-1118.33776926
C -2.901010 0.656380 -3.260264
C -1.773656 1.470418 -3.216535
C -1.031108 1.506235 -2.042417
C -1.375694 0.713306 -0.934399
C -2.574788 -0.033590 -0.969159
C -3.323130 -0.063876 -2.159627

N -0.583568 0.781906 0.254583
C -1.269288 0.646630 1.489029
C -2.488499 -0.196951 1.476144
C -3.067111 -0.638781 0.269682
C -2.998825 -0.631434 2.709398
C -4.060983 -1.525105 2.750823
C -4.620473 -1.992573 1.553760
C -4.133126 -1.552752 0.327877
O -0.824972 1.116353 2.530971
C 0.846686 0.527665 0.214121
C 1.536832 0.406285 -1.001665
C 2.911038 0.158069 -1.037457
C 3.681142 0.039933 0.122786
C 2.982525 0.177302 1.331990
C 1.614982 0.427388 1.390495
F -3.634915 0.610857 -4.394492
C 5.196592 -0.218734 0.116262
C 5.759288 -0.345149 -1.312061
C 5.921865 0.952775 0.820686
C 5.498331 -1.533845 0.873706
H -1.506225 2.080796 -4.072100
H -0.183860 2.177507 -1.974640
H -4.257199 -0.609546 -2.220088
H -2.533609 -0.267791 3.618854
H -4.449561 -1.866887 3.705415
H -5.442635 -2.701998 1.579571
H -4.582252 -1.926182 -0.586406
H 1.015526 0.434055 -1.946973
H 3.370162 0.057052 -2.014804
H 3.514469 0.101585 2.276582
H 1.152594 0.553807 2.356577
H 6.838568 -0.529247 -1.267568
H 5.303351 -1.179399 -1.857491
H 5.603504 0.570327 -1.894220
H 7.004605 0.777733 0.832446
H 5.591535 1.070648 1.858183
H 5.736111 1.898908 0.299181
H 5.006206 -2.386020 0.390724
H 6.578023 -1.726614 0.885547
H 5.155748 -1.492898 1.913130

30 Energy:-1118.3358048
C -2.957670 0.448335 -3.301102
C -1.854041 1.292908 -3.319726
C -1.090163 1.409792 -2.162954
C -1.388620 0.662516 -1.011711
C -2.578961 -0.103011 -0.981101
C -3.346554 -0.215700 -2.152704

N -0.577549 0.772473 0.160164
C -1.247622 0.748140 1.408092
C -2.467487 -0.091532 1.470969
C -3.050223 -0.626466 0.303977
C -2.967830 -0.432735 2.736611
C -4.023726 -1.327766 2.852626
C -4.585152 -1.889827 1.698181
C -4.108146 -1.541290 0.438907
O -0.781678 1.295609 2.400990
C 0.857118 0.556462 0.132110
C 1.609793 0.625405 -1.048159
C 2.985178 0.372668 -1.050782
C 3.690157 0.050315 0.110611
C 2.924526 -0.024582 1.285638
C 1.556666 0.220333 1.308589
F -3.709043 0.321463 -4.417569
C 5.203618 -0.216966 0.141873
C 5.847543 -0.079234 -1.250637
C 5.885883 0.795217 1.092949
C 5.462780 -1.653424 0.655621
H -1.620982 1.869180 -4.208354
H -0.280829 2.127382 -2.143989
H -4.271544 -0.779881 -2.164608
H -2.499071 0.001345 3.612913
H -4.404822 -1.598137 3.832786
H -5.401587 -2.601272 1.782834
H -4.560773 -1.986050 -0.441095
H 1.148435 0.824348 -2.002164
H 3.497096 0.437145 -2.004648
H 3.400483 -0.283019 2.227662
H 1.042264 0.154992 2.255379
H 6.923469 -0.274208 -1.179543
H 5.428155 -0.795361 -1.966575
H 5.720101 0.929262 -1.660493
H 6.966480 0.611208 1.131752
H 5.497731 0.719540 2.114216
H 5.728276 1.823930 0.748717
H 5.000061 -2.395406 -0.005437
H 6.540236 -1.855375 0.692024
H 5.061217 -1.804180 1.663321

31 Energy:-1118.35303058
C -2.747787 -0.106245 -3.292182
C -1.402793 -0.453794 -3.384473
C -0.682125 -0.622123 -2.212105
C -1.272015 -0.421093 -0.948008
C -2.658539 -0.145093 -0.874995
C -3.377921 0.030573 -2.074028

N -0.495401 -0.629358 0.224895
C -1.099826 -0.679793 1.496881
C -2.558065 -0.449688 1.567215
C -3.318836 -0.117659 0.429092
C -3.157287 -0.491692 2.837817
C -4.505212 -0.205620 2.988923
C -5.267567 0.138644 1.862826
C -4.684806 0.182450 0.603844
O -0.433194 -0.860524 2.514811
C 0.929494 -0.358350 0.212064
C 1.470733 0.624285 -0.621601
C 2.850027 0.835208 -0.672198
C 3.744533 0.058848 0.075360
C 3.183459 -0.950394 0.876190
C 1.811029 -1.173641 0.936437
F -3.461870 0.070192 -4.427468
C 5.268822 0.264341 0.041219
C 5.682551 1.414394 -0.896368
C 5.773484 0.596285 1.465913
C 5.952698 -1.032759 -0.453532
H -0.941423 -0.604565 -4.354346
H 0.356427 -0.919032 -2.276078
H -4.438265 0.249615 -2.064482
H -2.535975 -0.745029 3.689234
H -4.966025 -0.242216 3.971478
H -6.322561 0.373500 1.971906
H -5.299811 0.453034 -0.246900
H 0.820453 1.264831 -1.206877
H 3.215268 1.626423 -1.317041
H 3.829088 -1.597427 1.463089
H 1.424056 -1.976512 1.547990
H 6.772776 1.522879 -0.885113
H 5.379776 1.225513 -1.932656
H 5.251552 2.371880 -0.582368
H 6.861180 0.736629 1.459354
H 5.544434 -0.205013 2.176277
H 5.313899 1.518587 1.839595
H 5.620213 -1.289617 -1.466012
H 7.041342 -0.901904 -0.476326
H 5.733200 -1.884420 0.198907

32 Energy:-1118.35630923
C -2.743245 -0.215120 -3.293854
C -1.377320 -0.472008 -3.369232
C -0.654224 -0.563667 -2.189404
C -1.268346 -0.379565 -0.934658
C -2.670729 -0.185957 -0.876025
C -3.390286 -0.087609 -2.083303

N -0.499174 -0.497884 0.251253
C -1.091840 -0.489614 1.525053
C -2.560817 -0.348545 1.583179
C -3.337817 -0.146516 0.424859
C -3.161232 -0.357114 2.853720
C -4.528011 -0.168639 2.986274
C -5.308996 0.041007 1.839857
C -4.724806 0.052518 0.580974
O -0.407959 -0.562710 2.545657
C 0.931501 -0.276706 0.220447
C 1.484085 0.746678 -0.551366
C 2.868822 0.919144 -0.610931
C 3.747575 0.071183 0.075782
C 3.169264 -0.968913 0.824728
C 1.791837 -1.154670 0.890200
F -3.458749 -0.113236 -4.437730
C 5.277034 0.234327 0.032682
C 5.713554 1.427681 -0.838045
C 5.809091 0.462667 1.467793
C 5.915335 -1.049066 -0.550735
H -0.898763 -0.609624 -4.332738
H 0.403048 -0.786395 -2.241731
H -4.461526 0.069966 -2.086753
H -2.525999 -0.509435 3.718939
H -4.990009 -0.179445 3.968902
H -6.380184 0.195828 1.933058
H -5.355909 0.216957 -0.284908
H 0.838745 1.434142 -1.088304
H 3.251383 1.737358 -1.210336
H 3.804247 -1.664220 1.366024
H 1.382622 -1.976015 1.464600
H 6.806619 1.502801 -0.835812
H 5.392317 1.312548 -1.879559
H 5.316008 2.376176 -0.459336
H 6.900101 0.573493 1.453866
H 5.567103 -0.374865 2.130416
H 5.380449 1.371957 1.904693
H 5.562975 -1.232743 -1.572373
H 7.007007 -0.948810 -0.580672
H 5.678409 -1.932791 0.051009

33 Energy:-1118.35874732
C -2.731848 -0.342492 -3.291833
C -1.352659 -0.517894 -3.354528
C -0.630563 -0.533096 -2.170113
C -1.264271 -0.357996 -0.924431
C -2.675560 -0.233159 -0.876144
C -3.392461 -0.213410 -2.088393

N -0.504107 -0.389549 0.269103
C -1.091239 -0.337651 1.540028
C -2.564580 -0.257589 1.591204
C -3.347242 -0.167653 0.421801
C -3.167617 -0.224581 2.859925
C -4.543206 -0.105894 2.981134
C -5.330888 -0.011110 1.823995
C -4.744155 -0.040820 0.566670
O -0.399259 -0.334170 2.558232
C 0.930726 -0.210891 0.227134
C 1.494400 0.856909 -0.470600
C 2.882349 1.000372 -0.536677
C 3.746589 0.084856 0.078756
C 3.153342 -0.991368 0.763300
C 1.772520 -1.148901 0.832243
F -3.445829 -0.317107 -4.441281
C 5.279257 0.214072 0.026403
C 5.735261 1.455555 -0.763372
C 5.834497 0.326629 1.466475
C 5.877497 -1.039566 -0.655857
H -0.861304 -0.649252 -4.312467
H 0.439181 -0.687464 -2.212183
H -4.470321 -0.110898 -2.102010
H -2.527341 -0.291231 3.732346
H -5.007333 -0.083918 3.962582
H -6.409597 0.086323 1.907746
H -5.381117 0.034304 -0.307245
H 0.854303 1.589946 -0.951829
H 3.279620 1.849064 -1.081468
H 3.778565 -1.733717 1.250695
H 1.345415 -1.994128 1.359601
H 6.829821 1.503471 -0.770672
H 5.399030 1.422964 -1.806009
H 5.366631 2.384133 -0.312869
H 6.927595 0.412271 1.444674
H 5.580637 -0.550002 2.071509
H 5.433929 1.212289 1.972935
H 5.508694 -1.140506 -1.683199
H 6.970941 -0.964442 -0.693305
H 5.624889 -1.958150 -0.115848

34 Energy:-1118.36041406
C -2.720985 -0.484907 -3.283781
C -1.334157 -0.586516 -3.339864
C -0.614221 -0.523715 -2.155328
C -1.261922 -0.348860 -0.917408
C -2.677625 -0.283488 -0.874072
C -3.391258 -0.344438 -2.086552

N -0.509150 -0.297547 0.277106
C -1.091877 -0.209008 1.544536
C -2.567058 -0.173013 1.593277
C -3.351494 -0.182332 0.420803
C -3.172215 -0.093319 2.858686
C -4.551698 -0.024957 2.974854
C -5.341444 -0.030755 1.815232
C -4.752769 -0.107640 0.560827
O -0.394923 -0.145624 2.557768
C 0.928626 -0.153947 0.226142
C 1.505143 0.958579 -0.383888
C 2.895382 1.078266 -0.453314
C 3.744412 0.097506 0.077839
C 3.135819 -1.018736 0.680372
C 1.752378 -1.152792 0.750119
F -3.432392 -0.539030 -4.434080
C 5.279180 0.200301 0.022834
C 5.755073 1.489778 -0.672656
C 5.845529 0.190739 1.463015
C 5.847727 -1.008339 -0.758787
H -0.834444 -0.719379 -4.293287
H 0.463009 -0.615643 -2.190681
H -4.472698 -0.292505 -2.105661
H -2.530402 -0.084864 3.732567
H -5.017366 0.034470 3.954023
H -6.423418 0.025345 1.894769
H -5.391563 -0.109768 -0.315027
H 0.872238 1.737752 -0.798285
H 3.307266 1.959716 -0.931040
H 3.750321 -1.808282 1.102728
H 1.308687 -2.026651 1.215289
H 6.850297 1.516209 -0.685447
H 5.411133 1.544464 -1.711849
H 5.408822 2.387987 -0.148821
H 6.939964 0.256212 1.439237
H 5.577771 -0.724508 2.001311
H 5.466342 1.042789 2.038836
H 5.470712 -1.022249 -1.788011
H 6.942174 -0.951838 -0.798631
H 5.580268 -1.960915 -0.289542

35 Energy:-1118.36146025
C -2.704526 -0.634310 -3.268224
C -1.314324 -0.671102 -3.320698
C -0.599816 -0.530711 -2.139224
C -1.259199 -0.346924 -0.909333
C -2.676473 -0.334714 -0.868800
C -3.383853 -0.475739 -2.077991

N -0.514544 -0.211003 0.281450
C -1.096509 -0.076332 1.542405
C -2.572319 -0.084784 1.590225
C -3.354344 -0.193656 0.420575
C -3.181624 0.042470 2.849537
C -4.562928 0.061432 2.963230
C -5.350385 -0.044325 1.806861
C -4.757682 -0.169301 0.558215
O -0.398508 0.054253 2.548654
C 0.925648 -0.096501 0.226317
C 1.518456 1.054838 -0.286900
C 2.910414 1.152582 -0.357793
C 3.742248 0.112196 0.079459
C 3.116144 -1.039659 0.590164
C 1.730448 -1.151577 0.660061
F -3.410365 -0.766242 -4.415864
C 5.278497 0.188660 0.019075
C 5.775757 1.522240 -0.570177
C 5.855240 0.046785 1.448064
C 5.815880 -0.961042 -0.866544
H -0.807487 -0.811553 -4.269270
H 0.481029 -0.566534 -2.170010
H -4.466502 -0.468828 -2.100681
H -2.541501 0.125882 3.720786
H -5.031803 0.158426 3.937866
H -6.433847 -0.028648 1.884436
H -5.394859 -0.248648 -0.315265
H 0.895927 1.877049 -0.627742
H 3.337986 2.063411 -0.760744
H 3.717897 -1.873299 0.939862
H 1.270593 -2.052522 1.053918
H 6.871150 1.527268 -0.591345
H 5.424791 1.671265 -1.597704
H 5.452817 2.379889 0.030779
H 6.950580 0.091828 1.419685
H 5.572365 -0.904840 1.910039
H 5.498303 0.854940 2.096751
H 5.429537 -0.881787 -1.889326
H 6.910798 -0.921982 -0.913105
H 5.534084 -1.944302 -0.475516

36 Energy:-1118.36201614
C -2.688365 -0.800729 -3.243370
C -1.297810 -0.781159 -3.297252
C -0.589354 -0.560732 -2.124214
C -1.256838 -0.356696 -0.902276
C -2.673616 -0.390208 -0.860500
C -3.374443 -0.613888 -2.060867

N -0.519354 -0.136964 0.278947
C -1.102165 0.048215 1.531291
C -2.577517 0.004357 1.580850
C -3.355251 -0.200421 0.420806
C -3.191052 0.185969 2.831270
C -4.572311 0.166253 2.945946
C -5.355468 -0.035328 1.799443
C -4.758626 -0.215306 0.559493
O -0.405067 0.242862 2.527900
C 0.922435 -0.044249 0.222555
C 1.532393 1.139842 -0.183606
C 2.925621 1.219763 -0.251566
C 3.740189 0.128178 0.081637
C 3.096235 -1.055292 0.487028
C 1.708780 -1.148294 0.555714
F -3.388094 -1.013283 -4.382753
C 5.277343 0.182263 0.017368
C 5.796611 1.558485 -0.440061
C 5.860388 -0.112199 1.420257
C 5.786412 -0.883343 -0.982819
H -0.786209 -0.938262 -4.240645
H 0.492195 -0.547856 -2.154672
H -4.456649 -0.647316 -2.083254
H -2.554136 0.341331 3.695027
H -5.044464 0.306223 3.913755
H -6.438861 -0.051399 1.877903
H -5.392509 -0.368312 -0.306587
H 0.921703 1.999473 -0.444107
H 3.368419 2.156414 -0.569919
H 3.684676 -1.927772 0.755096
H 1.233390 -2.072747 0.869517
H 6.891784 1.545515 -0.467833
H 5.443757 1.815155 -1.445434
H 5.492738 2.358010 0.245080
H 6.956119 -0.082630 1.387696
H 5.564445 -1.100739 1.786588
H 5.521559 0.632336 2.149736
H 5.396281 -0.694297 -1.989632
H 6.881638 -0.861738 -1.033365
H 5.486244 -1.894937 -0.690012

37 Energy:-1118.36220704
C -2.688892 -0.958994 -3.214500
C -1.300298 -0.891702 -3.280458
C -0.592564 -0.597366 -2.123258
C -1.259284 -0.370599 -0.905063
C -2.674150 -0.442554 -0.852440
C -3.374188 -0.742237 -2.036544

N -0.522818 -0.073359 0.259038
C -1.102669 0.159346 1.504268
C -2.576655 0.086901 1.563635
C -3.353954 -0.204982 0.421875
C -3.189062 0.320719 2.805853
C -4.568668 0.268824 2.930279
C -5.351265 -0.020082 1.802217
C -4.755548 -0.252823 0.570529
O -0.404086 0.410589 2.487129
C 0.919867 -0.000289 0.199734
C 1.548090 1.207835 -0.089960
C 2.942428 1.272602 -0.148111
C 3.739563 0.141561 0.079571
C 3.077196 -1.065218 0.369809
C 1.688168 -1.142471 0.430689
F -3.387706 -1.245671 -4.338147
C 5.277585 0.178470 0.023268
C 5.818111 1.583246 -0.304474
C 5.851871 -0.252323 1.394177
C 5.774554 -0.797671 -1.069922
H -0.789843 -1.067314 -4.221210
H 0.487478 -0.542574 -2.163076
H -4.454994 -0.809439 -2.050224
H -2.552545 0.541269 3.655630
H -5.039998 0.450114 3.891610
H -6.433322 -0.062974 1.888611
H -5.388815 -0.473109 -0.281395
H 0.950329 2.097056 -0.267542
H 3.400453 2.228536 -0.374284
H 3.651920 -1.967821 0.554989
H 1.198087 -2.084437 0.658722
H 6.913007 1.556531 -0.333000
H 5.470807 1.937269 -1.281837
H 5.525057 2.320366 0.451689
H 6.948009 -0.236212 1.366296
H 5.540449 -1.265846 1.667783
H 5.521789 0.427127 2.188303
H 5.390788 -0.510746 -2.055838
H 6.870145 -0.787541 -1.115767
H 5.458768 -1.827458 -0.872496

12.2.5 Potential scan of compound 4b excited state (B3LYP/6-31G* PCM CH_2Cl_2)

D(4,7,16,17)
8 -1118.35426335 89.7321
25 -1118.34757909 99.732
30 -1118.34724539 109.7322

35 -1118.34684541 119.7323
41 -1118.34530216 129.7321
74 -1118.33667721 139.7323
83 -1118.31746675 149.732
89 -1118.31399405 159.7323
94 -1118.31213834 169.7322
98 -1118.31063726 179.7322
103 -1118.30952388 -170.2679
107 -1118.30847019 -160.268
112 -1118.30756353 -150.268
117 -1118.30680329 -140.268
123 -1118.30616419 -130.268
128 -1118.30569227 -120.268
135 -1118.30742515 -110.268
167 -1118.34773491 -100.2679
185 -1118.35420583 -90.268
195 -1118.34759786 -80.2678
200 -1118.3470889 -70.2677
205 -1118.3465484 -60.2678
210 -1118.34487532 -50.2679
231 -1118.32387 -40.2678
238 -1118.31632964 -30.2677
244 -1118.31425907 -20.2677
248 -1118.31256204 -10.2679
253 -1118.31093582 -0.2678
258 -1118.30962756 9.7322
262 -1118.3084268 19.7321
266 -1118.30721663 29.732
270 -1118.30599352 39.732
276 -1118.3047836 49.732
282 -1118.30392108 59.732
288 -1118.30411319 69.732
319 -1118.3473684 79.7321
342 -1118.35426345 89.7321

Progress of structural optimization and emission calculation

-1118.362218 -1118.219854 320.05 nm
-1118.357476 -1118.226864 348.84 nm
-1118.354813 -1118.227664 358.35 nm
-1118.354388 -1118.227768 359.84 nm
-1118.354288 -1118.227816 360.26 nm
-1118.354287 -1118.227833 360.32 nm
-1118.35436 -1118.227844 360.14 nm
-1118.354291 -1118.227847 360.34 nm
-1118.354263 -1118.227847 360.42 nm
-1118.353923 -1118.227712 361.01 nm
-1118.354146 -1118.227975 361.12 nm

-1118.353851	-1118.228109	362.36	nm
-1118.353133	-1118.228283	364.94	nm
-1118.352215	-1118.228428	368.08	nm
-1118.34756	-1118.228538	382.82	nm
-1118.352327	-1118.228593	368.24	nm
-1118.350104	-1118.228835	375.72	nm
-1118.349672	-1118.228858	377.14	nm
-1118.345093	-1118.228783	391.74	nm
-1118.348413	-1118.228906	381.26	nm
-1118.348895	-1118.228901	379.72	nm
-1118.347703	-1118.228919	383.58	nm
-1118.347806	-1118.228919	383.25	nm
-1118.347987	-1118.228918	382.66	nm
-1118.347608	-1118.22892	383.89	nm
-1118.347579	-1118.22892	383.99	nm
-1118.347662	-1118.229995	387.22	nm
-1118.347313	-1118.230206	389.08	nm
-1118.34727	-1118.230222	389.27	nm
-1118.347195	-1118.230224	389.53	nm
-1118.347245	-1118.230224	389.36	nm
-1118.346852	-1118.23101	393.32	nm
-1118.347304	-1118.231217	392.49	nm
-1118.346342	-1118.231222	395.79	nm
-1118.346873	-1118.231231	394	nm
-1118.346845	-1118.231231	394.1	nm
-1118.345831	-1118.231393	398.15	nm
-1118.345796	-1118.231649	399.16	nm
-1118.345451	-1118.231665	400.43	nm
-1118.345321	-1118.231668	400.9	nm
-1118.345303	-1118.231668	400.96	nm
-1118.345302	-1118.231668	400.97	nm
-1118.343636	-1118.231033	404.64	nm
-1118.342557	-1118.23144	410.05	nm
-1118.341384	-1118.231499	414.65	nm
-1118.339494	-1118.231548	422.1	nm
-1118.339018	-1118.23156	424.01	nm
-1118.338595	-1118.231567	425.71	nm
-1118.338304	-1118.231569	426.88	nm
-1118.337866	-1118.23157	428.65	nm
-1118.172965	-1118.076905	474.32	nm
-1118.331679	-1118.231265	453.76	nm
-1118.337672	-1118.23157	429.43	nm
-1118.335728	-1118.231544	437.34	nm
-1118.337699	-1118.231569	429.32	nm
-1118.335178	-1118.231554	439.7	nm
-1118.336554	-1118.231572	434.01	nm
-1118.337901	-1118.23157	428.5	nm
-1118.33601	-1118.231545	436.16	nm

-1118.336975	-1118.231571	432.27	nm
-1118.336989	-1118.231573	432.22	nm
-1118.337513	-1118.231571	430.08	nm
-1118.334438	-1118.231513	442.69	nm
-1118.336586	-1118.231569	433.87	nm
-1118.336744	-1118.231573	433.23	nm
-1118.33782	-1118.23157	428.83	nm
-1118.336037	-1118.231559	436.1	nm
-1118.337123	-1118.231572	431.67	nm
-1118.336918	-1118.231573	432.52	nm
-1118.337714	-1118.231571	429.26	nm
-1118.331465	-1118.23136	455.15	nm
-1118.335784	-1118.231563	437.18	nm
-1118.336555	-1118.231573	434.01	nm
-1118.336729	-1118.231573	433.3	nm
-1118.336677	-1118.231573	433.51	nm
-1118.335689	-1118.231632	437.87	nm
-1118.324257	-1118.233132	500.01	nm
-1118.320654	-1118.233598	523.38	nm
-1118.318214	-1118.233755	539.47	nm
-1118.319188	-1118.233735	533.2	nm
-1118.314716	-1118.233741	562.69	nm
-1118.317344	-1118.233786	545.29	nm
-1118.317474	-1118.233794	544.49	nm
-1118.317467	-1118.233794	544.54	nm
-1118.318083	-1118.235942	554.7	nm
-1118.313536	-1118.236368	590.45	nm
-1118.314254	-1118.236391	585.17	nm
-1118.313943	-1118.236394	587.55	nm
-1118.313998	-1118.236395	587.13	nm
-1118.313994	-1118.236395	587.16	nm
-1118.314676	-1118.238096	594.97	nm
-1118.312163	-1118.238478	618.35	nm
-1118.3123	-1118.238487	617.28	nm
-1118.312089	-1118.238488	619.06	nm
-1118.312138	-1118.238488	618.65	nm
-1118.312695	-1118.239424	621.84	nm
-1118.310439	-1118.239767	644.71	nm
-1118.310659	-1118.239772	642.76	nm
-1118.310637	-1118.239772	642.96	nm
-1118.310896	-1118.239675	639.75	nm
-1118.309336	-1118.239971	656.87	nm
-1118.309521	-1118.239974	655.15	nm
-1118.309532	-1118.239974	655.04	nm
-1118.309524	-1118.239974	655.12	nm
-1118.309334	-1118.238619	644.32	nm
-1118.308368	-1118.238895	655.85	nm
-1118.308452	-1118.238896	655.07	nm

-1118.30847	-1118.238896	654.89	nm
-1118.307737	-1118.236201	636.93	nm
-1118.30744	-1118.236494	642.22	nm
-1118.307551	-1118.236497	641.25	nm
-1118.307553	-1118.236498	641.24	nm
-1118.307564	-1118.236498	641.14	nm
-1118.3063	-1118.232542	617.74	nm
-1118.306666	-1118.232883	617.53	nm
-1118.306776	-1118.232885	616.62	nm
-1118.306811	-1118.232885	616.34	nm
-1118.306803	-1118.232885	616.4	nm
-1118.305104	-1118.227904	590.2	nm
-1118.306111	-1118.228341	585.87	nm
-1118.306223	-1118.228347	585.07	nm
-1118.306202	-1118.228347	585.24	nm
-1118.306184	-1118.228347	585.37	nm
-1118.306164	-1118.228347	585.52	nm
-1118.304227	-1118.222762	559.3	nm
-1118.305336	-1118.223273	555.22	nm
-1118.305555	-1118.223282	553.8	nm
-1118.305686	-1118.223283	552.93	nm
-1118.305692	-1118.223283	552.89	nm
-1118.303691	-1118.217437	528.24	nm
-1118.305373	-1118.218077	521.94	nm
-1118.306197	-1118.21812	517.32	nm
-1118.306933	-1118.218138	513.13	nm
-1118.307164	-1118.21814	511.81	nm
-1118.30738	-1118.218141	510.58	nm
-1118.307425	-1118.218141	510.32	nm
-1118.305599	-1118.212836	491.18	nm
-1118.31015	-1118.214368	475.7	nm
-1118.316866	-1118.214603	445.55	nm
-1118.324902	-1118.218407	427.84	nm
-1118.336168	-1118.222073	399.35	nm
-1118.346105	-1118.225327	377.25	nm
-1118.348166	-1118.226381	374.13	nm
-1118.348352	-1118.226736	374.65	nm
-1118.349815	-1118.22689	370.66	nm
-1118.350409	-1118.227014	369.25	nm
-1118.352691	-1118.227283	363.32	nm
-1118.353425	-1118.227464	361.72	nm
-1118.354501	-1118.227747	359.46	nm
-1118.353802	-1118.227743	361.45	nm
-1118.352727	-1118.228048	365.45	nm
-1118.35263	-1118.227774	364.93	nm
-1118.352671	-1118.228435	366.75	nm
-1118.351415	-1118.228204	369.8	nm
-1118.348069	-1118.228786	381.98	nm

-1118.35186	-1118.228321	368.82	nm
-1118.350352	-1118.228893	375.13	nm
-1118.351059	-1118.228664	372.27	nm
-1118.347545	-1118.228942	384.17	nm
-1118.351966	-1118.228395	368.72	nm
-1118.349306	-1118.228953	378.58	nm
-1118.349468	-1118.22894	378.03	nm
-1118.34723	-1118.228975	385.3	nm
-1118.348981	-1118.228958	379.62	nm
-1118.347846	-1118.228979	383.31	nm
-1118.34817	-1118.228977	382.26	nm
-1118.347741	-1118.228979	383.65	nm
-1118.347735	-1118.228979	383.67	nm
-1118.34691	-1118.227377	381.18	nm
-1118.348901	-1118.227677	375.86	nm
-1118.349931	-1118.227719	372.82	nm
-1118.350924	-1118.22774	369.88	nm
-1118.352671	-1118.227777	364.82	nm
-1118.353788	-1118.227754	361.52	nm
-1118.353391	-1118.227803	362.8	nm
-1118.35227	-1118.227769	365.97	nm
-1118.354533	-1118.227752	359.39	nm
-1118.35411	-1118.227824	360.8	nm
-1118.352251	-1118.227768	366.02	nm
-1118.354372	-1118.227818	360.03	nm
-1118.35425	-1118.227826	360.4	nm
-1118.354248	-1118.227819	360.39	nm
-1118.354113	-1118.227834	360.81	nm
-1118.354299	-1118.227832	360.28	nm
-1118.354175	-1118.227834	360.64	nm
-1118.354206	-1118.227834	360.55	nm
-1118.353815	-1118.227544	360.84	nm
-1118.352802	-1118.228365	366.16	nm
-1118.351838	-1118.228618	369.77	nm
-1118.349742	-1118.228778	376.67	nm
-1118.349026	-1118.228827	379.06	nm
-1118.348165	-1118.228872	381.95	nm
-1118.347638	-1118.228889	383.7	nm
-1118.347536	-1118.22889	384.03	nm
-1118.347609	-1118.22889	383.79	nm
-1118.347598	-1118.22889	383.83	nm
-1118.347786	-1118.229965	386.72	nm
-1118.345929	-1118.23016	393.57	nm
-1118.347809	-1118.230186	387.37	nm
-1118.347134	-1118.230199	389.65	nm
-1118.347089	-1118.230199	389.8	nm
-1118.346769	-1118.230977	393.49	nm
-1118.346393	-1118.231191	395.51	nm

-1118.3466	-1118.231198	394.82	nm
-1118.346572	-1118.231202	394.93	nm
-1118.346548	-1118.231202	395.01	nm
-1118.345501	-1118.231335	399.1	nm
-1118.345106	-1118.231607	401.44	nm
-1118.344892	-1118.231615	402.23	nm
-1118.344907	-1118.231615	402.18	nm
-1118.344875	-1118.231615	402.29	nm
-1118.343031	-1118.230965	406.58	nm
-1118.341711	-1118.231436	413.18	nm
-1118.340036	-1118.231533	419.92	nm
-1118.338172	-1118.231597	427.52	nm
-1118.336092	-1118.231649	436.25	nm
-1118.33358	-1118.231691	447.19	nm
-1118.333041	-1118.231721	449.7	nm
-1118.332166	-1118.231734	453.68	nm
-1118.32342	-1118.231701	496.77	nm
-1118.329164	-1118.231785	467.9	nm
-1118.328085	-1118.231803	473.23	nm
-1118.320392	-1118.23174	513.96	nm
-1118.324519	-1118.23181	491.46	nm
-1118.326505	-1118.231819	481.21	nm
-1118.327127	-1118.231807	478.01	nm
-1118.320919	-1118.231807	511.3	nm
-1118.324157	-1118.231836	493.53	nm
-1118.325539	-1118.23183	486.22	nm
-1118.323785	-1118.231837	495.54	nm
-1118.324859	-1118.231834	489.8	nm
-1118.32387	-1118.231837	495.08	nm
-1118.324	-1118.233572	503.86	nm
-1118.314894	-1118.234245	564.96	nm
-1118.317708	-1118.234345	546.56	nm
-1118.316287	-1118.234388	556.33	nm
-1118.316349	-1118.23439	555.93	nm
-1118.316321	-1118.23439	556.12	nm
-1118.31633	-1118.23439	556.06	nm
-1118.316781	-1118.236158	565.14	nm
-1118.312913	-1118.236598	597.04	nm
-1118.314694	-1118.236639	583.74	nm
-1118.31428	-1118.236645	586.89	nm
-1118.314276	-1118.236645	586.92	nm
-1118.314259	-1118.236645	587.05	nm
-1118.314694	-1118.237965	593.83	nm
-1118.312536	-1118.238382	614.44	nm
-1118.312676	-1118.238393	613.38	nm
-1118.312562	-1118.238394	614.32	nm
-1118.312936	-1118.239142	617.43	nm
-1118.311024	-1118.239488	636.93	nm

-1118.310951	-1118.239495	637.64	nm
-1118.310957	-1118.239496	637.6	nm
-1118.310936	-1118.239496	637.79	nm
-1118.311131	-1118.239443	635.58	nm
-1118.309353	-1118.239713	654.26	nm
-1118.309663	-1118.239716	651.4	nm
-1118.309641	-1118.239716	651.6	nm
-1118.309628	-1118.239716	651.73	nm
-1118.309467	-1118.238647	643.38	nm
-1118.308318	-1118.238888	656.25	nm
-1118.308404	-1118.238891	655.46	nm
-1118.308427	-1118.238891	655.25	nm
-1118.307805	-1118.23669	640.69	nm
-1118.307172	-1118.23694	648.75	nm
-1118.307199	-1118.236942	648.52	nm
-1118.307217	-1118.236942	648.36	nm
-1118.306125	-1118.233565	627.93	nm
-1118.305893	-1118.233845	632.4	nm
-1118.305979	-1118.233847	631.67	nm
-1118.305994	-1118.233847	631.54	nm
-1118.304388	-1118.229318	606.95	nm
-1118.304602	-1118.229684	608.18	nm
-1118.304768	-1118.229689	606.87	nm
-1118.304786	-1118.229689	606.73	nm
-1118.304789	-1118.229689	606.7	nm
-1118.304784	-1118.229689	606.75	nm
-1118.302774	-1118.224254	580.28	nm
-1118.303717	-1118.22471	576.7	nm
-1118.303858	-1118.22472	575.75	nm
-1118.303909	-1118.224721	575.38	nm
-1118.303913	-1118.224721	575.36	nm
-1118.303921	-1118.224722	575.3	nm
-1118.301652	-1118.218654	548.97	nm
-1118.303018	-1118.219247	543.9	nm
-1118.303598	-1118.219275	540.34	nm
-1118.303967	-1118.21928	538.02	nm
-1118.304091	-1118.219281	537.24	nm
-1118.304113	-1118.219282	537.1	nm
-1118.301803	-1118.213161	514.01	nm
-1118.304958	-1118.21413	501.64	nm
-1118.30845	-1118.214658	485.79	nm
-1118.313921	-1118.21565	463.65	nm
-1118.318723	-1118.216847	447.24	nm
-1118.331948	-1118.218536	401.75	nm
-1118.336597	-1118.221948	397.42	nm
-1118.340247	-1118.2241	392.29	nm
-1118.345822	-1118.226075	380.5	nm
-1118.348732	-1118.226435	372.56	nm

-1118.349708	-1118.226843	370.84	nm
-1118.350834	-1118.227034	368.04	nm
-1118.352304	-1118.227162	364.09	nm
-1118.352856	-1118.22731	362.92	nm
-1118.35421	-1118.227753	360.31	nm
-1118.353813	-1118.227785	361.53	nm
-1118.35368	-1118.22817	363.03	nm
-1118.352431	-1118.228503	367.66	nm
-1118.34168	-1118.22783	400.21	nm
-1118.350632	-1118.228832	374.08	nm
-1118.351391	-1118.228288	370.12	nm
-1118.347934	-1118.228882	382.72	nm
-1118.347798	-1118.228897	383.2	nm
-1118.348303	-1118.228924	381.67	nm
-1118.347943	-1118.228926	382.83	nm
-1118.347439	-1118.228932	384.48	nm
-1118.34798	-1118.228942	382.76	nm
-1118.34771	-1118.228944	383.64	nm
-1118.347271	-1118.228947	385.07	nm
-1118.347354	-1118.228947	384.8	nm
-1118.347368	-1118.228947	384.76	nm
-1118.346469	-1118.227382	382.61	nm
-1118.348016	-1118.227663	378.58	nm
-1118.349035	-1118.227698	375.51	nm
-1118.350253	-1118.227718	371.84	nm
-1118.351571	-1118.227738	367.94	nm
-1118.297625	-1118.174197	369.15	nm
-1118.352039	-1118.224414	357.01	nm
-1118.351977	-1118.227746	366.76	nm
-1118.341549	-1118.219574	373.55	nm
-1118.354281	-1118.227453	359.25	nm
-1118.353409	-1118.227774	362.67	nm
-1118.353832	-1118.227755	361.39	nm
-1118.353737	-1118.227689	361.48	nm
-1118.354197	-1118.227787	360.44	nm
-1118.353441	-1118.227803	362.65	nm
-1118.354029	-1118.227829	361.04	nm
-1118.354402	-1118.227825	359.97	nm
-1118.354217	-1118.22784	360.54	nm
-1118.354172	-1118.227839	360.66	nm
-1118.354293	-1118.227841	360.32	nm
-1118.354249	-1118.227841	360.45	nm
-1118.354267	-1118.227841	360.4	nm
-1118.354263	-1118.227841	360.41	nm

Geometries of structural optimization

1 Energy:-1118.35426335

C -2.749096 3.181287 0.001204
C -1.351786 3.291935 0.004979
C -0.617686 2.107198 0.006949
C -1.262847 0.846144 0.005632
C -2.725727 0.746312 0.000975
C -3.433451 1.969534 -0.001021
N -0.517494 -0.311358 0.009071
C -1.095448 -1.645374 0.003967
C -2.529687 -1.733890 -0.001544
C -3.348799 -0.537700 -0.001790
C -3.135825 -3.004425 -0.006367
C -4.513593 -3.142451 -0.010776
C -5.332273 -1.975791 -0.010269
C -4.763581 -0.719387 -0.005959
O -0.302751 -2.587753 0.004908
C 0.925884 -0.249074 0.015642
C 1.614712 -0.213422 1.224714
C 3.011033 -0.161187 1.224534
C 3.745113 -0.140477 0.029919
C 3.018766 -0.173968 -1.174522
C 1.627084 -0.226397 -1.190833
F -3.474137 4.310858 -0.000776
C 5.283059 -0.084651 -0.003442
C 5.898518 -0.053639 1.408340
C 5.825177 -1.332959 -0.740195
C 5.733739 1.190570 -0.755579
H -0.876329 4.265063 0.006201
H 0.463010 2.147092 0.009727
H -4.515089 1.996589 -0.004548
H -2.490996 -3.878048 -0.006251
H -4.967165 -4.128708 -0.014306
H -6.413997 -2.075217 -0.013294
H -5.416562 0.147424 -0.005681
H 1.065233 -0.232861 2.160990
H 3.520233 -0.139001 2.180925
H 3.543515 -0.161312 -2.125041
H 1.085741 -0.255704 -2.131552
H 6.990546 -0.013680 1.330385
H 5.575394 0.826481 1.975929
H 5.641029 -0.948615 1.985989
H 6.920721 -1.303217 -0.776750
H 5.458780 -1.391039 -1.770435
H 5.526866 -2.252861 -0.224447
H 5.370921 2.093073 -0.250203
H 6.828537 1.239263 -0.793150
H 5.363901 1.209892 -1.786088

2 Energy:-1118.34757909

C -2.670928 3.154314 -0.262658
C -1.302286 3.252808 0.012232
C -0.603614 2.067943 0.202082
C -1.247567 0.809125 0.080509
C -2.686563 0.728281 -0.135569
C -3.360603 1.943101 -0.323830
N -0.516275 -0.355089 0.102167
C -1.148078 -1.652645 0.447697
C -2.558648 -1.723729 0.281988
C -3.333667 -0.562280 -0.112084
C -3.218174 -2.970609 0.453928
C -4.569059 -3.100501 0.212534
C -5.327502 -1.974833 -0.219182
C -4.710264 -0.741592 -0.366169
O -0.378379 -2.540699 0.825265
C 0.915992 -0.315666 0.090722
C 1.661549 -0.469496 1.263117
C 3.054039 -0.391026 1.215111
C 3.739983 -0.149015 0.015248
C 2.963045 0.019718 -1.148272
C 1.575375 -0.049882 -1.117192
F -3.368004 4.283615 -0.453297
C 5.273207 -0.060561 -0.067534
C 5.947414 -0.266644 1.302019
C 5.797164 -1.150644 -1.033741
C 5.678736 1.334525 -0.601741
H -0.824012 4.222271 0.082499
H 0.453908 2.096514 0.430209
H -4.427275 1.975934 -0.506484
H -2.624531 -3.825244 0.764006
H -5.054834 -4.063372 0.343517
H -6.387221 -2.079213 -0.430026
H -5.311326 0.103899 -0.687963
H 1.153965 -0.652258 2.203824
H 3.602526 -0.516536 2.141501
H 3.447621 0.205740 -2.101935
H 0.995907 0.078221 -2.026606
H 7.034766 -0.196149 1.188480
H 5.640997 0.495703 2.027321
H 5.722208 -1.252635 1.723895
H 6.889746 -1.094443 -1.108116
H 5.387826 -1.033769 -2.042621
H 5.531556 -2.151951 -0.676004
H 5.327367 2.128166 0.067735
H 6.770489 1.408300 -0.672539
H 5.266618 1.526851 -1.597882

3 Energy:-1118.34724539

C -2.668536 3.139734 -0.417762
C -1.316080 3.258878 -0.083836
C -0.621814 2.085873 0.184659
C -1.251597 0.820205 0.080962
C -2.677563 0.721199 -0.185776
C -3.348243 1.922161 -0.457245
N -0.512501 -0.338705 0.185357
C -1.151871 -1.625075 0.563697
C -2.556715 -1.705601 0.359439
C -3.321437 -0.570504 -0.120307
C -3.219284 -2.945192 0.575180
C -4.560621 -3.093195 0.296674
C -5.306832 -1.995106 -0.218429
C -4.687753 -0.768706 -0.409964
O -0.393509 -2.500935 0.990707
C 0.911324 -0.297587 0.153241
C 1.683126 -0.561767 1.292408
C 3.073799 -0.490349 1.220359
C 3.738817 -0.149651 0.031985
C 2.938839 0.128065 -1.096630
C 1.553789 0.068479 -1.042476
F -3.363279 4.255473 -0.686858
C 5.269810 -0.067962 -0.075330
C 5.970136 -0.397204 1.256498
C 5.761309 -1.076041 -1.142979
C 5.679432 1.364252 -0.497075
H -0.847009 4.233535 -0.024736
H 0.422336 2.131083 0.466952
H -4.406259 1.939586 -0.686913
H -2.633987 -3.780036 0.948428
H -5.047954 -4.050145 0.461268
H -6.358804 -2.114616 -0.458262
H -5.280087 0.056637 -0.794334
H 1.193890 -0.816023 2.224980
H 3.639759 -0.697128 2.121261
H 3.405776 0.389389 -2.041337
H 0.958502 0.274782 -1.926839
H 7.055516 -0.326692 1.125730
H 5.687066 0.302594 2.051004
H 5.743415 -1.413861 1.596795
H 6.852683 -1.023644 -1.234967
H 5.333206 -0.868858 -2.129263
H 5.491911 -2.101824 -0.866864
H 5.353371 2.100322 0.246737
H 6.770099 1.432419 -0.587279
H 5.247016 1.646286 -1.462699

4 Energy:-1118.34684541

C -2.677625 3.123907 -0.559040
C -1.344990 3.273563 -0.168846
C -0.651621 2.118943 0.176080
C -1.259738 0.843266 0.091928
C -2.669278 0.716212 -0.226769
C -3.340064 1.896764 -0.578583
N -0.508774 -0.301876 0.285750
C -1.151630 -1.586035 0.676286
C -2.548249 -1.683097 0.427476
C -3.304872 -0.577842 -0.126997
C -3.208565 -2.919199 0.673912
C -4.537857 -3.091830 0.357377
C -5.274474 -2.023776 -0.229749
C -4.658611 -0.801394 -0.453715
O -0.404440 -2.450794 1.143657
C 0.906418 -0.258045 0.230950
C 1.703832 -0.621693 1.328642
C 3.092028 -0.566727 1.227454
C 3.737267 -0.149054 0.051805
C 2.915682 0.225078 -1.034518
C 1.533654 0.183713 -0.952474
F -3.373527 4.218934 -0.904634
C 5.265376 -0.083448 -0.085518
C 5.990297 -0.526808 1.199204
C 5.714374 -1.012614 -1.240484
C 5.689777 1.370964 -0.405512
H -0.889764 4.255417 -0.122755
H 0.376515 2.190025 0.508437
H -4.386914 1.890744 -0.855481
H -2.629297 -3.731546 1.102005
H -5.022776 -4.045620 0.545839
H -6.316882 -2.163000 -0.499121
H -5.244172 0.001307 -0.892531
H 1.233125 -0.930446 2.253017
H 3.674535 -0.845618 2.097796
H 3.365884 0.543904 -1.969524
H 0.924004 0.456222 -1.808452
H 7.073375 -0.465134 1.047112
H 5.739640 0.114215 2.051999
H 5.752132 -1.562687 1.465614
H 6.804048 -0.969158 -1.354027
H 5.267597 -0.722258 -2.197002
H 5.434248 -2.052635 -1.038499
H 5.393487 2.052275 0.400263
H 6.779164 1.428172 -0.516040
H 5.240857 1.733254 -1.336173

5 Energy:-1118.34530216

C -2.680780 3.110148 -0.665084
C -1.376186 3.294584 -0.206686
C -0.686707 2.161840 0.213311
C -1.267730 0.874885 0.135556
C -2.651126 0.714310 -0.254854
C -3.320224 1.870947 -0.681247
N -0.507463 -0.249463 0.426132
C -1.162205 -1.530090 0.835556
C -2.538359 -1.654178 0.501793
C -3.273415 -0.587375 -0.146763
C -3.199622 -2.887961 0.764897
C -4.502685 -3.095393 0.371453
C -5.212805 -2.067440 -0.310987
C -4.598867 -0.846000 -0.549246
O -0.441680 -2.368313 1.384673
C 0.898621 -0.208865 0.338665
C 1.727699 -0.659320 1.384423
C 3.111084 -0.624745 1.240254
C 3.730152 -0.147703 0.071630
C 2.882040 0.306599 -0.963562
C 1.504615 0.287016 -0.840068
F -3.375063 4.181348 -1.084321
C 5.253068 -0.102982 -0.110221
C 6.008572 -0.627703 1.125189
C 5.645851 -0.977541 -1.327206
C 5.696724 1.358422 -0.367430
H -0.939078 4.284809 -0.164294
H 0.318563 2.261568 0.604442
H -4.350037 1.836787 -1.014628
H -2.639060 -3.670637 1.266650
H -4.986258 -4.047603 0.571429
H -6.233186 -2.235119 -0.641565
H -5.164961 -0.072273 -1.059942
H 1.281623 -1.007668 2.305545
H 3.714596 -0.964331 2.073915
H 3.309161 0.667355 -1.894141
H 0.877086 0.612454 -1.663538
H 7.087327 -0.576080 0.942206
H 5.796062 -0.029690 2.018595
H 5.759743 -1.672391 1.342856
H 6.732016 -0.947937 -1.473581
H 5.174734 -0.627827 -2.251578
H 5.351338 -2.021438 -1.171232
H 5.439875 2.001630 0.481923
H 6.783096 1.400099 -0.509598
H 5.227047 1.777811 -1.263166

6 Energy:-1118.33667721

C -2.677429 3.107911 -0.721262
C -1.430537 3.350349 -0.148875
C -0.741561 2.255755 0.363119
C -1.271550 0.951368 0.268460
C -2.585131 0.726329 -0.270080
C -3.262327 1.841611 -0.774751
N -0.515337 -0.148802 0.664921
C -1.246009 -1.348411 1.240537
C -2.517970 -1.583003 0.653948
C -3.149587 -0.616415 -0.217754
C -3.204242 -2.799654 0.939886
C -4.397002 -3.102687 0.319224
C -4.973841 -2.193010 -0.605211
C -4.356045 -0.968384 -0.842601
O -0.680771 -1.992236 2.129333
C 0.871225 -0.163895 0.502681
C 1.751482 -0.691966 1.475565
C 3.122382 -0.684934 1.253706
C 3.694675 -0.160728 0.078712
C 2.803526 0.367591 -0.882545
C 1.436385 0.374996 -0.685567
F -3.370809 4.143707 -1.225220
C 5.204783 -0.147841 -0.184400
C 6.010500 -0.750102 0.982039
C 5.505476 -0.973622 -1.460874
C 5.675268 1.312456 -0.398978
H -1.034673 4.357461 -0.094503
H 0.221281 2.398423 0.840907
H -4.253021 1.754630 -1.204770
H -2.742670 -3.498066 1.631149
H -4.890107 -4.048687 0.526763
H -5.898758 -2.443058 -1.115508
H -4.829093 -0.269088 -1.527012
H 1.344882 -1.079195 2.398014
H 3.760274 -1.082321 2.034588
H 3.188895 0.763153 -1.817178
H 0.778422 0.750003 -1.462112
H 7.078772 -0.716979 0.742471
H 5.862912 -0.190307 1.912499
H 5.745041 -1.797430 1.164408
H 6.582563 -0.964607 -1.665292
H 4.995068 -0.568423 -2.340675
H 5.190833 -2.015890 -1.336881
H 5.482843 1.921584 0.491441
H 6.753214 1.331578 -0.598227
H 5.170960 1.785387 -1.247937

7 Energy:-1118.31746675

C -2.854561 3.121428 -0.753270
C -1.656686 3.480025 -0.140725
C -0.878307 2.461570 0.404904
C -1.299959 1.127337 0.314870
C -2.547216 0.772458 -0.285348
C -3.315177 1.806443 -0.826318
N -0.514144 0.047256 0.734396
C -1.278922 -1.017879 1.533648
C -2.406509 -1.477460 0.789136
C -2.970528 -0.638791 -0.241471
C -3.051832 -2.700484 1.106369
C -4.109451 -3.157366 0.340839
C -4.587604 -2.391320 -0.745243
C -4.034391 -1.138005 -1.004292
O -0.946992 -1.236056 2.707935
C 0.831416 -0.061316 0.501743
C 1.683591 -0.754011 1.406331
C 3.048946 -0.809877 1.175165
C 3.649607 -0.197215 0.058359
C 2.790697 0.495054 -0.832163
C 1.430707 0.576364 -0.625341
F -3.621409 4.091248 -1.288533
C 5.153977 -0.258162 -0.222146
C 5.922506 -1.045884 0.855470
C 5.389348 -0.947842 -1.590151
C 5.722023 1.182771 -0.271881
H -1.356438 4.520411 -0.093750
H 0.060454 2.692730 0.897529
H -4.273362 1.617736 -1.297104
H -2.665995 -3.290881 1.932505
H -4.564022 -4.118426 0.566546
H -5.399580 -2.764110 -1.362145
H -4.451599 -0.532090 -1.804983
H 1.249366 -1.205965 2.287832
H 3.662144 -1.334992 1.897973
H 3.203272 0.966269 -1.718656
H 0.800479 1.088238 -1.343695
H 6.988791 -1.058790 0.605741
H 5.820814 -0.589012 1.846253
H 5.585470 -2.086523 0.919248
H 6.463487 -0.988070 -1.805428
H 4.904576 -0.409223 -2.410783
H 5.004393 -1.973687 -1.583666
H 5.577964 1.694574 0.686244
H 6.797248 1.150963 -0.483069
H 5.246598 1.785113 -1.052719

8 Energy:-1118.31399405

C -2.918116 3.115531 -0.837147
C -1.779550 3.548623 -0.164010
C -0.979665 2.582344 0.445772
C -1.327503 1.230971 0.358273
C -2.514779 0.796801 -0.300063
C -3.303567 1.778207 -0.909640
N -0.514657 0.191385 0.848984
C -1.267505 -0.880341 1.641405
C -2.324805 -1.418886 0.844896
C -2.870219 -0.633561 -0.236857
C -2.928393 -2.662881 1.159891
C -3.919836 -3.189662 0.351879
C -4.372482 -2.474929 -0.778682
C -3.865372 -1.204071 -1.042625
O -0.995401 -1.036196 2.842255
C 0.808505 0.046699 0.569200
C 1.641096 -0.745184 1.416434
C 2.996833 -0.854504 1.158795
C 3.609804 -0.204156 0.069935
C 2.772371 0.585129 -0.762740
C 1.423239 0.722849 -0.530040
F -3.702314 4.034897 -1.436734
C 5.103411 -0.321658 -0.242294
C 5.849952 -1.205947 0.773993
C 5.279614 -0.942568 -1.651877
C 5.741460 1.090650 -0.222766
H -1.535976 4.604039 -0.123869
H -0.081330 2.870842 0.982107
H -4.221978 1.529433 -1.429351
H -2.560631 -3.212861 2.021424
H -4.339935 -4.166065 0.578535
H -5.130968 -2.901995 -1.427759
H -4.265174 -0.639974 -1.881796
H 1.199932 -1.222396 2.281129
H 3.593876 -1.453733 1.835720
H 3.197325 1.088888 -1.624897
H 0.811651 1.311869 -1.203086
H 6.910005 -1.254807 0.503454
H 5.786592 -0.802497 1.790802
H 5.464193 -2.231407 0.784696
H 6.346568 -1.022107 -1.890129
H 4.808119 -0.334629 -2.430675
H 4.844542 -1.947190 -1.695356
H 5.637897 1.554630 0.764419
H 6.810276 1.016572 -0.454280
H 5.286090 1.758395 -0.961167

9 Energy:-1118.31213834

C -2.945073 3.099080 -0.934439
C -1.882118 3.602034 -0.190264
C -1.078924 2.687729 0.493247
C -1.351573 1.322096 0.406505
C -2.466030 0.814298 -0.317708
C -3.256557 1.744263 -1.005729
N -0.517338 0.330163 0.976959
C -1.263290 -0.758968 1.745648
C -2.247261 -1.360178 0.901916
C -2.763379 -0.626569 -0.230792
C -2.816575 -2.621112 1.217060
C -3.737698 -3.210467 0.371251
C -4.155135 -2.544992 -0.802809
C -3.686832 -1.261913 -1.074167
O -1.030821 -0.898937 2.957119
C 0.785515 0.150462 0.653032
C 1.596888 -0.728498 1.439248
C 2.938721 -0.890292 1.146310
C 3.561044 -0.211079 0.080141
C 2.746367 0.662885 -0.691968
C 1.411774 0.853487 -0.425248
F -3.729096 3.967718 -1.608426
C 5.041052 -0.377656 -0.267694
C 5.762741 -1.354489 0.679395
C 5.164236 -0.919351 -1.715268
C 5.742996 1.001447 -0.176082
H -1.694071 4.668951 -0.155884
H -0.233732 3.029839 1.082145
H -4.123306 1.439321 -1.581256
H -2.475478 -3.132096 2.112872
H -4.128798 -4.198050 0.601449
H -4.857658 -3.020842 -1.480343
H -4.060905 -0.738186 -1.950508
H 1.152318 -1.226401 2.290208
H 3.518577 -1.554717 1.775295
H 3.181131 1.193010 -1.532919
H 0.819301 1.509883 -1.050470
H 6.814527 -1.434620 0.385459
H 5.735661 -1.010534 1.719352
H 5.330183 -2.360224 0.636557
H 6.222394 -1.033195 -1.977093
H 4.710163 -0.244398 -2.447768
H 4.681970 -1.898465 -1.810375
H 5.678339 1.408266 0.839172
H 6.802826 0.893021 -0.433388
H 5.306117 1.732826 -0.863582

10 Energy:-1118.31063726

C -2.956341 3.070156 -1.039063
C -1.975967 3.639561 -0.231611
C -1.177114 2.778135 0.522961
C -1.373700 1.400860 0.442525
C -2.411393 0.823578 -0.338743
C -3.195162 1.701882 -1.102434
N -0.521959 0.460582 1.088093
C -1.263442 -0.643740 1.838182
C -2.176084 -1.301853 0.959433
C -2.657577 -0.622260 -0.222309
C -2.713293 -2.576379 1.281536
C -3.563109 -3.225734 0.407062
C -3.941878 -2.610956 -0.808181
C -3.508814 -1.319724 -1.093692
O -1.060033 -0.772854 3.055922
C 0.767194 0.254433 0.738305
C 1.547138 -0.712981 1.454472
C 2.872557 -0.924670 1.125819
C 3.511482 -0.210712 0.092195
C 2.730606 0.752649 -0.607699
C 1.412374 0.991652 -0.307383
F -3.732378 3.887243 -1.785132
C 4.974897 -0.426948 -0.292917
C 5.659471 -1.502359 0.571249
C 5.047695 -0.871962 -1.776604
C 5.746836 0.906434 -0.119454
H -1.843345 4.715002 -0.206083
H -0.390344 3.171839 1.158888
H -4.005839 1.343494 -1.726897
H -2.401391 -3.047503 2.209203
H -3.927596 -4.221411 0.646031
H -4.588009 -3.133828 -1.506860
H -3.853477 -0.837253 -2.005008
H 1.094708 -1.239604 2.283328
H 3.426976 -1.657877 1.698469
H 3.181263 1.315538 -1.418206
H 0.846778 1.717771 -0.877095
H 6.700682 -1.615410 0.251845
H 5.667306 -1.230203 1.632538
H 5.174380 -2.479347 0.468179
H 6.094668 -1.020644 -2.064229
H 4.618817 -0.125743 -2.452953
H 4.514257 -1.816472 -1.929924
H 5.718311 1.244040 0.922410
H 6.795640 0.763449 -0.403254
H 5.337401 1.704678 -0.746755

11 Energy:-1118.30952388

C -2.957174 3.028873 -1.147250
C -2.050372 3.656854 -0.297746
C -1.259450 2.845561 0.518390
C -1.390341 1.460364 0.455986
C -2.361606 0.822453 -0.361502
C -3.135325 1.652027 -1.190389
N -0.526301 0.571363 1.168015
C -1.264371 -0.541116 1.910151
C -2.119888 -1.249032 1.015467
C -2.570366 -0.623033 -0.208119
C -2.631058 -2.530778 1.356036
C -3.422462 -3.233527 0.469656
C -3.768799 -2.669039 -0.780394
C -3.362879 -1.375553 -1.090316
O -1.074335 -0.658297 3.130749
C 0.756414 0.345372 0.810525
C 1.493609 -0.711978 1.443356
C 2.802499 -0.965377 1.082631
C 3.469143 -0.206307 0.099423
C 2.732888 0.846940 -0.515859
C 1.430434 1.125325 -0.186272
F -3.722553 3.797044 -1.955237
C 4.917290 -0.463085 -0.315078
C 5.550477 -1.639563 0.450913
C 4.960245 -0.785936 -1.831274
C 5.755574 0.811698 -0.038194
H -1.964954 4.737314 -0.288506
H -0.526469 3.285874 1.187262
H -3.896646 1.245852 -1.846890
H -2.343587 -2.962145 2.310513
H -3.766302 -4.232331 0.725641
H -4.369423 -3.233789 -1.487027
H -3.682140 -0.933931 -2.031081
H 1.025826 -1.277814 2.236591
H 3.322216 -1.769715 1.588155
H 3.207293 1.449701 -1.282753
H 0.899313 1.921258 -0.691695
H 6.582267 -1.779004 0.112165
H 5.579415 -1.456467 1.530745
H 5.014544 -2.578462 0.273062
H 5.997187 -0.961913 -2.138756
H 4.566739 0.034327 -2.439967
H 4.379586 -1.686894 -2.057509
H 5.747585 1.063092 1.028075
H 6.794723 0.640071 -0.340759
H 5.384726 1.678268 -0.594779

12 Energy:-1118.30847019

C -2.941520 2.979321 -1.255978
C -2.104931 3.660656 -0.375521
C -1.328030 2.897667 0.498493
C -1.401911 1.507606 0.462040
C -2.309968 0.814722 -0.382561
C -3.067751 1.597285 -1.272104
N -0.532998 0.670472 1.238533
C -1.271438 -0.446318 1.974806
C -2.073575 -1.200428 1.070651
C -2.489258 -0.627383 -0.190969
C -2.562649 -2.486190 1.432093
C -3.297819 -3.238460 0.539150
C -3.608788 -2.723542 -0.742321
C -3.225093 -1.430580 -1.078596
O -1.095948 -0.548483 3.198393
C 0.749065 0.427571 0.886692
C 1.436940 -0.712638 1.425599
C 2.728538 -1.000755 1.032033
C 3.427850 -0.196678 0.108530
C 2.742805 0.939841 -0.411116
C 1.456233 1.250567 -0.051506
F -3.690969 3.699827 -2.121757
C 4.859169 -0.489877 -0.337578
C 5.434663 -1.757732 0.320429
C 4.878692 -0.683423 -1.876197
C 5.762388 0.712837 0.039718
H -2.060074 4.743511 -0.387288
H -0.647410 3.380328 1.193055
H -3.781731 1.147678 -1.953030
H -2.301808 -2.878589 2.410708
H -3.624716 -4.238011 0.813780
H -4.165461 -3.327088 -1.452862
H -3.516321 -1.029085 -2.046048
H 0.949048 -1.320449 2.173983
H 3.208247 -1.871220 1.461629
H 3.244675 1.581911 -1.126839
H 0.962755 2.110119 -0.485434
H 6.456489 -1.919565 -0.037845
H 5.477759 -1.668598 1.411537
H 4.850913 -2.649938 0.068378
H 5.903961 -0.885239 -2.205909
H 4.525899 0.205220 -2.408995
H 4.251219 -1.530645 -2.173747
H 5.773473 0.870910 1.123707
H 6.789479 0.515606 -0.287127
H 5.433100 1.641810 -0.436454

13 Energy:-1118.30756353

C -2.910144 2.921762 -1.362587
C -2.127194 3.646946 -0.467527
C -1.368414 2.925713 0.456258
C -1.405357 1.533799 0.454360
C -2.266962 0.796539 -0.401555
C -3.004363 1.538107 -1.343703
N -0.538137 0.741810 1.286350
C -1.280001 -0.370217 2.025324
C -2.049990 -1.160931 1.126390
C -2.435082 -0.637931 -0.166580
C -2.530659 -2.442176 1.517263
C -3.228630 -3.234941 0.630579
C -3.509559 -2.768057 -0.676935
C -3.133710 -1.482542 -1.046649
O -1.109734 -0.453267 3.250484
C 0.745676 0.479990 0.944663
C 1.379192 -0.736348 1.371259
C 2.657271 -1.044443 0.950475
C 3.397428 -0.187000 0.109846
C 2.765682 1.022111 -0.301508
C 1.490959 1.350967 0.082745
F -3.640146 3.600547 -2.278052
C 4.817822 -0.498443 -0.357294
C 5.337613 -1.841989 0.187057
C 4.836264 -0.556082 -1.907182
C 5.767677 0.628211 0.125886
H -2.107822 4.729910 -0.506051
H -0.728993 3.443402 1.164670
H -3.682057 1.053180 -2.037360
H -2.292355 -2.797083 2.515737
H -3.549661 -4.229326 0.929780
H -4.037244 -3.403568 -1.381707
H -3.400998 -1.119190 -2.035816
H 0.863005 -1.395123 2.054481
H 3.093272 -1.975321 1.290537
H 3.299597 1.707660 -0.950381
H 1.037041 2.268064 -0.268589
H 6.354635 -2.012178 -0.180802
H 5.376450 -1.852050 1.281912
H 4.720999 -2.684243 -0.145659
H 5.854104 -0.769682 -2.252063
H 4.522040 0.389690 -2.359763
H 4.176488 -1.347647 -2.278891
H 5.778199 0.691394 1.219493
H 6.787637 0.416427 -0.213753
H 5.481353 1.608095 -0.268985

14 Energy:-1118.30680329

C -2.870676 2.851452 -1.473412
C -2.126851 3.616901 -0.578269
C -1.387937 2.936102 0.391120
C -1.404179 1.544445 0.434471
C -2.234089 0.767615 -0.418744
C -2.948787 1.468798 -1.409459
N -0.541722 0.794713 1.315392
C -1.288018 -0.304510 2.066393
C -2.043293 -1.127382 1.185568
C -2.403821 -0.655450 -0.133988
C -2.524813 -2.396557 1.614997
C -3.201780 -3.225480 0.746039
C -3.459477 -2.808770 -0.583860
C -3.081687 -1.536865 -0.994782
O -1.119046 -0.359082 3.292687
C 0.745758 0.513049 0.986543
C 1.323200 -0.765470 1.287578
C 2.591322 -1.082546 0.844456
C 3.377020 -0.173574 0.104518
C 2.799481 1.095274 -0.186711
C 1.532194 1.431416 0.216730
F -3.579407 3.489255 -2.434372
C 4.790954 -0.493427 -0.375852
C 5.248719 -1.906200 0.031797
C 4.831258 -0.390931 -1.922984
C 5.776427 0.537660 0.232977
H -2.120890 4.698226 -0.650974
H -0.779901 3.486389 1.102543
H -3.599698 0.951850 -2.105617
H -2.303609 -2.712391 2.630400
H -3.524484 -4.209528 1.075965
H -3.970860 -3.472919 -1.274114
H -3.330356 -1.213271 -2.002420
H 0.770771 -1.475738 1.885672
H 2.982042 -2.063638 1.083152
H 3.368899 1.822809 -0.754666
H 1.118257 2.396907 -0.042377
H 6.264027 -2.078418 -0.339813
H 5.269877 -2.031326 1.120055
H 4.605069 -2.683621 -0.394456
H 5.845067 -0.609676 -2.276320
H 4.562489 0.608865 -2.277998
H 4.146262 -1.111633 -2.382635
H 5.772851 0.486072 1.327218
H 6.792332 0.321211 -0.115729
H 5.534298 1.563938 -0.060561

15 Energy:-1118.30616419

C -2.826618 2.766439 -1.586846
C -2.107012 3.569179 -0.704433
C -1.388315 2.928526 0.306602
C -1.399992 1.539586 0.404344
C -2.213490 0.727299 -0.433461
C -2.903863 1.387676 -1.469397
N -0.543278 0.827036 1.324866
C -1.296885 -0.247165 2.099491
C -2.053735 -1.099293 1.249548
C -2.395749 -0.680310 -0.092751
C -2.546784 -2.346898 1.726830
C -3.219680 -3.207606 0.886419
C -3.459942 -2.844850 -0.462985
C -3.069467 -1.594197 -0.923271
O -1.134846 -0.254449 3.327451
C 0.748745 0.525159 1.006493
C 1.270369 -0.798863 1.166622
C 2.533729 -1.114009 0.708374
C 3.368834 -0.157232 0.091928
C 2.843999 1.155950 -0.064757
C 1.578795 1.489472 0.350783
F -3.513040 3.363505 -2.589482
C 4.781686 -0.474988 -0.393611
C 5.175304 -1.943406 -0.147515
C 4.866669 -0.191568 -1.916155
C 5.788577 0.436442 0.355108
H -2.103078 4.646951 -0.818015
H -0.801311 3.509447 1.011075
H -3.537297 0.841845 -2.159600
H -2.337658 -2.621194 2.756799
H -3.552589 -4.174909 1.253473
H -3.967956 -3.534441 -1.130452
H -3.304767 -1.313016 -1.946693
H 0.673113 -1.555704 1.655531
H 2.879512 -2.132499 0.832137
H 3.451801 1.922077 -0.533342
H 1.204601 2.493268 0.197722
H 6.192914 -2.111366 -0.514672
H 5.162890 -2.197257 0.918178
H 4.514455 -2.638347 -0.677371
H 5.880293 -0.408776 -2.270908
H 4.646361 0.853531 -2.155333
H 4.166887 -0.823807 -2.473377
H 5.754384 0.255964 1.435027
H 6.804504 0.222356 0.004943
H 5.592121 1.498991 0.180838

16 Energy:-1118.30569227

C -2.787009 2.677676 -1.690532
C -2.072089 3.511528 -0.833270
C -1.371190 2.907910 0.212194
C -1.396197 1.524474 0.370108
C -2.209331 0.683877 -0.442109
C -2.877888 1.305905 -1.515757
N -0.544778 0.843947 1.320098
C -1.305411 -0.200819 2.122174
C -2.073323 -1.078170 1.309856
C -2.408054 -0.706901 -0.048187
C -2.578776 -2.301293 1.834588
C -3.259883 -3.186782 1.026926
C -3.494231 -2.873175 -0.335882
C -3.089887 -1.645658 -0.843994
O -1.154035 -0.149198 3.350602
C 0.753688 0.523801 1.010888
C 1.220723 -0.825977 1.016818
C 2.484396 -1.131776 0.551241
C 3.369561 -0.138743 0.077728
C 2.894295 1.200472 0.066143
C 1.627486 1.525679 0.489233
F -3.454157 3.236829 -2.727164
C 4.784895 -0.449142 -0.406947
C 5.125028 -1.948585 -0.319268
C 4.919964 -0.000405 -1.885386
C 5.800008 0.336237 0.463410
H -2.057931 4.583526 -0.991611
H -0.789458 3.514338 0.899083
H -3.504668 0.736592 -2.192937
H -2.372547 -2.538695 2.874305
H -3.603423 -4.135760 1.430080
H -4.008361 -3.582662 -0.977345
H -3.320973 -1.402825 -1.878053
H 0.577175 -1.614256 1.383862
H 2.788016 -2.171145 0.550552
H 3.539901 1.995319 -0.291006
H 1.290477 2.553699 0.453038
H 6.147570 -2.108539 -0.676274
H 5.072474 -2.320407 0.709927
H 4.459566 -2.556451 -0.942153
H 5.935678 -0.210395 -2.238670
H 4.737834 1.071539 -2.010296
H 4.216498 -0.542697 -2.526540
H 5.728931 0.038369 1.515204
H 6.817845 0.125208 0.116929
H 5.644783 1.418080 0.404553

17 Energy:-1118.30742515

C -2.804243 2.562187 -1.805078
C -2.027410 3.416190 -1.024495
C -1.323284 2.849159 0.037550
C -1.403130 1.479917 0.291200
C -2.276506 0.625833 -0.447740
C -2.946787 1.208375 -1.540511
N -0.543173 0.829819 1.242570
C -1.282136 -0.184874 2.096336
C -2.110865 -1.072634 1.364061
C -2.500067 -0.742017 0.010916
C -2.620835 -2.260359 1.959919
C -3.365004 -3.155056 1.220066
C -3.657005 -2.885239 -0.140476
C -3.244554 -1.688919 -0.714771
O -1.081672 -0.070215 3.314361
C 0.774899 0.512538 0.952315
C 1.192791 -0.836178 0.787359
C 2.469289 -1.124124 0.337995
C 3.407214 -0.109612 0.053778
C 2.977474 1.231718 0.215577
C 1.697997 1.541582 0.625582
F -3.476195 3.081776 -2.856484
C 4.836865 -0.403412 -0.405068
C 5.115076 -1.912179 -0.540183
C 5.074174 0.263902 -1.784097
C 5.829560 0.187704 0.628685
H -1.972596 4.474255 -1.252144
H -0.698810 3.473426 0.667560
H -3.609432 0.625338 -2.169870
H -2.369041 -2.466283 2.996235
H -3.714227 -4.077943 1.675386
H -4.220303 -3.602886 -0.729296
H -3.519098 -1.480238 -1.745472
H 0.500927 -1.640982 1.003131
H 2.736685 -2.164280 0.197990
H 3.665939 2.044769 0.011442
H 1.398960 2.576919 0.733507
H 6.148475 -2.061999 -0.869633
H 4.993130 -2.437697 0.413403
H 4.460605 -2.383913 -1.281620
H 6.098607 0.062609 -2.117157
H 4.943766 1.350030 -1.745439
H 4.385941 -0.134996 -2.537390
H 5.690528 -0.271158 1.613745
H 6.857920 -0.006774 0.303591
H 5.712741 1.270471 0.738822

18 Energy:-1118.34773491

C -2.851473 2.223066 -1.999150
C -1.489959 2.153543 -2.315142
C -0.723952 1.219208 -1.630435
C -1.295200 0.398727 -0.623432
C -2.728216 0.433875 -0.360139
C -3.471045 1.390490 -1.066904
N -0.498013 -0.399159 0.163451
C -1.051901 -1.576525 0.875513
C -2.457539 -1.571013 1.092772
C -3.300498 -0.514430 0.565534
C -3.042904 -2.592762 1.887828
C -4.386254 -2.573071 2.196249
C -5.211614 -1.515069 1.718391
C -4.667219 -0.521675 0.918622
O -0.230033 -2.429615 1.222389
C 0.929348 -0.301234 0.090125
C 1.688315 -1.219768 -0.640894
C 3.074021 -1.072220 -0.714205
C 3.739401 -0.012906 -0.078641
C 2.948326 0.908245 0.636769
C 1.566806 0.779511 0.714936
F -3.612373 3.119925 -2.642971
C 5.265157 0.171211 -0.138339
C 5.956521 -0.926877 -0.968362
C 5.592871 1.541294 -0.779799
C 5.841636 0.127548 1.297711
H -1.067826 2.801351 -3.073867
H 0.330982 1.121255 -1.851270
H -4.538236 1.499413 -0.920381
H -2.398557 -3.382628 2.261847
H -4.815019 -3.360015 2.810335
H -6.265706 -1.486388 1.976192
H -5.318562 0.269641 0.559258
H 1.196346 -2.044282 -1.144890
H 3.633321 -1.802348 -1.287694
H 3.417189 1.742894 1.149025
H 0.976552 1.497654 1.276283
H 7.037771 -0.750843 -0.978838
H 5.787989 -1.924385 -0.547151
H 5.612413 -0.930532 -2.008907
H 6.678786 1.687937 -0.820850
H 5.165807 2.372819 -0.209494
H 5.203514 1.598596 -1.802845
H 5.630888 -0.836025 1.775478
H 6.929377 0.263733 1.271368
H 5.421957 0.916379 1.930683

19 Energy:-1118.35420583

C -2.920875 2.059233 -2.191231
C -1.531859 2.170745 -2.343192
C -0.734422 1.360382 -1.537392
C -1.309720 0.464173 -0.603396
C -2.764760 0.352283 -0.461627
C -3.537905 1.192605 -1.294038
N -0.502385 -0.313854 0.195301
C -1.006708 -1.285857 1.151611
C -2.433554 -1.394410 1.279130
C -3.316905 -0.569730 0.477813
C -2.969575 -2.313093 2.201496
C -4.337493 -2.449731 2.365995
C -5.218577 -1.644181 1.587481
C -4.719261 -0.737198 0.676277
O -0.163132 -1.932483 1.773921
C 0.935448 -0.213609 0.096919
C 1.633025 -1.030654 -0.788347
C 3.023982 -0.929053 -0.874278
C 3.743947 -0.016688 -0.089713
C 3.008702 0.798140 0.790639
C 1.622175 0.709066 0.887481
F -3.706174 2.832704 -2.957068
C 5.275721 0.117980 -0.160659
C 5.903316 -0.858334 -1.173530
C 5.643921 1.559550 -0.586544
C 5.881598 -0.175570 1.232801
H -1.110403 2.862915 -3.061739
H 0.342373 1.414882 -1.621902
H -4.619240 1.183928 -1.257599
H -2.278051 -2.914096 2.784285
H -4.736547 -3.162500 3.080958
H -6.293105 -1.744055 1.711601
H -5.418683 -0.139970 0.100107
H 1.095361 -1.746828 -1.402222
H 3.540400 -1.581160 -1.568948
H 3.522095 1.519272 1.419400
H 1.074263 1.344888 1.576298
H 6.990546 -0.724087 -1.185139
H 5.702127 -1.903445 -0.912269
H 5.538059 -0.682160 -2.191712
H 6.733579 1.672011 -0.635127
H 5.263281 2.304477 0.120063
H 5.234021 1.793090 -1.575989
H 5.642354 -1.194624 1.557607
H 6.973130 -0.077029 1.197502
H 5.509100 0.516621 1.995132

20 Energy:-1118.34759786

C -2.933744 1.854366 -2.382927
C -1.578517 2.199773 -2.337611
C -0.787392 1.551772 -1.398300
C -1.327019 0.550232 -0.549572
C -2.753536 0.254362 -0.563442
C -3.523063 0.926709 -1.523000
N -0.501805 -0.200081 0.254098
C -1.026557 -0.902388 1.453878
C -2.426360 -1.153942 1.464745
C -3.293143 -0.668748 0.407272
C -2.982429 -1.943617 2.507476
C -4.317392 -2.286795 2.506203
C -5.164516 -1.851655 1.447149
C -4.649469 -1.056816 0.433712
O -0.190361 -1.210733 2.307226
C 0.921650 -0.095261 0.131231
C 1.564464 -0.790803 -0.897832
C 2.953323 -0.747566 -1.009688
C 3.740760 -0.019678 -0.102521
C 3.069369 0.661864 0.930029
C 1.684882 0.624121 1.059920
F -3.719489 2.463468 -3.282445
C 5.274228 0.052345 -0.199012
C 5.825722 -0.760763 -1.385450
C 5.706674 1.527558 -0.380969
C 5.898083 -0.506746 1.102534
H -1.180554 2.953529 -3.006196
H 0.263802 1.796819 -1.316762
H -4.588291 0.756116 -1.614851
H -2.321749 -2.285433 3.298376
H -4.722828 -2.896283 3.308979
H -6.211571 -2.137649 1.432919
H -5.316763 -0.729816 -0.358406
H 0.974967 -1.372653 -1.600130
H 3.418214 -1.304790 -1.814575
H 3.636516 1.236815 1.655956
H 1.191685 1.153212 1.867658
H 6.917709 -0.676264 -1.410338
H 5.578552 -1.825103 -1.301355
H 5.444037 -0.394000 -2.345097
H 6.799318 1.594836 -0.444375
H 5.382045 2.154851 0.455815
H 5.286443 1.948849 -1.301520
H 5.614962 -1.554545 1.254503
H 6.992067 -0.454043 1.048483
H 5.580271 0.059121 1.984371

21 Energy:-1118.3470889

C -2.917394 1.728827 -2.482557
C -1.579711 2.127324 -2.407281
C -0.798050 1.545389 -1.417141
C -1.327198 0.556064 -0.550370
C -2.738602 0.212016 -0.592771
C -3.500067 0.815644 -1.602849
N -0.499459 -0.128107 0.313617
C -1.039699 -0.791289 1.531623
C -2.431467 -1.080892 1.513592
C -3.278991 -0.682125 0.406304
C -2.997062 -1.831158 2.581719
C -4.319117 -2.218384 2.557666
C -5.144036 -1.870591 1.450341
C -4.621254 -1.113993 0.411343
O -0.221155 -1.046279 2.419722
C 0.915871 -0.039241 0.181076
C 1.533543 -0.653863 -0.918966
C 2.918809 -0.631250 -1.048371
C 3.736545 -0.002360 -0.092786
C 3.093969 0.602260 1.004954
C 1.712192 0.584393 1.154742
F -3.696472 2.271958 -3.430121
C 5.268760 0.043447 -0.206323
C 5.784556 -0.670483 -1.470104
C 5.734862 1.519027 -0.259678
C 5.895300 -0.646266 1.030103
H -1.187991 2.873568 -3.087846
H 0.238032 1.839650 -1.306391
H -4.555595 0.603741 -1.718672
H -2.352119 -2.107992 3.410084
H -4.730868 -2.797711 3.379414
H -6.180222 -2.192602 1.418737
H -5.272300 -0.852337 -0.417835
H 0.922512 -1.164061 -1.657432
H 3.359892 -1.128365 -1.904390
H 3.684042 1.105045 1.765225
H 1.243817 1.059716 2.007929
H 6.877687 -0.607911 -1.504841
H 5.514116 -1.732431 -1.478608
H 5.397922 -0.209859 -2.386318
H 6.827893 1.565331 -0.333712
H 5.437657 2.074725 0.635774
H 5.313202 2.032537 -1.131387
H 5.589152 -1.696857 1.090075
H 6.989401 -0.613560 0.964522
H 5.602289 -0.156149 1.964344

22 Energy:-1118.3465484

C -2.892226 1.616307 -2.564352
C -1.575844 2.069760 -2.459397
C -0.807196 1.551164 -1.423532
C -1.325451 0.571712 -0.542057
C -2.718906 0.177293 -0.613233
C -3.467869 0.712933 -1.670581
N -0.498284 -0.041930 0.380883
C -1.056257 -0.686706 1.603898
C -2.437914 -1.017772 1.553171
C -3.262451 -0.692834 0.405942
C -3.014010 -1.740091 2.636025
C -4.321792 -2.169543 2.589707
C -5.121978 -1.894425 1.444507
C -4.590230 -1.166179 0.389973
O -0.259844 -0.900725 2.522352
C 0.909504 0.017276 0.237875
C 1.501767 -0.523205 -0.919669
C 2.882974 -0.523595 -1.068616
C 3.730721 0.012643 -0.081417
C 3.117152 0.549928 1.068234
C 1.739265 0.554523 1.239740
F -3.660542 2.094089 -3.556362
C 5.260506 0.032567 -0.216315
C 5.741489 -0.600100 -1.535848
C 5.759258 1.498222 -0.171715
C 5.888608 -0.756984 0.958338
H -1.189830 2.809897 -3.149765
H 0.210973 1.894079 -1.287196
H -4.510093 0.455395 -1.812152
H -2.386536 -1.961792 3.493824
H -4.740752 -2.727572 3.422505
H -6.146607 -2.249761 1.395881
H -5.223405 -0.959337 -0.468061
H 0.869836 -0.967370 -1.682581
H 3.299635 -0.966750 -1.965474
H 3.729760 0.987765 1.850456
H 1.295907 0.986228 2.127418
H 6.835194 -0.560558 -1.582569
H 5.445410 -1.652124 -1.616329
H 5.353801 -0.065633 -2.410520
H 6.851858 1.524955 -0.259712
H 5.488068 1.994841 0.765653
H 5.337231 2.081568 -0.998040
H 5.560180 -1.802454 0.948215
H 6.982039 -0.742529 0.877577
H 5.620073 -0.328629 1.929480

23 Energy:-1118.34487532

C -2.867406 1.525601 -2.626510
C -1.577925 2.039749 -2.492604
C -0.823094 1.583519 -1.417122
C -1.325095 0.606507 -0.526131
C -2.693346 0.151060 -0.629000
C -3.430169 0.622307 -1.724780
N -0.499791 0.069674 0.452395
C -1.081161 -0.552528 1.684326
C -2.442232 -0.950282 1.589116
C -3.234996 -0.711372 0.400254
C -3.030682 -1.650651 2.681596
C -4.313757 -2.143669 2.602694
C -5.077474 -1.956898 1.416162
C -4.536574 -1.247407 0.352622
O -0.319070 -0.695579 2.644285
C 0.900411 0.083742 0.297082
C 1.466862 -0.394501 -0.905670
C 2.843444 -0.424159 -1.075810
C 3.721553 0.025278 -0.071107
C 3.137810 0.508893 1.119155
C 1.765157 0.542718 1.313754
F -3.624580 1.940403 -3.655955
C 5.247811 0.012114 -0.228914
C 5.692717 -0.558977 -1.588557
C 5.785953 1.460152 -0.111262
C 5.871153 -0.857007 0.891219
H -1.201379 2.778693 -3.189460
H 0.172652 1.978702 -1.256099
H -4.455039 0.314032 -1.890429
H -2.429724 -1.805155 3.572410
H -4.740242 -2.686117 3.442045
H -6.081375 -2.363423 1.342492
H -5.143191 -1.105539 -0.537282
H 0.814814 -0.779939 -1.682897
H 3.234023 -0.823417 -2.004445
H 3.773405 0.887931 1.913560
H 1.348522 0.940644 2.228408
H 6.786333 -0.547084 -1.649194
H 5.365751 -1.596297 -1.722576
H 5.308980 0.034009 -2.426424
H 6.877410 1.462504 -0.215310
H 5.542130 1.910429 0.856455
H 5.367826 2.099290 -0.897244
H 5.515577 -1.891655 0.828534
H 6.963238 -0.865376 0.793866
H 5.627962 -0.476372 1.888507

24 Energy:-1118.32387

C -3.019564 1.502947 -2.658683
C -1.826184 2.208535 -2.522955
C -1.006243 1.886114 -1.445731
C -1.373221 0.865561 -0.549686
C -2.621106 0.172823 -0.679520
C -3.433142 0.515246 -1.762166
N -0.521854 0.415868 0.458229
C -1.207186 0.143459 1.807031
C -2.351719 -0.686589 1.643483
C -2.990912 -0.803757 0.353603
C -2.929842 -1.353551 2.757618
C -3.995836 -2.215877 2.586165
C -4.551856 -2.419295 1.301465
C -4.062613 -1.695272 0.214965
O -0.780490 0.716380 2.816465
C 0.849986 0.354233 0.298909
C 1.390238 -0.090458 -0.940083
C 2.755770 -0.243596 -1.105675
C 3.663766 0.008172 -0.057295
C 3.111735 0.410943 1.179552
C 1.750809 0.566733 1.375189
F -3.830288 1.797610 -3.691368
C 5.180587 -0.150262 -0.208383
C 5.585823 -0.609999 -1.621516
C 5.863746 1.211616 0.074998
C 5.691176 -1.199486 0.810778
H -1.565201 2.985303 -3.232192
H -0.073692 2.418178 -1.290351
H -4.391960 0.037081 -1.926598
H -2.484378 -1.205459 3.736973
H -4.399466 -2.752087 3.441025
H -5.371172 -3.118154 1.163909
H -4.535493 -1.818502 -0.756325
H 0.715726 -0.339686 -1.751984
H 3.115827 -0.597407 -2.064631
H 3.770580 0.613777 2.018362
H 1.358097 0.884275 2.330808
H 6.675941 -0.700711 -1.676974
H 5.160181 -1.588577 -1.870765
H 5.274348 0.107056 -2.389436
H 6.951314 1.109213 -0.018533
H 5.647927 1.575855 1.084591
H 5.530774 1.973775 -0.638517
H 5.229568 -2.177039 0.631769
H 6.777514 -1.312928 0.716107
H 5.475180 -0.907510 1.843531

25 Energy:-1118.31632964

C -3.124516 1.432799 -2.707766
C -1.980422 2.219578 -2.607000
C -1.118255 1.975505 -1.539843
C -1.406311 0.956696 -0.621346
C -2.600032 0.177611 -0.712948
C -3.453753 0.436980 -1.787763
N -0.528341 0.571793 0.400164
C -1.206842 0.363820 1.765061
C -2.269545 -0.581529 1.643149
C -2.886391 -0.796437 0.356399
C -2.801286 -1.246169 2.777708
C -3.791941 -2.200927 2.633466
C -4.315236 -2.498679 1.356053
C -3.878824 -1.779026 0.244570
O -0.888403 1.104704 2.706115
C 0.825444 0.455645 0.247055
C 1.389172 0.092502 -1.011972
C 2.749092 -0.112209 -1.143201
C 3.632253 0.003882 -0.046641
C 3.057112 0.321661 1.204647
C 1.700339 0.524138 1.370442
F -3.970955 1.652399 -3.732996
C 5.143920 -0.212270 -0.166198
C 5.577163 -0.564184 -1.601799
C 5.875755 1.086292 0.259063
C 5.572544 -1.370031 0.770258
H -1.783493 2.994749 -3.338533
H -0.217271 2.567536 -1.416041
H -4.379807 -0.110024 -1.923914
H -2.380227 -1.023322 3.753923
H -4.157820 -2.735722 3.505989
H -5.072914 -3.267834 1.241184
H -4.332910 -1.975654 -0.723669
H 0.735668 -0.049188 -1.865109
H 3.128685 -0.399611 -2.116554
H 3.694635 0.414010 2.078316
H 1.291073 0.786930 2.336265
H 6.663434 -0.700625 -1.630981
H 5.118179 -1.496130 -1.950559
H 5.324926 0.231589 -2.311694
H 6.960086 0.941369 0.188041
H 5.643054 1.368936 1.290743
H 5.600360 1.923358 -0.392232
H 5.074410 -2.305279 0.491306
H 6.655238 -1.525244 0.696737
H 5.336147 -1.159839 1.818260

26 Energy:-1118.31425907

C -3.155527 1.363317 -2.756566
C -2.072076 2.231034 -2.657528
C -1.203727 2.063737 -1.578749
C -1.430392 1.042740 -0.650827
C -2.563014 0.181772 -0.736171
C -3.422130 0.361155 -1.825416
N -0.537812 0.736181 0.394106
C -1.208572 0.511035 1.753547
C -2.204005 -0.507185 1.641004
C -2.795616 -0.784472 0.353643
C -2.703856 -1.181418 2.784295
C -3.631982 -2.198367 2.652425
C -4.124425 -2.552883 1.377235
C -3.723732 -1.830316 0.255085
O -0.938968 1.285413 2.684132
C 0.800073 0.553491 0.238246
C 1.371675 0.237536 -1.032385
C 2.723035 -0.014584 -1.150585
C 3.592864 0.009907 -0.034876
C 3.011562 0.290863 1.223029
C 1.662981 0.540099 1.378195
F -4.005031 1.506846 -3.794584
C 5.095453 -0.262139 -0.144004
C 5.537893 -0.560194 -1.588976
C 5.872166 0.982478 0.357039
C 5.459967 -1.481973 0.739704
H -1.921620 3.005727 -3.400536
H -0.344967 2.716426 -1.457978
H -4.305762 -0.252355 -1.960481
H -2.305464 -0.913245 3.758650
H -3.971055 -2.738276 3.532598
H -4.831952 -3.369795 1.272250
H -4.155857 -2.074673 -0.712289
H 0.730184 0.170599 -1.902884
H 3.109720 -0.264722 -2.131243
H 3.638721 0.316730 2.108374
H 1.250815 0.781305 2.348447
H 6.617724 -0.741503 -1.608343
H 5.046385 -1.453130 -1.991259
H 5.332051 0.280193 -2.261366
H 6.950472 0.796664 0.291316
H 5.636939 1.220973 1.399189
H 5.639920 1.862267 -0.253381
H 4.930756 -2.381101 0.405007
H 6.536867 -1.676355 0.676186
H 5.212509 -1.315251 1.792879

27 Energy:-1118.31256204

C -3.162495 1.290283 -2.803707
C -2.153451 2.242589 -2.697337
C -1.290766 2.153754 -1.603576
C -1.452611 1.128142 -0.671241
C -2.513125 0.183383 -0.758106
C -3.364913 0.281323 -1.866173
N -0.548997 0.903137 0.395058
C -1.215123 0.649919 1.747492
C -2.139212 -0.433608 1.638120
C -2.696909 -0.771747 0.349026
C -2.610999 -1.117132 2.788694
C -3.471984 -2.191790 2.666944
C -3.925405 -2.600795 1.392962
C -3.556203 -1.876995 0.261973
O -0.983945 1.435765 2.679494
C 0.772173 0.651343 0.236785
C 1.350736 0.375721 -1.041880
C 2.691581 0.076128 -1.151900
C 3.546542 0.016662 -0.023885
C 2.961116 0.270613 1.238618
C 1.623184 0.568327 1.386983
F -4.003044 1.355570 -3.858507
C 5.037414 -0.312017 -0.127000
C 5.488849 -0.560758 -1.578544
C 5.857181 0.874243 0.442826
C 5.336297 -1.586227 0.703470
H -2.050865 3.018044 -3.447643
H -0.484406 2.869697 -1.478974
H -4.196571 -0.399936 -2.006603
H -2.241818 -0.805845 3.761658
H -3.787232 -2.735486 3.553647
H -4.579327 -3.462213 1.296283
H -3.959122 -2.167741 -0.705110
H 0.721988 0.378836 -1.923554
H 3.084976 -0.141390 -2.137454
H 3.577944 0.237726 2.130711
H 1.209824 0.794429 2.360302
H 6.560452 -0.785676 -1.591780
H 4.967650 -1.412913 -2.028926
H 5.328145 0.317883 -2.213240
H 6.927446 0.646250 0.380663
H 5.618010 1.072427 1.492415
H 5.670082 1.790927 -0.127367
H 4.775450 -2.445547 0.319503
H 6.404978 -1.822581 0.645212
H 5.079700 -1.458174 1.759823

28 Energy:-1118.31093582

C -3.158580 1.216875 -2.845240
C -2.226824 2.244534 -2.731995
C -1.374335 2.227269 -1.626213
C -1.471861 1.198890 -0.690803
C -2.460178 0.180900 -0.777901
C -3.299091 0.204566 -1.902359
N -0.559846 1.050242 0.391635
C -1.225646 0.774827 1.738332
C -2.081999 -0.362598 1.634644
C -2.602862 -0.758539 0.345411
C -2.527960 -1.051772 2.793344
C -3.322888 -2.176292 2.683594
C -3.736498 -2.635772 1.412316
C -3.395378 -1.914742 0.272049
O -1.022810 1.566209 2.672442
C 0.745407 0.736440 0.235240
C 1.338359 0.516206 -1.048990
C 2.668263 0.173956 -1.152877
C 3.501094 0.022921 -0.015375
C 2.906371 0.240099 1.250500
C 1.579621 0.581433 1.393284
F -3.985582 1.210757 -3.914108
C 4.979237 -0.357631 -0.113256
C 5.443164 -0.560125 -1.568050
C 5.834079 0.770377 0.520245
C 5.215740 -1.677701 0.664432
H -2.171258 3.019760 -3.487472
H -0.622659 2.999895 -1.498081
H -4.077660 -0.536163 -2.046316
H -2.188699 -0.700961 3.763700
H -3.616430 -2.720723 3.577306
H -4.338493 -3.535304 1.325863
H -3.767336 -2.248204 -0.693619
H 0.727996 0.595564 -1.939448
H 3.073373 0.000176 -2.142168
H 3.508396 0.144649 2.147913
H 1.162431 0.782921 2.370221
H 6.504898 -0.827645 -1.575811
H 4.895542 -1.369624 -2.063499
H 5.328514 0.351445 -2.165115
H 6.895440 0.503305 0.462965
H 5.586246 0.931741 1.574064
H 5.691422 1.717553 -0.011602
H 4.627669 -2.496813 0.236027
H 6.275147 -1.952697 0.608792
H 4.949658 -1.585652 1.722136

29 Energy:-1118.30962756

C -3.149807 1.137227 -2.881386
C -2.279490 2.218275 -2.770793
C -1.437478 2.260120 -1.657614
C -1.484992 1.237856 -0.712968
C -2.417938 0.169216 -0.791686
C -3.243834 0.131792 -1.927512
N -0.566651 1.154396 0.379443
C -1.235940 0.873936 1.723385
C -2.045375 -0.297308 1.635084
C -2.535719 -0.746300 0.350532
C -2.474411 -0.978002 2.806417
C -3.220922 -2.135964 2.716705
C -3.603303 -2.642417 1.452475
C -3.279930 -1.936300 0.298870
O -1.043843 1.671425 2.654426
C 0.724796 0.787021 0.229325
C 1.342917 0.644676 -1.054947
C 2.664987 0.275158 -1.154624
C 3.468799 0.026895 -0.012591
C 2.855046 0.184790 1.253778
C 1.536654 0.555281 1.392246
F -3.963766 1.072203 -3.959338
C 4.937213 -0.388279 -0.106333
C 5.423899 -0.519468 -1.561848
C 5.809363 0.676857 0.607654
C 5.121498 -1.757511 0.597169
H -2.259753 2.987010 -3.534632
H -0.729414 3.073500 -1.532997
H -3.981136 -0.650859 -2.066986
H -2.158751 -0.590849 3.770891
H -3.500584 -2.670995 3.620514
H -4.167705 -3.567410 1.382715
H -3.626926 -2.308385 -0.661958
H 0.756506 0.805509 -1.950147
H 3.089702 0.163791 -2.144538
H 3.437158 0.021527 2.154373
H 1.110038 0.715181 2.372570
H 6.476602 -0.820353 -1.565497
H 4.861466 -1.280229 -2.114599
H 5.350458 0.428939 -2.105604
H 6.863683 0.382763 0.554419
H 5.544994 0.784399 1.664272
H 5.703798 1.656657 0.128917
H 4.519654 -2.533185 0.111005
H 6.173815 -2.058567 0.543999
H 4.837816 -1.719467 1.653576

30 Energy:-1118.3084268

C -3.144042 1.050056 -2.913084
C -2.321385 2.169483 -2.815118
C -1.487571 2.263296 -1.699081
C -1.495428 1.254672 -0.739265
C -2.387132 0.150598 -0.800557
C -3.202685 0.059612 -1.942525
N -0.570201 1.228937 0.357277
C -1.241775 0.955259 1.701656
C -2.021303 -0.236273 1.637879
C -2.489646 -0.735075 0.363144
C -2.438339 -0.897481 2.825722
C -3.152008 -2.076902 2.763959
C -3.513016 -2.628436 1.511366
C -3.201175 -1.946619 0.340965
O -1.046429 1.760573 2.624935
C 0.709802 0.816854 0.214931
C 1.363095 0.770128 -1.059576
C 2.680381 0.384372 -1.154807
C 3.447939 0.031106 -0.015395
C 2.805619 0.111400 1.244862
C 1.493124 0.501701 1.378511
F -3.947848 0.933095 -3.995069
C 4.909033 -0.408398 -0.103674
C 5.430193 -0.445104 -1.552614
C 5.784228 0.582308 0.707359
C 5.045508 -1.828750 0.503062
H -2.329640 2.926482 -3.590782
H -0.814454 3.107332 -1.584734
H -3.908928 -0.753181 -2.069872
H -2.139000 -0.475621 3.780785
H -3.422550 -2.594606 3.680560
H -4.052104 -3.569823 1.464405
H -3.530533 -2.355150 -0.611221
H 0.805783 1.017948 -1.953382
H 3.132107 0.349917 -2.138293
H 3.362085 -0.126720 2.144859
H 1.050112 0.604425 2.358919
H 6.475235 -0.771473 -1.552744
H 4.864581 -1.148844 -2.173380
H 5.393441 0.542128 -2.026425
H 6.833116 0.269104 0.657230
H 5.495938 0.618191 1.762538
H 5.711008 1.596461 0.299561
H 4.439699 -2.553200 -0.052169
H 6.092139 -2.149244 0.453009
H 4.736217 -1.859988 1.552431

31 Energy:-1118.30721663

C -3.139977 0.954037 -2.941412
C -2.352234 2.100304 -2.865222
C -1.524754 2.241874 -1.749604
C -1.503795 1.254338 -0.768511
C -2.366862 0.126448 -0.804251
C -3.173819 -0.014111 -1.948679
N -0.570821 1.280084 0.327793
C -1.243434 1.025193 1.674910
C -2.007842 -0.176167 1.643951
C -2.462125 -0.723216 0.383812
C -2.417612 -0.807245 2.851406
C -3.112330 -1.998547 2.824863
C -3.460800 -2.594841 1.588450
C -3.155007 -1.946315 0.398378
O -1.037305 1.843214 2.584068
C 0.699158 0.827827 0.193014
C 1.393992 0.887592 -1.059088
C 2.709591 0.495612 -1.149339
C 3.436859 0.034489 -0.022519
C 2.758028 0.020635 1.221671
C 1.448843 0.422302 1.350282
F -3.935805 0.789025 -4.023662
C 4.893634 -0.418923 -0.104827
C 5.459927 -0.335192 -1.534775
C 5.756385 0.480468 0.818255
C 4.989409 -1.890027 0.376206
H -2.380707 2.840306 -3.656620
H -0.877954 3.108337 -1.651988
H -3.857821 -0.848294 -2.057927
H -2.127689 -0.350801 3.793393
H -3.377992 -2.491565 3.756384
H -3.985573 -3.545267 1.569805
H -3.473668 -2.390590 -0.541337
H 0.867971 1.224394 -1.942745
H 3.193499 0.548842 -2.116428
H 3.284753 -0.298589 2.114223
H 0.982649 0.450521 2.324624
H 6.499669 -0.677947 -1.532416
H 4.904368 -0.971206 -2.232848
H 5.452382 0.690834 -1.919084
H 6.802003 0.156467 0.769743
H 5.437491 0.425088 1.863725
H 5.708665 1.528337 0.502408
H 4.392169 -2.551523 -0.260847
H 6.032539 -2.222302 0.330686
H 4.645137 -2.009350 1.408238

32 Energy:-1118.30599352

C -3.139878 0.861577 -2.963718
C -2.377474 2.026531 -2.913023
C -1.554235 2.210108 -1.800559
C -1.512026 1.246360 -0.796779
C -2.355275 0.102645 -0.804183
C -3.155842 -0.081888 -1.948026
N -0.569565 1.316696 0.295183
C -1.240721 1.084105 1.645831
C -1.999425 -0.120398 1.650150
C -2.446826 -0.710479 0.407012
C -2.403134 -0.718622 2.876812
C -3.087841 -1.915474 2.886816
C -3.431428 -2.552026 1.668498
C -3.129999 -1.938547 0.459424
O -1.021254 1.916565 2.538003
C 0.691185 0.826594 0.165159
C 1.431572 1.000448 -1.049606
C 2.747077 0.606507 -1.134464
C 3.430907 0.034044 -0.032827
C 2.710681 -0.084000 1.183190
C 1.402929 0.323498 1.306539
F -3.930033 0.654112 -4.043323
C 4.885987 -0.425111 -0.107662
C 5.498496 -0.225403 -1.506691
C 5.724413 0.383726 0.916202
C 4.956232 -1.932785 0.248554
H -2.421106 2.748000 -3.720652
H -0.927560 3.093102 -1.722132
H -3.823754 -0.931405 -2.037026
H -2.116879 -0.231167 3.804288
H -3.349858 -2.382114 3.832834
H -3.948875 -3.506605 1.678983
H -3.443796 -2.414654 -0.466195
H 0.938891 1.430592 -1.911957
H 3.265774 0.753181 -2.073397
H 3.204843 -0.488829 2.059469
H 0.906985 0.264188 2.264664
H 6.534029 -0.580525 -1.501692
H 4.958880 -0.791524 -2.274038
H 5.514080 0.829925 -1.800713
H 6.768524 0.054160 0.873425
H 5.370978 0.242518 1.942297
H 5.694791 1.455144 0.689945
H 4.374498 -2.532081 -0.460320
H 5.998043 -2.269402 0.205935
H 4.580181 -2.136741 1.255929

33 Energy:-1118.3047836

C -3.146048 0.756185 -2.981849
C -2.400431 1.933168 -2.966812
C -1.578504 2.160610 -1.861543
C -1.521252 1.228751 -0.828828
C -2.352456 0.075732 -0.798710
C -3.149412 -0.154879 -1.937547
N -0.566929 1.339899 0.254052
C -1.233322 1.141694 1.610323
C -1.994004 -0.060016 1.659628
C -2.441190 -0.694651 0.438508
C -2.392714 -0.616066 2.907832
C -3.074618 -1.813096 2.961614
C -3.419261 -2.492625 1.766453
C -3.121375 -1.922155 0.536021
O -1.005011 2.000844 2.473984
C 0.687547 0.818478 0.125618
C 1.476774 1.112580 -1.033141
C 2.794734 0.722474 -1.109129
C 3.431286 0.034149 -0.048018
C 2.662472 -0.203401 1.120905
C 1.354246 0.204779 1.236707
F -3.933309 0.504688 -4.054542
C 4.886394 -0.425943 -0.111577
C 5.557147 -0.083686 -1.455081
C 5.685007 0.262302 1.026038
C 4.935188 -1.963605 0.084647
H -2.455006 2.629879 -3.795210
H -0.966062 3.055541 -1.810551
H -3.806326 -1.015336 -1.999435
H -2.104741 -0.096171 3.816990
H -3.333673 -2.246981 3.923862
H -3.934588 -3.447370 1.811653
H -3.435316 -2.432040 -0.371354
H 1.019537 1.638235 -1.861730
H 3.351684 0.968995 -2.004226
H 3.119242 -0.703490 1.967718
H 0.819153 0.040819 2.161288
H 6.590168 -0.445993 -1.445625
H 5.047644 -0.560261 -2.299889
H 5.588960 0.996800 -1.633674
H 6.729025 -0.068112 0.988855
H 5.291011 0.013794 2.016346
H 5.667485 1.351969 0.915431
H 4.382954 -2.479216 -0.708572
H 5.976631 -2.302330 0.051434
H 4.514447 -2.270397 1.047291

34 Energy:-1118.30392108

C -3.157155 0.640022 -2.995569
C -2.415891 1.819519 -3.026253
C -1.593492 2.090789 -1.931356
C -1.532043 1.200648 -0.862726
C -2.360986 0.046968 -0.787140
C -3.156509 -0.230001 -1.917134
N -0.564897 1.349963 0.207005
C -1.220531 1.190777 1.570852
C -1.990863 -0.000691 1.671901
C -2.447894 -0.676845 0.477079
C -2.383219 -0.508315 2.942605
C -3.071388 -1.698604 3.044610
C -3.427139 -2.419087 1.876692
C -3.133780 -1.896512 0.624199
O -0.984789 2.083909 2.396991
C 0.689694 0.810135 0.077461
C 1.529709 1.232260 -1.001089
C 2.851120 0.846832 -1.065523
C 3.437093 0.036344 -0.065885
C 2.615379 -0.331340 1.032338
C 1.305005 0.073850 1.138392
F -3.943925 0.344361 -4.057372
C 4.893130 -0.422407 -0.116398
C 5.625844 0.072967 -1.377193
C 5.638429 0.119737 1.131050
C 4.930530 -1.972863 -0.103382
H -2.473666 2.484318 -3.880230
H -0.984978 2.989523 -1.915826
H -3.809228 -1.095344 -1.946408
H -2.085595 0.042521 3.830261
H -3.326734 -2.095827 4.023489
H -3.947191 -3.368728 1.959954
H -3.455951 -2.438585 -0.261324
H 1.110549 1.860326 -1.777344
H 3.448624 1.197666 -1.897430
H 3.031776 -0.933670 1.832207
H 0.725068 -0.199662 2.009157
H 6.657130 -0.294162 -1.363561
H 5.155673 -0.296641 -2.295197
H 5.666348 1.166814 -1.424270
H 6.682281 -0.212263 1.104156
H 5.197070 -0.241181 2.065213
H 5.628621 1.214957 1.150042
H 4.414710 -2.385991 -0.977018
H 5.971874 -2.312453 -0.129315
H 4.465376 -2.389487 0.795447

35 Energy:-1118.30411319

C -3.174569 0.497772 -3.005182
C -2.418749 1.664790 -3.097754
C -1.594499 1.982762 -2.017398
C -1.546072 1.152676 -0.900247
C -2.389729 0.013043 -0.765679
C -3.184723 -0.314318 -1.882178
N -0.565813 1.338193 0.149387
C -1.198689 1.223353 1.525322
C -1.992784 0.056991 1.689393
C -2.477691 -0.656396 0.528307
C -2.377755 -0.389349 2.984828
C -3.089009 -1.559949 3.143750
C -3.473746 -2.319217 2.010264
C -3.185789 -1.854570 0.733457
O -0.945688 2.156511 2.301237
C 0.703205 0.806761 0.019096
C 1.593430 1.367211 -0.944351
C 2.919966 0.987738 -0.994099
C 3.452835 0.046465 -0.087066
C 2.575072 -0.464320 0.905532
C 1.260376 -0.065947 0.999545
F -3.962374 0.154435 -4.050827
C 4.910473 -0.410939 -0.123528
C 5.712409 0.260044 -1.254238
C 5.585573 -0.066147 1.229197
C 4.947534 -1.946237 -0.339368
H -2.467493 2.283863 -3.985936
H -0.975201 2.873350 -2.049476
H -3.843040 -1.175738 -1.870162
H -2.056303 0.190600 3.845257
H -3.340246 -1.911746 4.140840
H -4.011509 -3.253905 2.138181
H -3.530802 -2.426278 -0.124261
H 1.214201 2.103800 -1.642501
H 3.559205 1.448959 -1.736397
H 2.948099 -1.178022 1.632073
H 0.632748 -0.453505 1.791369
H 6.742122 -0.111502 -1.236767
H 5.295873 0.032528 -2.241775
H 5.752784 1.348688 -1.137167
H 6.629524 -0.398585 1.210844
H 5.093568 -0.557937 2.074122
H 5.574237 1.014206 1.410050
H 4.483750 -2.220428 -1.293218
H 5.988554 -2.287785 -0.355193
H 4.429929 -2.487373 0.458875

36 Energy:-1118.3473684

C -3.034141 -0.079622 -2.943213
C -1.673812 -0.298454 -3.186696
C -0.840128 -0.415597 -2.082630
C -1.344838 -0.275065 -0.763404
C -2.773209 -0.114755 -0.528159
C -3.586607 -0.003560 -1.663917
N -0.486359 -0.226824 0.309182
C -0.954317 -0.551343 1.683076
C -2.353705 -0.434155 1.906106
C -3.269983 -0.111490 0.828561
C -2.862772 -0.583512 3.225083
C -4.198839 -0.384616 3.499128
C -5.095256 -0.021095 2.453956
C -4.625812 0.101182 1.154064
O -0.077262 -0.869843 2.490520
C 0.928559 -0.149913 0.102480
C 1.499456 1.087664 -0.212698
C 2.879866 1.201419 -0.369130
C 3.730504 0.094724 -0.211796
C 3.131196 -1.136404 0.114908
C 1.756186 -1.266300 0.280412
F -3.861244 0.037958 -3.991800
C 5.256917 0.184870 -0.378885
C 5.724794 1.609433 -0.731932
C 5.943861 -0.236587 0.942656
C 5.704905 -0.766702 -1.514715
H -1.303815 -0.384715 -4.201192
H 0.217124 -0.601693 -2.221880
H -4.657877 0.130373 -1.581735
H -2.165658 -0.840663 4.016764
H -4.567800 -0.496775 4.514837
H -6.143772 0.157165 2.671026
H -5.329457 0.370700 0.371756
H 0.860970 1.959265 -0.322062
H 3.288210 2.177158 -0.605148
H 3.748664 -2.019891 0.246715
H 1.319120 -2.224717 0.536967
H 6.814797 1.619532 -0.841324
H 5.294461 1.958471 -1.677512
H 5.464728 2.330761 0.051037
H 7.033770 -0.182977 0.834282
H 5.687237 -1.261997 1.228248
H 5.650694 0.425578 1.765288
H 5.240174 -0.486882 -2.467203
H 6.793118 -0.717405 -1.640178
H 5.440546 -1.808154 -1.303688

37 Energy:-1118.35426345

C -3.015167 -0.320321 -2.944000
C -1.633732 -0.270270 -3.176447
C -0.800716 -0.200753 -2.061355
C -1.333997 -0.181133 -0.749281
C -2.781368 -0.238008 -0.521572
C -3.591590 -0.306387 -1.677469
N -0.492374 -0.105095 0.337616
C -0.951747 -0.097141 1.716893
C -2.371312 -0.156913 1.930966
C -3.290467 -0.223560 0.811626
C -2.864258 -0.148016 3.249434
C -4.223500 -0.200267 3.508073
C -5.139681 -0.264187 2.418185
C -4.682786 -0.275280 1.116982
O -0.081322 -0.038348 2.586243
C 0.938492 -0.041471 0.148239
C 1.567683 1.192899 0.012718
C 2.953051 1.248505 -0.160961
C 3.734956 0.085273 -0.205483
C 3.068199 -1.146008 -0.068666
C 1.688276 -1.217889 0.105427
F -3.834250 -0.386617 -4.005260
C 5.262728 0.113257 -0.391787
C 5.809691 1.546566 -0.530729
C 5.939715 -0.546353 0.833640
C 5.638786 -0.673645 -1.670175
H -1.244513 -0.285718 -4.187021
H 0.271594 -0.159907 -2.195720
H -4.670572 -0.350112 -1.609313
H -2.146151 -0.098392 4.062320
H -4.589267 -0.192557 4.530128
H -6.207713 -0.304677 2.612309
H -5.408013 -0.324902 0.311135
H 0.981249 2.105984 0.049054
H 3.415503 2.223681 -0.259298
H 3.631540 -2.073854 -0.094843
H 1.194156 -2.178629 0.213492
H 6.896840 1.511827 -0.661575
H 5.388415 2.062217 -1.401246
H 5.603400 2.150970 0.359810
H 7.029444 -0.539193 0.711668
H 5.625483 -1.587296 0.963009
H 5.696848 -0.004299 1.754737
H 5.177869 -0.224386 -2.557462
H 6.726138 -0.665890 -1.811471
H 5.317632 -1.719158 -1.616412

12.2.6 Computed CT emission energies of compound 4b (B3LYP/6-31G* PCM CH_2Cl_2)

```
Excitation energies and oscillator strengths:
Excited State   1:      Singlet-A      1.9127 eV  648.22 nm  f=0.0470  <S**2>=0.000
      91 -> 92         0.70478
 This state for optimization and/or second-order correction.
 Total Energy, E(TD-HF/TD-DFT) =  -1118.23951628
 Copying the excited state density for this state as the 1-particle RhoCI density.

 Excited State   2:      Singlet-A      3.1824 eV  389.60 nm  f=0.0109  <S**2>=0.000
      90 -> 92        -0.25490
      91 -> 93         0.65333

 Excited State   3:      Singlet-A      3.2414 eV  382.50 nm  f=0.0390  <S**2>=0.000
      86 -> 92        -0.12675
      88 -> 92        -0.14186
      90 -> 92         0.61658
      91 -> 93         0.26056

 Excited State   4:      Singlet-A      3.6103 eV  343.41 nm  f=0.0164  <S**2>=0.000
      88 -> 92        -0.12574
      89 -> 92         0.67128
      90 -> 92        -0.12053

 Excited State   5:      Singlet-A      3.7648 eV  329.32 nm  f=0.0615  <S**2>=0.000
      86 -> 92         0.19694
      88 -> 92         0.61264
      89 -> 92         0.18531
      90 -> 92         0.14326

 Excited State   6:      Singlet-A      4.0368 eV  307.13 nm  f=0.0867  <S**2>=0.000
      91 -> 94         0.68666
```

12.2.7 Computed excitations energies of the triplet state of compound 4b (B3LYP/6-31G* PCM CH_2Cl_2)

```
Excitation energies and oscillator strengths:
Excited State   1:      Triplet-A      3.0101 eV  411.89 nm  f=0.0000  <S**2>=2.000
      85 -> 97         0.10588
      87 -> 93         0.16727
      90 -> 92        -0.30618
      90 -> 93         0.10126
      91 -> 92         0.54174
      91 -> 93        -0.17152
      91 -> 96        -0.10978
 This state for optimization and/or second-order correction.
 Total Energy, E(TD-HF/TD-DFT) =  -1118.25159796
 Copying the excited state density for this state as the 1-particle RhoCI density.
```

```
Excited State   2:      Triplet-A      3.3244 eV  372.96 nm  f=0.0000  <S**2>=2.000
      90 -> 92         0.34424
      90 -> 93        -0.12593
      90 -> 96         0.12796
      91 -> 92         0.41257
      91 -> 93         0.34429
      91 -> 96         0.17954

Excited State   3:      Triplet-A      3.6419 eV  340.43 nm  f=0.0000  <S**2>=2.000
      87 -> 92         0.11976
      90 -> 92        -0.38699
      91 -> 93         0.53484

Excited State   4:      Triplet-A      3.7162 eV  333.63 nm  f=0.0000  <S**2>=2.000
      88 -> 94        -0.15064
      88 -> 95        -0.37838
      89 -> 94         0.50678
      89 -> 95        -0.14426

Excited State   5:      Triplet-A      3.7925 eV  326.92 nm  f=0.0000  <S**2>=2.000
      85 -> 92        -0.17018
      87 -> 92         0.20686
      87 -> 93         0.45692
      88 -> 93         0.15014
      90 -> 96         0.15852
      90 -> 97         0.11589
      91 -> 92        -0.11493
      91 -> 96         0.26925
      91 -> 97        -0.14285

Excited State   6:      Triplet-A      4.1868 eV  296.13 nm  f=0.0000  <S**2>=2.000
      87 -> 92        -0.26373
      87 -> 93        -0.20543
      88 -> 92        -0.10769
      90 -> 92        -0.26679
      90 -> 93         0.16290
      90 -> 96         0.12045
      91 -> 96         0.47424

Excited State   7:      Triplet-A      4.2503 eV  291.71 nm  f=0.0000  <S**2>=2.000
      86 -> 92         0.43003
      86 -> 93         0.40480
      86 -> 98        -0.12791
      89 -> 92         0.27763
      89 -> 93         0.19799

Excited State   8:      Triplet-A      4.3134 eV  287.44 nm  f=0.0000  <S**2>=2.000
```

84 -> 92 0.10730
85 -> 92 0.10157
85 -> 93 0.10470
87 -> 92 -0.29051
87 -> 93 0.11311
88 -> 93 0.10127
90 -> 92 0.16063
90 -> 93 0.48461
90 -> 96 -0.12845
91 -> 93 0.14713
91 -> 96 -0.12897

Excited State 9: Triplet-A 4.4802 eV 276.74 nm f=0.0000 <S**2>=2.000
85 -> 92 0.26727
87 -> 92 0.41218
87 -> 93 -0.15043
88 -> 92 0.19432
90 -> 93 0.29055
90 -> 96 -0.10981
90 -> 97 -0.14745
91 -> 96 0.19142
91 -> 97 0.10545

Excited State 10: Triplet-A 4.5941 eV 269.87 nm f=0.0000 <S**2>=2.000
86 -> 92 -0.12861
88 -> 94 -0.23123
88 -> 95 0.11180
89 -> 92 0.41837
89 -> 93 -0.11936
89 -> 94 0.11674
89 -> 95 0.42202

Excited State 11: Triplet-A 4.6513 eV 266.56 nm f=0.0000 <S**2>=2.000
87 -> 95 -0.10184
88 -> 92 0.19652
88 -> 94 0.21348
88 -> 95 0.41499
89 -> 94 0.40269
89 -> 95 -0.14951

Excited State 12: Triplet-A 4.6753 eV 265.19 nm f=0.0000 <S**2>=2.000
85 -> 92 0.31921
85 -> 93 -0.14746
87 -> 92 -0.20030
87 -> 93 0.31902
90 -> 93 -0.23199
90 -> 96 -0.22013
90 -> 97 -0.15790

91 -> 95 0.11461
91 -> 96 0.19776
91 -> 97 0.15316

Excited State 13: Triplet-A 4.7898 eV 258.85 nm f=0.0000 <S**2>=2.000
86 -> 92 -0.14471
86 -> 93 -0.15536
88 -> 94 0.26511
88 -> 95 -0.15036
89 -> 92 0.45980
89 -> 94 -0.11786
89 -> 95 -0.24099
91 -> 94 -0.21200

Excited State 14: Triplet-A 4.8417 eV 256.08 nm f=0.0000 <S**2>=2.000
89 -> 92 0.13334
89 -> 95 -0.14126
91 -> 94 0.64517
91 -> 95 -0.12366

Excited State 15: Triplet-A 5.0099 eV 247.48 nm f=0.0000 <S**2>=2.000
86 -> 92 0.17722
88 -> 92 0.21605
88 -> 94 0.12382
89 -> 93 -0.26066
91 -> 94 0.10570
91 -> 95 0.51079
91 -> 96 -0.13520

12.2.8 Computed emission energies of the triplet state of compound 4b (B3LYP/6-31G* PCM CH_2Cl_2)

Excitation energies and oscillator strengths:
Excited State 1: Triplet-A 2.3456 eV 528.58 nm f=0.0000 <S**2>=2.000
85 -> 97 0.10152
86 -> 93 -0.10479
90 -> 92 0.21212
91 -> 92 0.64939
This state for optimization and/or second-order correction.
Total Energy, E(TD-HF/TD-DFT) = -1118.26327667
Copying the excited state density for this state as the 1-particle RhoCI density.

Excited State 2: Triplet-A 3.1828 eV 389.55 nm f=0.0000 <S**2>=2.000
90 -> 92 0.51628
90 -> 96 0.10895
91 -> 92 -0.24662
91 -> 93 -0.37147

Excited State   3:   Triplet-A   3.4247 eV 362.02 nm f=0.0000 <S**2>=2.000
86 -> 92   -0.10597
90 -> 92   0.38090
91 -> 93   0.56566

Excited State   4:   Triplet-A   3.7138 eV 333.84 nm f=0.0000 <S**2>=2.000
87 -> 94   -0.10541
88 -> 94   -0.15823
88 -> 95   -0.39476
89 -> 94   0.51011
89 -> 95   -0.14091

Excited State   5:   Triplet-A   3.8304 eV 323.68 nm f=0.0000 <S**2>=2.000
85 -> 92   -0.29762
86 -> 93   0.42297
88 -> 93   0.10492
90 -> 93   0.17288
90 -> 96   0.19446
90 -> 97   -0.11426
91 -> 96   -0.19399
91 -> 97   -0.26091

Excited State   6:   Triplet-A   4.1438 eV 299.20 nm f=0.0000 <S**2>=2.000
86 -> 92   0.56717
86 -> 93   0.22538
88 -> 92   0.12676
90 -> 92   0.10113
90 -> 93   -0.13560
91 -> 96   0.21941

Excited State   7:   Triplet-A   4.2011 eV 295.12 nm f=0.0000 <S**2>=2.000
87 -> 92   0.33901
87 -> 93   0.33818
89 -> 92   0.46325
89 -> 93   0.18248

Excited State   8:   Triplet-A   4.2538 eV 291.46 nm f=0.0000 <S**2>=2.000
85 -> 92   -0.14720
86 -> 92   -0.29560
86 -> 93   0.13705
88 -> 92   -0.10011
90 -> 93   -0.11847
90 -> 96   -0.15604
91 -> 95   0.10710
91 -> 96   0.51774

Excited State   9:   Triplet-A   4.3538 eV 284.77 nm f=0.0000 <S**2>=2.000
84 -> 92   0.14885

85 -> 92 0.33692
86 -> 93 0.29478
88 -> 93 0.10826
90 -> 93 0.34765
90 -> 96 -0.21878
90 -> 97 0.12630
91 -> 97 0.13236
91 -> 98 -0.13384

Excited State 10: Triplet-A 4.4017 eV 281.67 nm f=0.0000 <S**2>=2.000
87 -> 92 -0.29994
87 -> 93 -0.30306
89 -> 92 0.50040
89 -> 93 -0.13691
89 -> 95 0.16320

Excited State 11: Triplet-A 4.4843 eV 276.49 nm f=0.0000 <S**2>=2.000
85 -> 92 -0.17709
86 -> 92 0.16285
86 -> 93 -0.30684
90 -> 93 0.48243
91 -> 95 0.10124
91 -> 96 0.20630
91 -> 97 -0.14723

Excited State 12: Triplet-A 4.5892 eV 270.16 nm f=0.0000 <S**2>=2.000
86 -> 92 -0.12169
88 -> 92 0.47837
88 -> 94 0.16095
88 -> 95 0.28637
89 -> 94 0.32158
89 -> 95 -0.13908

Excited State 13: Triplet-A 4.6967 eV 263.98 nm f=0.0000 <S**2>=2.000
87 -> 93 0.11525
88 -> 94 -0.31357
88 -> 95 0.13418
89 -> 92 -0.14496
89 -> 94 0.13570
89 -> 95 0.40627
90 -> 94 0.11614
91 -> 94 -0.34937

Excited State 14: Triplet-A 4.7226 eV 262.54 nm f=0.0000 <S**2>=2.000
87 -> 92 -0.14369
87 -> 93 0.14233
88 -> 94 -0.16691
89 -> 95 0.23726

91 -> 94 0.56854

Excited State 15: Triplet-A 4.7427 eV 261.42 nm f=0.0000 <S**2>=2.000

87 -> 92	0.49202
87 -> 93	-0.30361
88 -> 92	0.10807
88 -> 94	-0.13271
89 -> 93	-0.27570
89 -> 95	0.14119
91 -> 94	0.14229

12.3 Quantum chemical calculation data of 5-(4-(*tert*-Butyl)phenyl)-2-fluoro-[1,3]dioxolo[4,5-j]phenanthridin-6(5*H*)-one (5b)

12.3.1 Computed xyz-coordinates of compound 5b (B3LYP/6-31G* PCM CH_2Cl_2)

C	-1.410243	3.870927	-0.002967
C	-0.029083	3.698394	0.003153
C	0.478388	2.40664	0.008594
C	-0.379107	1.290882	0.007812
C	-1.784442	1.480918	0.001308
C	-2.279586	2.799637	-0.003903
N	0.154431	-0.011324	0.013327
C	-0.625167	-1.166645	0.01141
C	-2.085284	-0.973382	0.00545
C	-2.665169	0.313913	0.000234
C	-2.875991	-2.148834	0.004769
C	-4.236665	-1.989939	-0.001143
C	-4.823517	-0.717121	-0.006537
C	-4.080093	0.437503	-0.006033
C	1.587173	-0.203402	0.018873
O	-0.097695	-2.281387	0.014677
F	-1.916235	5.127129	-0.008149
C	2.27709	-0.291909	1.224838
C	3.662209	-0.474283	1.224679
C	4.389239	-0.571697	0.029501
C	3.665906	-0.4779	-1.173505
C	2.285361	-0.296491	-1.186251
O	-5.214474	-2.947407	-0.002845
C	-6.468379	-2.248079	-0.011378
O	-6.182641	-0.837545	-0.011859
C	5.915207	-0.771921	-0.006473
C	6.528125	-0.858736	1.404111
C	6.571037	0.418683	-0.74629
C	6.243478	-2.084716	-0.757222
H	0.628777	4.560687	0.003635
H	1.550457	2.259064	0.013614
H	-3.343232	3.002552	-0.008706
H	-2.395247	-3.118822	0.008846
H	-4.576908	1.399053	-0.010459
H	1.733904	-0.221408	2.162679
H	4.168785	-0.541354	2.180523
H	4.184896	-0.548838	-2.124762
H	1.747109	-0.229425	-2.127155
H	-7.024485	-2.504791	-0.917888
H	-7.035145	-2.501992	0.889246
H	6.124142	-1.703409	1.973611
H	7.611381	-1.001927	1.324139
H	6.357714	0.057329	1.981112

H	6.207523	0.51043	-1.775204
H	7.658757	0.285529	-0.787197
H	6.3637	1.363336	-0.230277
H	7.328842	-2.236831	-0.794728
H	5.796271	-2.947994	-0.251227
H	5.87341	-2.070011	-1.787718
C	-1.410243	3.870927	-0.002967
C	-0.029083	3.698394	0.003153
C	0.478388	2.40664	0.008594
C	-0.379107	1.290882	0.007812
C	-1.784442	1.480918	0.001308
C	-2.279586	2.799637	-0.003903
N	0.154431	-0.011324	0.013327
C	-0.625167	-1.166645	0.01141
C	-2.085284	-0.973382	0.00545
C	-2.665169	0.313913	0.000234
C	-2.875991	-2.148834	0.004769
C	-4.236665	-1.989939	-0.001143
C	-4.823517	-0.717121	-0.006537
C	-4.080093	0.437503	-0.006033
C	1.587173	-0.203402	0.018873

SCF Done: E(RB3LYP) = -1306.89399943 A.U. after   15 cycles

Zero-point correction = 0.388088 (Hartree/Particle)

Thermal correction to Energy = 0.412225

Thermal correction to Enthalpy = 0.413170

Thermal correction to Gibbs Free Energy = 0.332730

Sum of electronic and zero-point Energies = -1306.505912

Sum of electronic and thermal Energies = -1306.481774

Sum of electronic and thermal Enthalpies = -1306.480830

Sum of electronic and thermal Free Energies = -1306.561270

12.3.2 Computed excitations energies of compound 5b (B3LYP/6-31G* PCM CH_2Cl_2)

Excitation energies and oscillator strengths:

Excited State   1:   Singlet-A   3.8837 eV 319.24 nm f=0.2547 <S**2>=0.000
    101 ->103   -0.12483
    101 ->104   0.15190
    102 ->103   0.66866
 This state for optimization and/or second-order correction.
 Total Energy, E(TD-HF/TD-DFT) = -1306.75127474
 Copying the excited state density for this state as the 1-particle RhoCI density.

 Excited State   2:   Singlet-A   4.1247 eV 300.59 nm f=0.0114 <S**2>=0.000
     99 ->103   -0.15872

101 ->103 -0.45287
102 ->104 0.50483

Excited State 3: Singlet-A 4.3309 eV 286.28 nm f=0.1849 <S**2>=0.000
99 ->103 -0.17901
101 ->103 0.49478
102 ->103 0.10048
102 ->104 0.40324
102 ->107 -0.14422

Excited State 4: Singlet-A 4.6111 eV 268.88 nm f=0.0021 <S**2>=0.000
97 ->103 0.36078
97 ->104 0.24410
100 ->103 0.51405
100 ->104 0.16493

Excited State 5: Singlet-A 4.6194 eV 268.40 nm f=0.1603 <S**2>=0.000
99 ->103 -0.23227
101 ->104 0.62038
102 ->103 -0.15040
102 ->104 -0.13756

Excited State 6: Singlet-A 4.7659 eV 260.15 nm f=0.0006 <S**2>=0.000
101 ->105 -0.22574
102 ->105 0.65652

Excited State 7: Singlet-A 4.8598 eV 255.12 nm f=0.0003 <S**2>=0.000
97 ->103 -0.41837
97 ->104 -0.26062
100 ->103 0.47480
100 ->104 -0.12388

Excited State 8: Singlet-A 4.9348 eV 251.25 nm f=0.0154 <S**2>=0.000
99 ->103 -0.15076
101 ->106 -0.17984
102 ->105 0.10066
102 ->106 0.64876

Excited State 9: Singlet-A 5.0155 eV 247.20 nm f=0.5248 <S**2>=0.000
98 ->103 0.12215
99 ->103 0.46831
101 ->104 0.19946
102 ->104 0.17832
102 ->106 0.15592
102 ->107 0.35771

Excited State 10: Singlet-A 5.0728 eV 244.41 nm f=0.0712 <S**2>=0.000
98 ->103 0.68581

Excited State 11: Singlet-A 5.1538 eV 240.57 nm f=0.1814 <S**2>=0.000
99 ->103 -0.34566
99 ->104 -0.10225
100 ->104 0.12441
101 ->104 -0.15840
102 ->107 0.51241
102 ->108 -0.11447

Excited State 12: Singlet-A 5.1572 eV 240.41 nm f=0.0075 <S**2>=0.000
97 ->103 -0.35225
97 ->104 0.20599
100 ->104 0.54349
102 ->107 -0.11830

Excited State 13: Singlet-A 5.2368 eV 236.76 nm f=0.0033 <S**2>=0.000
101 ->105 0.64714
102 ->105 0.22651

Excited State 14: Singlet-A 5.3579 eV 231.41 nm f=0.0010 <S**2>=0.000
97 ->103 -0.19109
97 ->104 0.45115
98 ->105 0.26729
100 ->104 -0.25175
100 ->105 0.10049
100 ->106 0.27447
101 ->106 0.10500

Excited State 15: Singlet-A 5.3863 eV 230.19 nm f=0.0008 <S**2>=0.000
97 ->104 -0.11751
99 ->104 -0.17382
101 ->106 0.62518
102 ->106 0.17850

12.3.3 Computed emission energies of compound 5b (B3LYP/6-31G* PCM CH_2Cl_2)

Excitation energies and oscillator strengths:

Excited State 1: Singlet-A 3.2457 eV 382.00 nm f=0.2638 <S**2>=0.000
102 ->103 0.68944
This state for optimization and/or second-order correction.
Total Energy, E(TD-HF/TD-DFT) = -1306.76038558
Copying the excited state density for this state as the 1-particle RhoCI density.

Excited State 2: Singlet-A 3.6822 eV 336.72 nm f=0.0717 <S**2>=0.000
101 ->103 0.59994
102 ->104 -0.34893

Excited State 3: Singlet-A 3.9264 eV 315.77 nm f=0.1130 <S**2>=0.000

```
    99 ->103        0.12522
   101 ->103         0.31813
   102 ->104         0.58161
   102 ->105        -0.11697

 Excited State   4:      Singlet-A      4.2050 eV  294.85 nm  f=0.0489  <S**2>=0.000
    97 ->103        0.18363
    98 ->103        0.35434
   100 ->103         0.32774
   101 ->104         0.44586

 Excited State   5:      Singlet-A      4.2749 eV  290.03 nm  f=0.0824  <S**2>=0.000
    97 ->103       -0.22253
    99 ->103        0.38299
   100 ->103       -0.36175
   101 ->104         0.34877
   102 ->105         0.11137

 Excited State   6:      Singlet-A      4.4028 eV  281.61 nm  f=0.0172  <S**2>=0.000
    97 ->103       -0.15144
    98 ->103       -0.29262
    99 ->103        0.37202
   100 ->103         0.47391
```

12.3.4 Potential scan of compound 5b ground state (B3LYP/6-31G* PCM CH_2Cl_2)

```
 D(4,7,15,18)
0  -1306.89399943 89.9445
8  -1306.89382614 99.9444
13  -1306.89331088 109.9445
18  -1306.89238581 119.9444
23  -1306.89090604 129.9444
27  -1306.88870497 139.9443
32  -1306.8855944 149.9443
38  -1306.88154428 159.9444
49  -1306.87726563 169.9444
54  -1306.87347219 179.9443
59  -1306.87035239 -170.0556
65  -1306.86805809 -160.0557
71  -1306.86685238 -150.0555
86  -1306.88867426 -140.0554
90  -1306.89087476 -130.0554
95  -1306.89234323 -120.0554
99  -1306.89327627 -110.0555
103  -1306.89380241 -100.0554
108  -1306.8940003 -90.0555
114  -1306.89382569 -80.0556
119  -1306.89326886 -70.0556
```

125 -1306.89226914 -60.0556
131 -1306.89063978 -50.0556
137 -1306.88825423 -40.0556
142 -1306.88500122 -30.0557
150 -1306.8809055 -20.0557
156 -1306.87643617 -10.0556
163 -1306.87246673 -0.0556
169 -1306.86940084 9.9444
176 -1306.86743765 19.9444
201 -1306.88495076 29.9445
206 -1306.88821583 39.9446
210 -1306.89061252 49.9446
215 -1306.89224062 59.9446
219 -1306.89327076 69.9446
223 -1306.89381672 79.9446
228 -1306.89399056 89.9445

Progress of structural optimization and emission calculation

-1306.893999
-1306.893695
-1306.893805
-1306.893815
-1306.893822
-1306.893825
-1306.893826
-1306.893826
-1306.893826
-1306.893195
-1306.89331
-1306.893311
-1306.893311
-1306.893311
-1306.892268
-1306.892384
-1306.892386
-1306.892386
-1306.892386
-1306.890777
-1306.890902
-1306.890905
-1306.890906
-1306.890906
-1306.888526
-1306.888699
-1306.888704
-1306.888705
-1306.885289
-1306.885578

-1306.885593
-1306.885594
-1306.885594
-1306.881091
-1306.881485
-1306.881537
-1306.881544
-1306.881544
-1306.881544
-1306.876368
-1306.877125
-1306.877246
-1306.877259
-1306.877262
-1306.877262
-1306.877264
-1306.877265
-1306.877265
-1306.877266
-1306.877266
-1306.872586
-1306.87337
-1306.873468
-1306.873472
-1306.873472
-1306.869456
-1306.870315
-1306.870351
-1306.870352
-1306.870352
-1306.867138
-1306.86801
-1306.868055
-1306.868058
-1306.868058
-1306.868058
-1306.86582
-1306.866739
-1306.866839
-1306.86685
-1306.866852
-1306.866852
-1306.865988
-1306.867385
-1306.86871
-1306.868561
-1306.877175
-1306.882306

-1306.8748
-1306.887042
-1306.888008
-1306.888569
-1306.888652
-1306.888671
-1306.888674
-1306.888674
-1306.888674
-1306.890707
-1306.890868
-1306.890874
-1306.890875
-1306.892212
-1306.89234
-1306.892343
-1306.892343
-1306.892343
-1306.893161
-1306.893273
-1306.893276
-1306.893276
-1306.893686
-1306.8938
-1306.893802
-1306.893802
-1306.893863
-1306.893995
-1306.893999
-1306.894
-1306.894
-1306.893688
-1306.893823
-1306.893825
-1306.893826
-1306.893826
-1306.893826
-1306.893082
-1306.893266
-1306.893269
-1306.893269
-1306.893269
-1306.892038
-1306.892261
-1306.892269
-1306.892269
-1306.892269
-1306.892269

-1306.890355
-1306.890627
-1306.890639
-1306.89064
-1306.89064
-1306.89064
-1306.887889
-1306.888231
-1306.888254
-1306.888254
-1306.888254
-1306.888254
-1306.884569
-1306.884957
-1306.885
-1306.885001
-1306.885001
-1306.880299
-1306.880825
-1306.880887
-1306.880902
-1306.880905
-1306.880905
-1306.880905
-1306.880906
-1306.875555
-1306.876308
-1306.876419
-1306.876435
-1306.876436
-1306.876436
-1306.871282
-1306.872339
-1306.872438
-1306.872463
-1306.872466
-1306.872467
-1306.872467
-1306.868333
-1306.869287
-1306.86938
-1306.869399
-1306.869401
-1306.869401
-1306.866424
-1306.867317
-1306.867411
-1306.867435

-1306.867437
-1306.867438
-1306.867438
-1306.865984
-1306.866887
-1306.867093
-1306.867387
-1306.867604
-1306.662881
-1306.866165
-1306.86775
-1306.866168
-1306.772278
-1306.78212
-1306.868047
-1306.803526
-1306.871795
-1306.873924
-1306.873067
-1306.880097
-1306.878796
-1306.883171
-1306.884256
-1306.884863
-1306.884927
-1306.884948
-1306.884951
-1306.884951
-1306.887853
-1306.888191
-1306.888215
-1306.888216
-1306.888216
-1306.890305
-1306.8906
-1306.890612
-1306.890613
-1306.891983
-1306.892229
-1306.89224
-1306.892241
-1306.892241
-1306.893071
-1306.893267
-1306.893271
-1306.893271
-1306.893646
-1306.893815

-1306.893817
-1306.893817
-1306.893828
-1306.893988
-1306.89399
-1306.893991

Geometries of structural optimization

1 Energy:-1306.89399943
C -1.410243 3.870927 -0.002967
C -0.029083 3.698394 0.003153
C 0.478388 2.406640 0.008594
C -0.379107 1.290882 0.007812
C -1.784442 1.480918 0.001308
C -2.279586 2.799637 -0.003903
N 0.154431 -0.011324 0.013327
C -0.625167 -1.166645 0.011410
C -2.085284 -0.973382 0.005450
C -2.665169 0.313913 0.000234
C -2.875991 -2.148834 0.004769
C -4.236665 -1.989939 -0.001143
C -4.823517 -0.717121 -0.006537
C -4.080093 0.437503 -0.006033
C 1.587173 -0.203402 0.018873
O -0.097695 -2.281387 0.014677
F -1.916235 5.127129 -0.008149
C 2.277090 -0.291909 1.224838
C 3.662209 -0.474283 1.224679
C 4.389239 -0.571697 0.029501
C 3.665906 -0.477900 -1.173505
C 2.285361 -0.296491 -1.186251
O -5.214474 -2.947407 -0.002845
C -6.468379 -2.248079 -0.011378
O -6.182641 -0.837545 -0.011859
C 5.915207 -0.771921 -0.006473
C 6.528125 -0.858736 1.404111
C 6.571037 0.418683 -0.746290
C 6.243478 -2.084716 -0.757222
H 0.628777 4.560687 0.003635
H 1.550457 2.259064 0.013614
H -3.343232 3.002552 -0.008706
H -2.395247 -3.118822 0.008846
H -4.576908 1.399053 -0.010459
H 1.733904 -0.221408 2.162679
H 4.168785 -0.541354 2.180523
H 4.184896 -0.548838 -2.124762

H 1.747109 -0.229425 -2.127155
H -7.024485 -2.504791 -0.917888
H -7.035145 -2.501992 0.889246
H 6.124142 -1.703409 1.973611
H 7.611381 -1.001927 1.324139
H 6.357714 0.057329 1.981112
H 6.207523 0.510430 -1.775204
H 7.658757 0.285529 -0.787197
H 6.363700 1.363336 -0.230277
H 7.328842 -2.236831 -0.794728
H 5.796271 -2.947994 -0.251227
H 5.873410 -2.070011 -1.787718

2 Energy:-1306.89382614
C -1.421371 3.874030 -0.156736
C -0.040566 3.708718 -0.097978
C 0.470864 2.420975 -0.019118
C -0.381776 1.301513 -0.002020
C -1.787122 1.484898 -0.052408
C -2.286450 2.799608 -0.133296
N 0.156563 0.003565 0.081861
C -0.620553 -1.153332 0.127784
C -2.080794 -0.966116 0.083466
C -2.664171 0.315956 -0.012427
C -2.867765 -2.143159 0.130264
C -4.228284 -1.990982 0.079598
C -4.818536 -0.723377 -0.016028
C -4.078752 0.432652 -0.063314
C 1.589661 -0.184673 0.070193
O -0.091471 -2.265114 0.195526
F -1.931307 5.126109 -0.235381
C 2.278697 -0.396380 1.260986
C 3.662234 -0.587850 1.242468
C 4.389361 -0.573415 0.043389
C 3.666890 -0.360211 -1.144661
C 2.287135 -0.171654 -1.139328
O -5.202839 -2.951352 0.108041
C -6.458085 -2.259499 0.023922
O -6.176644 -0.850044 -0.050196
C 5.913888 -0.779937 -0.012065
C 6.526247 -1.000903 1.384047
C 6.578388 0.469045 -0.639193
C 6.232408 -2.020073 -0.881233
H 0.613910 4.573473 -0.111577
H 1.542578 2.279672 0.031673
H -3.350289 2.997058 -0.175896
H -2.384455 -3.109178 0.202249
H -4.578038 1.390066 -0.137171

H 1.735938 -0.413405 2.201346
H 4.167933 -0.751335 2.187084
H 4.185152 -0.344183 -2.098853
H 1.749515 -0.015004 -2.070001
H -6.987682 -2.570639 -0.881441
H -7.049276 -2.463634 0.921488
H 6.117183 -1.892294 1.873001
H 7.608651 -1.142543 1.290685
H 6.361201 -0.141237 2.043529
H 6.216233 0.657604 -1.655291
H 7.665174 0.332517 -0.692219
H 6.377453 1.363609 -0.038366
H 7.316636 -2.175609 -0.934434
H 5.779569 -2.923256 -0.456420
H 5.861287 -1.907596 -1.905282

3 Energy:-1306.89331088
C -1.437291 3.872870 -0.314796
C -0.058201 3.721444 -0.203136
C 0.458762 2.442612 -0.051350
C -0.385168 1.316615 -0.017148
C -1.789868 1.488062 -0.110534
C -2.295195 2.793743 -0.267082
N 0.160228 0.027585 0.142268
C -0.614737 -1.130592 0.225910
C -2.074757 -0.952963 0.149634
C -2.662241 0.318054 -0.028518
C -2.856899 -2.130498 0.244410
C -4.216805 -1.989292 0.159125
C -4.811108 -0.732351 -0.017782
C -4.076007 0.423620 -0.113966
C 1.593286 -0.158159 0.117918
O -0.085004 -2.237237 0.347455
F -1.952688 5.115781 -0.467202
C 2.277003 -0.496248 1.282458
C 3.658167 -0.700324 1.247685
C 4.389483 -0.576891 0.057470
C 3.672243 -0.243726 -1.105742
C 2.294333 -0.043504 -1.084800
O -5.187141 -2.952293 0.221749
C -6.443816 -2.273358 0.076173
O -6.167590 -0.868618 -0.071200
C 5.912151 -0.791459 -0.014349
C 6.518015 -1.151444 1.355438
C 6.589861 0.505970 -0.516610
C 6.222476 -1.944901 -0.998252
H 0.590697 4.590044 -0.230913
H 1.528890 2.314221 0.044674

H -3.358713 2.980530 -0.348087
H -2.370512 -3.088377 0.377711
H -4.578333 1.372602 -0.249612
H 1.732086 -0.599831 2.215569
H 4.159018 -0.961095 2.172865
H 4.192413 -0.143927 -2.053822
H 1.761571 0.202795 -1.998692
H -6.954966 -2.637391 -0.820003
H -7.050741 -2.430583 0.972695
H 6.100036 -2.082341 1.755087
H 7.599529 -1.292566 1.251619
H 6.357666 -0.358081 2.094385
H 6.232395 0.795072 -1.510533
H 7.675550 0.365225 -0.580323
H 6.395169 1.340254 0.167274
H 7.305461 -2.104407 -1.063965
H 5.760457 -2.880713 -0.663441
H 5.855359 -1.730844 -2.007447

4 Energy:-1306.89238581
C -1.455599 3.868520 -0.460631
C -0.081146 3.739064 -0.283615
C 0.441639 2.474646 -0.053228
C -0.389348 1.338903 -0.008728
C -1.791276 1.491459 -0.158322
C -2.303214 2.782533 -0.395471
N 0.165035 0.065008 0.234225
C -0.608390 -1.093727 0.357327
C -2.066840 -0.931500 0.232505
C -2.657551 0.320181 -0.042100
C -2.843035 -2.108873 0.371029
C -4.200126 -1.986141 0.231138
C -4.797416 -0.748146 -0.042713
C -4.068076 0.406951 -0.183231
C 1.597867 -0.118770 0.191054
O -0.080211 -2.191376 0.545789
F -1.976879 5.096580 -0.691702
C 2.284230 -0.570231 1.315467
C 3.661745 -0.790991 1.253091
C 4.388816 -0.577270 0.073334
C 3.669223 -0.136438 -1.051834
C 2.294751 0.080138 -1.004225
O -5.164667 -2.953010 0.318502
C -6.420686 -2.296251 0.089502
O -6.149903 -0.900734 -0.135637
C 5.907367 -0.807895 -0.027673
C 6.515865 -1.291352 1.302302
C 6.602099 0.517052 -0.423818

C 6.193827 -1.878654 -1.107671
H 0.559407 4.613514 -0.319875
H 1.507423 2.366053 0.098293
H -3.364881 2.952724 -0.523253
H -2.354437 -3.052578 0.576971
H -4.572497 1.340944 -0.394068
H 1.745399 -0.746317 2.240507
H 4.163005 -1.138747 2.149013
H 4.183973 0.033794 -1.992801
H 1.761978 0.405470 -1.892935
H -6.898632 -2.719785 -0.798791
H -7.056338 -2.403286 0.973428
H 6.086087 -2.246527 1.624668
H 7.594508 -1.438964 1.179282
H 6.371737 -0.561235 2.106858
H 6.243127 0.894154 -1.387240
H 7.685137 0.366251 -0.507031
H 6.424300 1.293587 0.329206
H 7.273790 -2.048129 -1.194822
H 5.719654 -2.832433 -0.849248
H 5.823684 -1.575085 -2.092566

5 Energy:-1306.89090604
C -1.472286 3.860135 -0.596362
C -0.106470 3.759603 -0.347896
C 0.420793 2.514072 -0.039692
C -0.393601 1.365937 0.008434
C -1.789808 1.493751 -0.202472
C -2.307259 2.765527 -0.519797
N 0.170263 0.112724 0.335246
C -0.602583 -1.045835 0.493517
C -2.057809 -0.903749 0.316221
C -2.649311 0.321332 -0.056281
C -2.828152 -2.079561 0.496585
C -4.179886 -1.981081 0.298418
C -4.777495 -0.769315 -0.074191
C -4.053790 0.383426 -0.257029
C 1.602750 -0.071099 0.271920
O -0.077976 -2.131150 0.749069
F -1.998287 5.068616 -0.905571
C 2.290800 -0.631571 1.346005
C 3.663573 -0.870377 1.252608
C 4.386294 -0.575931 0.088206
C 3.664919 -0.034396 -0.990856
C 2.295634 0.202653 -0.912516
O -5.138055 -2.951955 0.408068
C -6.390110 -2.325386 0.090137
O -6.123645 -0.943099 -0.209837

C 5.898988 -0.826571 -0.045239
C 6.509598 -1.419428 1.238754
C 6.616222 0.510711 -0.348557
C 6.154987 -1.818890 -1.204873
H 0.523159 4.641795 -0.387341
H 1.478525 2.430574 0.171907
H -3.364811 2.914348 -0.697994
H -2.339358 -3.003773 0.776695
H -4.558026 1.296796 -0.544581
H 1.759275 -0.874077 2.259008
H 4.164024 -1.299586 2.112973
H 4.173031 0.198169 -1.922135
H 1.764305 0.601691 -1.771204
H -6.828376 -2.808080 -0.788367
H -7.059232 -2.385487 0.953534
H 6.065401 -2.388568 1.492538
H 7.584396 -1.576061 1.094717
H 6.385010 -0.749582 2.097216
H 6.256418 0.965148 -1.277640
H 7.695360 0.346125 -0.453593
H 6.460073 1.232097 0.461914
H 7.230570 -2.002276 -1.315378
H 5.664072 -2.780004 -1.013566
H 5.782413 -1.435258 -2.160459

6 Energy:-1306.88870497
C -1.479697 3.845344 -0.725921
C -0.129535 3.781238 -0.393257
C 0.397546 2.559548 -0.002183
C -0.396626 1.396589 0.041887
C -1.781486 1.493216 -0.242737
C -2.300128 2.740462 -0.645380
N 0.174984 0.171109 0.459623
C -0.600283 -0.985017 0.652625
C -2.048678 -0.868750 0.409056
C -2.634474 0.319918 -0.072214
C -2.815042 -2.040677 0.629363
C -4.156285 -1.973837 0.359813
C -4.747322 -0.798122 -0.122553
C -4.027358 0.349796 -0.346219
C 1.607232 -0.015917 0.372941
O -0.085171 -2.053289 0.985601
F -2.006051 5.029097 -1.117888
C 2.300273 -0.674087 1.388452
C 3.666331 -0.933694 1.255460
C 4.379675 -0.573808 0.104667
C 3.653335 0.057554 -0.920768
C 2.291892 0.318475 -0.803004

O -5.108538 -2.948294 0.487652
C -6.349923 -2.361358 0.068476
O -6.082985 -0.999029 -0.311976
C 5.883729 -0.847152 -0.071291
C 6.499891 -1.541269 1.158102
C 6.626628 0.492110 -0.293079
C 6.099379 -1.761112 -1.301438
H 0.486454 4.673102 -0.429805
H 1.440963 2.506179 0.279246
H -3.349285 2.862769 -0.883131
H -2.331382 -2.938246 0.992365
H -4.525802 1.235167 -0.718840
H 1.782161 -0.969468 2.291985
H 4.167975 -1.434410 2.075645
H 4.150435 0.341071 -1.843883
H 1.760477 0.781393 -1.628268
H -6.740286 -2.907959 -0.794993
H -7.059442 -2.371760 0.901118
H 6.037640 -2.515934 1.351275
H 7.568660 -1.710604 0.986128
H 6.402933 -0.930368 2.062849
H 6.262618 1.018365 -1.181806
H 7.699955 0.311917 -0.428068
H 6.499867 1.158189 0.568275
H 7.168727 -1.958971 -1.443253
H 5.590296 -2.722698 -1.169021
H 5.720248 -1.303690 -2.221362

7 Energy:-1306.8855944
C -1.473086 3.828046 -0.839732
C -0.147630 3.808411 -0.413891
C 0.373077 2.616006 0.064838
C -0.397229 1.436424 0.101232
C -1.762378 1.493735 -0.269767
C -2.276653 2.711015 -0.761312
N 0.178161 0.246653 0.616750
C -0.608261 -0.894777 0.867929
C -2.041459 -0.816535 0.530677
C -2.607985 0.318214 -0.083440
C -2.806931 -1.981289 0.789917
C -4.125574 -1.961628 0.419790
C -4.695171 -0.840698 -0.199252
C -3.976231 0.299941 -0.460991
C 1.609046 0.045741 0.499925
O -0.115617 -1.930944 1.315162
F -1.994115 4.981997 -1.317304
C 2.312282 -0.699892 1.448483
C 3.668126 -0.983463 1.262414

C 4.364176 -0.572406 0.119709
C 3.627644 0.138751 -0.844193
C 2.278336 0.427452 -0.673657
O -5.070994 -2.941632 0.555964
C -6.284675 -2.416485 -0.003019
O -6.009456 -1.083128 -0.470594
C 5.855182 -0.871841 -0.112654
C 6.483681 -1.652736 1.056936
C 6.627097 0.459444 -0.275598
C 6.015729 -1.716226 -1.399656
H 0.451261 4.712043 -0.444263
H 1.394555 2.597168 0.422213
H -3.311884 2.801519 -1.065077
H -2.339779 -2.838829 1.256265
H -4.456317 1.142869 -0.940809
H 1.817370 -1.035665 2.348666
H 4.174356 -1.546641 2.038253
H 4.105836 0.462989 -1.763976
H 1.744065 0.946210 -1.461989
H -6.598113 -3.037997 -0.846984
H -7.056753 -2.375897 0.770952
H 6.000870 -2.625413 1.204468
H 7.543077 -1.837975 0.847318
H 6.424999 -1.094673 1.998377
H 6.253791 1.046685 -1.121269
H 7.691470 0.261357 -0.450666
H 6.540302 1.075893 0.626687
H 7.075690 -1.930799 -1.582058
H 5.485974 -2.671586 -1.309911
H 5.624660 -1.196322 -2.280598

8 Energy:-1306.88154428
C -1.460873 3.810455 -0.948778
C -0.167524 3.844553 -0.433329
C 0.345178 2.687187 0.131610
C -0.395727 1.488812 0.163181
C -1.733430 1.496329 -0.295509
C -2.242518 2.677870 -0.872796
N 0.179933 0.341219 0.774771
C -0.630444 -0.761770 1.119503
C -2.035543 -0.742827 0.667472
C -2.567032 0.315103 -0.095640
C -2.802413 -1.896041 0.970747
C -4.082811 -1.944236 0.485915
C -4.613516 -0.902426 -0.286686
C -3.893746 0.227439 -0.590895
C 1.606800 0.110382 0.627671
O -0.179297 -1.733639 1.725391

F -1.975552 4.929397 -1.508449
C 2.314085 -0.722158 1.502040
C 3.655484 -1.034327 1.259498
C 4.338998 -0.573976 0.129895
C 3.599699 0.219968 -0.764228
C 2.266482 0.540067 -0.538584
O -5.018925 -2.931970 0.630654
C -6.184658 -2.495619 -0.084715
O -5.892414 -1.204485 -0.650506
C 5.813024 -0.902940 -0.160625
C 6.442139 -1.781942 0.936726
C 6.623719 0.412052 -0.251924
C 5.916361 -1.660665 -1.506051
H 0.409103 4.762651 -0.459341
H 1.338920 2.708750 0.561184
H -3.260230 2.729941 -1.238620
H -2.364983 -2.695236 1.554632
H -4.340429 1.009087 -1.191756
H 1.839093 -1.099115 2.393854
H 4.159484 -1.662139 1.985817
H 4.061394 0.585833 -1.676950
H 1.736601 1.116609 -1.287177
H -6.405531 -3.201637 -0.890679
H -7.026603 -2.404473 0.607707
H 5.930360 -2.746500 1.030124
H 7.489673 -1.985918 0.688372
H 6.423708 -1.288310 1.915066
H 6.248662 1.067583 -1.045048
H 7.676650 0.194452 -0.468366
H 6.578688 0.966758 0.692500
H 6.964270 -1.894624 -1.729693
H 5.358149 -2.603240 -1.468569
H 5.522066 -1.069198 -2.339153

9 Energy:-1306.87726563
C -1.470361 3.805542 -1.046902
C -0.208810 3.902358 -0.464587
C 0.308452 2.783523 0.170786
C -0.394799 1.563999 0.205092
C -1.703367 1.509063 -0.324846
C -2.220196 2.650923 -0.969981
N 0.182238 0.456922 0.891235
C -0.664400 -0.566224 1.374235
C -2.023565 -0.645976 0.799909
C -2.510210 0.311406 -0.111315
C -2.785226 -1.789041 1.149338
C -4.009520 -1.933555 0.550354
C -4.489986 -0.996259 -0.374031

C -3.776138 0.125523 -0.721802
C 1.597224 0.171815 0.714808
O -0.274159 -1.409231 2.180744
F -1.990425 4.887099 -1.670715
C 2.281888 -0.762437 1.504960
C 3.605556 -1.110522 1.218142
C 4.303705 -0.584208 0.127949
C 3.589459 0.312414 -0.684218
C 2.273122 0.668911 -0.417370
O -4.922315 -2.942581 0.697921
C -6.043274 -2.600293 -0.131155
O -5.712532 -1.387951 -0.833554
C 5.760959 -0.945758 -0.203586
C 6.358883 -1.941929 0.807684
C 6.624371 0.338295 -0.187428
C 5.826443 -1.586482 -1.610709
H 0.336834 4.839143 -0.491709
H 1.275116 2.851737 0.655089
H -3.222200 2.657041 -1.380925
H -2.387006 -2.509876 1.851567
H -4.178558 0.826621 -1.441907
H 1.800443 -1.194807 2.366213
H 4.084676 -1.820610 1.883233
H 4.056408 0.736283 -1.568933
H 1.770091 1.322162 -1.118077
H -6.220541 -3.400190 -0.854983
H -6.923674 -2.426293 0.495602
H 5.808090 -2.889306 0.821247
H 7.395919 -2.166001 0.533758
H 6.365404 -1.535162 1.825353
H 6.271257 1.075128 -0.916743
H 7.666316 0.098687 -0.432128
H 6.607522 0.809530 0.802180
H 6.862771 -1.841616 -1.863429
H 5.230696 -2.505717 -1.650675
H 5.451941 -0.909014 -2.385420

10 Energy:-1306.87347219
C -1.502302 3.795683 -1.159349
C -0.266560 3.953750 -0.537500
C 0.264787 2.872803 0.151928
C -0.398406 1.632831 0.198653
C -1.687705 1.519093 -0.367676
C -2.219479 2.620570 -1.066526
N 0.187524 0.559122 0.931684
C -0.678375 -0.398098 1.510979
C -2.006503 -0.563077 0.883374
C -2.469177 0.309863 -0.120731

C -2.757477 -1.696127 1.282681
C -3.943978 -1.920710 0.633087
C -4.397401 -1.070424 -0.384284
C -3.694887 0.043676 -0.779318
C 1.590324 0.226924 0.739868
O -0.321449 -1.126033 2.435162
F -2.035409 4.838718 -1.835116
C 2.218941 -0.818428 1.435184
C 3.529396 -1.198628 1.133863
C 4.277984 -0.592374 0.120958
C 3.621831 0.417360 -0.599757
C 2.317079 0.807811 -0.318518
O -4.836270 -2.943451 0.808526
C -5.921692 -2.694676 -0.097560
O -5.582034 -1.532383 -0.876314
C 5.724670 -0.984649 -0.220913
C 6.253464 -2.109352 0.689083
C 6.644725 0.248012 -0.052304
C 5.793727 -1.475480 -1.686899
H 0.247265 4.908008 -0.574565
H 1.207581 2.990016 0.672850
H -3.209623 2.581291 -1.503949
H -2.380604 -2.350354 2.058347
H -4.075254 0.679333 -1.569120
H 1.703300 -1.315628 2.240295
H 3.958105 -2.000023 1.725809
H 4.125705 0.912482 -1.425327
H 1.866417 1.550956 -0.961129
H -6.045822 -3.552246 -0.763995
H -6.835654 -2.494514 0.470775
H 5.660436 -3.025825 0.591352
H 7.285519 -2.352007 0.412123
H 6.254263 -1.813233 1.744298
H 6.342581 1.073523 -0.705626
H 7.680378 -0.013489 -0.301485
H 6.625639 0.612768 0.981249
H 6.823124 -1.750401 -1.947207
H 5.158816 -2.356640 -1.835253
H 5.467004 -0.703258 -2.391439

11 Energy:-1306.87035239
C -1.547031 3.771360 -1.284387
C -0.331916 3.986869 -0.641485
C 0.218665 2.943151 0.091618
C -0.404337 1.684600 0.156814
C -1.682406 1.517348 -0.423806
C -2.233456 2.579088 -1.165559
N 0.194382 0.638339 0.919851

C -0.675996 -0.265978 1.575233
C -1.989132 -0.496473 0.937196
C -2.441590 0.302308 -0.131881
C -2.729747 -1.614692 1.391606
C -3.894780 -1.903755 0.727700
C -4.335727 -1.130250 -0.354184
C -3.643267 -0.029422 -0.802865
C 1.586182 0.269439 0.721930
O -0.324672 -0.902581 2.565510
F -2.097432 4.775834 -2.003074
C 2.141986 -0.881668 1.306050
C 3.445045 -1.285927 1.010090
C 4.264019 -0.597442 0.109313
C 3.682674 0.521244 -0.504008
C 2.384501 0.939173 -0.225033
O -4.772087 -2.931285 0.945420
C -5.840579 -2.758438 0.002250
O -5.497445 -1.646834 -0.846099
C 5.706633 -1.013549 -0.221334
C 6.143628 -2.268075 0.558411
C 6.670321 0.141804 0.140272
C 5.822469 -1.318989 -1.733922
H 0.150647 4.956625 -0.693249
H 1.137219 3.111504 0.640555
H -3.216236 2.498687 -1.614261
H -2.361842 -2.210724 2.217046
H -4.013733 0.547838 -1.640883
H 1.576398 -1.452304 2.025267
H 3.810833 -2.174744 1.512751
H 4.242234 1.090929 -1.240914
H 2.000508 1.774656 -0.791248
H -5.940171 -3.660335 -0.607131
H -6.768930 -2.532181 0.536593
H 5.516307 -3.134921 0.321193
H 7.175761 -2.524440 0.294322
H 6.109041 -2.106999 1.641934
H 6.433993 1.056596 -0.413739
H 7.704158 -0.136063 -0.098624
H 6.619480 0.374074 1.210368
H 6.849719 -1.609883 -1.985263
H 5.156688 -2.141615 -2.019612
H 5.563544 -0.448811 -2.346465

12 Energy:-1306.86805809
C -1.599182 3.732169 -1.421144
C -0.396569 3.998198 -0.776088
C 0.175601 2.991490 -0.006139
C -0.412399 1.719059 0.088989

C -1.688249 1.506466 -0.485603
C -2.260484 2.529186 -1.263089
N 0.202133 0.694821 0.869351
C -0.658240 -0.169657 1.589450
C -1.973249 -0.442836 0.973201
C -2.429841 0.293267 -0.138948
C -2.704498 -1.540833 1.486913
C -3.864386 -1.877512 0.835955
C -4.307301 -1.170016 -0.289270
C -3.624348 -0.087825 -0.795418
C 1.586026 0.302381 0.671115
O -0.287915 -0.744564 2.609242
F -2.169264 4.698464 -2.175771
C 2.061931 -0.941352 1.122769
C 3.362327 -1.363724 0.847508
C 4.260846 -0.598358 0.095300
C 3.761792 0.617870 -0.388538
C 2.466384 1.057680 -0.123789
O -4.733251 -2.899062 1.107846
C -5.792793 -2.797731 0.144126
O -5.461709 -1.724291 -0.756473
C 5.703864 -1.032976 -0.209143
C 6.040990 -2.403551 0.407842
C 6.687470 0.013206 0.367443
C 5.902972 -1.129610 -1.740648
H 0.059578 4.979196 -0.850467
H 1.071964 3.211028 0.559656
H -3.241498 2.413468 -1.708056
H -2.333236 -2.087770 2.344332
H -3.997338 0.438858 -1.665128
H 1.433437 -1.584135 1.719718
H 3.660874 -2.329841 1.239824
H 4.387978 1.256772 -1.005269
H 2.157090 1.981878 -0.586169
H -5.865735 -3.731950 -0.418865
H -6.732289 -2.564264 0.654994
H 5.395606 -3.197511 0.014911
H 7.076563 -2.670152 0.168541
H 5.945267 -2.393717 1.499583
H 6.521632 1.006825 -0.062471
H 7.722653 -0.276914 0.149787
H 6.578997 0.095215 1.455159
H 6.931952 -1.431026 -1.971727
H 5.225128 -1.871927 -2.177794
H 5.717241 -0.171302 -2.237282

13 Energy:-1306.86685238
C -1.669364 3.672687 -1.577265

C -0.460557 3.974691 -0.963236
C 0.138910 3.003108 -0.167574
C -0.427324 1.726674 -0.017691
C -1.716856 1.484061 -0.555267
C -2.315378 2.470540 -1.357783
N 0.208917 0.714838 0.761200
C -0.623006 -0.123586 1.542248
C -1.959292 -0.412168 0.983093
C -2.445605 0.282448 -0.143266
C -2.676529 -1.487647 1.559773
C -3.854749 -1.847030 0.955550
C -4.327403 -1.183393 -0.184056
C -3.658009 -0.123018 -0.750877
C 1.593893 0.323605 0.575746
O -0.209098 -0.668995 2.561073
F -2.264560 4.603159 -2.357225
C 1.990949 -0.995539 0.859583
C 3.294116 -1.429053 0.626205
C 4.277503 -0.592172 0.082975
C 3.863643 0.708720 -0.226942
C 2.565114 1.161964 0.006147
O -4.717306 -2.854771 1.291592
C -5.797101 -2.796794 0.347077
O -5.494991 -1.752692 -0.597330
C 5.725392 -1.039359 -0.178114
C 5.964098 -2.505494 0.229710
C 6.693226 -0.146615 0.634699
C 6.045626 -0.897526 -1.685338
H -0.018918 4.958597 -1.076729
H 1.025844 3.270439 0.388722
H -3.304486 2.329570 -1.776727
H -2.280281 -2.002473 2.425901
H -4.055513 0.369017 -1.629888
H 1.289856 -1.701863 1.279690
H 3.526652 -2.458996 0.873839
H 4.564056 1.412154 -0.668627
H 2.339029 2.169888 -0.298937
H -5.872546 -3.750937 -0.181448
H -6.728123 -2.554069 0.868709
H 5.327600 -3.195210 -0.336357
H 7.006471 -2.777884 0.029761
H 5.778595 -2.666927 1.297739
H 6.598548 0.909625 0.360999
H 7.732110 -0.447455 0.452223
H 6.497732 -0.234315 1.709693
H 7.079240 -1.205513 -1.884886
H 5.381028 -1.527175 -2.288232
H 5.933372 0.135971 -2.029885

14 Energy:-1306.88867426
C -1.580183 3.336777 -1.955197
C -0.247201 3.058120 -2.243714
C 0.302709 1.882175 -1.755865
C -0.450550 0.990216 -0.967045
C -1.821706 1.261485 -0.733684
C -2.363497 2.464255 -1.230344
N 0.142810 -0.206726 -0.499676
C -0.599172 -1.192181 0.173596
C -2.036262 -0.932560 0.368673
C -2.639419 0.278558 -0.027652
C -2.769733 -1.947680 1.032681
C -4.096979 -1.708700 1.272354
C -4.704931 -0.506654 0.885398
C -4.016665 0.493099 0.242882
C 1.583408 -0.297744 -0.397993
O -0.063883 -2.215021 0.603327
F -2.128070 4.484994 -2.417203
C 2.248368 -1.469504 -0.758022
C 3.627520 -1.583953 -0.567885
C 4.383519 -0.554309 0.007297
C 3.686785 0.604336 0.394513
C 2.312985 0.729776 0.214768
O -5.018227 -2.513165 1.886288
C -6.258835 -1.790655 1.877843
O -6.020755 -0.520364 1.244149
C 5.902961 -0.651338 0.230432
C 6.481861 -1.991768 -0.260409
C 6.210377 -0.516213 1.741162
C 6.608927 0.490835 -0.538864
H 0.337545 3.743990 -2.846932
H 1.331820 1.647865 -1.994228
H -3.402758 2.721657 -1.069483
H -2.273124 -2.862223 1.330015
H -4.527478 1.404699 -0.038446
H 1.697531 -2.287814 -1.204054
H 4.105242 -2.507049 -0.875723
H 4.218378 1.426965 0.863902
H 1.806953 1.628004 0.553696
H -7.003511 -2.348069 1.301810
H -6.592736 -1.624727 2.906189
H 6.320010 -2.137425 -1.334552
H 7.562929 -2.011086 -0.083121
H 6.044403 -2.844501 0.271037
H 5.862018 0.440299 2.144869
H 7.291585 -0.577515 1.914351
H 5.728559 -1.318163 2.312198

H 7.693219 0.438887 -0.382829
H 6.416817 0.415734 -1.615477
H 6.270960 1.477468 -0.204769

15 Energy:-1306.89087476
C -1.575967 3.264647 -2.055307
C -0.222150 3.013405 -2.258491
C 0.331514 1.872753 -1.695735
C -0.443810 0.988463 -0.920758
C -1.830937 1.235246 -0.762086
C -2.375185 2.401720 -1.335653
N 0.146052 -0.170649 -0.369395
C -0.593593 -1.137692 0.325253
C -2.041599 -0.901036 0.454882
C -2.655408 0.267867 -0.041540
C -2.778431 -1.893712 1.147883
C -4.120419 -1.676647 1.315685
C -4.740016 -0.518045 0.827791
C -4.048788 0.459279 0.154707
C 1.585561 -0.274930 -0.285148
O -0.046202 -2.125372 0.818445
F -2.127148 4.378220 -2.592775
C 2.240671 -1.410940 -0.755877
C 3.623858 -1.535303 -0.608696
C 4.390734 -0.546062 0.022317
C 3.703245 0.579256 0.511144
C 2.324350 0.712146 0.376479
O -5.048444 -2.467850 1.936693
C -6.302042 -1.774580 1.842621
O -6.070470 -0.547062 1.127460
C 5.916191 -0.652059 0.197235
C 6.484203 -1.955266 -0.396037
C 6.265112 -0.615141 1.704487
C 6.598047 0.540793 -0.514873
H 0.377550 3.694451 -2.852476
H 1.379240 1.661331 -1.863507
H -3.426536 2.640417 -1.235779
H -2.272901 -2.775303 1.520496
H -4.569339 1.337174 -0.205079
H 1.674833 -2.195449 -1.245066
H 4.097122 -2.430339 -0.995803
H 4.246950 1.368533 1.021863
H 1.821132 1.583615 0.784097
H -7.018262 -2.387029 1.287176
H -6.669408 -1.544077 2.847104
H 6.295539 -2.029927 -1.473058
H 7.569443 -1.982747 -0.247811
H 6.061769 -2.842781 0.088587

H 5.928055 0.312198 2.179423
H 7.350726 -0.685334 1.843058
H 5.799624 -1.453667 2.235055
H 7.686330 0.483113 -0.392345
H 6.376508 0.535290 -1.588441
H 6.267221 1.502169 -0.107936

16 Energy:-1306.89234323
C -1.567685 3.180948 -2.162931
C -0.199963 2.957249 -2.291314
C 0.354538 1.856269 -1.654509
C -0.437551 0.983926 -0.883752
C -1.835115 1.206146 -0.790577
C -2.379211 2.331988 -1.439918
N 0.148349 -0.132140 -0.250999
C -0.589173 -1.072332 0.475688
C -2.044948 -0.860616 0.545007
C -2.665181 0.258783 -0.049331
C -2.785551 -1.827213 1.269657
C -4.138222 -1.635908 1.369733
C -4.764759 -0.527631 0.783631
C -4.070031 0.423827 0.077919
C 1.587129 -0.251726 -0.184082
O -0.032440 -2.018130 1.036608
F -2.119446 4.255593 -2.774463
C 2.230206 -1.344270 -0.760505
C 3.616132 -1.477471 -0.653251
C 4.395082 -0.536301 0.034995
C 3.719072 0.547998 0.622652
C 2.337094 0.688339 0.528357
O -5.071936 -2.411047 2.002305
C -6.337437 -1.760485 1.811125
O -6.105707 -0.573761 1.030452
C 5.924320 -0.651749 0.167498
C 6.480145 -1.903290 -0.537939
C 6.306149 -0.731563 1.665054
C 6.589488 0.594658 -0.464208
H 0.410880 3.630220 -2.883165
H 1.414754 1.667449 -1.758954
H -3.438461 2.551729 -1.393855
H -2.274741 -2.670687 1.716179
H -4.596470 1.262558 -0.358612
H 1.650906 -2.089070 -1.295973
H 4.082558 -2.338380 -1.118175
H 4.275084 1.298387 1.176811
H 1.839635 1.527880 1.004984
H -7.012520 -2.426259 1.265516
H -6.754725 -1.478648 2.782455

H 6.267676 -1.894503 -1.613087
H 7.568408 -1.939248 -0.416369
H 6.069496 -2.826887 -0.114500
H 5.977568 0.154473 2.218333
H 7.394700 -0.808138 1.773979
H 5.854345 -1.610835 2.138410
H 7.680206 0.529698 -0.370625
H 6.344176 0.672517 -1.529753
H 6.267704 1.520737 0.023764

17 Energy:-1306.89327627
C -1.558301 3.087373 -2.276657
C -0.182015 2.890685 -2.343143
C 0.371548 1.830637 -1.639180
C -0.432789 0.971978 -0.866464
C -1.836398 1.171013 -0.826210
C -2.378954 2.254701 -1.544566
N 0.149327 -0.100316 -0.162242
C -0.587272 -1.014416 0.593352
C -2.047288 -0.822686 0.618821
C -2.670102 0.247260 -0.059171
C -2.790916 -1.759748 1.377956
C -4.149328 -1.590162 1.428021
C -4.778670 -0.532204 0.757955
C -4.081173 0.389808 0.016805
C 1.587505 -0.232688 -0.108082
O -0.025354 -1.923403 1.208168
F -2.108940 4.121397 -2.955835
C 2.223311 -1.265917 -0.791009
C 3.610786 -1.404913 -0.713657
C 4.396360 -0.524725 0.044551
C 3.727311 0.504522 0.731190
C 2.343839 0.649701 0.666765
O -5.086880 -2.344748 2.079522
C -6.358146 -1.733217 1.812527
O -6.125476 -0.591312 0.968012
C 5.927254 -0.648348 0.148609
C 6.476392 -1.832387 -0.669586
C 6.326047 -0.857926 1.629147
C 6.583881 0.649706 -0.379283
H 0.436547 3.554101 -2.937780
H 1.439490 1.663960 -1.690205
H -3.442934 2.455873 -1.543139
H -2.277938 -2.565597 1.887188
H -4.610069 1.189629 -0.484973
H 1.636036 -1.961055 -1.382814
H 4.073207 -2.219814 -1.258499
H 4.290753 1.207686 1.337386

H 1.849597 1.446534 1.214955
H -7.003765 -2.444580 1.289235
H -6.809763 -1.402001 2.752413
H 6.251323 -1.730635 -1.737372
H 7.566073 -1.875808 -0.564271
H 6.072474 -2.790496 -0.323380
H 6.003665 -0.023885 2.261265
H 7.415761 -0.942503 1.718517
H 5.879734 -1.775601 2.029094
H 7.675609 0.578991 -0.303735
H 6.326613 0.819745 -1.431202
H 6.266553 1.528878 0.191266

18 Energy:-1306.89380241
C -1.547844 2.987612 -2.388413
C -0.166594 2.816716 -2.403538
C 0.384799 1.799869 -1.636664
C -0.428359 0.957830 -0.855471
C -1.835508 1.134723 -0.859789
C -2.375471 2.174168 -1.642341
N 0.150295 -0.067322 -0.083461
C -0.586428 -0.944344 0.711965
C -2.049378 -0.774844 0.695814
C -2.672865 0.238102 -0.064686
C -2.796367 -1.679391 1.490030
C -4.158820 -1.536609 1.490700
C -4.788966 -0.536295 0.737945
C -4.088221 0.353813 -0.038315
C 1.587783 -0.214084 -0.041506
O -0.021838 -1.808524 1.386422
F -2.096270 3.979257 -3.129932
C 2.216255 -1.178416 -0.824478
C 3.604747 -1.322470 -0.773744
C 4.396934 -0.513558 0.053763
C 3.734894 0.450114 0.836039
C 2.351026 0.600609 0.796970
O -5.100100 -2.269791 2.161029
C -6.375484 -1.710688 1.811830
O -6.140058 -0.612063 0.912337
C 5.928797 -0.644919 0.131891
C 6.470163 -1.749360 -0.795496
C 6.341056 -0.987801 1.583584
C 6.580716 0.696099 -0.282224
H 0.457666 3.468166 -3.005408
H 1.457261 1.655374 -1.642476
H -3.442031 2.357977 -1.677658
H -2.282941 -2.442186 2.061387
H -4.617729 1.108871 -0.604703

H 1.622584 -1.816695 -1.472045
H 4.062402 -2.083457 -1.395209
H 4.304910 1.097912 1.495425
H 1.861084 1.347567 1.414801
H -6.981445 -2.468516 1.306345
H -6.871830 -1.339938 2.713456
H 6.235680 -1.550923 -1.847551
H 7.560713 -1.801683 -0.704252
H 6.069023 -2.735185 -0.534354
H 6.023737 -0.215282 2.291836
H 7.431583 -1.078516 1.655076
H 5.898925 -1.938678 1.902355
H 7.673089 0.619014 -0.224584
H 6.312905 0.961155 -1.311623
H 6.270045 1.519680 0.369187

19 Energy:-1306.8940003
C -1.549282 2.874518 -2.515742
C -0.164903 2.731819 -2.491387
C 0.387881 1.760586 -1.668387
C -0.427767 0.934418 -0.873234
C -1.837004 1.089408 -0.908583
C -2.378357 2.081325 -1.749614
N 0.151370 -0.043816 -0.043780
C -0.584771 -0.895783 0.777491
C -2.048776 -0.739870 0.741668
C -2.674010 0.224313 -0.078704
C -2.795256 -1.609978 1.573772
C -4.159039 -1.481612 1.552931
C -4.790834 -0.529316 0.741581
C -4.090659 0.325776 -0.073634
C 1.587968 -0.200131 -0.006430
O -0.017514 -1.729577 1.487279
F -2.099138 3.819867 -3.314449
C 2.220237 -1.075426 -0.885148
C 3.609299 -1.220122 -0.847112
C 4.397224 -0.500494 0.062697
C 3.731097 0.375719 0.938911
C 2.347341 0.528712 0.910354
O -5.100250 -2.188792 2.250660
C -6.376894 -1.656572 1.865458
O -6.142967 -0.610286 0.905370
C 5.929058 -0.637174 0.132025
C 6.475681 -1.635676 -0.905830
C 6.335929 -1.132630 1.540552
C 6.580715 0.740995 -0.134043
H 0.460649 3.370639 -3.105333
H 1.462961 1.639870 -1.640041

H -3.446897 2.244771 -1.813678
H -2.280488 -2.336502 2.189505
H -4.621476 1.044426 -0.684485
H 1.629388 -1.644547 -1.596885
H 4.070398 -1.910588 -1.543834
H 4.298436 0.952861 1.663011
H 1.854021 1.209608 1.597760
H -6.977322 -2.444997 1.402203
H -6.878411 -1.237077 2.742692
H 6.244374 -1.326642 -1.931601
H 7.566015 -1.695716 -0.816794
H 6.075602 -2.644338 -0.752587
H 6.013846 -0.440615 2.325706
H 7.426314 -1.228823 1.606908
H 5.894408 -2.112834 1.753961
H 7.672936 0.658847 -0.080978
H 6.316292 1.114201 -1.130246
H 6.267096 1.490340 0.600309

20 Energy:-1306.89382569
C -1.550483 2.763284 -2.629173
C -0.164278 2.655800 -2.564266
C 0.389720 1.734947 -1.686080
C -0.426120 0.921536 -0.877855
C -1.836969 1.049378 -0.945969
C -2.379722 1.988366 -1.844887
N 0.154242 -0.005024 0.008888
C -0.583971 -0.829800 0.856479
C -2.048922 -0.692163 0.796164
C -2.674781 0.216239 -0.084919
C -2.796118 -1.528525 1.661678
C -4.161073 -1.422547 1.612864
C -4.793450 -0.524673 0.742043
C -4.092636 0.296787 -0.106486
C 1.589287 -0.176897 0.037038
O -0.017952 -1.629470 1.605518
F -2.101802 3.657065 -3.484215
C 2.219402 -0.961429 -0.925507
C 3.608249 -1.112540 -0.903458
C 4.398519 -0.489142 0.072707
C 3.735017 0.299206 1.030736
C 2.352117 0.459904 1.017289
O -5.103043 -2.103269 2.335468
C -6.381350 -1.627043 1.887656
O -6.146610 -0.615301 0.891379
C 5.930028 -0.635569 0.126763
C 6.472454 -1.537295 -0.998223
C 6.338517 -1.257314 1.483719

C 6.584089 0.759854 -0.014550
H 0.461914 3.283667 -3.188769
H 1.466283 1.644965 -1.624102
H -3.449720 2.125198 -1.939341
H -2.280901 -2.213421 2.323046
H -4.623760 0.975401 -0.761296
H 1.627347 -1.456456 -1.689839
H 4.066837 -1.731652 -1.665794
H 4.304442 0.802601 1.806399
H 1.861555 1.074467 1.766149
H -6.943275 -2.452333 1.439818
H -6.923117 -1.186694 2.729621
H 6.242629 -1.134964 -1.991456
H 7.562534 -1.609808 -0.915509
H 6.067965 -2.554017 -0.937526
H 6.020157 -0.638410 2.329080
H 7.428776 -1.361747 1.538381
H 5.894861 -2.251710 1.608683
H 7.676245 0.671636 0.029757
H 6.319360 1.221906 -0.972695
H 6.272026 1.440092 0.784856

21 Energy:-1306.89326886
C -1.563428 2.641513 -2.751209
C -0.176730 2.576774 -2.651439
C 0.382177 1.711344 -1.721703
C -0.427816 0.907313 -0.897815
C -1.839892 1.002800 -0.992791
C -2.387929 1.883540 -1.946039
N 0.158314 0.035747 0.041286
C -0.580693 -0.761303 0.916632
C -2.046246 -0.643487 0.838900
C -2.675612 0.203873 -0.098143
C -2.790410 -1.442296 1.741877
C -4.156081 -1.359674 1.673914
C -4.792070 -0.521371 0.748052
C -4.094172 0.262064 -0.138019
C 1.591512 -0.151090 0.062714
O -0.014947 -1.525269 1.702039
F -2.119793 3.477954 -3.659215
C 2.226158 -0.835284 -0.971360
C 3.614408 -0.993005 -0.957198
C 4.400949 -0.476642 0.082130
C 3.733548 0.211882 1.111812
C 2.351891 0.380641 1.105894
O -5.095698 -2.012118 2.425075
C -6.375883 -1.583025 1.936935
O -6.145225 -0.623031 0.889718

C 5.931476 -0.634142 0.130006
C 6.478247 -1.412766 -1.081501
C 6.328433 -1.399231 1.415315
C 6.591715 0.765450 0.142691
H 0.446269 3.195420 -3.288208
H 1.459036 1.658868 -1.631505
H -3.458913 1.988839 -2.065971
H -2.272439 -2.081929 2.445049
H -4.628249 0.896447 -0.833420
H 1.638599 -1.248436 -1.786115
H 4.074912 -1.532405 -1.776856
H 4.299614 0.631354 1.938214
H 1.859951 0.919525 1.909622
H -6.918437 -2.440965 1.528169
H -6.935420 -1.106899 2.747136
H 6.255439 -0.906004 -2.027421
H 7.567599 -1.497608 -1.000859
H 6.070450 -2.428728 -1.131985
H 6.006064 -0.873170 2.319986
H 7.417938 -1.513447 1.465763
H 5.880526 -2.399456 1.430105
H 7.683198 0.668164 0.184395
H 6.335336 1.328242 -0.762392
H 6.276853 1.358111 1.008000

22 Energy:-1306.89226914
C -1.577802 2.531329 -2.854642
C -0.192811 2.520932 -2.718015
C 0.371215 1.715827 -1.738867
C -0.429351 0.913403 -0.903452
C -1.841272 0.964438 -1.029625
C -2.395172 1.783200 -2.033724
N 0.164751 0.107102 0.090300
C -0.576161 -0.654038 0.999426
C -2.042228 -0.573493 0.892463
C -2.674492 0.193379 -0.109018
C -2.783385 -1.336497 1.828594
C -4.148915 -1.299600 1.727204
C -4.787712 -0.541577 0.736337
C -4.092755 0.205430 -0.182888
C 1.594584 -0.105575 0.098143
O -0.012373 -1.365067 1.834171
F -2.139474 3.307116 -3.811636
C 2.225731 -0.701308 -0.992233
C 3.611877 -0.876542 -0.990523
C 4.402910 -0.464064 0.090633
C 3.740851 0.139855 1.175280
C 2.361804 0.326430 1.182441

O -5.086071 -1.931755 2.498486
C -6.366571 -1.575705 1.955542
O -6.140119 -0.675601 0.855510
C 5.930995 -0.644932 0.126678
C 6.471511 -1.316430 -1.149958
C 6.313994 -1.529337 1.337455
C 6.609394 0.738737 0.268762
H 0.424776 3.136732 -3.362750
H 1.446175 1.711641 -1.618101
H -3.466104 1.850559 -2.178389
H -2.263400 -1.916091 2.580557
H -4.629117 0.777103 -0.928976
H 1.637670 -1.036407 -1.841533
H 4.066710 -1.346891 -1.854716
H 4.309706 0.479908 2.035625
H 1.876863 0.800759 2.029096
H -6.871988 -2.474582 1.589288
H -6.960395 -1.068655 2.721292
H 6.257442 -0.722562 -2.045901
H 7.559518 -1.421964 -1.075705
H 6.051407 -2.318369 -1.293771
H 5.994291 -1.084131 2.285421
H 7.401846 -1.661296 1.380322
H 5.853903 -2.521084 1.259485
H 7.699446 0.623658 0.302221
H 6.362205 1.385844 -0.580818
H 6.300618 1.253256 1.184771

23 Energy:-1306.89063978
C -1.592487 2.430779 -2.944466
C -0.212681 2.485397 -2.770257
C 0.355659 1.742664 -1.745858
C -0.431585 0.933034 -0.903874
C -1.840912 0.930417 -1.061927
C -2.400308 1.685076 -2.112584
N 0.172117 0.196176 0.140198
C -0.572128 -0.533161 1.077290
C -2.037534 -0.496786 0.941480
C -2.670754 0.182076 -0.120261
C -2.775434 -1.225783 1.907168
C -4.138415 -1.244030 1.772618
C -4.777829 -0.574701 0.720190
C -4.086001 0.137651 -0.228487
C 1.597132 -0.050287 0.133140
O -0.012059 -1.190149 1.957155
F -2.158860 3.143592 -3.946293
C 2.225160 -0.561364 -1.002259
C 3.607775 -0.760989 -1.012446

C 4.403686 -0.451919 0.098798
C 3.748027 0.075124 1.226223
C 2.372579 0.284629 1.247072
O -5.072279 -1.860193 2.560646
C -6.350125 -1.586567 1.966113
O -6.127116 -0.750953 0.815600
C 5.927980 -0.662730 0.122491
C 6.459159 -1.243977 -1.201509
C 6.291909 -1.644569 1.261951
C 6.631158 0.693126 0.371495
H 0.396665 3.104996 -3.419158
H 1.425308 1.794701 -1.593631
H -3.469436 1.707630 -2.282019
H -2.255050 -1.739784 2.705068
H -4.622597 0.640251 -1.022532
H 1.637642 -0.820366 -1.877711
H 4.055455 -1.167791 -1.911927
H 4.319810 0.338266 2.111325
H 1.897260 0.700913 2.127805
H -6.814367 -2.524792 1.647158
H -6.980733 -1.053586 2.683309
H 6.258914 -0.578825 -2.049282
H 7.544779 -1.375861 -1.134390
H 6.020188 -2.223500 -1.422673
H 5.977183 -1.268041 2.240842
H 7.377143 -1.798874 1.296303
H 5.814582 -2.619007 1.106984
H 7.718887 0.555995 0.397468
H 6.397962 1.408095 -0.426043
H 6.329095 1.141085 1.323963

24 Energy:-1306.88825423
C -1.601796 2.339283 -3.020850
C -0.232066 2.467409 -2.808280
C 0.337482 1.788834 -1.741483
C -0.433496 0.965692 -0.896779
C -1.836428 0.901169 -1.088283
C -2.398694 1.589907 -2.182303
N 0.179834 0.302486 0.194051
C -0.571104 -0.390618 1.158698
C -2.033802 -0.409072 0.990004
C -2.662932 0.170639 -0.130432
C -2.770080 -1.104305 1.981828
C -4.126474 -1.189921 1.811577
C -4.760908 -0.621471 0.698579
C -4.070630 0.056728 -0.276023
C 1.597863 0.012156 0.170882
O -0.019157 -0.983359 2.087411

F -2.170178 2.987539 -4.064134
C 2.222511 -0.422090 -0.999401
C 3.599817 -0.652870 -1.022797
C 4.401753 -0.442425 0.106551
C 3.754644 0.019494 1.266367
C 2.384077 0.257066 1.302858
O -5.057455 -1.792109 2.613699
C -6.326096 -1.619462 1.964209
O -6.103603 -0.850769 0.767727
C 5.920766 -0.687861 0.115056
C 6.439430 -1.192537 -1.244809
C 6.262683 -1.749974 1.187333
C 6.653591 0.632914 0.451913
H 0.366672 3.095992 -3.458378
H 1.397328 1.902330 -1.556738
H -3.463334 1.562157 -2.377143
H -2.253557 -1.543801 2.825361
H -4.602603 0.480737 -1.117584
H 1.636158 -0.609837 -1.892921
H 4.038264 -1.004049 -1.949893
H 4.330111 0.211774 2.167290
H 1.921851 0.626508 2.209459
H -6.736032 -2.597648 1.694532
H -7.003853 -1.071692 2.624922
H 6.255598 -0.468211 -2.046552
H 7.521592 -1.354162 -1.187428
H 5.977396 -2.144505 -1.530114
H 5.955559 -1.431758 2.189111
H 7.344281 -1.929891 1.210663
H 5.764292 -2.701545 0.969262
H 7.738051 0.470471 0.468774
H 6.437179 1.403478 -0.297114
H 6.360019 1.024263 1.431584

25 Energy:-1306.88500122
C -1.608379 2.243448 -3.092862
C -0.253698 2.448470 -2.845505
C 0.314687 1.833547 -1.740680
C -0.436552 0.993595 -0.894044
C -1.829510 0.864695 -1.115675
C -2.392506 1.487536 -2.248632
N 0.186878 0.403533 0.237100
C -0.573655 -0.255764 1.224124
C -2.031977 -0.328172 1.027478
C -2.652877 0.151559 -0.142754
C -2.768168 -0.986955 2.044201
C -4.115134 -1.137644 1.846202
C -4.740089 -0.670784 0.681873

C -4.049677 -0.030061 -0.317870
C 1.596878 0.065891 0.200406
O -0.034178 -0.786723 2.195867
F -2.176497 2.827056 -4.173363
C 2.226164 -0.289801 -0.995459
C 3.598012 -0.548670 -1.029114
C 4.400390 -0.432496 0.112842
C 3.755600 -0.035075 1.296764
C 2.389868 0.227401 1.345648
O -5.043266 -1.725429 2.662124
C -6.301901 -1.635187 1.977470
O -6.073516 -0.951997 0.731091
C 5.913700 -0.709536 0.109410
C 6.428952 -1.124154 -1.281798
C 6.227147 -1.854168 1.102532
C 6.672597 0.566850 0.545189
H 0.331817 3.088365 -3.496561
H 1.360539 2.009703 -1.526375
H -3.449998 1.407313 -2.466350
H -2.258988 -1.351883 2.926746
H -4.573289 0.314980 -1.199796
H 1.646840 -0.411199 -1.903993
H 4.031786 -0.844180 -1.977661
H 4.329898 0.090753 2.210216
H 1.937793 0.552726 2.272228
H -6.681367 -2.641012 1.774054
H -7.007062 -1.057700 2.582166
H 6.262910 -0.339872 -2.029199
H 7.507443 -1.311193 -1.232906
H 5.949519 -2.043357 -1.637219
H 5.919334 -1.603493 2.123097
H 7.304726 -2.057751 1.118515
H 5.711049 -2.776747 0.812729
H 7.753308 0.380397 0.553845
H 6.476326 1.394838 -0.145875
H 6.382648 0.891436 1.550043

26 Energy:-1306.8809055
C -1.615914 2.178103 -3.152021
C -0.284419 2.475566 -2.872818
C 0.283928 1.933141 -1.730868
C -0.438888 1.072282 -0.880636
C -1.812912 0.853624 -1.136002
C -2.378965 1.404598 -2.304341
N 0.195362 0.570446 0.291118
C -0.581169 -0.009111 1.321039
C -2.023853 -0.194338 1.075642
C -2.623303 0.138224 -0.154591

C -2.758205 -0.822037 2.113293
C -4.078497 -1.095672 1.870808
C -4.678277 -0.781715 0.643727
C -3.989779 -0.171577 -0.376671
C 1.592034 0.171250 0.242266
O -0.066967 -0.401639 2.368389
F -2.185572 2.692784 -4.265767
C 2.218234 -0.129410 -0.973059
C 3.577745 -0.442040 -1.022690
C 4.384593 -0.426511 0.121485
C 3.752235 -0.071568 1.323975
C 2.397659 0.242901 1.391215
O -4.996569 -1.692437 2.691636
C -6.229553 -1.737689 1.957675
O -5.984449 -1.172282 0.656541
C 5.885717 -0.761450 0.099911
C 6.383664 -1.120826 -1.312802
C 6.155208 -1.968609 1.029792
C 6.693572 0.459544 0.601373
H 0.279454 3.132477 -3.525975
H 1.309092 2.181878 -1.487818
H -3.425365 1.260866 -2.542515
H -2.267530 -1.073736 3.044460
H -4.490260 0.052161 -1.309797
H 1.642972 -0.182316 -1.889560
H 3.998414 -0.697968 -1.988621
H 4.328920 -0.015587 2.243036
H 1.965754 0.539460 2.334496
H -6.550651 -2.777056 1.845185
H -6.986263 -1.139857 2.474729
H 6.248773 -0.291467 -2.016527
H 7.453861 -1.353142 -1.276979
H 5.867526 -1.999633 -1.715886
H 5.858023 -1.760815 2.063081
H 7.224061 -2.214569 1.032161
H 5.603485 -2.853773 0.692721
H 7.766323 0.231357 0.598710
H 6.529854 1.329919 -0.044615
H 6.415422 0.741336 1.622362

27 Energy:-1306.87643617
C -1.644872 2.124388 -3.228136
C -0.344262 2.527802 -2.935725
C 0.234779 2.072039 -1.761131
C -0.444400 1.194513 -0.893805
C -1.790574 0.865295 -1.167947
C -2.370841 1.330772 -2.365577
N 0.203680 0.788675 0.310882

C -0.593248 0.337200 1.392937
C -2.000040 -0.014411 1.110877
C -2.568442 0.139194 -0.168260
C -2.721811 -0.612422 2.174193
C -3.992286 -1.048633 1.902372
C -4.555351 -0.919181 0.625651
C -3.880509 -0.334845 -0.419522
C 1.583120 0.326263 0.263193
O -0.121126 0.167312 2.516002
F -2.225993 2.557532 -4.369907
C 2.218067 0.074168 -0.961654
C 3.557872 -0.312162 -1.016436
C 4.348406 -0.424098 0.133120
C 3.714480 -0.119663 1.347135
C 2.379294 0.269749 1.422218
O -4.884595 -1.664227 2.737655
C -6.065269 -1.912633 1.959661
O -5.811237 -1.449128 0.620356
C 5.828729 -0.839943 0.105464
C 6.326077 -1.132057 -1.322879
C 6.018818 -2.118992 0.955322
C 6.694897 0.298233 0.696260
H 0.184227 3.200073 -3.602658
H 1.233627 2.402579 -1.503649
H -3.402073 1.109687 -2.611377
H -2.259650 -0.721429 3.146661
H -4.347416 -0.254720 -1.392943
H 1.661918 0.106285 -1.889447
H 3.975873 -0.524788 -1.994128
H 4.276039 -0.161116 2.276519
H 1.954581 0.525799 2.378773
H -6.269869 -2.986692 1.937365
H -6.906606 -1.354533 2.381594
H 6.247558 -0.251375 -1.970573
H 7.381073 -1.426453 -1.291810
H 5.767327 -1.951354 -1.789588
H 5.718161 -1.963838 1.996839
H 7.072583 -2.423264 0.953275
H 5.424860 -2.948195 0.553787
H 7.753565 0.011548 0.690914
H 6.588800 1.217101 0.107985
H 6.416581 0.527031 1.730405

28 Energy:-1306.87246673
C -1.709360 2.051014 -3.326709
C -0.441410 2.554608 -3.049087
C 0.164383 2.185289 -1.856085
C -0.456912 1.296992 -0.959620

C -1.776123 0.865359 -1.221269
C -2.384607 1.243919 -2.433945
N 0.210712 0.969094 0.259707
C -0.596740 0.648693 1.382364
C -1.958445 0.141607 1.111951
C -2.506103 0.137393 -0.185724
C -2.651241 -0.426478 2.209466
C -3.868833 -1.001612 1.950593
C -4.407224 -1.031610 0.657253
C -3.761449 -0.474378 -0.421633
C 1.577676 0.470962 0.229081
O -0.161614 0.704424 2.530186
F -2.315770 2.400359 -4.483682
C 2.260535 0.303107 -0.985746
C 3.581478 -0.146920 -1.021080
C 4.308583 -0.422909 0.141433
C 3.625390 -0.221098 1.349803
C 2.310061 0.232178 1.407874
O -4.720081 -1.631993 2.817117
C -5.864182 -2.028234 2.045705
O -5.606288 -1.679740 0.672694
C 5.767265 -0.909142 0.135906
C 6.323607 -1.065761 -1.291972
C 5.854493 -2.282680 0.843056
C 6.653323 0.110092 0.891402
H 0.040173 3.237588 -3.740195
H 1.134152 2.598231 -1.605445
H -3.399904 0.947718 -2.668059
H -2.208651 -0.409783 3.197115
H -4.206665 -0.515921 -1.407802
H 1.758317 0.450840 -1.931536
H 4.034808 -0.278670 -1.997434
H 4.131686 -0.400491 2.294520
H 1.853980 0.403620 2.369131
H -6.001435 -3.109930 2.123886
H -6.748599 -1.486684 2.396049
H 6.317253 -0.115782 -1.838609
H 7.361497 -1.414219 -1.246430
H 5.753361 -1.799167 -1.873583
H 5.508183 -2.228817 1.880631
H 6.891806 -2.638899 0.855058
H 5.244372 -3.030527 0.323302
H 7.696489 -0.228652 0.903583
H 6.620747 1.092682 0.406520
H 6.331829 0.237415 1.930530

29 Energy:-1306.86940084
C -1.779364 1.945200 -3.421209

C -0.537859 2.521497 -3.172269
C 0.096492 2.224195 -1.972140
C -0.470516 1.333834 -1.044349
C -1.772948 0.835047 -1.275162
C -2.409943 1.140897 -2.492073
N 0.215937 1.056958 0.177415
C -0.586411 0.835036 1.327181
C -1.924407 0.250236 1.099122
C -2.465100 0.129750 -0.196635
C -2.594162 -0.270466 2.232950
C -3.780169 -0.923575 2.012198
C -4.308038 -1.072706 0.722969
C -3.685637 -0.559820 -0.391941
C 1.579776 0.554255 0.168272
O -0.155487 1.026551 2.460897
F -2.411926 2.223390 -4.583328
C 2.340666 0.516779 -1.010339
C 3.650803 0.032179 -1.017095
C 4.287984 -0.420169 0.141539
C 3.521626 -0.366886 1.315794
C 2.216497 0.114778 1.345507
O -4.603078 -1.534367 2.919073
C -5.720560 -2.042736 2.174718
O -5.472036 -1.779538 0.781099
C 5.732834 -0.944707 0.169534
C 6.386923 -0.923435 -1.224904
C 5.741737 -2.403148 0.686414
C 6.582270 -0.063499 1.116436
H -0.098874 3.207079 -3.888685
H 1.036653 2.708629 -1.738699
H -3.414521 0.793952 -2.701985
H -2.158773 -0.162095 3.218190
H -4.122735 -0.691977 -1.373981
H 1.914511 0.799494 -1.961388
H 4.167632 0.017204 -1.970474
H 3.950019 -0.699129 2.257668
H 1.700645 0.155566 2.291658
H -5.802672 -3.121777 2.329268
H -6.633695 -1.523906 2.483249
H 6.439141 0.091545 -1.635257
H 7.411515 -1.305971 -1.156865
H 5.844845 -1.553898 -1.939038
H 5.322002 -2.480092 1.695033
H 6.768283 -2.787880 0.721785
H 5.156724 -3.055776 0.028004
H 7.614940 -0.431354 1.154267
H 6.604289 0.976081 0.769396
H 6.188691 -0.068138 2.138448

30 Energy:-1306.86743765
C -1.853587 1.780299 -3.515016
C -0.629619 2.404814 -3.306651
C 0.033173 2.174429 -2.105496
C -0.487182 1.298543 -1.138654
C -1.785490 0.764336 -1.324276
C -2.449255 1.001574 -2.540729
N 0.218822 1.050666 0.077152
C -0.558485 0.910787 1.254264
C -1.898306 0.311871 1.083240
C -2.451660 0.108315 -0.197412
C -2.550939 -0.141912 2.254585
C -3.733702 -0.817096 2.088079
C -4.272618 -1.051654 0.816405
C -3.666834 -0.603788 -0.335378
C 1.594223 0.587410 0.091246
O -0.098356 1.169886 2.362339
F -2.511095 1.990347 -4.677629
C 2.452250 0.732485 -1.007634
C 3.761184 0.241146 -0.980352
C 4.294620 -0.408779 0.134431
C 3.423326 -0.557716 1.225700
C 2.118581 -0.078806 1.217235
O -4.541078 -1.378058 3.039940
C -5.665604 -1.933449 2.341116
O -5.428930 -1.764343 0.930948
C 5.734033 -0.944248 0.200854
C 6.515111 -0.676148 -1.099397
C 5.705007 -2.472418 0.442312
C 6.484759 -0.258213 1.367185
H -0.225137 3.080115 -4.052537
H 0.943735 2.721387 -1.900492
H -3.448929 0.623422 -2.717883
H -2.105246 0.029716 3.226231
H -4.114031 -0.800094 -1.302018
H 2.118876 1.181204 -1.929584
H 4.361185 0.384803 -1.872289
H 3.762227 -1.065093 2.124889
H 1.516318 -0.221904 2.101490
H -5.747135 -2.999503 2.568183
H -6.575668 -1.393759 2.622198
H 6.594229 0.395866 -1.313406
H 7.532917 -1.071135 -1.005671
H 6.048995 -1.164130 -1.963065
H 5.193762 -2.727384 1.376674
H 6.726520 -2.867350 0.502244
H 5.188528 -2.988469 -0.375371

H 7.511746 -0.637620 1.433936
H 6.532175 0.826768 1.218293
H 5.997108 -0.444034 2.330035

31 Energy:-1306.88495076
C -1.735557 1.052196 -3.620579
C -0.421472 0.600575 -3.707397
C 0.169734 0.071728 -2.570478
C -0.515484 0.012415 -1.340188
C -1.876306 0.402184 -1.293138
C -2.462393 0.946070 -2.454608
N 0.126298 -0.560130 -0.210344
C -0.594506 -0.845220 0.967215
C -2.026489 -0.499007 0.991943
C -2.651029 0.175312 -0.075998
C -2.723119 -0.804416 2.188213
C -4.035695 -0.420528 2.265581
C -4.662754 0.258376 1.211927
C -4.009895 0.567735 0.043671
C 1.564835 -0.456435 -0.058021
O -0.038173 -1.328327 1.954307
F -2.324557 1.580457 -4.718254
C 2.271142 0.633538 -0.573645
C 3.663143 0.690235 -0.478180
C 4.407088 -0.345282 0.101195
C 3.681436 -1.450771 0.577212
C 2.294470 -1.522171 0.488165
O -4.921493 -0.590434 3.294732
C -6.163894 -0.016866 2.860628
O -5.955332 0.534573 1.547327
C 5.940661 -0.314704 0.220223
C 6.546265 0.978124 -0.358357
C 6.541016 -1.516153 -0.548596
C 6.341210 -0.411180 1.711762
H 0.113905 0.648924 -4.649310
H 1.181198 -0.306870 -2.636619
H -3.496329 1.266872 -2.464296
H -2.211119 -1.313929 2.994129
H -4.533096 1.094177 -0.743915
H 1.740921 1.469598 -1.015972
H 4.159722 1.569428 -0.872697
H 4.205942 -2.293395 1.018737
H 1.777496 -2.401542 0.846126
H -6.929220 -0.797292 2.807620
H -6.456915 0.783290 3.546076
H 6.180487 1.869420 0.164113
H 7.636143 0.954059 -0.248207
H 6.324344 1.091138 -1.425698

H 6.181886 -2.472989 -0.155293
H 7.634587 -1.510694 -0.465660
H 6.280872 -1.470343 -1.612493
H 7.433260 -0.399505 1.812492
H 5.938200 0.434177 2.281338
H 5.973091 -1.333333 2.173478

32 Energy:-1306.88821583
C -1.739663 0.942087 -3.647304
C -0.401093 0.563313 -3.696923
C 0.198743 0.102497 -2.534724
C -0.508582 0.035746 -1.317850
C -1.887614 0.362752 -1.301926
C -2.480535 0.836648 -2.489850
N 0.132131 -0.459039 -0.155641
C -0.574496 -0.702906 1.034477
C -2.018803 -0.415481 1.032918
C -2.664478 0.156839 -0.081942
C -2.711681 -0.680591 2.240711
C -4.043107 -0.361865 2.283270
C -4.692920 0.212826 1.182425
C -4.044222 0.481326 0.001985
C 1.573197 -0.397101 -0.032066
O 0.003278 -1.109834 2.043965
F -2.336279 1.402608 -4.771468
C 2.283476 0.721571 -0.470838
C 3.677667 0.750341 -0.392457
C 4.410539 -0.335338 0.104545
C 3.675416 -1.461031 0.516420
C 2.286657 -1.504766 0.440787
O -4.931059 -0.514353 3.313299
C -6.193613 -0.023201 2.837963
O -6.002899 0.438236 1.487876
C 5.946035 -0.336152 0.204574
C 6.565278 0.983476 -0.293364
C 6.516777 -1.491647 -0.652146
C 6.364139 -0.539651 1.680556
H 0.149267 0.617224 -4.629885
H 1.232017 -0.215773 -2.571600
H -3.527924 1.108177 -2.525625
H -2.182750 -1.112364 3.080495
H -4.586524 0.926376 -0.822039
H 1.753765 1.588984 -0.850895
H 4.185111 1.646536 -0.730734
H 4.193530 -2.336284 0.897553
H 1.754606 -2.394444 0.752957
H -6.927440 -0.834719 2.842381
H -6.519910 0.813015 3.463061

H 6.221328 1.842640 0.293707
H 7.655928 0.934801 -0.200586
H 6.331394 1.172481 -1.347311
H 6.146689 -2.467298 -0.320042
H 7.611197 -1.508938 -0.583645
H 6.244349 -1.369312 -1.706876
H 7.457450 -0.550299 1.766232
H 5.981509 0.270769 2.311615
H 5.988646 -1.485793 2.084231

33 Energy:-1306.89061252
C -1.744056 0.825242 -3.671612
C -0.386104 0.520534 -3.689458
C 0.221362 0.132601 -2.504346
C -0.503212 0.061470 -1.298466
C -1.896447 0.325692 -1.308510
C -2.495584 0.724450 -2.520227
N 0.137882 -0.350480 -0.108264
C -0.557147 -0.554365 1.091551
C -2.011013 -0.323574 1.069353
C -2.673581 0.144860 -0.084287
C -2.700759 -0.547756 2.286961
C -4.046948 -0.295327 2.301151
C -4.714623 0.173768 1.161576
C -4.069275 0.401354 -0.029147
C 1.580473 -0.329692 -0.009344
O 0.037501 -0.888601 2.118289
F -2.347511 1.213011 -4.819664
C 2.301428 0.809257 -0.367005
C 3.696986 0.807749 -0.304061
C 4.413500 -0.324326 0.106913
C 3.663708 -1.464328 0.448815
C 2.273777 -1.477911 0.384600
O -4.935753 -0.427329 3.333286
C -6.218910 -0.043129 2.816444
O -6.037508 0.349886 1.443732
C 5.949852 -0.358715 0.190626
C 6.588699 0.979171 -0.227562
C 6.491571 -1.466037 -0.744916
C 6.377446 -0.664494 1.646153
H 0.175458 0.577549 -4.615560
H 1.272192 -0.123729 -2.515006
H -3.553829 0.945446 -2.578420
H -2.158502 -0.900537 3.154785
H -4.626239 0.764433 -0.883069
H 1.777976 1.706902 -0.681488
H 4.218869 1.716012 -0.582773
H 4.170733 -2.370606 0.766787

H 1.724062 -2.374842 0.645594
H -6.902310 -0.896559 2.860970
H -6.604039 0.806279 3.387736
H 6.268173 1.804229 0.418649
H 7.679131 0.904229 -0.151529
H 6.346847 1.241148 -1.263953
H 6.108387 -2.454949 -0.472221
H 7.586124 -1.505735 -0.689860
H 6.210794 -1.271475 -1.786523
H 7.471105 -0.699409 1.719782
H 6.014998 0.109865 2.332002
H 5.988842 -1.628149 1.991779

34 Energy:-1306.89224062
C -1.743067 0.684394 -3.695630
C -0.370976 0.451452 -3.685937
C 0.240406 0.139850 -2.480073
C -0.498727 0.070261 -1.283579
C -1.901962 0.274701 -1.316023
C -2.504086 0.594408 -2.549139
N 0.141829 -0.258262 -0.070259
C -0.545485 -0.418930 1.136663
C -2.006230 -0.239391 1.097455
C -2.679767 0.125444 -0.087550
C -2.694548 -0.417496 2.323196
C -4.050700 -0.224914 2.314961
C -4.729854 0.139373 1.144296
C -4.086263 0.320320 -0.055312
C 1.584992 -0.269529 0.012581
O 0.060713 -0.682000 2.177568
F -2.350155 0.995257 -4.865240
C 2.315900 0.887495 -0.250536
C 3.711856 0.862260 -0.198794
C 4.414112 -0.310208 0.112465
C 3.651855 -1.465140 0.366720
C 2.261295 -1.454445 0.311808
O -4.941110 -0.334205 3.348356
C -6.233890 -0.025217 2.805659
O -6.062106 0.269675 1.407424
C 5.950568 -0.372365 0.179920
C 6.605835 0.987474 -0.127440
C 6.468622 -1.402525 -0.852269
C 6.384638 -0.807883 1.600049
H 0.199592 0.507136 -4.606618
H 1.304184 -0.055960 -2.466374
H -3.570208 0.766123 -2.627356
H -2.143777 -0.692475 3.213599
H -4.652628 0.602038 -0.933469

H 1.798617 1.812198 -0.488811
H 4.245769 1.782637 -0.405421
H 4.149117 -2.399898 0.608401
H 1.697898 -2.361061 0.505672
H -6.893073 -0.891554 2.914156
H -6.643392 0.853328 3.312922
H 6.301983 1.758638 0.589526
H 7.695510 0.890795 -0.066813
H 6.360998 1.340586 -1.135690
H 6.072554 -2.405671 -0.662569
H 7.562812 -1.462432 -0.810398
H 6.183091 -1.114703 -1.870735
H 7.478206 -0.863353 1.660831
H 6.038111 -0.090665 2.352951
H 5.985467 -1.792476 1.865120

35 Energy:-1306.89327076
C -1.744818 0.532083 -3.717209
C -0.364070 0.359311 -3.689308
C 0.250507 0.121933 -2.467980
C -0.497284 0.063224 -1.276856
C -1.906325 0.219091 -1.323445
C -2.511133 0.461736 -2.572469
N 0.144401 -0.185266 -0.047434
C -0.536327 -0.305195 1.165020
C -2.001151 -0.163402 1.116374
C -2.682385 0.109439 -0.089399
C -2.687209 -0.292440 2.349289
C -4.048838 -0.144140 2.328204
C -4.735773 0.128377 1.137307
C -4.094656 0.259626 -0.070069
C 1.587681 -0.220872 0.023503
O 0.078505 -0.508268 2.214471
F -2.354913 0.767836 -4.902904
C 2.326737 0.949714 -0.131175
C 3.722549 0.906960 -0.084873
C 4.413477 -0.296270 0.116060
C 3.641623 -1.463111 0.265733
C 2.250881 -1.434245 0.215659
O -4.938337 -0.220477 3.365370
C -6.239130 0.010509 2.803300
O -6.072363 0.231292 1.390914
C 5.949505 -0.378833 0.174543
C 6.617311 0.997412 -0.006952
C 6.456396 -1.314052 -0.949451
C 6.380982 -0.948029 1.547377
H 0.211115 0.406094 -4.607620
H 1.322306 -0.022355 -2.436667

H -3.582146 0.591663 -2.664189
H -2.130673 -0.498378 3.254692
H -4.666958 0.470887 -0.964058
H 1.814834 1.895449 -0.283851
H 4.265825 1.836899 -0.206797
H 4.131145 -2.419845 0.421670
H 1.677075 -2.348580 0.329822
H -6.867430 -0.871370 2.959879
H -6.681737 0.901223 3.258848
H 6.320950 1.701876 0.778498
H 7.706036 0.885461 0.043120
H 6.375482 1.444242 -0.978062
H 6.050314 -2.326436 -0.852656
H 7.549948 -1.388528 -0.915000
H 6.173144 -0.930345 -1.936447
H 7.474116 -1.017176 1.601052
H 6.040571 -0.301477 2.364318
H 5.974769 -1.950159 1.720112

36 Energy:-1306.89381672
C -1.751920 0.376934 -3.734899
C -0.365840 0.255913 -3.697181
C 0.252705 0.089805 -2.465960
C -0.498410 0.047246 -1.276623
C -1.911035 0.161587 -1.330509
C -2.519655 0.330635 -2.589609
N 0.146626 -0.124957 -0.037353
C -0.528081 -0.205881 1.179965
C -1.995630 -0.094474 1.127174
C -2.683732 0.093978 -0.091177
C -2.678266 -0.174216 2.365987
C -4.043306 -0.061940 2.338704
C -4.737012 0.126330 1.135530
C -4.099487 0.207543 -0.078123
C 1.589808 -0.179205 0.024588
O 0.093540 -0.355907 2.234434
F -2.365767 0.540766 -4.930853
C 2.335865 0.996058 -0.010307
C 3.731125 0.939181 0.035729
C 4.413108 -0.283147 0.118514
C 3.633614 -1.454025 0.150949
C 2.242888 -1.410387 0.101722
O -4.930471 -0.103049 3.379857
C -6.237036 0.059867 2.807425
O -6.075918 0.208733 1.385022
C 5.948463 -0.380999 0.173900
C 6.625640 1.001759 0.124737
C 6.454286 -1.209735 -1.031163

C 6.370507 -1.079272 1.488873
H 0.210484 0.289353 -4.615366
H 1.329426 -0.009715 -2.424418
H -3.593875 0.424177 -2.688474
H -2.116767 -0.317109 3.280468
H -4.676752 0.354088 -0.981840
H 1.829004 1.954802 -0.070953
H 4.281501 1.872484 0.008089
H 4.116349 -2.424732 0.214875
H 1.661668 -2.327242 0.126454
H -6.839880 -0.829966 3.012047
H -6.705319 0.960747 3.214665
H 6.331378 1.631046 0.972322
H 7.713432 0.878245 0.166962
H 6.389855 1.539785 -0.800546
H 6.043049 -2.224613 -1.030692
H 7.547319 -1.292821 -1.000625
H 6.176323 -0.733939 -1.978773
H 7.462859 -1.162140 1.538900
H 6.032352 -0.508893 2.361570
H 5.955510 -2.089614 1.566248

37 Energy:-1306.89399056
C -1.766259 0.213222 -3.749236
C -0.376588 0.145395 -3.708927
C 0.247840 0.052084 -2.472980
C -0.501733 0.026000 -1.282330
C -1.917043 0.097080 -1.337587
C -2.531655 0.190997 -2.601564
N 0.149321 -0.071679 -0.038608
C -0.519379 -0.110713 1.183610
C -1.989099 -0.030705 1.130832
C -2.684825 0.071630 -0.093596
C -2.666550 -0.059810 2.374656
C -4.034064 0.016557 2.346425
C -4.735377 0.118306 1.137229
C -4.103092 0.147730 -0.081512
C 1.592206 -0.138689 0.017063
O 0.108426 -0.205445 2.240870
F -2.385842 0.303600 -4.950071
C 2.343760 1.029632 0.109294
C 3.738147 0.960154 0.160506
C 4.413477 -0.268158 0.120591
C 3.628462 -1.431755 0.025489
C 2.238035 -1.375414 -0.025915
O -4.917614 0.007875 3.391489
C -6.227747 0.129882 2.817081
O -6.075557 0.176559 1.386706

C 5.947804 -0.380170 0.178203
C 6.632300 0.996273 0.277588
C 6.460293 -1.083186 -1.101575
C 6.354289 -1.212765 1.417763
H 0.197949 0.165168 -4.628627
H 1.327779 -0.001772 -2.428209
H -3.608343 0.246799 -2.702179
H -2.099321 -0.138959 3.293342
H -4.686078 0.227873 -0.989888
H 1.841457 1.991953 0.144121
H 4.293643 1.887914 0.234294
H 4.106210 -2.406429 -0.007524
H 1.651791 -2.286933 -0.096391
H -6.828810 -0.742647 3.088520
H -6.693961 1.057968 3.161804
H 6.334353 1.536337 1.183432
H 7.719075 0.862613 0.315064
H 6.406791 1.628358 -0.588908
H 6.043609 -2.090076 -1.209567
H 7.552521 -1.175841 -1.070197
H 6.193781 -0.510313 -1.997355
H 7.445693 -1.306946 1.468398
H 6.011009 -0.733226 2.341589
H 5.933438 -2.223242 1.387055

12.3.5 Potential scan of compound 5b excited state (B3LYP/6-31G* PCM CH_2Cl_2)

D(4,7,15,18)
7 -1306.88679547 89.9445
23 -1306.88558948 99.9445
32 -1306.88292221 109.9446
38 -1306.88085697 119.9445
44 -1306.87868586 129.9444
52 -1306.87355915 139.9444
62 -1306.84986482 149.9446
68 -1306.84608602 159.9446
73 -1306.84388801 169.9445
78 -1306.84238895 179.9446
83 -1306.84122216 -170.0555
87 -1306.84020717 -160.0556
91 -1306.83925325 -150.0556
96 -1306.83846514 -140.0557
100 -1306.83799436 -130.0557
105 -1306.83763537 -120.0556
147 -1306.88289445 -110.0555
157 -1306.88547947 -100.0555
164 -1306.88675061 -90.0555

175 -1306.88568317 -80.0555
183 -1306.88273569 -70.0555
189 -1306.88062702 -60.0554
195 -1306.87833346 -50.0555
204 -1306.87107662 -40.0555
215 -1306.84864298 -30.0554
220 -1306.84627099 -20.0554
225 -1306.84428315 -10.0555
230 -1306.84277725 -0.0554
235 -1306.8413697 9.9445
240 -1306.84013661 19.9444
245 -1306.83890187 29.9444
249 -1306.83766887 39.9443
253 -1306.83647347 49.9443
259 -1306.83558386 59.9444
265 -1306.83592743 69.9444
295 -1306.8857163 79.9444
303 -1306.88677465 89.9445

Progress of structural optimization and emission calculation

-1306.893999 -1306.753157 323.51 nm
-1306.889711 -1306.759729 350.54 nm
-1306.886844 -1306.760427 360.42 nm
-1306.886672 -1306.760473 361.04 nm
-1306.886715 -1306.760485 360.96 nm
-1306.886765 -1306.760489 360.82 nm
-1306.886805 -1306.760491 360.72 nm
-1306.886795 -1306.760492 360.74 nm
-1306.886453 -1306.760321 361.24 nm
-1306.886548 -1306.760512 361.51 nm
-1306.886497 -1306.760589 361.88 nm
-1306.886105 -1306.760662 363.22 nm
-1306.885959 -1306.760714 363.79 nm
-1306.885533 -1306.760757 365.16 nm
-1306.885625 -1306.760761 364.9 nm
-1306.885607 -1306.760761 364.96 nm
-1306.885599 -1306.760761 364.98 nm
-1306.8856 -1306.760761 364.98 nm
-1306.885606 -1306.760761 364.96 nm
-1306.885612 -1306.760761 364.94 nm
-1306.885613 -1306.760762 364.94 nm
-1306.885606 -1306.760762 364.96 nm
-1306.885597 -1306.760763 364.99 nm
-1306.885589 -1306.760763 365.01 nm
-1306.885282 -1306.76104 366.73 nm
-1306.883568 -1306.761347 372.8 nm
-1306.883429 -1306.761384 373.33 nm

-1306.882949	-1306.7614	374.86	nm
-1306.88296	-1306.761404	374.83	nm
-1306.882903	-1306.761405	375.01	nm
-1306.882915	-1306.761406	374.98	nm
-1306.882921	-1306.761406	374.96	nm
-1306.882922	-1306.761406	374.96	nm
-1306.88241	-1306.761791	377.75	nm
-1306.880492	-1306.762006	384.55	nm
-1306.881254	-1306.762024	382.15	nm
-1306.880894	-1306.762031	383.33	nm
-1306.880865	-1306.762031	383.42	nm
-1306.880857	-1306.762031	383.45	nm
-1306.879772	-1306.761919	386.61	nm
-1306.878918	-1306.762159	390.23	nm
-1306.878677	-1306.76217	391.08	nm
-1306.878689	-1306.762172	391.04	nm
-1306.878689	-1306.762173	391.05	nm
-1306.878686	-1306.762173	391.06	nm
-1306.876899	-1306.761272	394.06	nm
-1306.875849	-1306.761606	398.83	nm
-1306.875091	-1306.761644	401.63	nm
-1306.874194	-1306.761668	404.91	nm
-1306.873897	-1306.761673	406	nm
-1306.873608	-1306.761677	407.07	nm
-1306.87356	-1306.761677	407.24	nm
-1306.873559	-1306.761677	407.24	nm
-1306.871642	-1306.760537	410.1	nm
-1306.864341	-1306.761936	444.93	nm
-1306.857266	-1306.762668	481.65	nm
-1306.854283	-1306.763257	500.55	nm
-1306.850841	-1306.763503	521.69	nm
-1306.850972	-1306.763588	521.42	nm
-1306.849865	-1306.763621	528.3	nm
-1306.849957	-1306.763628	527.79	nm
-1306.849829	-1306.763629	528.58	nm
-1306.849865	-1306.76363	528.36	nm
-1306.85055	-1306.765758	537.36	nm
-1306.844298	-1306.766145	583	nm
-1306.846608	-1306.766205	566.68	nm
-1306.846173	-1306.766211	569.82	nm
-1306.846097	-1306.766212	570.36	nm
-1306.846086	-1306.766212	570.44	nm
-1306.84679	-1306.767902	577.57	nm
-1306.844041	-1306.768279	601.39	nm
-1306.844044	-1306.76829	601.46	nm
-1306.843838	-1306.768292	603.12	nm
-1306.843888	-1306.768292	602.72	nm
-1306.844476	-1306.769228	605.51	nm

-1306.842096	-1306.769556	628.12	nm
-1306.842442	-1306.769562	625.19	nm
-1306.84238	-1306.769562	625.72	nm
-1306.842389	-1306.769563	625.64	nm
-1306.842655	-1306.769458	622.47	nm
-1306.840917	-1306.769746	640.19	nm
-1306.841235	-1306.769749	637.38	nm
-1306.841222	-1306.76975	637.5	nm
-1306.841222	-1306.76975	637.49	nm
-1306.841035	-1306.76839	627.21	nm
-1306.840037	-1306.768669	638.43	nm
-1306.840181	-1306.768672	637.17	nm
-1306.840207	-1306.768672	636.94	nm
-1306.8395	-1306.766004	619.95	nm
-1306.839083	-1306.766299	626.01	nm
-1306.839229	-1306.766301	624.78	nm
-1306.839253	-1306.766302	624.57	nm
-1306.838025	-1306.762369	602.25	nm
-1306.83841	-1306.762706	601.86	nm
-1306.83841	-1306.76271	601.89	nm
-1306.838476	-1306.76271	601.38	nm
-1306.838465	-1306.76271	601.46	nm
-1306.836798	-1306.757773	576.57	nm
-1306.837949	-1306.758203	571.35	nm
-1306.837985	-1306.758208	571.13	nm
-1306.837994	-1306.758208	571.07	nm
-1306.836106	-1306.752666	546.06	nm
-1306.837351	-1306.753171	541.26	nm
-1306.83752	-1306.753187	540.28	nm
-1306.837617	-1306.753189	539.67	nm
-1306.837635	-1306.753189	539.55	nm
-1306.835712	-1306.747444	516.19	nm
-1306.837314	-1306.748066	510.53	nm
-1306.83808	-1306.748108	506.41	nm
-1306.839027	-1306.748133	501.28	nm
-1306.839868	-1306.748148	496.76	nm
-1306.840835	-1306.748158	491.64	nm
-1306.841442	-1306.748161	488.46	nm
-1306.841812	-1306.748163	486.53	nm
-1306.841958	-1306.748163	485.77	nm
-1306.84267	-1306.748164	482.12	nm
-1306.847713	-1306.748069	457.26	nm
-1306.833597	-1306.741338	493.87	nm
-1306.828791	-1306.741202	520.2	nm
-1306.836716	-1306.745107	497.36	nm
-1306.836931	-1306.745082	496.07	nm
-1306.837698	-1306.746524	499.74	nm
-1306.842667	-1306.748164	482.14	nm

-1306.854382	-1306.74842	429.99	nm
-1306.857547	-1306.749191	420.5	nm
-1306.866487	-1306.750308	392.18	nm
-1306.87247	-1306.752669	380.33	nm
-1306.871245	-1306.752267	382.95	nm
-1306.879206	-1306.756433	371.12	nm
-1306.881673	-1306.757871	368.03	nm
-1306.882992	-1306.758327	365.49	nm
-1306.884398	-1306.758651	362.34	nm
-1306.884978	-1306.75916	362.14	nm
-1306.885364	-1306.759564	362.19	nm
-1306.885502	-1306.759895	362.74	nm
-1306.885364	-1306.760072	363.66	nm
-1306.884745	-1306.760432	366.52	nm
-1306.883665	-1306.760733	370.64	nm
-1306.883626	-1306.761179	372.11	nm
-1306.883739	-1306.761379	372.37	nm
-1306.882704	-1306.7614	375.61	nm
-1306.882819	-1306.761403	375.27	nm
-1306.883417	-1306.761406	373.44	nm
-1306.882692	-1306.761425	375.73	nm
-1306.882875	-1306.761429	375.17	nm
-1306.882877	-1306.76143	375.17	nm
-1306.882883	-1306.76143	375.15	nm
-1306.882894	-1306.76143	375.12	nm
-1306.88264	-1306.760424	372.81	nm
-1306.884272	-1306.760699	368.72	nm
-1306.885141	-1306.760752	366.3	nm
-1306.885457	-1306.760762	365.4	nm
-1306.885543	-1306.760765	365.15	nm
-1306.88557	-1306.760767	365.08	nm
-1306.88555	-1306.760768	365.14	nm
-1306.885479	-1306.760769	365.35	nm
-1306.885478	-1306.760769	365.36	nm
-1306.885479	-1306.760769	365.35	nm
-1306.885083	-1306.760012	364.3	nm
-1306.886409	-1306.760378	361.53	nm
-1306.886667	-1306.760438	360.96	nm
-1306.886753	-1306.760461	360.78	nm
-1306.886756	-1306.760467	360.79	nm
-1306.886749	-1306.76047	360.82	nm
-1306.886751	-1306.76047	360.81	nm
-1306.886445	-1306.760278	361.13	nm
-1306.886035	-1306.760631	363.33	nm
-1306.885905	-1306.760671	363.83	nm
-1306.885699	-1306.760679	364.45	nm
-1306.885706	-1306.76068	364.43	nm
-1306.885705	-1306.76068	364.44	nm

-1306.8857	-1306.76068	364.45	nm
-1306.885696	-1306.760681	364.46	nm
-1306.885672	-1306.76068	364.53	nm
-1306.885688	-1306.760681	364.49	nm
-1306.885683	-1306.760681	364.5	nm
-1306.885332	-1306.76085	366.02	nm
-1306.883212	-1306.761261	373.62	nm
-1306.882963	-1306.761318	374.56	nm
-1306.882705	-1306.76133	375.39	nm
-1306.882714	-1306.761331	375.37	nm
-1306.882758	-1306.761331	375.23	nm
-1306.882735	-1306.761331	375.31	nm
-1306.882736	-1306.761331	375.3	nm
-1306.882235	-1306.761669	377.91	nm
-1306.879931	-1306.761919	386.09	nm
-1306.880908	-1306.761942	383	nm
-1306.880652	-1306.761951	383.85	nm
-1306.880635	-1306.761951	383.91	nm
-1306.880627	-1306.761951	383.93	nm
-1306.87948	-1306.761788	387.14	nm
-1306.878387	-1306.762059	391.68	nm
-1306.878346	-1306.762068	391.85	nm
-1306.87837	-1306.762069	391.77	nm
-1306.878334	-1306.762069	391.89	nm
-1306.878333	-1306.762069	391.89	nm
-1306.876339	-1306.761122	395.46	nm
-1306.875116	-1306.761539	401.17	nm
-1306.87393	-1306.761606	405.64	nm
-1306.872977	-1306.761643	409.25	nm
-1306.872266	-1306.761655	411.93	nm
-1306.871489	-1306.761662	414.87	nm
-1306.871094	-1306.761663	416.37	nm
-1306.871054	-1306.761663	416.52	nm
-1306.871077	-1306.761664	416.43	nm
-1306.869146	-1306.760918	420.99	nm
-1306.860582	-1306.762748	465.72	nm
-1306.853861	-1306.763451	503.96	nm
-1306.851495	-1306.763603	518.4	nm
-1306.847605	-1306.763981	544.86	nm
-1306.849738	-1306.764171	532.49	nm
-1306.849064	-1306.764212	536.98	nm
-1306.848082	-1306.764218	543.3	nm
-1306.848867	-1306.764221	538.29	nm
-1306.848622	-1306.764222	539.85	nm
-1306.848643	-1306.764222	539.72	nm
-1306.849096	-1306.766016	548.43	nm
-1306.845248	-1306.766457	578.28	nm
-1306.846599	-1306.766485	568.74	nm

-1306.846289	-1306.76649	570.98	nm
-1306.846271	-1306.76649	571.1	nm
-1306.846723	-1306.767827	577.51	nm
-1306.844543	-1306.768236	597.11	nm
-1306.844444	-1306.768249	597.98	nm
-1306.844271	-1306.76825	599.35	nm
-1306.844283	-1306.768251	599.26	nm
-1306.844643	-1306.768996	602.32	nm
-1306.842605	-1306.769328	621.79	nm
-1306.842807	-1306.769332	620.12	nm
-1306.842741	-1306.769333	620.68	nm
-1306.842777	-1306.769333	620.38	nm
-1306.842926	-1306.769251	618.44	nm
-1306.841158	-1306.769523	636.05	nm
-1306.84139	-1306.769526	634.02	nm
-1306.841365	-1306.769526	634.25	nm
-1306.84137	-1306.769526	634.2	nm
-1306.841193	-1306.768446	626.33	nm
-1306.839951	-1306.768687	639.36	nm
-1306.840127	-1306.768689	637.8	nm
-1306.840136	-1306.76869	637.72	nm
-1306.840137	-1306.76869	637.72	nm
-1306.839512	-1306.766497	624.02	nm
-1306.838796	-1306.766742	632.34	nm
-1306.838862	-1306.766743	631.78	nm
-1306.838888	-1306.766744	631.56	nm
-1306.838902	-1306.766744	631.44	nm
-1306.837793	-1306.763377	612.27	nm
-1306.837643	-1306.763668	615.93	nm
-1306.837668	-1306.763671	615.74	nm
-1306.837669	-1306.763672	615.75	nm
-1306.836083	-1306.759188	592.54	nm
-1306.836394	-1306.759546	592.9	nm
-1306.836511	-1306.759553	592.05	nm
-1306.836473	-1306.759553	592.34	nm
-1306.834508	-1306.754174	567.18	nm
-1306.835312	-1306.754628	564.71	nm
-1306.835389	-1306.754641	564.26	nm
-1306.83553	-1306.754643	563.29	nm
-1306.835569	-1306.754643	563.03	nm
-1306.835584	-1306.754643	562.92	nm
-1306.833346	-1306.74862	537.77	nm
-1306.83486	-1306.749186	531.83	nm
-1306.835521	-1306.749217	527.94	nm
-1306.835809	-1306.749222	526.22	nm
-1306.835914	-1306.749222	525.58	nm
-1306.835927	-1306.749222	525.5	nm
-1306.833641	-1306.74315	503.51	nm

-1306.836984	-1306.744266	491.42	nm
-1306.841871	-1306.745306	471.84	nm
-1306.847289	-1306.743635	439.57	nm
-1306.854826	-1306.748108	426.95	nm
-1306.86407	-1306.749794	398.71	nm
-1306.86776	-1306.750538	388.69	nm
-1306.87108	-1306.751173	379.99	nm
-1306.87728	-1306.752263	364.46	nm
-1306.878376	-1306.753178	363.93	nm
-1306.879678	-1306.753876	362.18	nm
-1306.88218	-1306.755966	361	nm
-1306.884145	-1306.757843	360.75	nm
-1306.883619	-1306.758907	365.35	nm
-1306.881636	-1306.759815	374.02	nm
-1306.881631	-1306.760146	375.05	nm
-1306.881847	-1306.760328	374.95	nm
-1306.882615	-1306.760435	372.92	nm
-1306.883203	-1306.760479	371.27	nm
-1306.884243	-1306.760546	368.35	nm
-1306.88532	-1306.760632	365.42	nm
-1306.886105	-1306.76068	363.27	nm
-1306.885723	-1306.760695	364.43	nm
-1306.885649	-1306.760697	364.65	nm
-1306.885751	-1306.760703	364.37	nm
-1306.885736	-1306.760706	364.42	nm
-1306.885699	-1306.760707	364.53	nm
-1306.885707	-1306.760707	364.51	nm
-1306.885714	-1306.760707	364.49	nm
-1306.885716	-1306.760707	364.48	nm
-1306.885348	-1306.760048	363.63	nm
-1306.886626	-1306.760447	361.1	nm
-1306.886812	-1306.760496	360.71	nm
-1306.886802	-1306.760497	360.74	nm
-1306.886774	-1306.760502	360.84	nm
-1306.886777	-1306.760502	360.83	nm
-1306.886776	-1306.760502	360.83	nm
-1306.886775	-1306.760502	360.83	nm

Geometries of structural optimization

1 Energy:-1306.88679547
C -1.418417 3.866247 -0.005166
C -0.023001 3.712946 0.005844
C 0.490722 2.413341 0.013326
C -0.365072 1.294183 0.009803
C -1.820106 1.463272 0.000109
C -2.305512 2.811119 -0.008211
N 0.163541 0.013036 0.014192

C -0.636622 -1.171591 0.015397
C -2.070065 -1.003728 0.008215
C -2.663454 0.331216 -0.000387
C -2.869116 -2.163834 0.008353
C -4.235318 -1.997701 0.000250
C -4.832424 -0.707766 -0.008229
C -4.103966 0.440941 -0.008794
C 1.594866 -0.182002 0.019367
O -0.050832 -2.261268 0.022182
F -1.914093 5.122380 -0.013010
C 2.281904 -0.272521 1.226538
C 3.666083 -0.465116 1.224922
C 4.391517 -0.566236 0.029104
C 3.669394 -0.465318 -1.174168
C 2.289832 -0.273581 -1.187385
O -5.210066 -2.950849 -0.000998
C -6.466253 -2.256474 -0.012223
O -6.192382 -0.850812 -0.014884
C 5.915817 -0.778606 -0.007305
C 6.528456 -0.871049 1.403039
C 6.580678 0.407311 -0.746610
C 6.233309 -2.093537 -0.758968
H 0.622763 4.583108 0.008303
H 1.561746 2.262875 0.021692
H -3.365331 3.027031 -0.017023
H -2.404901 -3.142880 0.014682
H -4.601352 1.402056 -0.015196
H 1.738625 -0.200022 2.163968
H 4.172254 -0.537894 2.180483
H 4.187666 -0.539531 -2.125471
H 1.751187 -0.201721 -2.127519
H -7.017373 -2.524317 -0.918793
H -7.030177 -2.518364 0.888140
H 6.118152 -1.712914 1.972174
H 7.610518 -1.022633 1.322534
H 6.365441 0.046006 1.980581
H 6.217863 0.502423 -1.775447
H 7.667300 0.265616 -0.787580
H 6.380720 1.353249 -0.230083
H 7.317411 -2.254212 -0.796760
H 5.779414 -2.953544 -0.253345
H 5.863177 -2.075248 -1.789379

2 Energy:-1306.88558948
C -1.389443 3.842180 -0.235976
C -0.007091 3.692568 -0.038951
C 0.493234 2.396611 0.095995
C -0.361261 1.277143 0.030264

C -1.809721 1.446986 -0.110302
C -2.277924 2.787234 -0.275450
N 0.162590 -0.003742 0.075535
C -0.651760 -1.178918 0.216046
C -2.079353 -1.004476 0.144124
C -2.660388 0.318907 -0.060337
C -2.891070 -2.155414 0.244213
C -4.250992 -1.987986 0.140206
C -4.834490 -0.708807 -0.068894
C -4.094841 0.428633 -0.171139
C 1.589522 -0.205929 0.067604
O -0.070616 -2.259803 0.370534
F -1.872541 5.093030 -0.385549
C 2.275943 -0.463232 1.252783
C 3.661010 -0.644711 1.226907
C 4.390848 -0.569460 0.032090
C 3.670756 -0.300928 -1.147950
C 2.291581 -0.114929 -1.137273
O -5.234190 -2.932246 0.198275
C -6.482363 -2.236720 0.061595
O -6.193467 -0.848946 -0.144089
C 5.915979 -0.765957 -0.028688
C 6.525897 -1.050511 1.356902
C 6.576219 0.514698 -0.593791
C 6.242424 -1.961641 -0.955514
H 0.636983 4.562825 0.003656
H 1.554281 2.245347 0.242740
H -3.327618 3.002593 -0.424319
H -2.437138 -3.128215 0.391656
H -4.581669 1.382837 -0.326866
H 1.731403 -0.525759 2.189598
H 4.166096 -0.846220 2.164458
H 4.191304 -0.237719 -2.098798
H 1.754955 0.088158 -2.059267
H -7.021117 -2.630569 -0.804903
H -7.064850 -2.357995 0.980618
H 6.119530 -1.965803 1.801961
H 7.609095 -1.182934 1.259645
H 6.355651 -0.223745 2.055920
H 6.216243 0.749675 -1.600897
H 7.663734 0.385555 -0.649766
H 6.369360 1.378729 0.048259
H 7.327559 -2.108616 -1.013844
H 5.793778 -2.886418 -0.575031
H 5.872454 -1.802270 -1.973723

3 Energy:-1306.88292221
C -1.365010 3.811771 -0.425583

C -0.007779 3.687341 -0.089389
C 0.477866 2.405038 0.155712
C -0.362086 1.275147 0.052575
C -1.795996 1.428213 -0.196389
C -2.245485 2.746017 -0.482393
N 0.165744 0.000776 0.151114
C -0.666779 -1.163952 0.401998
C -2.082237 -0.992970 0.257878
C -2.649154 0.295923 -0.115996
C -2.909319 -2.132613 0.436369
C -4.255615 -1.980660 0.235596
C -4.820296 -0.735057 -0.149873
C -4.068539 0.387024 -0.327891
C 1.583331 -0.202565 0.127458
O -0.085849 -2.216193 0.691641
F -1.839705 5.044488 -0.689519
C 2.275947 -0.607184 1.272024
C 3.659072 -0.787416 1.218703
C 4.388309 -0.568790 0.040638
C 3.665722 -0.152393 -1.096621
C 2.290245 0.036915 -1.059580
O -5.247097 -2.918848 0.322992
C -6.484463 -2.220564 0.121122
O -6.172535 -0.890227 -0.310668
C 5.911232 -0.762569 -0.048391
C 6.524618 -1.212547 1.291004
C 6.575911 0.573340 -0.460593
C 6.228479 -1.841218 -1.112606
H 0.624188 4.564769 -0.023906
H 1.519553 2.265560 0.414274
H -3.279094 2.948342 -0.731329
H -2.468384 -3.084107 0.709362
H -4.540990 1.317182 -0.618501
H 1.735767 -0.777725 2.196460
H 4.166160 -1.099772 2.124295
H 4.184041 0.021835 -2.034614
H 1.753075 0.350136 -1.949782
H -7.063036 -2.728486 -0.653963
H -7.035933 -2.179908 1.067596
H 6.116128 -2.172974 1.625101
H 7.606908 -1.336273 1.174526
H 6.359888 -0.474499 2.084280
H 6.214092 0.928080 -1.431344
H 7.662513 0.446369 -0.535789
H 6.375794 1.355972 0.280304
H 7.312753 -1.983910 -1.192872
H 5.777768 -2.802953 -0.842207
H 5.854803 -1.560778 -2.102959

4 Energy:-1306.88085697
C -1.359619 3.794280 -0.567096
C -0.023380 3.704019 -0.153474
C 0.455961 2.439809 0.178394
C -0.365208 1.295552 0.077383
C -1.783437 1.420596 -0.243273
C -2.226840 2.713356 -0.614571
N 0.172429 0.032118 0.255346
C -0.673308 -1.125827 0.564987
C -2.077232 -0.968849 0.355375
C -2.633254 0.280673 -0.139745
C -2.912513 -2.100507 0.584890
C -4.244778 -1.974697 0.308259
C -4.793829 -0.768690 -0.202456
C -4.036368 0.343015 -0.428602
C 1.580053 -0.170665 0.208589
O -0.097240 -2.150022 0.948385
F -1.836082 5.004273 -0.914897
C 2.284368 -0.689078 1.304589
C 3.662516 -0.881012 1.216892
C 4.384073 -0.567892 0.054481
C 3.654038 -0.040585 -1.033108
C 2.284685 0.163461 -0.963106
O -5.238114 -2.913032 0.413284
C -6.465843 -2.231564 0.119848
O -6.135534 -0.948073 -0.426223
C 5.901644 -0.773632 -0.069015
C 6.523185 -1.347008 1.218463
C 6.581277 0.583966 -0.372582
C 6.190554 -1.760231 -1.227156
H 0.595271 4.590897 -0.090736
H 1.481014 2.323983 0.507411
H -3.249323 2.893738 -0.920869
H -2.481281 -3.025060 0.950225
H -4.497344 1.243811 -0.815380
H 1.755156 -0.927983 2.218624
H 4.175506 -1.275684 2.086252
H 4.163618 0.206005 -1.959573
H 1.743144 0.554727 -1.818868
H -7.030202 -2.805514 -0.618351
H -7.039300 -2.100279 1.045412
H 6.104546 -2.327289 1.472657
H 7.602101 -1.474724 1.077889
H 6.378342 -0.678732 2.074911
H 6.214050 1.026212 -1.304508
H 7.664820 0.448292 -0.472005
H 6.401379 1.301932 0.435821

H 7.271671 -1.910438 -1.332042
H 5.728365 -2.735311 -1.035683
H 5.810791 -1.389252 -2.184783

5 Energy:-1306.87868586
C -1.355147 3.779405 -0.686961
C -0.042430 3.729082 -0.205667
C 0.431170 2.487537 0.210826
C -0.367983 1.327767 0.119817
C -1.765678 1.417005 -0.272156
C -2.205594 2.681889 -0.722422
N 0.179890 0.081741 0.393836
C -0.680041 -1.069831 0.744469
C -2.068393 -0.936593 0.452957
C -2.608565 0.267456 -0.154179
C -2.910721 -2.060389 0.719634
C -4.224120 -1.969616 0.361801
C -4.752169 -0.810385 -0.264319
C -3.989969 0.292444 -0.524996
C 1.577054 -0.123828 0.315781
O -0.118984 -2.061101 1.223152
F -1.833446 4.962644 -1.114683
C 2.298488 -0.734809 1.357352
C 3.668310 -0.946367 1.224877
C 4.374599 -0.568610 0.070833
C 3.632055 0.045845 -0.962625
C 2.271361 0.271045 -0.849746
O -5.216136 -2.911753 0.472664
C -6.430447 -2.257283 0.081187
O -6.077050 -1.022481 -0.555785
C 5.883932 -0.791470 -0.097599
C 6.518461 -1.469734 1.131165
C 6.582633 0.573454 -0.314441
C 6.132176 -1.694073 -1.331787
H 0.561829 4.626276 -0.151309
H 1.437199 2.402347 0.603338
H -3.215545 2.834015 -1.081753
H -2.493191 -2.951982 1.172200
H -4.435059 1.158146 -1.000845
H 1.787748 -1.016455 2.268097
H 4.191283 -1.405366 2.055779
H 4.126347 0.340338 -1.883277
H 1.721417 0.719276 -1.670984
H -6.969591 -2.889021 -0.627771
H -7.037121 -2.050145 0.971519
H 6.084518 -2.457728 1.321776
H 7.591444 -1.607528 0.958789
H 6.404600 -0.863306 2.036838

H 6.205698 1.089553 -1.203517
H 7.661188 0.426165 -0.445995
H 6.432129 1.232437 0.548293
H 7.208242 -1.854665 -1.467842
H 5.655209 -2.672411 -1.204274
H 5.742925 -1.245849 -2.251781

6 Energy:-1306.87355915
C -1.334287 3.775159 -0.755526
C -0.060696 3.778349 -0.185507
C 0.402737 2.568991 0.328266
C -0.365572 1.390506 0.239594
C -1.720232 1.424293 -0.264412
C -2.157101 2.652764 -0.795080
N 0.186427 0.170004 0.624650
C -0.712521 -0.938446 1.084887
C -2.051805 -0.873411 0.615226
C -2.540695 0.245545 -0.168168
C -2.915215 -1.977692 0.921379
C -4.176129 -1.958849 0.405261
C -4.638710 -0.894189 -0.408347
C -3.862145 0.195080 -0.698266
C 1.562981 -0.063971 0.483582
O -0.212492 -1.817361 1.794147
F -1.811159 4.923111 -1.272009
C 2.321618 -0.750602 1.457010
C 3.674762 -0.988846 1.247737
C 4.342252 -0.569451 0.082162
C 3.569436 0.115577 -0.882817
C 2.224014 0.368279 -0.696409
O -5.162469 -2.911371 0.500087
C -6.329892 -2.328805 -0.093767
O -5.913690 -1.176051 -0.837601
C 5.833836 -0.822714 -0.166806
C 6.506235 -1.570497 0.999884
C 6.558701 0.532613 -0.359371
C 5.997999 -1.676212 -1.449787
H 0.522923 4.689458 -0.136164
H 1.378442 2.523825 0.798534
H -3.144801 2.763648 -1.225320
H -2.543715 -2.802789 1.517325
H -4.256975 0.992739 -1.317039
H 1.847278 -1.061176 2.376017
H 4.222644 -1.499762 2.030925
H 4.027544 0.439878 -1.812081
H 1.651250 0.859592 -1.475507
H -6.789642 -3.050350 -0.772059
H -7.028534 -2.023037 0.695522

H 6.055873 -2.555259 1.167386
H 7.565953 -1.726487 0.770841
H 6.450491 -1.002615 1.935473
H 6.154459 1.097118 -1.205951
H 7.624881 0.362384 -0.550127
H 6.468678 1.156356 0.537204
H 7.061844 -1.856831 -1.643882
H 5.501751 -2.647119 -1.341067
H 5.577989 -1.178286 -2.329764

7 Energy:-1306.84986482
C -1.478715 3.881113 -0.737518
C -0.235229 4.003516 -0.124152
C 0.322838 2.853813 0.435152
C -0.354619 1.631245 0.357411
C -1.652202 1.522389 -0.235679
C -2.193577 2.684686 -0.796569
N 0.208923 0.418126 0.777126
C -0.736984 -0.476583 1.581455
C -1.945255 -0.698765 0.861895
C -2.344682 0.230395 -0.172035
C -2.811927 -1.773025 1.240361
C -3.943405 -1.958681 0.496277
C -4.280267 -1.115475 -0.581884
C -3.520452 -0.020952 -0.920055
C 1.504654 0.049742 0.525905
O -0.427769 -0.786948 2.742054
F -2.036509 4.977175 -1.290226
C 2.221620 -0.794271 1.419642
C 3.547876 -1.108993 1.170239
C 4.239417 -0.621585 0.044054
C 3.515416 0.220510 -0.837150
C 2.199261 0.560127 -0.611737
O -4.876403 -2.964103 0.591104
C -5.925316 -2.610149 -0.320679
O -5.423069 -1.585781 -1.188309
C 5.701666 -0.964565 -0.254515
C 6.319596 -1.887891 0.812183
C 6.530461 0.343926 -0.307552
C 5.788068 -1.679767 -1.626914
H 0.267762 4.962775 -0.089265
H 1.290170 2.896919 0.925053
H -3.168820 2.689117 -1.269847
H -2.536214 -2.435278 2.053143
H -3.830680 0.634520 -1.727167
H 1.720612 -1.156634 2.306930
H 4.059508 -1.741537 1.886050
H 3.997773 0.603834 -1.730678

H 1.668690 1.183074 -1.322590
H -6.200500 -3.484938 -0.913307
H -6.785110 -2.224589 0.242654
H 5.792975 -2.846544 0.877169
H 7.361564 -2.100350 0.550218
H 6.316484 -1.424631 1.805199
H 6.167556 1.028767 -1.080823
H 7.577849 0.111133 -0.532332
H 6.496718 0.869046 0.653591
H 6.832960 -1.921315 -1.853898
H 5.216055 -2.614268 -1.618960
H 5.405756 -1.055244 -2.440759

8 Energy:-1306.84608602
C -1.537365 3.914363 -0.816228
C -0.331706 4.113264 -0.150571
C 0.254196 3.007065 0.468146
C -0.363508 1.757028 0.396351
C -1.623652 1.567438 -0.246231
C -2.191998 2.685509 -0.870821
N 0.228578 0.574173 0.883152
C -0.711076 -0.317032 1.692480
C -1.873649 -0.624557 0.926922
C -2.263856 0.248253 -0.158428
C -2.712639 -1.718048 1.309856
C -3.802287 -1.978494 0.526482
C -4.124326 -1.191551 -0.596953
C -3.394167 -0.080806 -0.946070
C 1.492388 0.166851 0.585979
O -0.441974 -0.547460 2.882642
F -2.117959 4.968026 -1.428466
C 2.162595 -0.777923 1.420777
C 3.466905 -1.151091 1.146072
C 4.183345 -0.630639 0.050338
C 3.507009 0.309077 -0.772255
C 2.214656 0.709590 -0.522124
O -4.697887 -3.017712 0.621370
C -5.720750 -2.739124 -0.344431
O -5.220913 -1.730471 -1.231567
C 5.622855 -1.034239 -0.276603
C 6.190495 -2.055734 0.726581
C 6.522702 0.227586 -0.251477
C 5.666370 -1.664388 -1.692304
H 0.125833 5.095371 -0.125072
H 1.197609 3.108242 0.995212
H -3.143917 2.628971 -1.386490
H -2.447137 -2.337300 2.159155
H -3.694587 0.530571 -1.790579

H 1.646328 -1.162623 2.289879
H 3.942057 -1.858591 1.814998
H 4.011944 0.721745 -1.639656
H 1.723332 1.409748 -1.186949
H -5.939413 -3.645840 -0.912049
H -6.616379 -2.365054 0.168707
H 5.612624 -2.986617 0.733162
H 7.218853 -2.307368 0.446544
H 6.214138 -1.656731 1.746808
H 6.198591 0.978285 -0.979414
H 7.554977 -0.049458 -0.494707
H 6.519189 0.692596 0.740632
H 6.695970 -1.948403 -1.938786
H 5.043403 -2.564383 -1.740692
H 5.318725 -0.969031 -2.463060

9 Energy:-1306.84388801
C -1.566312 3.935819 -0.901848
C -0.418432 4.215924 -0.166753
C 0.179385 3.159232 0.524761
C -0.372215 1.881475 0.454794
C -1.578106 1.607738 -0.252980
C -2.156286 2.676481 -0.954919
N 0.247191 0.739182 1.020335
C -0.693122 -0.164326 1.809383
C -1.798558 -0.547530 0.994800
C -2.167514 0.267973 -0.142454
C -2.611546 -1.661568 1.376354
C -3.647366 -1.996694 0.550394
C -3.942340 -1.266193 -0.618024
C -3.240844 -0.139916 -0.973300
C 1.481100 0.294903 0.679820
O -0.462405 -0.362239 3.013869
F -2.154330 4.940735 -1.587477
C 2.105814 -0.733725 1.454990
C 3.380811 -1.168057 1.142017
C 4.113446 -0.630625 0.064880
C 3.485402 0.394346 -0.695239
C 2.223252 0.856149 -0.408106
O -4.503339 -3.069693 0.639882
C -5.485146 -2.870500 -0.385942
O -4.981615 -1.875049 -1.285641
C 5.520711 -1.100140 -0.307004
C 6.039773 -2.206469 0.630265
C 6.494844 0.103329 -0.230498
C 5.504497 -1.654303 -1.755072
H -0.009813 5.219521 -0.147562
H 1.083486 3.321732 1.103224

H -3.070254 2.556317 -1.525482
H -2.364501 -2.237383 2.260994
H -3.520786 0.425451 -1.855972
H 1.580349 -1.131977 2.312264
H 3.820522 -1.939163 1.762857
H 4.007630 0.825332 -1.543038
H 1.770343 1.622672 -1.024385
H -5.637401 -3.806021 -0.928006
H -6.420714 -2.516235 0.066518
H 5.406601 -3.100043 0.597648
H 7.046536 -2.503626 0.318515
H 6.102951 -1.865260 1.669567
H 6.207673 0.910019 -0.912540
H 7.504842 -0.222077 -0.504829
H 6.532712 0.513867 0.784605
H 6.511929 -1.983000 -2.034818
H 4.829706 -2.513322 -1.838942
H 5.185965 -0.899978 -2.481642

10 Energy:-1306.84238895
C -1.581041 3.936711 -0.998687
C -0.493126 4.291643 -0.206288
C 0.109241 3.283719 0.551523
C -0.379296 1.981800 0.489141
C -1.531212 1.631826 -0.270360
C -2.111206 2.652495 -1.042954
N 0.263715 0.883049 1.127477
C -0.679396 -0.030451 1.900647
C -1.733529 -0.478162 1.053311
C -2.080273 0.280390 -0.130389
C -2.524270 -1.606649 1.442729
C -3.510914 -2.009007 0.588306
C -3.780348 -1.333104 -0.619229
C -3.102791 -0.197854 -0.988890
C 1.475631 0.408369 0.761609
O -0.472878 -0.209974 3.112225
F -2.170023 4.893574 -1.751082
C 2.042890 -0.703920 1.467169
C 3.288201 -1.191133 1.119104
C 4.048465 -0.628057 0.074215
C 3.479915 0.480481 -0.614793
C 2.247433 0.993948 -0.294247
O -4.333082 -3.108165 0.682928
C -5.278163 -2.979314 -0.387570
O -4.769175 -2.003616 -1.305132
C 5.425771 -1.151380 -0.333378
C 5.876733 -2.351839 0.519498
C 6.468033 -0.015175 -0.170715

C 5.379913 -1.596265 -1.818155
H -0.130318 5.312821 -0.196293
H 0.971590 3.503630 1.173274
H -2.986093 2.473942 -1.657812
H -2.295151 -2.139669 2.358432
H -3.362835 0.322731 -1.904488
H 1.500614 -1.125206 2.302337
H 3.682676 -2.026645 1.684143
H 4.027328 0.936482 -1.432933
H 1.840990 1.825320 -0.855596
H -5.380082 -3.939198 -0.897875
H -6.241567 -2.638084 0.013806
H 5.191402 -3.201333 0.423972
H 6.864267 -2.684738 0.183510
H 5.959402 -2.091659 1.580584
H 6.229624 0.855204 -0.790327
H 7.457334 -0.378378 -0.471333
H 6.528330 0.315880 0.871901
H 6.366772 -1.963491 -2.121839
H 4.655212 -2.404898 -1.963451
H 5.109812 -0.773565 -2.487760

11 Energy:-1306.84122216
C -1.583282 3.924184 -1.094919
C -0.551629 4.344585 -0.259688
C 0.049458 3.382338 0.556300
C -0.384151 2.061274 0.508149
C -1.486625 1.645385 -0.289442
C -2.062718 2.621243 -1.123615
N 0.276432 1.005528 1.210978
C -0.671205 0.086613 1.972774
C -1.681001 -0.416221 1.104858
C -2.004832 0.288304 -0.118760
C -2.452457 -1.555259 1.505871
C -3.394488 -2.016777 0.632421
C -3.639463 -1.391801 -0.608186
C -2.981586 -0.251580 -0.995056
C 1.474551 0.505871 0.836261
O -0.479869 -0.079178 3.188455
F -2.168175 4.835910 -1.905746
C 1.972803 -0.688685 1.457703
C 3.189473 -1.219784 1.077000
C 3.989999 -0.622263 0.082043
C 3.491514 0.567203 -0.522641
C 2.286852 1.122709 -0.171532
O -4.187384 -3.136822 0.736775
C -5.097341 -3.072421 -0.369053
O -4.582995 -2.116125 -1.303663

C 5.339556 -1.189237 -0.356599
C 5.717043 -2.472295 0.406969
C 6.440651 -0.126782 -0.105832
C 5.280875 -1.519206 -1.870742
H -0.228234 5.378949 -0.262639
H 0.871845 3.652886 1.211417
H -2.901114 2.391503 -1.771467
H -2.240860 -2.048686 2.447570
H -3.221732 0.227206 -1.938419
H 1.404580 -1.142056 2.257660
H 3.529111 -2.119460 1.574821
H 4.073187 1.053699 -1.298270
H 1.934451 2.016913 -0.668557
H -5.155677 -4.052229 -0.847325
H -6.083137 -2.744438 -0.013428
H 4.988018 -3.273974 0.245040
H 6.688101 -2.832624 0.051972
H 5.804177 -2.295997 1.484806
H 6.256713 0.797581 -0.662549
H 7.411180 -0.521975 -0.426290
H 6.508969 0.124560 0.958287
H 6.248127 -1.918314 -2.196262
H 4.513205 -2.272399 -2.079250
H 5.063603 -0.634771 -2.477935

12 Energy:-1306.84020717
C -1.574997 3.893170 -1.200214
C -0.593945 4.372546 -0.335575
C 0.001192 3.454772 0.534006
C -0.386660 2.119153 0.509086
C -1.445771 1.644752 -0.313989
C -2.013520 2.576818 -1.204570
N 0.285444 1.106598 1.272036
C -0.667033 0.187403 2.027980
C -1.642105 -0.362932 1.151532
C -1.943023 0.287567 -0.107841
C -2.400058 -1.505329 1.571726
C -3.304371 -2.019909 0.689195
C -3.525639 -1.446448 -0.581224
C -2.881330 -0.307301 -0.991505
C 1.477354 0.586022 0.903071
O -0.483205 0.035985 3.246211
F -2.151983 4.760589 -2.064546
C 1.900617 -0.682605 1.427087
C 3.091586 -1.246860 1.015869
C 3.941853 -0.611032 0.087622
C 3.520119 0.652346 -0.417998
C 2.338777 1.238343 -0.039990

O -4.075631 -3.153585 0.811452
C -4.954851 -3.148120 -0.320464
O -4.431859 -2.217769 -1.276110
C 5.267168 -1.211610 -0.377956
C 5.557328 -2.577534 0.271568
C 6.418745 -0.239527 -0.012756
C 5.222719 -1.399867 -1.916816
H -0.302936 5.416227 -0.357399
H 0.786080 3.771519 1.214001
H -2.819268 2.301065 -1.875492
H -2.205683 -1.958964 2.536826
H -3.102209 0.129557 -1.959554
H 1.297468 -1.173755 2.177161
H 3.370511 -2.205672 1.434496
H 4.143703 1.172946 -1.136683
H 2.044109 2.189599 -0.462769
H -4.981847 -4.146008 -0.762740
H -5.956223 -2.826141 -0.004954
H 4.788273 -3.318444 0.026646
H 6.513635 -2.959375 -0.100486
H 5.633003 -2.504134 1.362114
H 6.297133 0.740733 -0.484391
H 7.372536 -0.657977 -0.353133
H 6.478537 -0.091075 1.070990
H 6.173535 -1.822129 -2.260793
H 4.419690 -2.086764 -2.205562
H 5.067556 -0.453758 -2.444901

13 Energy:-1306.83925325
C -1.550221 3.847380 -1.305642
C -0.615046 4.377736 -0.419818
C -0.032597 3.501179 0.499461
C -0.386092 2.155982 0.502667
C -1.407251 1.631864 -0.338501
C -1.959196 2.522669 -1.281659
N 0.290419 1.184150 1.320987
C -0.668477 0.268309 2.071949
C -1.617690 -0.320207 1.193815
C -1.894410 0.279712 -0.096154
C -2.369480 -1.459646 1.635612
C -3.243605 -2.018404 0.750454
C -3.440854 -1.493235 -0.545346
C -2.802230 -0.360774 -0.980684
C 1.480154 0.643449 0.965008
O -0.490081 0.131245 3.292124
F -2.112749 4.672876 -2.219873
C 1.827279 -0.688243 1.375559
C 2.998697 -1.272126 0.937534

C 3.904554 -0.595869 0.093483
C 3.559019 0.728993 -0.299506
C 2.395099 1.333171 0.102821
O -4.002355 -3.158340 0.893610
C -4.853669 -3.204384 -0.258393
O -4.318840 -2.302089 -1.233979
C 5.210140 -1.218335 -0.397454
C 5.419698 -2.648392 0.134292
C 6.398676 -0.340295 0.072597
C 5.190548 -1.268985 -1.947563
H -0.347682 5.427021 -0.463168
H 0.716993 3.858833 1.198787
H -2.734741 2.207299 -1.970482
H -2.192582 -1.875768 2.620751
H -3.003944 0.036967 -1.969519
H 1.181400 -1.220822 2.058563
H 3.216362 -2.280975 1.264763
H 4.227753 1.283842 -0.948492
H 2.157308 2.332930 -0.235202
H -4.857988 -4.217623 -0.665157
H -5.866265 -2.884281 0.021230
H 4.624064 -3.327009 -0.192455
H 6.366735 -3.041629 -0.249243
H 5.470036 -2.674646 1.228431
H 6.336770 0.681403 -0.315117
H 7.338325 -0.776427 -0.284352
H 6.440301 -0.288038 1.166034
H 6.127854 -1.705552 -2.310108
H 4.362656 -1.888027 -2.310048
H 5.091727 -0.273802 -2.392352

14 Energy:-1306.83846514
C -1.519726 3.783011 -1.414760
C -0.616551 4.355071 -0.521388
C -0.048953 3.516005 0.441134
C -0.384048 2.166645 0.479958
C -1.381564 1.603485 -0.365508
C -1.914954 2.455991 -1.355196
N 0.293251 1.230108 1.343721
C -0.670935 0.325648 2.099052
C -1.611957 -0.290058 1.232385
C -1.871600 0.262709 -0.082196
C -2.365783 -1.416907 1.704003
C -3.226929 -2.008624 0.828337
C -3.408536 -1.529304 -0.488094
C -2.766673 -0.411827 -0.955162
C 1.481997 0.668556 1.000274
O -0.491719 0.210229 3.320750

F -2.065395 4.569958 -2.372680
C 1.755806 -0.712016 1.277925
C 2.916997 -1.300999 0.820152
C 3.884419 -0.582189 0.085889
C 3.608348 0.788598 -0.181817
C 2.452271 1.396938 0.237653
O -3.985452 -3.144819 1.001103
C -4.822489 -3.231982 -0.158675
O -4.276386 -2.363583 -1.158621
C 5.185181 -1.207423 -0.413930
C 5.316107 -2.691506 -0.024027
C 6.384367 -0.432710 0.191463
C 5.232170 -1.099667 -1.960564
H -0.361166 5.405848 -0.591517
H 0.674754 3.907721 1.149399
H -2.668956 2.108388 -2.052370
H -2.200086 -1.797885 2.705177
H -2.956048 -0.050729 -1.960324
H 1.058298 -1.288307 1.868614
H 3.075982 -2.348897 1.041113
H 4.325447 1.377574 -0.743006
H 2.267496 2.435410 -0.002919
H -4.821036 -4.258735 -0.530012
H -5.838710 -2.903427 0.096934
H 4.510899 -3.299368 -0.451172
H 6.263790 -3.083599 -0.407109
H 5.316247 -2.830222 1.062735
H 6.378957 0.624554 -0.091648
H 7.321376 -0.870446 -0.170275
H 6.379268 -0.492907 1.285241
H 6.167886 -1.535706 -2.327777
H 4.398931 -1.644191 -2.417862
H 5.189934 -0.061329 -2.304138

15 Energy:-1306.83799436
C -1.494402 3.700506 -1.528311
C -0.604186 4.305786 -0.643722
C -0.049404 3.501547 0.355419
C -0.383802 2.154164 0.439878
C -1.375039 1.561348 -0.394553
C -1.891527 2.377169 -1.424394
N 0.293918 1.247730 1.338330
C -0.671554 0.361111 2.108804
C -1.622065 -0.272792 1.267526
C -1.876726 0.237024 -0.065461
C -2.383041 -1.378391 1.777508
C -3.249362 -1.992717 0.923237
C -3.427667 -1.556465 -0.409088

C -2.777433 -0.460956 -0.914587
C 1.484320 0.668916 1.004950
O -0.485733 0.278916 3.331754
F -2.025659 4.450967 -2.523102
C 1.692771 -0.742006 1.130951
C 2.854041 -1.322908 0.662874
C 3.884928 -0.564677 0.066348
C 3.668655 0.835459 -0.060979
C 2.509992 1.435799 0.366442
O -4.017074 -3.116265 1.134169
C -4.855873 -3.234942 -0.021371
O -4.302934 -2.405301 -1.050255
C 5.192741 -1.181188 -0.426447
C 5.245183 -2.705586 -0.213573
C 6.374361 -0.536496 0.344013
C 5.348556 -0.890945 -1.941938
H -0.348260 5.353579 -0.747704
H 0.662904 3.920362 1.059536
H -2.635384 2.004146 -2.119297
H -2.218994 -1.726821 2.790767
H -2.965024 -0.133895 -1.931608
H 0.937700 -1.355797 1.601926
H 2.960770 -2.395814 0.761513
H 4.433450 1.455694 -0.515294
H 2.371481 2.501058 0.236784
H -4.863730 -4.273479 -0.358204
H -5.868925 -2.888895 0.223368
H 4.447823 -3.222774 -0.758704
H 6.200882 -3.089692 -0.584603
H 5.170880 -2.973293 0.846171
H 6.422284 0.546776 0.195078
H 7.318351 -0.965019 -0.010678
H 6.294226 -0.729537 1.419316
H 6.288645 -1.324510 -2.300793
H 4.527031 -1.335631 -2.514046
H 5.372429 0.181820 -2.157685

16 Energy:-1306.83763537
C -1.467876 3.610168 -1.638617
C -0.580127 4.242815 -0.770698
C -0.038989 3.472926 0.262208
C -0.384931 2.132461 0.398121
C -1.377609 1.515958 -0.419953
C -1.876354 2.295006 -1.486700
N 0.290581 1.252476 1.324359
C -0.676530 0.386892 2.111356
C -1.639871 -0.262648 1.298933
C -1.892428 0.208581 -0.048531

C -2.410512 -1.343230 1.846477
C -3.286381 -1.974656 1.014654
C -3.463179 -1.578603 -0.330622
C -2.802878 -0.506747 -0.872717
C 1.487522 0.659792 1.004871
O -0.490460 0.353214 3.336715
F -1.984123 4.324676 -2.666864
C 1.638982 -0.759577 0.967442
C 2.806143 -1.327227 0.495517
C 3.896201 -0.544160 0.056671
C 3.733861 0.866822 0.087512
C 2.568215 1.455418 0.518175
O -4.066109 -3.082027 1.262752
C -4.908401 -3.228735 0.112995
O -4.349544 -2.436746 -0.942336
C 5.210256 -1.149473 -0.435078
C 5.202914 -2.689007 -0.397003
C 6.367536 -0.641419 0.463402
C 5.458141 -0.695075 -1.897025
H -0.315830 5.284073 -0.912293
H 0.669594 3.914821 0.955454
H -2.614778 1.900396 -2.175375
H -2.246720 -1.661502 2.869691
H -2.991004 -0.210762 -1.899049
H 0.829488 -1.393254 1.304645
H 2.868090 -2.407701 0.460306
H 4.545141 1.506919 -0.241403
H 2.471844 2.533481 0.515905
H -4.926731 -4.277106 -0.191377
H -5.917526 -2.865392 0.348359
H 4.423682 -3.110667 -1.041511
H 6.166526 -3.064184 -0.756607
H 5.057496 -3.072213 0.619022
H 6.459097 0.449040 0.441347
H 7.315387 -1.063698 0.111395
H 6.221773 -0.950251 1.504266
H 6.403444 -1.118100 -2.254883
H 4.656651 -1.042499 -2.558008
H 5.524852 0.393811 -1.985637

17 Energy:-1306.88289445
C -1.591292 3.215713 -1.863015
C -0.253003 2.930106 -2.176025
C 0.300776 1.773867 -1.631501
C -0.453054 0.933437 -0.783982
C -1.874918 1.187594 -0.549264
C -2.392684 2.397060 -1.087179
N 0.151565 -0.126415 -0.133298

C -0.615861 -1.212533 0.454121
C -2.027121 -1.007855 0.594390
C -2.653405 0.232760 0.157166
C -2.786422 -2.029353 1.223092
C -4.123837 -1.803600 1.413284
C -4.743838 -0.589513 1.012644
C -4.058581 0.416498 0.400845
C 1.578043 -0.253421 -0.119863
O 0.014406 -2.214246 0.811669
F -2.130093 4.343886 -2.364454
C 2.221320 -1.266758 -0.836175
C 3.613751 -1.359607 -0.808463
C 4.400772 -0.455747 -0.079752
C 3.726546 0.562466 0.625738
C 2.342139 0.672764 0.604474
O -5.054204 -2.612244 2.006077
C -6.319344 -1.951521 1.856734
O -6.069214 -0.631058 1.359446
C 5.935373 -0.537860 -0.026108
C 6.490356 -1.703961 -0.865705
C 6.385274 -0.741070 1.441220
C 6.540459 0.779662 -0.568316
H 0.313026 3.591404 -2.820883
H 1.330757 1.519368 -1.845607
H -3.419761 2.694885 -0.920806
H -2.302344 -2.943591 1.545905
H -4.571939 1.324192 0.108652
H 1.635575 -1.975603 -1.410542
H 4.081260 -2.155897 -1.375986
H 4.290975 1.281921 1.211173
H 1.843676 1.459758 1.162446
H -6.934142 -2.504751 1.137344
H -6.809706 -1.886689 2.830818
H 6.228980 -1.604981 -1.925436
H 7.583539 -1.717644 -0.794844
H 6.124006 -2.673767 -0.510545
H 6.058925 0.081362 2.086328
H 7.479179 -0.794721 1.495912
H 5.978000 -1.672644 1.850460
H 7.635382 0.736681 -0.526605
H 6.246425 0.946490 -1.610993
H 6.218799 1.648265 0.015674

18 Energy:-1306.88547947
C -1.626069 3.127951 -2.021015
C -0.252726 2.906610 -2.213597
C 0.322054 1.803358 -1.580940
C -0.450647 0.945279 -0.772024

C -1.895012 1.146962 -0.630939
C -2.438605 2.304380 -1.268906
N 0.152636 -0.089330 -0.077550
C -0.590456 -1.122149 0.591020
C -2.018452 -0.957858 0.673896
C -2.670590 0.210788 0.091024
C -2.757769 -1.942834 1.364972
C -4.115473 -1.761893 1.473826
C -4.766622 -0.625351 0.922054
C -4.098266 0.348836 0.247032
C 1.587315 -0.223715 -0.075088
O 0.049678 -2.068653 1.064557
F -2.180052 4.204554 -2.616239
C 2.215783 -1.141716 -0.914378
C 3.608605 -1.250879 -0.904068
C 4.403275 -0.451246 -0.070390
C 3.740447 0.473622 0.759386
C 2.354113 0.595212 0.758320
O -5.037025 -2.562688 2.083164
C -6.309626 -1.916308 1.932493
O -6.106342 -0.710217 1.186785
C 5.938693 -0.549477 -0.035186
C 6.480326 -1.615107 -1.006744
C 6.396257 -0.922853 1.395406
C 6.549189 0.817549 -0.427565
H 0.328153 3.578428 -2.834258
H 1.378934 1.607108 -1.702707
H -3.488288 2.552723 -1.185705
H -2.252296 -2.800482 1.792971
H -4.635439 1.195835 -0.160139
H 1.620590 -1.771099 -1.568286
H 4.067703 -1.976816 -1.565128
H 4.312666 1.112530 1.425283
H 1.862633 1.312592 1.408760
H -6.986347 -2.577120 1.382641
H -6.709412 -1.672513 2.921740
H 6.215828 -1.392340 -2.046721
H 7.573644 -1.645906 -0.943453
H 6.106751 -2.616407 -0.764257
H 6.080095 -0.178167 2.133314
H 7.490045 -0.989670 1.436294
H 5.984669 -1.892564 1.697936
H 7.644132 0.764260 -0.399464
H 6.249075 1.104387 -1.442076
H 6.236750 1.615478 0.254213

19 Energy:-1306.88675061
C -1.672790 2.998748 -2.223807

C -0.273253 2.894683 -2.259503
C 0.324397 1.873238 -1.516205
C -0.453811 0.977114 -0.757218
C -1.913916 1.092587 -0.725543
C -2.486331 2.153394 -1.499891
N 0.157244 -0.031820 -0.029702
C -0.560255 -0.972114 0.773187
C -1.998671 -0.857768 0.803529
C -2.678436 0.186317 0.040581
C -2.716537 -1.781521 1.588200
C -4.087967 -1.668752 1.611238
C -4.768979 -0.662311 0.873843
C -4.120449 0.251971 0.103303
C 1.595230 -0.170095 -0.044964
O 0.096148 -1.821322 1.388234
F -2.249491 3.986409 -2.941633
C 2.207811 -0.972722 -1.003112
C 3.598842 -1.107170 -1.009881
C 4.404418 -0.446942 -0.070974
C 3.755883 0.359842 0.881874
C 2.370482 0.502931 0.900265
O -4.994879 -2.432186 2.284832
C -6.292190 -1.904708 1.971224
O -6.113607 -0.794358 1.084324
C 5.938352 -0.575363 -0.049898
C 6.464604 -1.497374 -1.166097
C 6.385147 -1.160503 1.311306
C 6.573083 0.823285 -0.239162
H 0.312106 3.590721 -2.848610
H 1.400744 1.767229 -1.523562
H -3.555681 2.312897 -1.532096
H -2.189229 -2.546606 2.145622
H -4.680309 1.000406 -0.442166
H 1.601956 -1.495158 -1.737387
H 4.046485 -1.743105 -1.764737
H 4.337800 0.888869 1.630603
H 1.889755 1.128133 1.646649
H -6.885107 -2.678719 1.474541
H -6.774629 -1.564033 2.892347
H 6.205557 -1.121769 -2.162587
H 7.557184 -1.554634 -1.108424
H 6.074800 -2.517209 -1.071340
H 6.079212 -0.525402 2.149223
H 7.477509 -1.251577 1.342841
H 5.956074 -2.156541 1.469659
H 7.666851 0.747329 -0.219536
H 6.280161 1.260079 -1.200912
H 6.273207 1.518687 0.551784

20 Energy:-1306.88568317
C -1.689301 2.856036 -2.400265
C -0.288855 2.875820 -2.299592
C 0.315037 1.938695 -1.459495
C -0.456384 1.001268 -0.742954
C -1.919816 1.027495 -0.808432
C -2.497510 1.984123 -1.699729
N 0.163986 0.025918 0.019304
C -0.548095 -0.835297 0.922295
C -1.986551 -0.766265 0.902249
C -2.677689 0.147575 -0.002351
C -2.700840 -1.630779 1.759918
C -4.073556 -1.586043 1.711870
C -4.764223 -0.712275 0.828819
C -4.120709 0.139448 -0.014784
C 1.598132 -0.114995 -0.009198
O 0.121081 -1.588031 1.640002
F -2.274253 3.750244 -3.224207
C 2.202602 -0.792721 -1.066538
C 3.589728 -0.950140 -1.090142
C 4.403749 -0.438095 -0.067922
C 3.764866 0.240099 0.985985
C 2.382305 0.402147 1.024636
O -4.977000 -2.316540 2.427580
C -6.280036 -1.871591 2.022666
O -6.110804 -0.895438 0.987990
C 5.935080 -0.593223 -0.063695
C 6.449091 -1.365644 -1.293411
C 6.369850 -1.362799 1.206721
C 6.592131 0.808056 -0.063727
H 0.290127 3.599555 -2.860817
H 1.392400 1.922853 -1.362173
H -3.568611 2.052206 -1.835990
H -2.166601 -2.303234 2.420686
H -4.687175 0.788883 -0.669737
H 1.589909 -1.203086 -1.863744
H 4.028287 -1.488690 -1.922106
H 4.353311 0.652294 1.800247
H 1.910624 0.925591 1.850215
H -6.846439 -2.722477 1.633647
H -6.786946 -1.414583 2.878742
H 6.198471 -0.855767 -2.230614
H 7.540465 -1.447924 -1.243843
H 6.042689 -2.382512 -1.336290
H 6.073444 -0.840927 2.122577
H 7.460505 -1.475452 1.224870
H 5.924055 -2.363778 1.230417

H 7.684528 0.712937 -0.053227
H 6.309086 1.373925 -0.958848
H 6.300377 1.396222 0.812658

21 Energy:-1306.88273569
C -1.687667 2.713703 -2.546740
C -0.306133 2.852915 -2.342564
C 0.293446 2.004476 -1.414798
C -0.459413 1.030880 -0.723028
C -1.913250 0.963071 -0.871980
C -2.483003 1.817980 -1.853217
N 0.173752 0.100611 0.080565
C -0.547107 -0.677330 1.078107
C -1.977012 -0.670076 0.993818
C -2.666250 0.094294 -0.037273
C -2.699679 -1.475651 1.913497
C -4.062611 -1.518844 1.788726
C -4.745949 -0.798774 0.772141
C -4.097274 -0.007801 -0.127818
C 1.597469 -0.044484 0.038291
O 0.131981 -1.304082 1.899077
F -2.275635 3.513646 -3.457019
C 2.195005 -0.600770 -1.098303
C 3.575162 -0.785161 -1.143212
C 4.400600 -0.423677 -0.064496
C 3.773920 0.131221 1.066770
C 2.396718 0.317412 1.130507
O -4.969485 -2.229193 2.526764
C -6.267924 -1.841026 2.054884
O -6.088105 -1.061686 0.865859
C 5.926862 -0.612665 -0.081450
C 6.426080 -1.234030 -1.399632
C 6.341174 -1.546587 1.081409
C 6.615795 0.762134 0.096498
H 0.257288 3.599491 -2.889115
H 1.357194 2.077972 -1.228104
H -3.543881 1.804209 -2.067051
H -2.170009 -2.040313 2.671764
H -4.657851 0.525140 -0.885834
H 1.574622 -0.900942 -1.937466
H 4.002054 -1.229775 -2.034617
H 4.371032 0.428775 1.923585
H 1.938689 0.746205 2.014523
H -6.848526 -2.735813 1.818986
H -6.765013 -1.234016 2.820516
H 6.188624 -0.603764 -2.264197
H 7.515234 -1.346076 -1.361698
H 5.997586 -2.228060 -1.570842

H 6.053253 -1.138462 2.055804
H 7.429136 -1.683989 1.085019
H 5.874023 -2.532593 0.977814
H 7.705620 0.640772 0.092764
H 6.347112 1.444066 -0.718505
H 6.336874 1.239755 1.041534

22 Energy:-1306.88062702
C -1.671752 2.607530 -2.641733
C -0.309045 2.816057 -2.390967
C 0.282109 2.034293 -1.402221
C -0.456615 1.054186 -0.703705
C -1.896486 0.924671 -0.898916
C -2.457660 1.709166 -1.934750
N 0.183889 0.181661 0.159736
C -0.553886 -0.553129 1.196595
C -1.974326 -0.595288 1.060950
C -2.649849 0.065574 -0.044771
C -2.709247 -1.361967 2.012522
C -4.059769 -1.469421 1.836496
C -4.724741 -0.855463 0.742149
C -4.066127 -0.103132 -0.186124
C 1.594946 0.012982 0.102539
O 0.118449 -1.100374 2.077364
F -2.259368 3.338769 -3.606991
C 2.182834 -0.449728 -1.086707
C 3.555624 -0.658542 -1.156291
C 4.395256 -0.411718 -0.054486
C 3.784228 0.055367 1.125382
C 2.413429 0.264299 1.216830
O -4.972563 -2.168412 2.583127
C -6.261861 -1.841404 2.046133
O -6.058684 -1.173817 0.794106
C 5.916102 -0.626913 -0.099265
C 6.394728 -1.148593 -1.467381
C 6.320277 -1.658989 0.981687
C 6.631759 0.716734 0.184115
H 0.244830 3.565551 -2.942971
H 1.331295 2.166478 -1.168985
H -3.508879 1.646180 -2.185510
H -2.191190 -1.850414 2.829437
H -4.613278 0.349110 -1.004467
H 1.552945 -0.667695 -1.943663
H 3.968135 -1.033952 -2.085447
H 4.392814 0.271011 1.998454
H 1.972809 0.633292 2.134360
H -6.827690 -2.760453 1.879454
H -6.785268 -1.171275 2.738960

H 6.165712 -0.445241 -2.276008
H 7.481478 -1.285176 -1.446573
H 5.944745 -2.116490 -1.715776
H 6.047289 -1.325294 1.988132
H 7.405432 -1.815738 0.964587
H 5.833973 -2.624736 0.803068
H 7.718934 0.575376 0.162255
H 6.371202 1.467009 -0.571197
H 6.367585 1.121145 1.166732

23 Energy:-1306.87833346
C -1.654108 2.516218 -2.717629
C -0.311836 2.786252 -2.432477
C 0.270529 2.065557 -1.392943
C -0.452873 1.082894 -0.683467
C -1.875536 0.894823 -0.916292
C -2.428975 1.615737 -1.996979
N 0.194423 0.275132 0.242333
C -0.563242 -0.426720 1.304473
C -1.971706 -0.525231 1.116920
C -2.628992 0.042623 -0.047436
C -2.719070 -1.259752 2.090349
C -4.054695 -1.429029 1.871118
C -4.697093 -0.909596 0.716958
C -4.028071 -0.188652 -0.230978
C 1.590980 0.068665 0.166910
O 0.092453 -0.898199 2.239344
F -2.239979 3.184492 -3.728354
C 2.169466 -0.315715 -1.060675
C 3.534465 -0.549465 -1.154959
C 4.387846 -0.400828 -0.044998
C 3.792814 -0.003672 1.170023
C 2.429914 0.229072 1.288584
O -4.972262 -2.120843 2.622416
C -6.249701 -1.856933 2.027145
O -6.019627 -1.279510 0.735323
C 5.902031 -0.643125 -0.117083
C 6.360300 -1.081857 -1.520735
C 6.292483 -1.752583 0.890225
C 6.646686 0.664863 0.249311
H 0.233220 3.536043 -2.992642
H 1.303788 2.251224 -1.125756
H -3.469245 1.505664 -2.276296
H -2.215823 -1.678328 2.953882
H -4.558545 0.192267 -1.095602
H 1.531427 -0.466370 -1.925522
H 3.931940 -0.868524 -2.111311
H 4.412601 0.144692 2.049138

H 2.007506 0.553382 2.229706
H -6.796222 -2.795144 1.912231
H -6.804492 -1.146548 2.652859
H 6.142032 -0.320931 -2.278553
H 7.443792 -1.243019 -1.517336
H 5.887466 -2.020940 -1.829759
H 6.032725 -1.481868 1.918774
H 7.374284 -1.927716 0.853863
H 5.786708 -2.694944 0.651225
H 7.730367 0.502471 0.209889
H 6.397187 1.468153 -0.453270
H 6.397185 1.008902 1.258319

24 Energy:-1306.87107662
C -1.642995 2.479910 -2.771040
C -0.342490 2.860153 -2.442134
C 0.238994 2.233937 -1.341613
C -0.445710 1.235832 -0.620591
C -1.820581 0.914704 -0.919470
C -2.380497 1.549451 -2.041610
N 0.210833 0.519645 0.379550
C -0.591108 -0.029393 1.529733
C -1.941761 -0.339143 1.221330
C -2.539612 0.011316 -0.052849
C -2.714868 -1.019908 2.221610
C -3.988331 -1.382267 1.899487
C -4.551921 -1.100620 0.630712
C -3.866821 -0.414232 -0.337107
C 1.577272 0.235004 0.272555
O -0.011005 -0.182529 2.609681
F -2.234943 3.057885 -3.832842
C 2.112860 -0.135974 -0.987718
C 3.456456 -0.444885 -1.127024
C 4.343980 -0.398802 -0.034703
C 3.797683 -0.024315 1.213350
C 2.458351 0.285938 1.379952
O -4.897951 -2.093480 2.645597
C -6.122987 -2.064556 1.901506
O -5.814048 -1.642914 0.566774
C 5.836405 -0.728060 -0.154015
C 6.239494 -1.117388 -1.588681
C 6.176201 -1.911497 0.786066
C 6.667152 0.511488 0.264416
H 0.174498 3.620509 -3.014774
H 1.241913 2.507762 -1.033713
H -3.397511 1.349368 -2.356342
H -2.266918 -1.259780 3.178586
H -4.338155 -0.205226 -1.290843

H 1.448165 -0.223942 -1.840226
H 3.810019 -0.748230 -2.105523
H 4.445699 0.047876 2.081491
H 2.078691 0.592797 2.342944
H -6.553553 -3.067442 1.873698
H -6.814284 -1.346982 2.362004
H 6.051253 -0.305749 -2.300507
H 7.311349 -1.340937 -1.618559
H 5.706325 -2.009976 -1.934836
H 5.951662 -1.681366 1.832578
H 7.244691 -2.147566 0.717461
H 5.610587 -2.807950 0.508128
H 7.737279 0.284691 0.190762
H 6.454211 1.365194 -0.388852
H 6.460549 0.814735 1.295870

25 Energy:-1306.84864298
C -1.874193 2.576088 -2.851443
C -0.622663 3.117842 -2.573459
C 0.078712 2.595741 -1.486139
C -0.470339 1.555985 -0.727420
C -1.775622 1.034542 -0.991966
C -2.462181 1.566697 -2.089151
N 0.233995 0.889517 0.286278
C -0.576490 0.654965 1.566285
C -1.787493 -0.030811 1.264233
C -2.326332 0.027979 -0.076151
C -2.519551 -0.679067 2.308601
C -3.657431 -1.349149 1.954644
C -4.127423 -1.381712 0.626433
C -3.501331 -0.697700 -0.388504
C 1.548747 0.524968 0.190672
O -0.187672 1.172505 2.624255
F -2.569206 3.061104 -3.900306
C 2.126550 0.207754 -1.074805
C 3.428382 -0.245625 -1.162464
C 4.232901 -0.436193 -0.016947
C 3.636194 -0.160249 1.234472
C 2.334065 0.285182 1.356371
O -4.475777 -2.124107 2.741984
C -5.595937 -2.471296 1.916362
O -5.241101 -2.187737 0.556881
C 5.680556 -0.931153 -0.087241
C 6.144721 -1.191825 -1.532644
C 5.810860 -2.251820 0.712796
C 6.611602 0.138789 0.538215
H -0.221394 3.915864 -3.187156
H 1.058304 2.985007 -1.227891

H -3.454513 1.225524 -2.361296
H -2.140717 -0.674386 3.324300
H -3.913517 -0.705594 -1.392133
H 1.524457 0.300327 -1.971222
H 3.818215 -0.486070 -2.144403
H 4.212402 -0.299286 2.143865
H 1.909555 0.506179 2.326056
H -5.806752 -3.537401 2.021612
H -6.464083 -1.863561 2.203135
H 6.101956 -0.284557 -2.145687
H 7.184367 -1.536040 -1.524500
H 5.544358 -1.967312 -2.021590
H 5.540785 -2.122679 1.765740
H 6.846991 -2.607852 0.674972
H 5.166923 -3.031463 0.290760
H 7.650976 -0.208154 0.503984
H 6.549391 1.083922 -0.012748
H 6.360277 0.339403 1.584623

26 Energy:-1306.84627099
C -1.910508 2.543304 -2.919704
C -0.698250 3.165525 -2.638504
C 0.019514 2.709751 -1.530608
C -0.478504 1.658542 -0.758572
C -1.744822 1.055460 -1.022128
C -2.447140 1.518677 -2.142584
N 0.247528 1.057307 0.289626
C -0.561580 0.818968 1.564316
C -1.727703 0.055726 1.267596
C -2.251892 0.048789 -0.080349
C -2.438486 -0.600565 2.321461
C -3.535147 -1.339181 1.974553
C -3.985201 -1.434305 0.642689
C -3.382505 -0.748321 -0.384487
C 1.532668 0.625622 0.186549
O -0.208302 1.385565 2.610616
F -2.618434 2.962537 -3.989901
C 2.132507 0.354474 -1.081731
C 3.414429 -0.149762 -1.158499
C 4.180774 -0.434231 -0.003770
C 3.565450 -0.195114 1.246798
C 2.282340 0.300020 1.360040
O -4.321622 -2.134253 2.774660
C -5.411325 -2.562886 1.946782
O -5.054532 -2.299264 0.583812
C 5.607254 -0.986003 -0.065954
C 6.096903 -1.194455 -1.511399
C 5.661675 -2.349092 0.669596

C 6.565510 0.011550 0.634220
H -0.335540 3.970540 -3.266924
H 0.973022 3.159036 -1.271439
H -3.414185 1.113313 -2.417857
H -2.073097 -0.547108 3.340686
H -3.780329 -0.807803 -1.392130
H 1.562385 0.523904 -1.987114
H 3.822596 -0.353909 -2.141087
H 4.112906 -0.403206 2.160517
H 1.847195 0.499671 2.329675
H -5.567476 -3.635247 2.080225
H -6.313789 -1.993992 2.206083
H 6.108512 -0.256828 -2.078374
H 7.120516 -1.583312 -1.495800
H 5.477358 -1.918056 -2.053003
H 5.371679 -2.260931 1.721449
H 6.682690 -2.746533 0.636649
H 4.996295 -3.078356 0.194228
H 7.589610 -0.378463 0.607179
H 6.557418 0.983893 0.129184
H 6.296159 0.172089 1.682992

27 Energy:-1306.84428315
C -1.927331 2.503089 -2.987009
C -0.768073 3.214886 -2.694784
C -0.043806 2.832381 -1.562678
C -0.485856 1.767221 -0.780360
C -1.701855 1.074970 -1.049852
C -2.408797 1.462054 -2.198477
N 0.260787 1.238746 0.301730
C -0.552341 0.984038 1.566605
C -1.665295 0.144828 1.270460
C -2.166398 0.070818 -0.085245
C -2.355536 -0.522705 2.331541
C -3.402459 -1.331637 1.989837
C -3.824370 -1.489254 0.654473
C -3.244551 -0.799644 -0.382486
C 1.514307 0.737738 0.191055
O -0.234829 1.578688 2.609416
F -2.638018 2.849114 -4.082803
C 2.132632 0.501010 -1.076946
C 3.391086 -0.056730 -1.147632
C 4.118957 -0.425534 0.010157
C 3.491270 -0.206210 1.258971
C 2.231548 0.343028 1.367524
O -4.155379 -2.148884 2.800441
C -5.205440 -2.660987 1.969134
O -4.841842 -2.415733 0.604658

C 5.518063 -1.042111 -0.048663
C 6.029744 -1.209283 -1.491882
C 5.488579 -2.438073 0.624438
C 6.507094 -0.126011 0.717175
H -0.446782 4.028329 -3.334793
H 0.873531 3.348285 -1.296544
H -3.340236 0.985705 -2.482749
H -2.009880 -0.420793 3.353954
H -3.621056 -0.911163 -1.393860
H 1.594443 0.740293 -1.985522
H 3.814888 -0.232997 -2.128837
H 4.011643 -0.472307 2.173166
H 1.790341 0.531660 2.336585
H -5.303553 -3.736346 2.130961
H -6.142629 -2.137108 2.198652
H 6.100519 -0.248917 -2.014711
H 7.032323 -1.649193 -1.472754
H 5.388216 -1.876137 -2.078755
H 5.177389 -2.383024 1.672427
H 6.490175 -2.882244 0.595912
H 4.801417 -3.112014 0.100998
H 7.511780 -0.563198 0.688625
H 6.554927 0.868926 0.260855
H 6.225174 -0.004485 1.767855

28 Energy:-1306.84277725
C -1.930675 2.450226 -3.046374
C -0.824196 3.238684 -2.744608
C -0.100373 2.920423 -1.592169
C -0.491768 1.844203 -0.800835
C -1.660087 1.078041 -1.074022
C -2.364006 1.398315 -2.247300
N 0.272324 1.380978 0.307609
C -0.546992 1.112503 1.564234
C -1.615881 0.217591 1.271871
C -2.094199 0.084139 -0.088401
C -2.291460 -0.452271 2.342084
C -3.296609 -1.315090 2.009689
C -3.692895 -1.527186 0.673390
C -3.129126 -0.842251 -0.374382
C 1.496715 0.819079 0.193075
O -0.250249 1.717535 2.607246
F -2.637511 2.731737 -4.164205
C 2.145076 0.634941 -1.069870
C 3.384204 0.037361 -1.136939
C 4.067232 -0.418240 0.018591
C 3.417534 -0.235704 1.262878
C 2.177652 0.354656 1.368493

O -4.024364 -2.143406 2.832650
C -5.041601 -2.719504 2.002700
O -4.668501 -2.498831 0.636853
C 5.443450 -1.082954 -0.038254
C 5.986318 -1.197371 -1.475229
C 5.343403 -2.508717 0.562208
C 6.444852 -0.244517 0.797556
H -0.539557 4.058217 -3.394118
H 0.780694 3.493339 -1.319988
H -3.260511 0.862415 -2.537831
H -1.964389 -0.307412 3.365413
H -3.485577 -0.998633 -1.387037
H 1.644449 0.946790 -1.977413
H 3.832100 -0.097022 -2.113868
H 3.906592 -0.562012 2.174612
H 1.724232 0.519026 2.336170
H -5.100161 -3.792860 2.194145
H -6.001737 -2.226633 2.204824
H 6.108200 -0.215591 -1.946279
H 6.970332 -1.677132 -1.453645
H 5.335730 -1.808074 -2.111143
H 5.008176 -2.493984 1.604064
H 6.327960 -2.989282 0.534232
H 4.644880 -3.128275 -0.010956
H 7.432862 -0.718003 0.770377
H 6.541170 0.769519 0.394105
H 6.140811 -0.165676 1.846060

29 Energy:-1306.8413697
C -1.932555 2.393916 -3.098172
C -0.872044 3.243013 -2.793429
C -0.149546 2.978543 -1.626696
C -0.497110 1.896278 -0.824621
C -1.625507 1.072885 -1.095440
C -2.325309 1.337212 -2.287059
N 0.282022 1.488619 0.302838
C -0.544202 1.216647 1.554195
C -1.579324 0.281138 1.271869
C -2.037815 0.094057 -0.089570
C -2.242858 -0.384896 2.353163
C -3.213601 -1.290099 2.034141
C -3.588716 -1.550889 0.699644
C -3.037693 -0.875684 -0.360163
C 1.480229 0.873826 0.188599
O -0.256193 1.828931 2.595364
F -2.635199 2.621497 -4.231834
C 2.172741 0.760629 -1.060043
C 3.396849 0.134123 -1.124012

C 4.025637 -0.415235 0.022126
C 3.340991 -0.287757 1.255285
C 2.116998 0.333282 1.357875
O -3.921034 -2.122120 2.871583
C -4.910832 -2.751736 2.047649
O -4.530089 -2.556699 0.680257
C 5.384551 -1.113638 -0.031441
C 5.966906 -1.167134 -1.456446
C 5.229324 -2.565792 0.488908
C 6.380945 -0.347367 0.876826
H -0.619045 4.065415 -3.452325
H 0.699387 3.597500 -1.353121
H -3.192185 0.754751 -2.578001
H -1.931305 -0.202134 3.375244
H -3.376891 -1.071616 -1.371830
H 1.716633 1.150624 -1.960477
H 3.879581 0.056879 -2.090318
H 3.792043 -0.680048 2.160426
H 1.641837 0.457167 2.320855
H -4.938371 -3.820818 2.267827
H -5.887958 -2.283800 2.226657
H 6.132659 -0.165849 -1.869387
H 6.935024 -1.677977 -1.432874
H 5.317804 -1.721944 -2.143071
H 4.863894 -2.597413 1.520114
H 6.201767 -3.070350 0.463211
H 4.532840 -3.135062 -0.136457
H 7.356584 -0.845830 0.852899
H 6.515624 0.683379 0.530834
H 6.047038 -0.315336 1.918664

30 Energy:-1306.84013661
C -1.939135 2.322812 -3.150452
C -0.916787 3.220459 -2.852303
C -0.194707 3.005760 -1.675311
C -0.504462 1.924949 -0.856488
C -1.600885 1.056377 -1.116470
C -2.297767 1.269218 -2.321727
N 0.289564 1.569763 0.284986
C -0.540821 1.301411 1.534349
C -1.551723 0.336122 1.270233
C -1.996749 0.098189 -0.088097
C -2.203091 -0.318584 2.366837
C -3.148069 -1.256578 2.068540
C -3.509998 -1.563259 0.739459
C -2.970669 -0.904503 -0.335845
C 1.467051 0.913157 0.174531
O -0.251715 1.920556 2.570912

F -2.639140 2.500410 -4.295395
C 2.216242 0.885114 -1.046455
C 3.431229 0.241494 -1.105641
C 3.998626 -0.404757 0.021978
C 3.267468 -0.345674 1.234161
C 2.054609 0.296106 1.332272
O -3.838509 -2.083903 2.925123
C -4.809973 -2.757335 2.114481
O -4.425766 -2.592953 0.744191
C 5.341629 -1.132663 -0.027044
C 5.985236 -1.094481 -1.425875
C 5.126202 -2.615016 0.373497
C 6.315403 -0.466040 0.979137
H -0.690849 4.040310 -3.524068
H 0.626705 3.663035 -1.406923
H -3.140595 0.649380 -2.606084
H -1.901162 -0.099944 3.384706
H -3.298705 -1.137668 -1.343267
H 1.809415 1.355157 -1.932032
H 3.958562 0.233549 -2.051380
H 3.674835 -0.807221 2.127041
H 1.549128 0.367548 2.284839
H -4.816191 -3.820112 2.364698
H -5.797848 -2.305477 2.275242
H 6.194910 -0.069870 -1.752499
H 6.937915 -1.633389 -1.400468
H 5.353567 -1.576457 -2.180328
H 4.714088 -2.713493 1.382632
H 6.085965 -3.143286 0.350996
H 4.444592 -3.115548 -0.322955
H 7.278618 -0.988015 0.955989
H 6.489932 0.584161 0.720684
H 5.938352 -0.505726 2.005856

31 Energy:-1306.83890187
C -1.945503 2.249043 -3.195393
C -0.950461 3.180879 -2.908382
C -0.228864 3.006494 -1.724568
C -0.511593 1.932487 -0.887489
C -1.586743 1.033386 -1.132863
C -2.280665 1.203879 -2.347918
N 0.296319 1.619010 0.262353
C -0.535401 1.359640 1.512248
C -1.535724 0.379187 1.269772
C -1.974313 0.099074 -0.082834
C -2.178675 -0.257802 2.382578
C -3.110173 -1.215418 2.107588
C -3.466831 -1.560441 0.785843

C -2.934939 -0.922951 -0.305281
C 1.455228 0.924853 0.153253
O -0.234934 1.982763 2.542823
F -2.643230 2.385534 -4.347943
C 2.268449 0.994500 -1.024626
C 3.479975 0.344361 -1.079052
C 3.983583 -0.401517 0.016422
C 3.195928 -0.428809 1.194716
C 1.988410 0.222921 1.288398
O -3.790772 -2.030478 2.984053
C -4.754463 -2.733638 2.189871
O -4.369306 -2.601502 0.816427
C 5.319964 -1.141064 -0.027385
C 6.028119 -1.004943 -1.388382
C 5.070987 -2.646573 0.247957
C 6.251898 -0.565760 1.070721
H -0.743560 3.994680 -3.593476
H 0.572265 3.691536 -1.464390
H -3.106743 0.557748 -2.622866
H -1.880465 -0.009295 3.394633
H -3.258059 -1.186844 -1.306640
H 1.912369 1.547299 -1.883927
H 4.057924 0.416413 -1.991816
H 3.556457 -0.964605 2.065934
H 1.442613 0.224326 2.220949
H -4.751379 -3.789545 2.467597
H -5.746815 -2.286923 2.337162
H 6.266530 0.038193 -1.623852
H 6.971382 -1.560043 -1.361895
H 5.425855 -1.416220 -2.206105
H 4.610231 -2.816042 1.226112
H 6.025612 -3.184087 0.229997
H 4.418734 -3.083418 -0.516122
H 7.210631 -1.095939 1.050271
H 6.446952 0.499041 0.902697
H 5.827719 -0.680302 2.073093

32 Energy:-1306.83766887
C -1.956377 2.166719 -3.239632
C -0.981858 3.125536 -2.970480
C -0.259201 2.990174 -1.782286
C -0.520359 1.928778 -0.922528
C -1.580395 1.006091 -1.147558
C -2.273023 1.135417 -2.369567
N 0.302136 1.653354 0.232055
C -0.527399 1.408629 1.485094
C -1.524472 0.419578 1.269839
C -1.961796 0.099052 -0.074248

C -2.159179 -0.194126 2.400948
C -3.083417 -1.165665 2.153585
C -3.440133 -1.548029 0.841646
C -2.915208 -0.936253 -0.267060
C 1.445975 0.927046 0.122997
O -0.213510 2.041765 2.505086
F -2.653419 2.263356 -4.397093
C 2.327737 1.099352 -0.993281
C 3.539391 0.448578 -1.040275
C 3.976414 -0.398237 0.008254
C 3.124438 -0.522191 1.135509
C 1.919166 0.134347 1.222843
O -3.756174 -1.962946 3.052453
C -4.716478 -2.693086 2.278986
O -4.334961 -2.594298 0.901739
C 5.309430 -1.143632 -0.028547
C 6.092931 -0.891704 -1.330495
C 5.037205 -2.665368 0.093919
C 6.181284 -0.679403 1.167173
H -0.790432 3.929173 -3.671847
H 0.525719 3.698425 -1.535220
H -3.086571 0.467957 -2.630723
H -1.860292 0.083182 3.405296
H -3.237758 -1.229887 -1.260233
H 2.023515 1.735815 -1.813929
H 4.171489 0.605415 -1.905165
H 3.433259 -1.137659 1.973278
H 1.323436 0.052372 2.120573
H -4.705577 -3.741353 2.584115
H -5.711668 -2.249593 2.416573
H 6.350292 0.165872 -1.455947
H 7.029938 -1.457214 -1.303328
H 5.534522 -1.220134 -2.214140
H 4.519817 -2.918411 1.024529
H 5.988643 -3.208585 0.081380
H 4.428134 -3.024235 -0.742925
H 7.137218 -1.214681 1.150738
H 6.390041 0.394330 1.107893
H 5.702049 -0.880534 2.130270

33 Energy:-1306.83647347
C -1.975951 2.079210 -3.280924
C -1.012409 3.056030 -3.037232
C -0.285461 2.956741 -1.848119
C -0.531957 1.914055 -0.961598
C -1.584942 0.976214 -1.159681
C -2.279706 1.067152 -2.384785
N 0.306766 1.670119 0.192507

C -0.514911 1.446058 1.452366
C -1.515999 0.455220 1.270743
C -1.960500 0.098712 -0.061909
C -2.140967 -0.130532 2.422363
C -3.065610 -1.108901 2.206828
C -3.430721 -1.525283 0.907124
C -2.914051 -0.942725 -0.220776
C 1.439992 0.917743 0.079285
O -0.187670 2.099191 2.454953
F -2.675838 2.138952 -4.439489
C 2.391718 1.197558 -0.953716
C 3.607544 0.552313 -0.989398
C 3.977486 -0.396071 -0.005729
C 3.054552 -0.628300 1.047064
C 1.847442 0.026072 1.125175
O -3.731369 -1.883890 3.130196
C -4.696209 -2.634075 2.382239
O -4.324082 -2.570101 1.000300
C 5.311263 -1.140341 -0.029742
C 6.179477 -0.759254 -1.243467
C 5.033386 -2.665217 -0.083285
C 6.100385 -0.807391 1.263243
H -0.832096 3.845086 -3.757796
H 0.490405 3.681118 -1.619870
H -3.086336 0.384526 -2.627604
H -1.834727 0.172787 3.416960
H -3.242771 -1.263401 -1.203458
H 2.139131 1.918774 -1.720419
H 4.295465 0.798582 -1.788357
H 3.308934 -1.328549 1.834948
H 1.192071 -0.151268 1.966144
H -4.682352 -3.674251 2.713796
H -5.690873 -2.187989 2.515072
H 6.442805 0.304261 -1.240898
H 7.113510 -1.329494 -1.212919
H 5.681681 -0.991234 -2.191516
H 4.458840 -3.010560 0.781890
H 5.984564 -3.208916 -0.091892
H 4.479079 -2.932695 -0.989521
H 7.055527 -1.344242 1.255286
H 6.312042 0.265346 1.329589
H 5.557965 -1.104054 2.166295

34 Energy:-1306.83558386
C -2.001010 1.978541 -3.321786
C -1.042216 2.967593 -3.110861
C -0.309685 2.906126 -1.922578
C -0.546717 1.889789 -1.003560

C -1.597733 0.941972 -1.168719
C -2.295986 0.992394 -2.394664
N 0.309394 1.675784 0.146431
C -0.498787 1.472940 1.415823
C -1.509135 0.486376 1.273463
C -1.967152 0.096395 -0.045137
C -2.124314 -0.065634 2.447056
C -3.055585 -1.044712 2.267127
C -3.434712 -1.494134 0.982206
C -2.926352 -0.945194 -0.166184
C 1.441712 0.909514 0.027992
O -0.158031 2.153987 2.394891
F -2.705135 1.999380 -4.479220
C 2.462623 1.299850 -0.894714
C 3.683458 0.660445 -0.916200
C 3.985171 -0.392494 -0.022472
C 2.988589 -0.739516 0.927889
C 1.778129 -0.089374 0.994752
O -3.716115 -1.791546 3.217033
C -4.690581 -2.557768 2.498628
O -4.332038 -2.531110 1.111644
C 5.321016 -1.133436 -0.031771
C 6.277325 -0.614884 -1.121962
C 5.055553 -2.640084 -0.286474
C 6.007207 -0.958960 1.348020
H -0.869245 3.736499 -3.854596
H 0.461791 3.642365 -1.719375
H -3.098680 0.297650 -2.615046
H -1.806020 0.262186 3.430051
H -3.266282 -1.291715 -1.136148
H 2.263323 2.108902 -1.586254
H 4.427126 0.997770 -1.627048
H 3.186463 -1.529167 1.644373
H 1.060156 -0.362852 1.755813
H -4.676545 -3.589013 2.856983
H -5.682545 -2.105190 2.629265
H 6.532741 0.440301 -0.974430
H 7.209944 -1.187205 -1.086787
H 5.855146 -0.732640 -2.126103
H 4.415524 -3.081996 0.483530
H 6.006867 -3.183616 -0.283399
H 4.576317 -2.796048 -1.259114
H 6.962878 -1.494878 1.349614
H 6.207676 0.097548 1.556595
H 5.398995 -1.358052 2.165687

35 Energy:-1306.83592743
C -2.034438 1.863269 -3.361921

C -1.059483 2.845208 -3.197233
C -0.320497 2.819014 -2.012059
C -0.567103 1.846489 -1.048485
C -1.632854 0.906097 -1.170999
C -2.336213 0.918025 -2.394752
N 0.305597 1.657645 0.090908
C -0.478275 1.469551 1.374814
C -1.509072 0.501508 1.278827
C -1.998233 0.092907 -0.022326
C -2.111016 -0.013161 2.475984
C -3.063761 -0.978017 2.334652
C -3.474372 -1.447809 1.066743
C -2.977589 -0.933423 -0.102948
C 1.458215 0.903441 -0.028231
O -0.112999 2.180591 2.323812
F -2.745410 1.847235 -4.514348
C 2.544671 1.413655 -0.798089
C 3.771892 0.780478 -0.805125
C 4.006988 -0.382142 -0.040290
C 2.938300 -0.851714 0.768213
C 1.720824 -0.210579 0.821853
O -3.720063 -1.691488 3.312147
C -4.719320 -2.457853 2.628556
O -4.387341 -2.464687 1.234499
C 5.346471 -1.118206 -0.035896
C 6.383109 -0.463960 -0.968180
C 5.117974 -2.578876 -0.503808
C 5.916782 -1.124536 1.406105
H -0.880196 3.581359 -3.971908
H 0.463269 3.550760 -1.844421
H -3.145819 0.222949 -2.586408
H -1.767466 0.328393 3.445721
H -3.342275 -1.295670 -1.057971
H 2.396823 2.314215 -1.381856
H 4.569173 1.211537 -1.397503
H 3.081192 -1.734001 1.382701
H 0.941518 -0.581631 1.474768
H -4.713017 -3.481720 3.007662
H -5.701862 -1.988109 2.768603
H 6.617722 0.562092 -0.663794
H 7.314408 -1.038556 -0.933488
H 6.043424 -0.447577 -2.009741
H 4.422548 -3.116643 0.148207
H 6.071275 -3.119021 -0.494107
H 4.719900 -2.607450 -1.524018
H 6.874031 -1.657723 1.417719
H 6.089805 -0.104063 1.764866
H 5.247285 -1.624715 2.112809

36 Energy:-1306.8857163
C -2.002447 1.131594 -3.364123
C -0.629450 0.876717 -3.511281
C 0.072032 0.430605 -2.389836
C -0.576736 0.248564 -1.151575
C -2.021020 0.458642 -1.023742
C -2.696950 0.941685 -2.186980
N 0.150663 -0.113609 -0.030374
C -0.460907 -0.499930 1.210976
C -1.889593 -0.350734 1.316361
C -2.670472 0.169536 0.198044
C -2.500497 -0.693163 2.542215
C -3.859235 -0.516636 2.649204
C -4.634658 -0.001810 1.575033
C -4.092338 0.340974 0.375216
C 1.588731 -0.189335 -0.090766
O 0.282009 -0.916078 2.107897
F -2.679161 1.579526 -4.442154
C 2.344669 0.978217 -0.000815
C 3.738674 0.909438 -0.038550
C 4.409601 -0.316524 -0.167315
C 3.619919 -1.477336 -0.254956
C 2.228894 -1.424588 -0.215713
O -4.671512 -0.770035 3.715911
C -6.002097 -0.409683 3.316362
O -5.943164 0.070593 1.968106
C 5.944118 -0.427199 -0.212949
C 6.633964 0.945649 -0.101349
C 6.371774 -1.075580 -1.551601
C 6.430422 -1.309564 0.961799
H -0.144434 1.025994 -4.468620
H 1.132284 0.229571 -2.466789
H -3.756790 1.158399 -2.173211
H -1.900307 -1.074282 3.359979
H -4.721492 0.726234 -0.416866
H 1.845237 1.936508 0.106791
H 4.297860 1.834349 0.041270
H 4.094634 -2.448924 -0.354293
H 1.639785 -2.333826 -0.280799
H -6.644144 -1.294620 3.362171
H -6.372507 0.384191 3.972050
H 6.395506 1.447139 0.843455
H 7.720823 0.813170 -0.139793
H 6.353032 1.612363 -0.924585
H 5.948592 -2.078143 -1.673582
H 7.463700 -1.166696 -1.595199
H 6.047572 -0.466819 -2.403566

H 7.522881 -1.401943 0.937471
H 6.147651 -0.870168 1.925231
H 6.009404 -2.319346 0.916107

37 Energy:-1306.88677465
C -2.017488 0.910657 -3.419460
C -0.618644 0.835701 -3.511000
C 0.095029 0.515659 -2.352835
C -0.569241 0.276285 -1.133740
C -2.029326 0.358170 -1.046486
C -2.722784 0.687841 -2.256072
N 0.155976 -0.041194 0.003721
C -0.442729 -0.297271 1.276311
C -1.881080 -0.214199 1.361797
C -2.678637 0.117919 0.183696
C -2.482369 -0.463263 2.611129
C -3.854216 -0.382291 2.686235
C -4.648413 -0.060111 1.552219
C -4.114696 0.187965 0.325926
C 1.596487 -0.132448 -0.054780
O 0.308545 -0.573048 2.219719
F -2.706438 1.219241 -4.539059
C 2.376625 0.994344 0.188246
C 3.769877 0.898870 0.134810
C 4.411591 -0.311896 -0.163106
C 3.595489 -1.431460 -0.408092
C 2.205823 -1.350556 -0.358939
O -4.662069 -0.579394 3.766750
C -6.008107 -0.356368 3.321520
O -5.964799 -0.054960 1.922083
C 5.943468 -0.451472 -0.225850
C 6.665170 0.879114 0.059660
C 6.358387 -0.932823 -1.636907
C 6.406505 -1.488169 0.825759
H -0.121436 1.022388 -4.455530
H 1.173929 0.450261 -2.391679
H -3.801171 0.767863 -2.282563
H -1.868967 -0.708151 3.470150
H -4.758096 0.429730 -0.509952
H 1.899586 1.940895 0.424092
H 4.350607 1.792050 0.333815
H 4.046981 -2.391277 -0.640406
H 1.594380 -2.227691 -0.548396
H -6.595647 -1.264963 3.482482
H -6.431472 0.493101 3.866617
H 6.438459 1.260107 1.061857
H 7.748647 0.727497 0.001309
H 6.399832 1.652607 -0.670062

H 5.913668 -1.902054 -1.885834
H 7.448077 -1.041763 -1.693385
H 6.048952 -0.213114 -2.403617
H 7.496412 -1.603319 0.788203
H 6.132961 -1.168710 1.837883
H 5.960888 -2.473213 0.652029

12.3.6 Computed CT emission energies of compound 5b (B3LYP/6-31G* PCM CH_2Cl_2)

Excitation energies and oscillator strengths:

Excited State 1: Singlet-A 1.9690 eV 629.67 nm f=0.0554 <S**2>=0.000

102 ->103 0.70488

This state for optimization and/or second-order correction.

Total Energy, E(TD-HF/TD-DFT) = -1306.76929579

Copying the excited state density for this state as the 1-particle RhoCI density.

Excited State 2: Singlet-A 3.0485 eV 406.71 nm f=0.0681 <S**2>=0.000

99 ->103 0.15282

101 ->103 0.66918

102 ->104 0.10914

Excited State 3: Singlet-A 3.2743 eV 378.65 nm f=0.0078 <S**2>=0.000

102 ->104 0.69383

Excited State 4: Singlet-A 3.5890 eV 345.46 nm f=0.0464 <S**2>=0.000

96 ->103 0.17227

97 ->103 0.11841

99 ->103 0.36932

100 ->103 0.51428

101 ->103 -0.16394

Excited State 5: Singlet-A 3.7203 eV 333.26 nm f=0.0136 <S**2>=0.000

99 ->103 0.51184

100 ->103 -0.45644

Excited State 6: Singlet-A 3.9564 eV 313.38 nm f=0.0036 <S**2>=0.000

98 ->103 0.56397

101 ->104 0.41023

12.3.7 Computed excitations energies of the triplet state of compound 5b (B3LYP/6-31G* PCM CH_2Cl_2)

Excitation energies and oscillator strengths:

```
Excited State   1:      Triplet-A      2.8748 eV  431.28 nm  f=0.0000  <S**2>=2.000
      99 ->104         0.14994
     101 ->103         0.14427
     102 ->103         0.63410
     102 ->107        -0.10706
 This state for optimization and/or second-order correction.
 Total Energy, E(TD-HF/TD-DFT) =  -1306.78835260
 Copying the excited state density for this state as the 1-particle RhoCI density.

 Excited State   2:      Triplet-A      3.3121 eV  374.34 nm  f=0.0000  <S**2>=2.000
      99 ->103         0.17312
     101 ->103        -0.38329
     101 ->107        -0.15397
     102 ->103         0.19149
     102 ->104         0.46768
     102 ->107         0.10703

 Excited State   3:      Triplet-A      3.4561 eV  358.74 nm  f=0.0000  <S**2>=2.000
     101 ->103         0.49241
     101 ->104         0.12155
     102 ->103        -0.12390
     102 ->104         0.45442

 Excited State   4:      Triplet-A      3.7144 eV  333.80 nm  f=0.0000  <S**2>=2.000
      97 ->105        -0.10771
      98 ->105        -0.15338
      98 ->106        -0.39864
     100 ->105         0.51303
     100 ->106        -0.13252

 Excited State   5:      Triplet-A      3.8256 eV  324.09 nm  f=0.0000  <S**2>=2.000
      96 ->103        -0.15342
      96 ->104         0.16597
      99 ->103        -0.30579
     101 ->103        -0.19342
     101 ->104         0.18551
     101 ->107         0.33230
     102 ->104         0.15549
     102 ->107        -0.23261
```

```
     102 ->108        -0.19488

 Excited State   6:      Triplet-A      3.8597 eV  321.23 nm  f=0.0000  <S**2>=2.000
      99 ->104         0.36368
      99 ->108        -0.10846
     101 ->103        -0.10787
     101 ->104         0.51543
     102 ->103        -0.11992

 Excited State   7:      Triplet-A      4.1843 eV  296.31 nm  f=0.0000  <S**2>=2.000
      96 ->103        -0.10625
      96 ->104        -0.12403
      99 ->103        -0.35586
      99 ->104         0.40757
     101 ->103        -0.10857
     101 ->104        -0.23176
     101 ->108        -0.11184
     102 ->104         0.10196
     102 ->107         0.20461

 Excited State   8:      Triplet-A      4.2759 eV  289.96 nm  f=0.0000  <S**2>=2.000
      96 ->104         0.15830
      99 ->103        -0.33192
      99 ->104        -0.17798
     101 ->104         0.22615
     101 ->107        -0.26201
     102 ->107         0.37937
     102 ->108        -0.11604

 Excited State   9:      Triplet-A      4.3307 eV  286.29 nm  f=0.0000  <S**2>=2.000
      97 ->103         0.43844
      97 ->104         0.36966
      97 ->109        -0.11466
     100 ->103         0.31606
     100 ->104         0.19079

 Excited State  10:      Triplet-A      4.5351 eV  273.39 nm  f=0.0000  <S**2>=2.000
      96 ->103         0.42727
      98 ->103         0.14611
      99 ->103        -0.29425
      99 ->107         0.11494
      99 ->108        -0.13978
     102 ->107        -0.28532
     102 ->108         0.20094

 Excited State  11:      Triplet-A      4.6124 eV  268.81 nm  f=0.0000  <S**2>=2.000
      97 ->103        -0.13972
      97 ->106        -0.10555
```

98 ->105 -0.28153
98 ->106 0.10865
100 ->103 0.33411
100 ->104 -0.11777
100 ->105 0.11394
100 ->106 0.46984

Excited State 12: Triplet-A 4.6536 eV 266.43 nm f=0.0000 <S**2>=2.000
98 ->103 0.19209
98 ->105 0.19791
98 ->106 0.44766
100 ->105 0.40988
100 ->106 -0.12368

Excited State 13: Triplet-A 4.7495 eV 261.05 nm f=0.0000 <S**2>=2.000
101 ->105 -0.23359
102 ->105 0.64363
102 ->106 -0.10105

Excited State 14: Triplet-A 4.8493 eV 255.68 nm f=0.0000 <S**2>=2.000
97 ->103 -0.19459
97 ->104 -0.16307
98 ->105 0.29582
98 ->106 -0.13847
100 ->103 0.50671
100 ->105 -0.10485
100 ->106 -0.19787

Excited State 15: Triplet-A 4.8793 eV 254.10 nm f=0.0000 <S**2>=2.000
96 ->104 0.10898
96 ->107 0.12555
99 ->104 0.13359
99 ->107 0.13218
101 ->104 -0.17000
101 ->107 -0.33492
102 ->106 0.36368
102 ->107 -0.30732
102 ->108 -0.17983

12.3.8 Computed emission energies of the triplet state of compound 5b (B3LYP/6-31G* PCM CH_2Cl_2)

Excited State 1: Triplet-A 2.2453 eV 552.19 nm f=0.0000 <S**2>=2.000
102 ->103 0.67851
This state for optimization and/or second-order correction.
Total Energy, E(TD-HF/TD-DFT) = -1306.79944341
Copying the excited state density for this state as the 1-particle RhoCI density.

```
Excited State   2:      Triplet-A      3.1369 eV  395.25 nm  f=0.0000  <S**2>=2.000
     99 ->103       -0.14501
    101 ->103        0.53884
    102 ->103       -0.10843
    102 ->104       -0.38781

Excited State   3:      Triplet-A      3.2755 eV  378.53 nm  f=0.0000  <S**2>=2.000
    101 ->103        0.41372
    102 ->104        0.55414

Excited State   4:      Triplet-A      3.6742 eV  337.44 nm  f=0.0000  <S**2>=2.000
     96 ->103        0.19101
     96 ->104       -0.10044
     99 ->103        0.50782
    101 ->103        0.10958
    101 ->104       -0.14693
    101 ->107       -0.21653
    102 ->104       -0.14118
    102 ->108        0.20421

Excited State   5:      Triplet-A      3.7112 eV  334.08 nm  f=0.0000  <S**2>=2.000
     97 ->105       -0.12091
     98 ->105       -0.15199
     98 ->106       -0.39617
    100 ->105        0.50849
    100 ->106       -0.12577

Excited State   6:      Triplet-A      3.9561 eV  313.40 nm  f=0.0000  <S**2>=2.000
     96 ->104       -0.10561
     99 ->104        0.35312
    101 ->104        0.50464
    102 ->107       -0.18594

Excited State   7:      Triplet-A      4.1064 eV  301.93 nm  f=0.0000  <S**2>=2.000
     96 ->103        0.14601
     96 ->104       -0.13968
     99 ->103       -0.33004
     99 ->104        0.25573
    101 ->104       -0.11355
    101 ->107       -0.26294
    102 ->107        0.35586
    102 ->108        0.18730

Excited State   8:      Triplet-A      4.1730 eV  297.11 nm  f=0.0000  <S**2>=2.000
     97 ->103        0.41574
     97 ->104        0.28149
    100 ->103        0.44921
    100 ->104        0.15627
```

Excited State 9: Triplet-A 4.2918 eV 288.89 nm f=0.0000 <S**2>=2.000
95 ->103 0.11790
96 ->103 -0.28724
99 ->103 0.16372
101 ->104 0.21431
101 ->107 -0.18058
102 ->106 0.16165
102 ->107 0.42267
102 ->108 -0.22493

Excited State 10: Triplet-A 4.3502 eV 285.01 nm f=0.0000 <S**2>=2.000
96 ->103 -0.34793
96 ->104 -0.20643
98 ->103 -0.15128
99 ->103 0.15503
99 ->104 0.35354
99 ->107 -0.10530
101 ->104 -0.23937
101 ->107 0.17509

Excited State 11: Triplet-A 4.4603 eV 277.97 nm f=0.0000 <S**2>=2.000
97 ->103 -0.37023
97 ->104 -0.19032
100 ->103 0.49685
100 ->106 0.21062

Excited State 12: Triplet-A 4.5743 eV 271.04 nm f=0.0000 <S**2>=2.000
101 ->105 -0.14913
102 ->105 0.67556
102 ->106 -0.10096

Excited State 13: Triplet-A 4.6115 eV 268.86 nm f=0.0000 <S**2>=2.000
98 ->103 0.39968
98 ->105 0.18197
98 ->106 0.34516
100 ->105 0.34901
100 ->106 -0.15038

Excited State 14: Triplet-A 4.7107 eV 263.20 nm f=0.0000 <S**2>=2.000
97 ->103 0.11550
97 ->106 -0.12282
98 ->105 -0.35524
98 ->106 0.15577
100 ->103 -0.19658
100 ->105 0.16448
100 ->106 0.44717
102 ->106 0.12167

Excited State 15: Triplet-A 4.7159 eV 262.91 nm f=0.0000 <S**2>=2.000

99 ->104	0.12670
99 ->107	0.13844
100 ->106	-0.11784
101 ->104	-0.17620
101 ->107	-0.29970
102 ->106	0.38691
102 ->107	-0.32130
102 ->108	-0.12976

13 References

1 L. Liang, J. Li, B. Shen, Y. Zhang, J. Liu, J. Chen, D. Liu, *The effect of carbonyl on the isomerization of a galanthan ring system and total synthesis of (±)-β-lycorane*, *Org. Biomol. Chem.* **2021**, *19*, 2767-2772, DOI: 10.1039/D0OB02398A.

2 M. Yang, Z. Xing, B. Fang, X. Xie, X. She, *Visible light photoredox catalyzed deprotection of 1,3-oxathiolanes*, *Org. Biomol. Chem.* **2020**, *18*, 288-291, DOI: 10.1039/c9ob02517k.

3 C. A. Moore, B. F. Ohman, M. J. Garman, M. E. Liquori, D. M. Degan, K. B. Voellinger, M. J. DePersis, E. T. Pelkey, *Investigating the stereochemical outcome of a tandem cyclization-coupling reaction leading to a 3-arylmethylideneisobenzofuran-1-one*, *Arkivoc* **2018**, 50-69, DOI: 10.24820/ark.5550190.p010.480.

4 U. V. Mentzel, D. Tanner, J. E. Tønder, *Comparative study of the Kumada, Negishi, Stille, and Suzuki-Miyaura reactions in the synthesis of the indole alkaloids hippadine and pratosine*, *J. Org. Chem.* **2006**, *71*, 5807-5810, DOI: 10.1021/jo060729b.

5 S. K. Bera, P. Mal, *Mechanochemical-Cascaded C–N Cross-Coupling and Halogenation Using N-Bromo- and N-Chlorosuccinimide as Bifunctional Reagents*, *J. Org. Chem.* **2021**, *86*, 14144-14159, DOI: 10.1021/acs.joc.1c01742.

6 J. V. Suárez-Meneses, A. Oukhrib, M. Gouygou, M. Urrutigoïty, J.-C. Daran, A. Cordero-Vargas, M. C. Ortega-Alfaro, J. G. López-Cortés, *[N,P]-pyrrole $PdCl_2$ complexes catalyzed the formation of dibenzo-α-pyrone and lactam analogues*, *Dalton Trans.* **2016**, *45*, 9621-9630, DOI: 10.1039/C6DT01022A.

7 D. Nageswar Rao, S. Rasheed, P. Das, *Palladium/Silver synergistic catalysis in direct aerobic carbonylation of C(sp^2)−H bonds using DMF as a carbon source: Synthesis of pyrido-fused quinazolinones and phenanthridinones*, *Org. Lett.* **2016**, *18*, 3142-3145, DOI: 10.1021/acs.orglett.6b01292.

8 Y. Yang, H. Huang, L. Wu, *Palladium-catalyzed annulation of benzynes with N-substituted-N-(2-halophenyl)formamides: synthesis of phenanthridinones*, *Org. Biomol. Chem.* **2014**, *12*, 5351-5355, DOI: 10.1039/C4OB00997E.

9 Y. Kuwata, M. Sonoda, S. Tanimori, *Facile Synthesis of Phenanthridinone Alkaloids via Suzuki–Miyaura Cross‐coupling*, *J. Heterocycl. Chem.* **2017**, *54*, 1645-1651, DOI: 10.1002/jhet.2725.

10 J. Mooney, P. Kambhampati, *Get the basics right: Jacobian conversion of wavelength and energy scales for quantitative analysis of emission spectra*, *J. Phys. Chem. Lett.* **2013**, *4*, 3316-3318, DOI: 10.1021/jz401508t.

11 J. S. Swenton, T. J. Ikeler, G. L. Smyser, *Effect of biphenyl geometry and substituents on the multiplicity and efficiency of the photocyclization reactions of 2-substituted biphenyls*, *J. Org. Chem* **1973**, *38*, 1157-1166, DOI: 10.1021/jo00946a021.
